# Systematic Analysis of Molecular Subtypes Based on the Expression Profile of Immune-Related Genes in Pancreatic Cancer

**DOI:** 10.1155/2022/3124122

**Published:** 2022-12-15

**Authors:** Mujing Ke

**Affiliations:** Department of Ultrasound, Xiangya Hospital, Central South University, Changsha, Hunan 410008, China

## Abstract

Immunotherapy has a good therapeutic effect and provides a new approach for cancer treatment. However, only limited studies have focused on the use of molecular typing to construct an immune characteristic index for gene expression in pancreatic adenocarcinoma (PAAD) and to assess the effectiveness of immunotherapy in patients with PAAD. Clinical follow-up data and gene expression profile of PAAD patients were retrieved from The Cancer Genome Atlas (TCGA) database. Based on 184 immune features, molecular subtypes of pancreatic cancer were found by the “ConsensusClusterPlus” package, and the association between clinical features and immune cell subtype distribution was analysed. In addition, the relationship between the proportion of immune subtypes and the expression of immune checkpoints was analysed. The CIBERSORT algorithm was introduced to evaluate the immune scores of different molecular subtypes. We used the tumor immune dysfunction and exclusion (TIDE) algorithm to assess the potential clinical effect of immunotherapy interventions on single-molecule subtypes. In addition, the oxidative stress index was constructed by linear discriminant analysis DNA (LDA), and weighted correlation network analysis was performed to identify the core module of the index and its characteristic genes. Expression of hub genes was verified by immunohistochemical analysis in an independent PAAD cohort. Pancreatic cancer is divided into three molecular subtypes (IS1, IS2, and IS3), with significant differences in prognosis between multiple cohorts. Expression of immune checkpoint-associated genes was significantly reduced in IS3 and higher in IS1 and IS2, suggesting that the three subgroups have different responsiveness to immunotherapy interventions. The results of the CIBERSORT analysis showed that IS1 exhibited the highest levels of immune infiltration, whereas the results of the TIDE analysis showed that the T-cell dysfunction score of IS1 was higher than that of IS2 and IS3. Furthermore, IS3 was found to be more sensitive to 5-FU and to have a higher immune signature index than IS1 and IS2. Based on WGCNA analysis, 10 potential gene markers were identified, and their expression at the protein level was verified by immunohistochemical analysis. Specific molecular expression patterns in pancreatic cancer can predict the efficacy of immunotherapy and influence the prognosis of patients.

## 1. Introduction

Pancreatic cancer is a life-threatening malignancy and is ranked fourth among the major contributors to cancer-associated mortality globally [1]. The mortality rate of pancreatic cancer in China increased during 1991–2000 and is expected to peak in the future [2]. It is predicted that pancreatic cancer could become the second greatest cause of mortality due to cancer by the year 2030 [3]. In contrast with other malignancies, pancreatic cancer is asymptomatic until patients are at an advanced stage. Therefore, surgery is the only effective treatment modality, which may be followed by adjuvant chemotherapy with gemcitabine or the oral fluoropyrimidine derivative S-1. Patients who are not candidates for surgery but are in good physical condition are ideal candidates for FOLFIRINOX (fluorouracil, folic acid, irinotecan, and oxaliplatin) and gemcitabine combined with albumin-bound paclitaxel [4–6]. Although surgical resection, chemotherapy, and antivascular therapy have been widely used, their efficacy remains limited. Patients who have been diagnosed with pancreatic cancer have a dismal long-term prognosis, with a median survival time of lower than six months and a five-year survival probability of <5% [7, 8].

Recently, immune checkpoint treatments targeting programmed cell death 1/programmed cell death-ligand 1 (PD-1/PD-L1) and cytotoxic T-lymphocyte-associated antigen 4 (CTLA-4) have been rapidly developed as therapeutic approaches for cancer. Pancreatic cancer has been shown to be among the most immunotolerant types of tumours, with clinical trials showing unsatisfactory results and poor response to PD-1/PD-L1 blockade monotherapy [9, 10]. Ipilimumab (anti-CTLA-4) administered at a dosage of 3.0 mg/kg in a phase-2 clinical trial was ineffective in treating either locally advanced or metastatic pancreatic cancer [11]. These unsatisfactory results can be attributed to several reasons; however, the immunosuppressive tumour microenvironment, which is characterized by decreased mutation load, prominent myeloid inflammation, and inadequate infiltration of effector T cells, is the primary explanation for this phenomenon [12–14]. A small proportion of individuals diagnosed with pancreatic cancer has been shown in a few trials to have substantial T-cell infiltration and long overall survival duration, indicating the potential of immunotherapy for the successful treatment of pancreatic cancer [15, 16]. Mirlekar et al. stated that B cells are emerging candidates for pancreatic cancer therapy; in particular, B cells producing interleukin- (IL-) 35 play a major role in pancreatic tumorigenesis [17–19].

Furthermore, a few pancreatic cancer patients may exhibit a high antigen load, indicating that PD-1 inhibitors are also effective therapeutic agents [20, 21]. As a consequence, it is essential to discover reliable biological markers to enhance the effectiveness of immune checkpoint inhibitors in pancreatic cancer therapy.

The expanded public genomic data provide an ideal resource for conducting large-scale immunoassays. To date, many immune markers for pancreatic cancer have been identified using such databases. Zhang et al. identified immune markers of prognostic value based on PD-L2 expression, which can be combined with tumour-infiltrating cells to predict PAAD patients' survival after surgery [22]. Bu et al. identified eight markers premised on immune-associated genes to predict the prognostic characteristics of pancreatic cancer patients [23]. Therefore, using a combination of bioinformatic algorithms, potential therapeutic targets can be predicted to enhance the effectiveness of immunotherapeutic regimens.

In this research, the immune characteristics of PAAD were examined using a single-sample gene set enrichment analysis (ssGSEA) premised on the expression levels of 184 marker genes associated with immune cells. Based on the immune scores, PAAD was divided into different immune subtypes (ISs). Subsequently, the correlation between different ISs and immune checkpoint expression was analysed, and the immune characteristic index of each sample was evaluated via linear discriminant analysis (LDA) to assess the patients' immune features. This new method can help determine the patients' responsiveness to immunotherapy and predict the efficacy of immunotherapy for pancreatic cancer.

## 2. Materials and Methods

### 2.1. Data Acquisition and Preprocessing

The RNA sequencing (RNA-seq) data of The Cancer Genome Atlas- (TCGA-) PAAD cohort, which contained 177 samples, were extracted from TCGA GDC API. The International Cancer Genome Consortium (ICGC) was searched to retrieve the gene expression patterns and clinical follow-up data of 237 samples included in the database. Additionally, the GSE71729 dataset with survival data was extracted from the Gene Expression Omnibus (GEO) database, which contained 125 samples. Information regarding the immunocytologic features was derived from a previous study [24].

### 2.2. Preprocessing of the RNA-seq Data of the TCGA-PAAD Cohort

1. Primary pancreatic cancer samples were extracted, and samples without clinical data were eliminated
2. Ensemble Gene (ENSG) IDs were matched to the corresponding gene symbols, and 25,554 gene expression profiles were retrieved

### 2.3. Preprocessing of Data from the GSE71729 Dataset

1. Standardised datasets were downloaded from the GEO database
2. Primary pancreatic cancer samples were extracted, samples without clinical data were removed, and 18,007 gene expression profiles were acquired

### 2.4. Preprocessing of the ICGC Data

1. Any probes that had null results for gene detection were eliminated
2. The probe was aligned with the human genome
3. Samples without clinical data were removed, and 23,294 gene expression profiles were identified

The preprocessed clinical information form is shown in Table 1.

### 2.5. Identification of ISs and Immune Gene Modules

“ConsensusClusterPlus” package in R was utilized to build a consistency matrix, which was then used to categorise samples according to the results of a consensus clustering [25]. The ISs of samples were identified using the normalised enrichment scores of the selected immune features. The PAM algorithm and the Canberra distance were utilised to conduct a total of 500 bootstraps, each of which comprised 80% of the patients who were included in the training set To determine the best classification, the cluster number was set between 2 and 10, and we analysed the consistency matrix as well as the consistency cumulative distribution function.

### 2.6. Immunophenotyping of Chemokines and Immune Checkpoint-Related Genes

Chemokines and receptors perform an integral function in the onset and progression of tumours. They can mediate the entry of multiple immune cells into the tumour microenvironment (TME) and help T cells to infiltrate the tumour, thereby affecting tumour immunity and therapeutic efficacy. In this study, immunophenotyping was performed to assess whether the three ISs of PAAD had differentially expressed chemokines and chemokine receptors. CD8+ T cells in the TME may release interferon-gamma (IFN-*γ*), which can stimulate the upmodulation of *IDO1* genes and PD-1/PD-L1 [26, 27]. Studies have shown that *IDO1* upregulation has a positive link to unfavourable prognosis, tumour progression, and metastasis [28, 29]. Data on Th1/IFN-*γ* gene signatures were extracted from a previous study, and the ssGSEA was used to compute each patient's IFN-*γ* scores. In addition, variations in IFN-*γ* scores across subgroups were analysed. The cytolytic activity (CYT) score is an innovative measure of cancer immunity that is derived depending on the levels of mRNA expression of *PRF1* and *GZMA*. The tumour-specific T-cell lytic activity was assessed in each patient using the mean mRNA expression levels of *GZMA* and *PRF1* as described in a previous study [30]. Gene sets associated with angiogenesis that were retrieved from a previous research study [31] were employed to assess each patient's angiogenic score and analyse the variations in the scores among distinct groups.

### 2.7. Response of ISs to Immunotherapy/Chemotherapy

The three subtypes were compared to determine their responsiveness to chemotherapy and immunotherapy. To evaluate the possible therapeutic impacts of immunotherapy in each of the 3 types of PAAD, the TIDE algorithm (http://tide.dfci.harvard.edu/) was adopted. The higher the prediction value of TIDE, the greater the possibility of immunological evasion, implying that there is less likelihood of patients benefiting from immunotherapy. In addition, the “pRRophetic” package was used to examine how various subtypes respond to standard chemotherapy medications such as gemcitabine, cisplatin, erlotinib, and 5-FU. “pRRophetic” [32] is an R Package used for predicting clinical responsiveness to chemotherapy based on the levels of tumour gene expressions. In this particular process, the array probes will need to be remapped to the newest version of EntrezGene. The expression data of training and test sets were quantile-normalised independently and were then integrated by utilizing the empirical Bayesian approach to normalise each gene's mean and variance. We eliminated the genes that had a very low degree of variation across the samples. The rest of the genes was employed as predictors, whereas the levels of drug sensitivity (IC50) were utilised as outcome variables. In the end, the model was applied to analyse the processed and normalised and screened clinical tumour expression data to determine the drug sensitivity of each individual patient.

### 2.8. LDA and Establishment of a Characteristic Index of Immunophenotype

LDA was conducted to create a subtype classification characteristic index to enhance the quantification of the immunological features of patients in various groups. This was done in light of the fact that various subtypes have distinct molecular features. LDA is a method that may be applied for supervised dimensionality reduction, and it is often applied to a wide range of situations. In particular, Z-score LDA (Z-LDA; Z-transformation) was performed on prognostic-related immune features, and Fisher's LDA optimisation criteria were used to specify that each group's centroids were scattered as far as possible. It was discovered that the linear combination A led to the greatest impact on the interclass variance of A in comparison to the intraclass variance. The LDA model was used to determine each patient's subtype characteristic index in the TCGA dataset.

### 2.9. Determination of Coexpressed Gene Modules Based on the Immune Characterisation Index

The “WGCNA” package included in R was utilised to determine the coexpression modules of genes associated with the immune cells. Specifically, a median absolute deviation (MAD) of >50% was selected as the cut-off for gene expression profiling in the TCGA dataset. Initially, after clustering the data, a low cutoff value was adopted to choose the coexpression modules for analysis. A coexpression network conforms to a scale-free network, which means that the degree of connectivity of a node, as denoted by its logarithm log(*k*), has a negative link to the likelihood of the node's occurrence, as denoted by its logarithm log(*P*(*k*)), and the correlation coefficient is >0.85. A suitable *β*-value was decided upon to ascertain the scale-free nature of the established coexpression network. Subsequently, the expression matrix was converted into an adjacency matrix, which was then transformed into a topological matrix. The topological overlap matrix (TOM) served as the foundation for the application of the average-linkage hierarchical clustering approach, which was then employed to cluster genes in compliance with the requirements of the hybrid dynamic shear tree, and the minimal number of genes required for each gene network module was established. Additionally, the dynamic shear approach was used to identify the gene modules, and then, each module's eigengenes were computed. Finally, after clustering the modules, their adjacent modules were incorporated into a single new module.

### 2.10. Immunohistochemical (IHC) Analysis

To verify the protein expression of the 3 genes that have been discovered, tissue microarrays (TMAs) comprising 37 tissue samples from patients with PAAD and 23 samples from healthy controls were procured from Shanghai Outdo Biotech Co., Ltd. (Shanghai, China). We carried out the experiment in compliance with the International Ethical Guidelines for Biomedical Research Involving Human Subjects (CIOMS), and the Ethics Committee of the Central South University Xiangya School of Medicine, China, gave its approval to the study procedures. After being dried throughout the night at 377°C, the TMA slides were next dewaxed in xylene and desiccated in a series of increasing doses of ethanol. Antigen was extracted from the tissue sections by heating them in a microwave oven while they were within a box that was loaded with EDTA antigen repair buffer (pH 9.0), after which the tissue blocks were submerged in hydrogen peroxide at a concentration of 3% for 25 minutes to inhibit the activities of endogenous peroxidase. To attenuate nonspecific staining, the TMA slides were first sealed before being treated with bovine serum albumin (BSA) at a concentration of 3% for half an hour at ambient temperature. Thereafter, the slides were subjected to overnight incubation at 4°C with antibodies as follows: anti-EPSTI1 (1 : 50 dilution; Proteintech,11627-1-AP), anti-IFI44 (1 : 50 dilution; Proteintech, 27233-1-AP), anti-IFIH1 (1 : 100 dilution; Proteintech, 21775-1-AP), anti-OSA1 (1 : 100 dilution; Proteintech, 14955-1-AP), anti-OSA2 (1 : 50 dilution; Proteintech, 19279-1-AP), anti-OSA3 (1 : 50 dilution; Proteintech, 21915-1-AP), anti-PARP14 (1 : 100 dilution; Sigma, HPA008846), anti-UBE2L6 (1 : 250 dilution; Abcam, ab109086), anti-CMPK2 (1 : 100 dilution; Sigma, HPA041430), and anti-IFIT3 (1 : 100 dilution; Proteintech, 15201-1-AP). The tissues were subsequently washed thrice for a total of 5 minutes each wash with 0.01 mol/L of phosphate-buffered saline (PBS; pH = 7.4). The next step involved incubating the tissues with goat antirabbit horseradish peroxidase- (HRP-) labelled secondary antibody (1 : 200 dilution, Servicebio, GB23303) for 50 minutes at ambient temperature. PBS was then used to wash the tissue sections, and 3,3-diaminobenzidine (DAB) was used to stain them. The tissue slices were thereafter counterstained with Mayer's haematoxylin before being dried and fixed. The evaluation of IHC staining was performed utilising semiquantitative scoring criteria.

Three different pathologists, all of whom were blinded to the patient's clinical features, independently scored the stained sections. The percentage of positively stained cells present across all tissues was taken into account in the scoring system, as was the intensity to which these cells stained. The following criteria were used for assessing the intensity of staining: 0, negative; 1, weak; 2, moderate; 3, strong. The proportion of positively stained cells was determined based on the staining ratio as follows: 0 (<5%), 1 (5–25%), 2 (26–50%), 3 (51–75%), and 4 (>75%). The findings of the IHC staining were categorized into the following categories depending on the staining intensity, as well as the percentage of positively stained cells: grade 0–1, negative (–); grades > 1–4, weakly positive (+); grades > 4–8, moderately positive (++); grades > 8–12, strongly positive (+++).

### 2.11. Statistical Analysis

To analyse statistical data, the R software (version 3.5.3) was used. The Kaplan–Meier technique was utilised to generate survival curves, and the log-rank test was executed to examine the variations in survival rates across different groups. Cox regression models were used for univariate and multivariate analyses to determine the independent prognostic significance of risk scores combined with other clinical parameters. By applying the receiver operating characteristic (ROC) curves, the effectiveness of the risk model in the prediction of OS over one, three, and five years was evaluated. The Wilcoxon test was performed to analyse the differences across two groups, whereas differences across numerous groups were evaluated utilizing a one-way analysis of variance on ranks (ns denotes *p* > 0.05, ^∗^ denotes *p* ≤ 0.05, ^∗∗^ denotes *p* ≤ 0.01, ^∗∗∗^ denotes *p* ≤ 0.001, and ^∗∗∗∗^ denotes *p* ≤ 0.0001).

## 3. Results

### 3.1. Identification of Molecular Subtypes Premised on Immune-Associated Features

**Figure 1:**
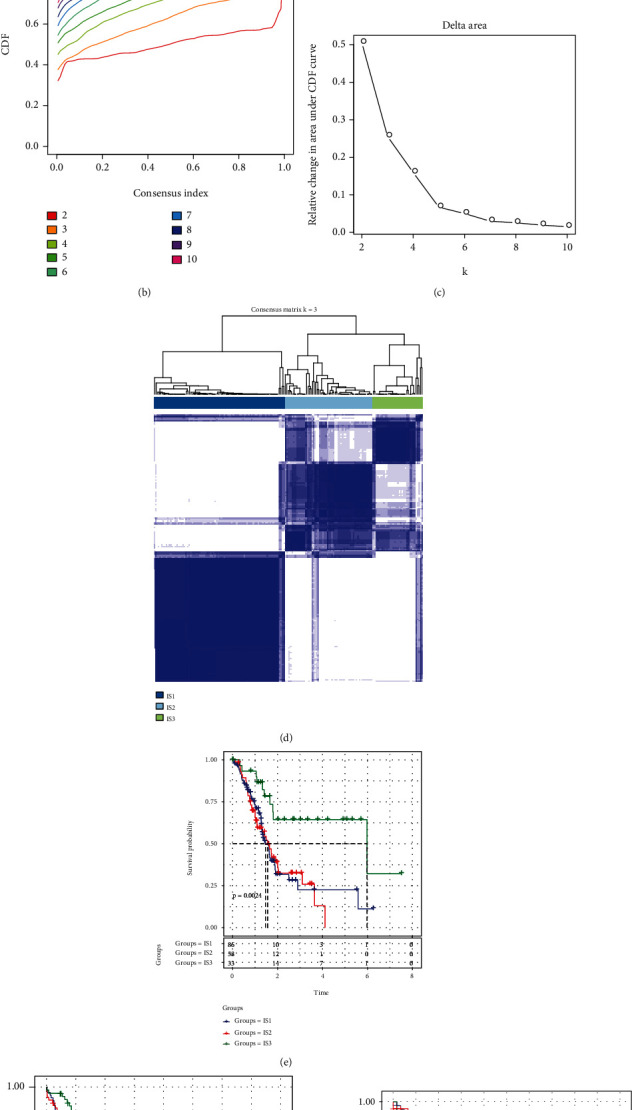
The immune subtypes of pancreatic adenocarcinoma (PAAD). (a) Venn diagram demonstrating the intersection between prognostic-associated immune characteristics in the three datasets: Gene Expression Omnibus (GEO), International Cancer Genome Consortium (ICGC), and The Cancer Genome Atlas (TCGA). (b) Cumulative distribution function (CDF) curve of TCGA cohort. (c) CDF delta area curve of TCGA cohort as well as the delta area curve of consensus clustering illustrating the relative alterations in the area under the CDF curve for each category number *k* in comparison with category number *k* − 1; the category number *k* is shown along the horizontal axis, whereas the relative change in area under the CDF curve is shown along the vertical axis. (d) Heat map illustrating sample clustering when consensus *k* is 3. (e) Kaplan–Meier (KM) curve for analysing the prognosis of the 3 immune subtypes (ISs) in TCGA cohort. (f) KM curve for analysing the prognosis of the 3 ISs in the ICGC cohort. (g) KM curve for analysing the prognosis of the 3 ISs in the GEO cohort.

First, the enrichment scores of 184 immune-associated features in GEO, ICGC, and TCGA cohorts were calculated (Supplement Table 1). The relationship between these enrichment scores and the prognosis of pancreatic cancer was analysed via univariate survival analysis, which showed that 48, 64, and 16 immune-related features were linked to prognosis in TCGA, ICGC, and GEO cohorts, correspondingly. As depicted in Figure 1(a), there was less overlap among the features, suggesting that the consistency of these features among datasets of different platforms is poor and a feature varies significantly among different cohorts. Therefore, 16 immune-related features related to prognosis identified from a minimum of two cohorts were used for further analysis.

The “ConsensusClusterPlus” function in R was adopted to perform clustering on 177 PAAD samples that were included in the TCGA cohort, and the cumulative distribution function (CDF) was utilised to determine the optimum number of clusters. From the CDF delta curve, the optimal number of clusters was 3, which resulted in highly stable clustering (Figures 1(b) and 1(c)). Consequently, three ISs (IS1, IS2, and IS3) were identified using the *k* value of 3 (Figure 1(d)). The prognostic aspects of these 3 ISs were additionally analysed, and it was shown that there are considerable differences in how well they correlate with the prognosis of patients with pancreatic cancer (Figure 1(e)). The prognosis for IS1 and IS2 was much less favourable in contrast with that of IS3. In addition, the same method was used for molecular typing of pancreatic cancer patients in the ICGC cohort. The substantial variations in prognosis that were found across the 3 ISs (Figure 1(f)) were similar to those that were seen among the ISs in the training set. The same phenomenon was observed in the GEO cohort (Figure 1(g)). From these findings, the three molecular subtypes identified based on the immune characteristic scores can be used for different cohorts.

### 3.2. Expression of Chemokines and Immune Checkpoint-Related Genes Determined via Immunophenotyping

**Figure 2:**
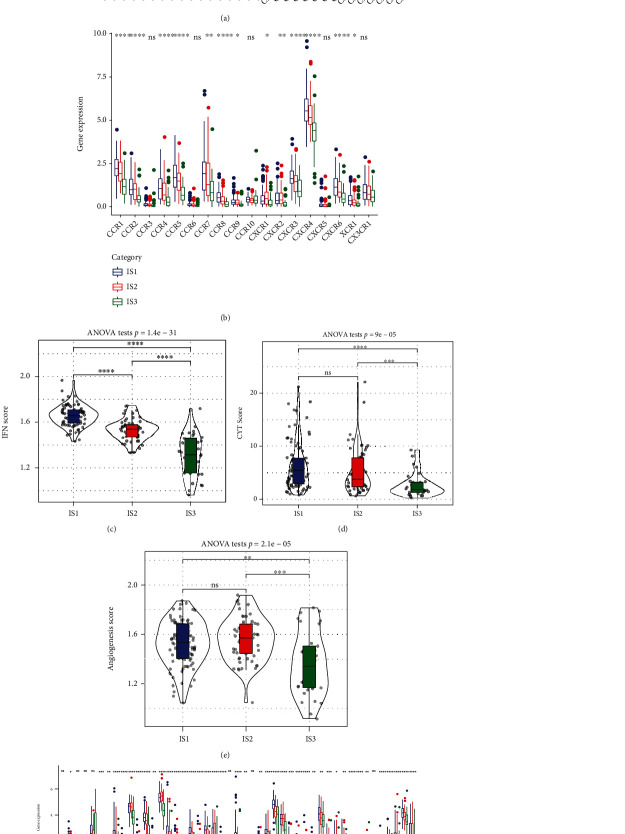
(a) Variations in the distribution and expression levels of chemokines across participants in The Cancer Genome Atlas (TCGA) cohort. (b) Variations in the distribution and expression levels of chemokine receptors in the TCGA cohort. (c) Variations in the IFN-*γ* score distribution across various subgroups in the TCGA cohort. (d) Variations in the lytic activity of the immune T cells exhibited across various subgroups. (e) Variations in angiogenesis scores recorded across various subgroups. (f) Variations in the expression and distribution of genes associated with immune checkpoints in TCGA cohort. One-way analysis of variance (ANOVA) was employed to conduct statistical tests to determine the significance level (^∗^*p* < 0.05; ^∗∗^*p* < 0.01; ^∗∗∗^*p* < 0.001; ^∗∗∗∗^*p* < 0.0001).

In the TCGA cohort, the expression of most chemokines was higher in IS1 and IS2 than in IS3 (Figure 2(a)), and IS1 and IS2 also had elevated levels of chemokine receptor expression as opposed to IS3, which had lower levels (Figure 2(b)). This finding suggested that the three immunological subtypes exhibited varying infiltration degrees of immune cells, possibly resulting in differences in tumour progression and immunotherapeutic effects.

The IFN-*γ* scores were highest in IS1 and lowest in IS3 (Figure 2(c)). In addition, it was shown that the CYT scores of the three subtypes were substantially different from one another (Figure 2(d)). The immune T-cell lytic activity was highest in IS1 and lowest in IS3.

Furthermore, there were remarkable variations in angiogenesis scores identified across the various subtypes (Figure 2(e)). The angiogenesis scores of IS1 were remarkably higher in contrast with the scores of IS2 and IS3.

Across the 3 subtypes, there were considerable variations in the expression of 40 (40/47, 85.1%) genes linked to immune checkpoints. As depicted in Figure 2(f), the expression levels of the majority of genes linked to immune checkpoints were remarkably greater in IS1 and IS2 as opposed to IS3. T-cell exhaustion marker genes, including HAVCR2, CD274, PDCD1, CTLA4, and LAG3, had substantially greater expression levels in IS1 compared to those in IS3 among these immune checkpoint-associated genes. According to the results of the CYT score evaluation, IS1 and IS2 had a high lytic activity of immune T cells but the worst prognosis, which could be attributed to T-cell exhaustion in IS1.

### 3.3. Immune and Pathway Characteristics of Different Immune Subtypes

**Figure 3:**
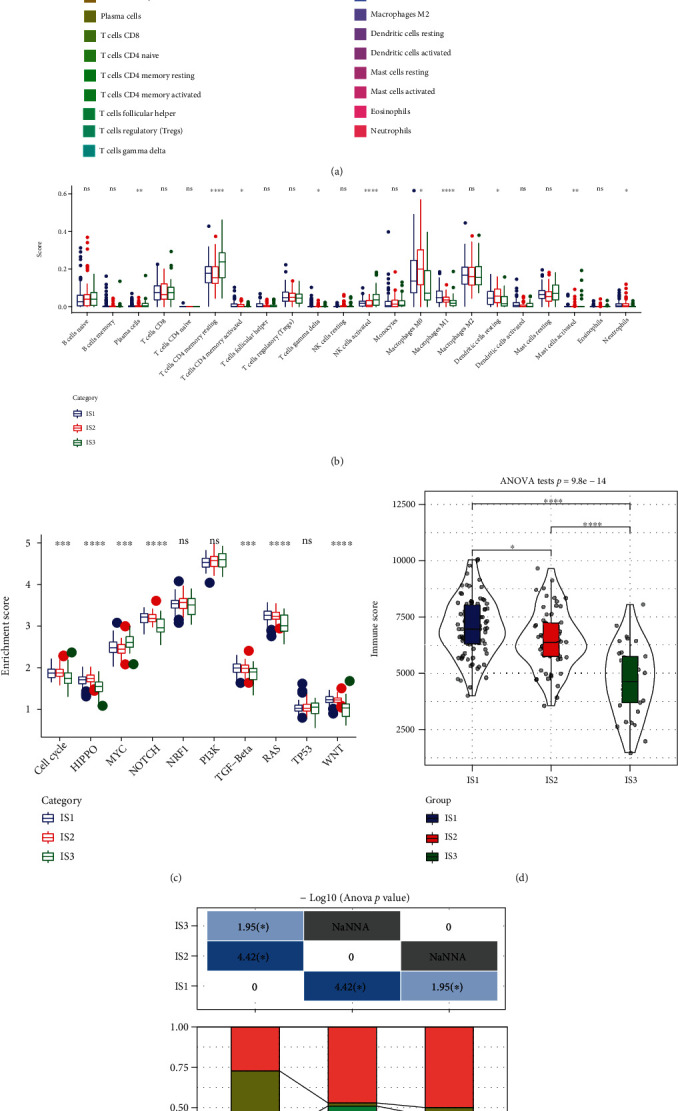
(a) Distribution of 22 distinct immune cell components in the three subtypes. (b) Variations in the distribution of 22 different immune cell components across the three subtypes. (c) Variations in the scores of the ten pathways linked to tumour aberrations across the three subtypes. (d) Variations in the infiltration levels of immune cells across the three subtypes. (e) The intersection of three immunomolecular subtypes with the subtypes reported in a previous study on pancarcinoma.

To examine the scores of 22 different kinds of immune cells present in each sample included in the TCGA dataset, the CIBERSORT method was used.  Figure 3(a) illustrates the pattern of distribution of these immune cell scores across the 3 subgroups, whereas Figure 3(b) illustrates the variations in these scores across the 3 subtypes. Based on these scores, significant differences in immune-related features were observed among different subtypes. The proportion of memory and resting CD4+ T cells, M0 macrophages, and M1 macrophages was elevated in all subtypes, thus indicating the importance of these immune cell types in pancreatic cancer. On analysing differences in the involvement of ten oncogenic pathways among the 3 subtypes as described in a previous study [33], it was observed that except for the NRF1, PI3K, and TP53 pathways, the remaining 7 pathways had significantly different enrichment scores. Among these 7 pathways, except for the MYC pathway, the enrichment scores of the remaining 6 pathways in IS1 and IS2 were substantially elevated as opposed to those in IS3. Among the 6 pathways, the enrichment score of the transforming growth factor-beta (TGF-*β*) pathway was substantially elevated in IS1 and IS2 compared to IS3 (Figure 3(c)). As per the findings of the infiltration analysis, IS1 had the highest immune cell infiltration level, followed by IS2; however, IS3 had the lowest infiltration level (Figure 3(d)). However, the expression level of most genes linked to the immune checkpoint was considerably elevated in IS1 and IS2 in contrast with that in IS3. To examine the correlation between the three ISs and six immunophenotypes reported in a previous study on pancancer, the molecular subtype data were extracted from the study for comparison [34]. Six immunophenotypes were found to have significantly different distributions across the 3 ISs (Figure 3(e)). The proportion of patients with a dismal prognosis belonging to the C2 subtype was substantially greater in IS1 in comparison to IS2 and IS3, whereas the proportion of patients with a dismal prognosis belonging to the C6 subtype was substantially greater in IS1 and IS2 in comparison to IS3, which was in line with the dismal prognosis for IS1 and IS2. Based on these findings, it appears that the 3 subtypes that have been established may be utilised as a supplement to the six subtypes that were established in the previous research.

### 3.4. The Efficacy of Chemotherapy and Immunotherapy in Treating Various Immune Subgroups

**Figure 4:**
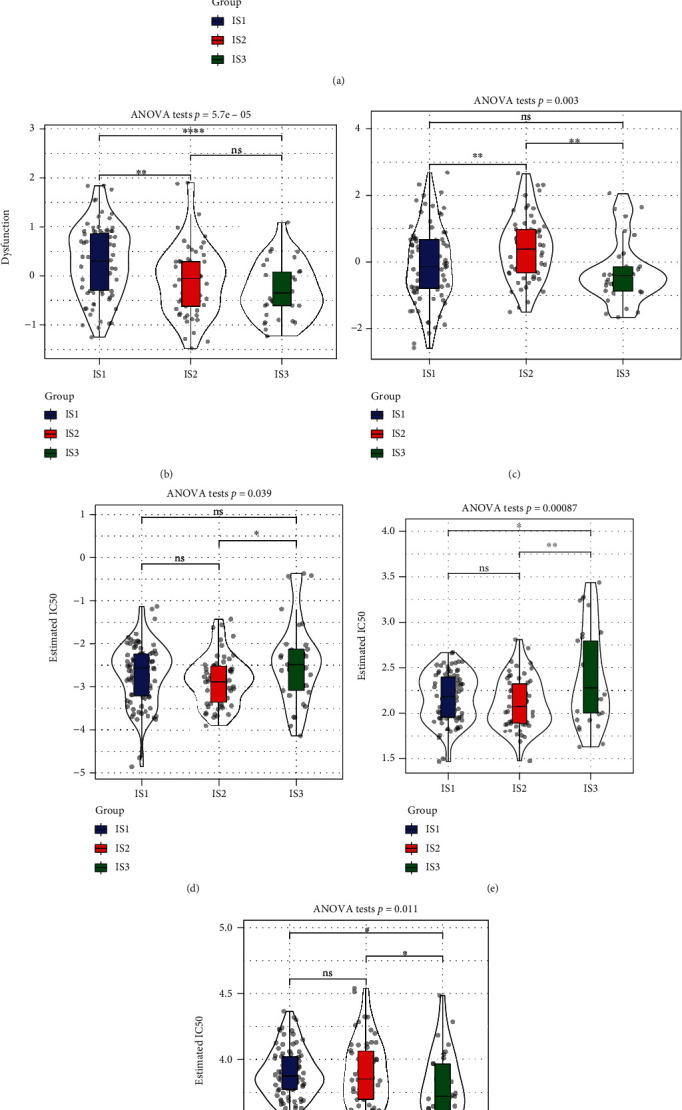
(a) Differences in TIDE scores across the three subtypes. (b) Differences in T-cell dysfunction scores across the three subtypes. (c) Differences in T-cell rejection scores across the three subtypes. The box plots demonstrate the estimated IC50 values for gemcitabine (d), erlotinib (e), and 5-Fu (f).

As shown in Figure 4(a), TIDE scores were remarkably elevated in IS1 and IS2 in contrast with IS3, suggesting that IS3 showed a better response to immunotherapy than IS1 and IS2. TIDE can also predict the response to immunotherapy (responders versus nonresponders).

Furthermore, the predicted T-cell rejection and dysfunctional scores of the three subtypes were compared (Figures 4(b) and 4(c)). The predicted T-cell dysfunction scores were highest in IS1 and were not significantly different between IS2 and IS3, which validated that although IS1 had the highest immune scores, the poor prognosis might be attributed to T-cell dysfunction. In addition, the predicted T-cell rejection scores were highest in IS2 and lowest in IS3, which is in line with the unfavourable prognosis of IS2 and the favourable prognosis of IS3. The responsiveness of various subtypes to the conventional chemotherapeutic drugs gemcitabine (Figure 4(d)), erlotinib (Figure 4(e)), and 5-FU (Figure 4(f)) was also analysed, and it was found that IS1 and IS2 were more sensitive to gemcitabine and erlotinib, whereas IS3 had a greater sensitivity to 5-FU.

### 3.5. LDA and Establishment of a Characteristic Index Based on Immunophenotypes

**Figure 5:**
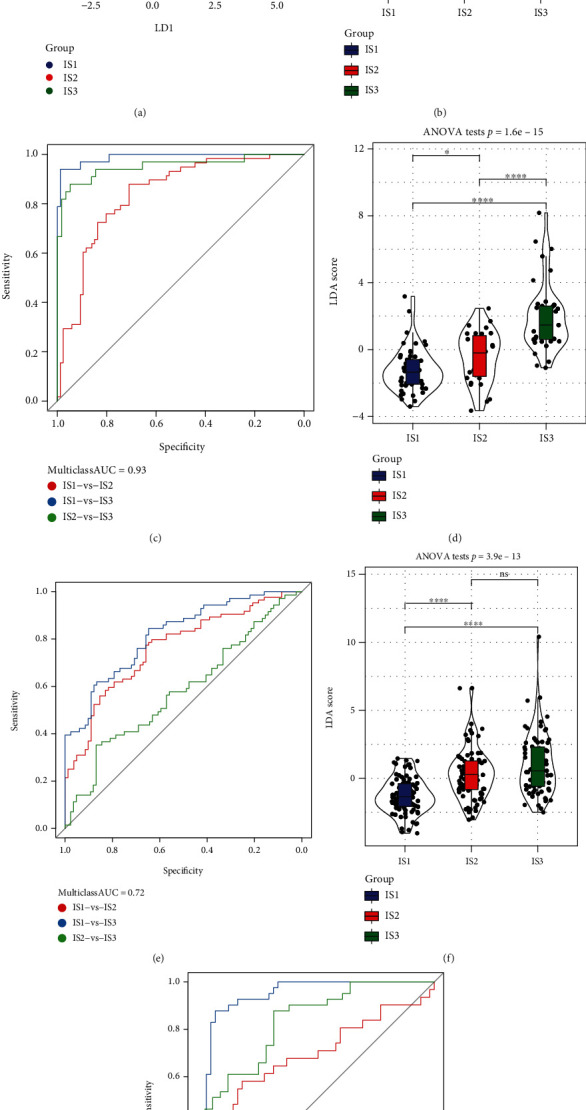
(a) Correlation between the first two features and immune characteristic index across the three immune subtypes. (b) Variations in the immune characteristic index across the three subtypes in The Cancer Genome Atlas (TCGA) cohort. (c) Receiver operating characteristic (ROC) curve of the immune characteristic index in TCGA dataset. (d) Variations in the immune characteristic index across three subtypes in the ICGC cohort. (e) ROC curve of the immune characteristic index in the ICGC cohort. (f) Differences in the immune characteristic index among the three subtypes in the GSE71729 dataset. (g) ROC curve of the immune characteristic index in the GSE71729 dataset.

LDA was carried out to develop a subtype classification characteristic index to provide an improved measurement of the immune features shared by patients within distinct groups. The first two model characteristics clearly distinguished samples of distinct subtypes (Figure 5(a)), and remarkable variations were observed in the characteristic indices among the three subtypes (Figure 5(b)). ROC curves were charted to demonstrate the effectiveness of the characteristic index in classifying various subtypes (Figure 5(c)), and the combined area under the ROC curve (AUC) of three subtypes for prediction was 0.93. When the immune subtype characteristic index was used in the ICGC dataset, the findings were comparable to those recorded in the TCGA dataset. Remarkable variations were observed in the characteristic index among the three subgroups (Figure 5(d)), and ROC analysis illustrated a combined AUC value of 0.72 (Figure 5(e)). Additionally, when the immune subtype characteristic index was used in the GEO dataset, the findings were comparable to those recorded in the TCGA dataset. Remarkable variations were observed in the characteristic index among the three subtypes (Figure 5(f)), and ROC analysis illustrated a combined AUC value of 0.81 (Figure 5(g)). These findings illustrate that the immune subtype feature index is a useful tool for evaluating the immune-related characteristics of PAAD patients. A high characteristic index indicates lower immunosuppression (IS3), whereas a lower characteristic index indicates stronger immunosuppression (IS1).

### 3.6. Coexpression Gene Module Identification Using the Immunological Characteristic Index

**Figure 6:**
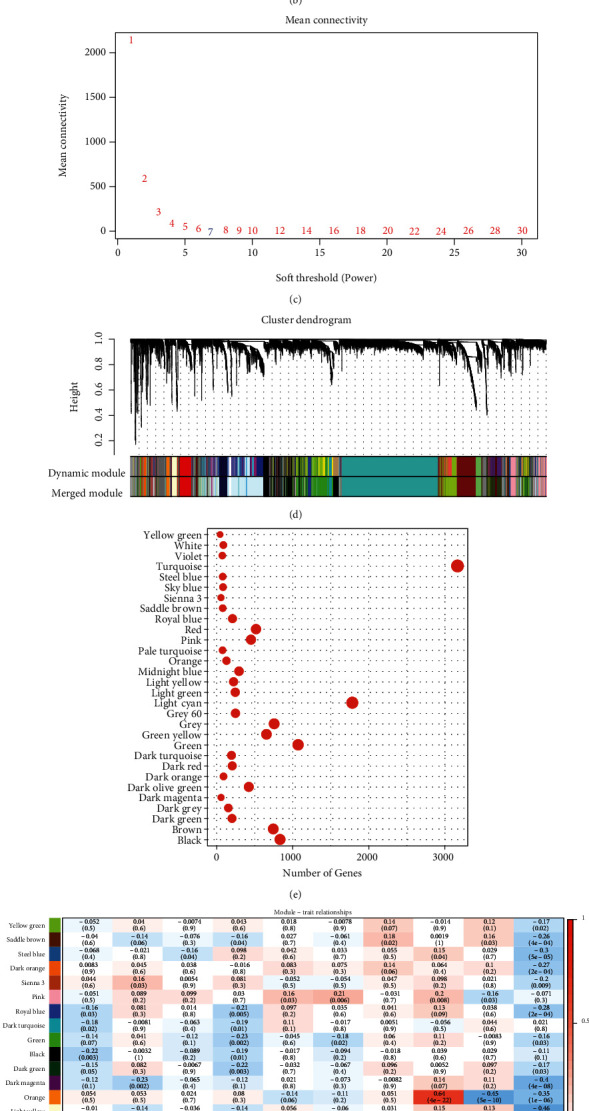
(a) Cluster tree for each sample. (b) An examination of the scale-free fit index using a variety of soft-thresholding powers (*β*). (c) An investigation of the mean connectivity at a number of different soft-thresholding powers. (d) A dendrogram showing the clustering of all remarkably expressed genes and long noncoding RNAs depending on a dissimilarity metric (1-TOM). (e) The proportion of genes included in each every module. (f) Relationship between each module and clinical characteristics.

The “WGCNA” package included in R was utilised to detect the gene coexpression modules relevant to the immune system. After clustering the samples (Figure 6(a)), we selected a soft threshold of 7 and a *β*-value of 7 to guarantee the establishment of a network that is free of scaling (Figures 6(b) and 6(c)). As per the standards established by the hybrid dynamic shear tree, at least 30 genes were set as the threshold required for each module, and the modules were subjected to cluster analysis with the following parameters: minModuleSize, 30; deepSplit, 1; height, 0.25. In total, 30 modules were acquired (Figure 6(d)). There was no clustering of the grey module with the other modules in the gene set. The statistical information on the transcripts of each module is presented in Figure 6(e) which demonstrates that the grey module could not be assigned to a gene module. Furthermore, the association of each module with age, M stage, sex, N stage, tumour stage, T stage, tumour grade, IS1, IS2, and IS3 was analysed (Figure 6(f)). A significantly positive association was found between the orange module and IS1, whereas a substantial association was observed between the orange module and IS2 and IS3, respectively.

### 3.7. Identification of the Immune Characteristic Index of the Coexpressed Gene Modules

**Figure 7:**
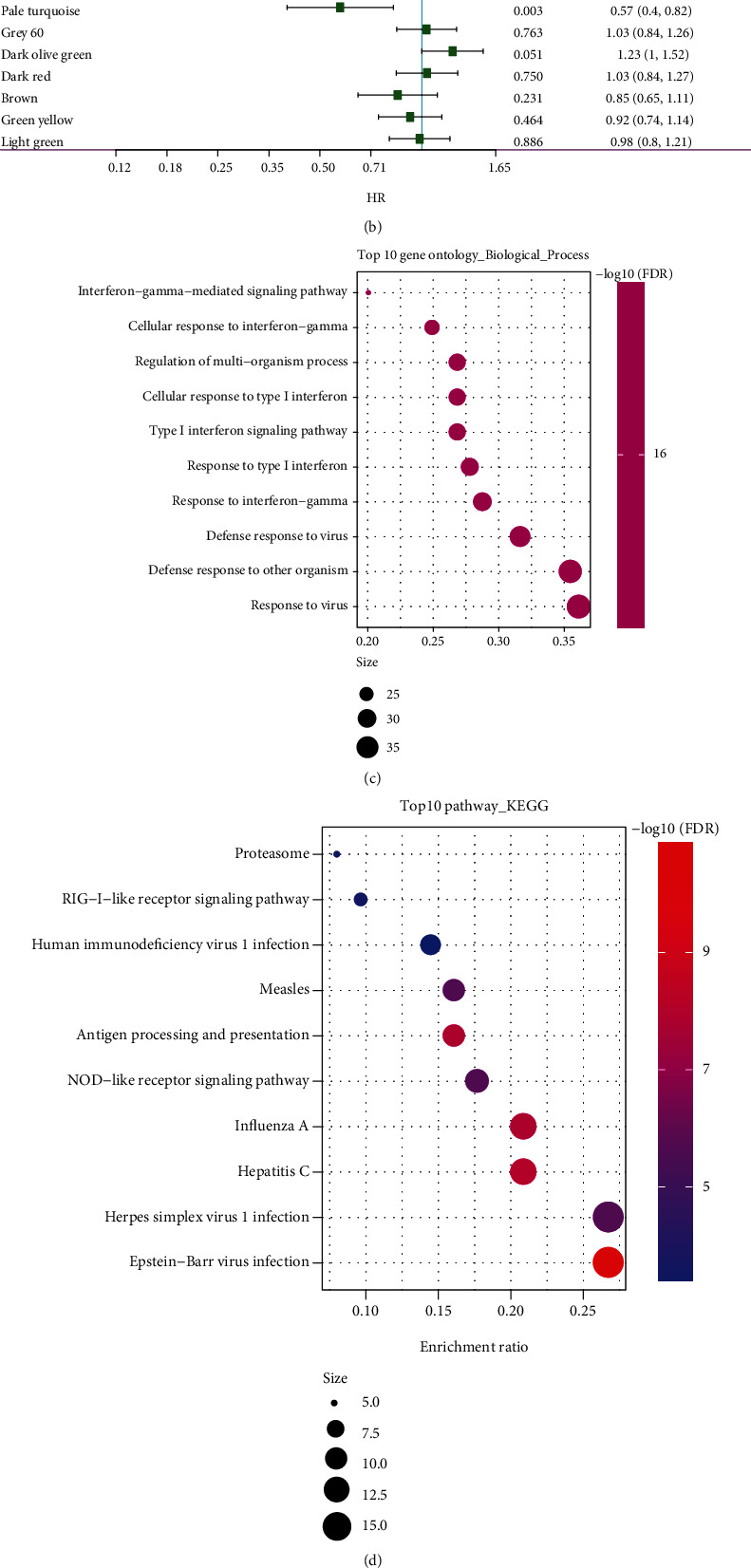
(a) Association of the module characteristic vector with immune characteristic index. (b) Correlation between prognosis and each gene module linked to the immune characteristic index. (c) The findings of the biological functional enrichment analysis regarding the orange module. (d) The findings of the biological functional enrichment analysis regarding the orange module. (e) Protein interaction network of putative gene markers associated with the immune characteristic index.

The association of the immune-related features of 29 gene modules with the immune characteristic index was examined (Figure 7(a)). The immune feature index was shown to have strong correlations with 18 different modules. Subsequently, modules were chosen depending on their substantial association with the immune characteristic index for prognosis, and eight modules, including black, orange, violet, and white modules, were strongly linked to the prognosis of pancreatic cancer (*p* < 0.05) (Figure 7(b)). The orange module was identified as a candidate module based on its association with the molecular subtype and prognosis. Subsequently, the functions of genes within the orange module were assessed via enrichment analysis (Figures 7(c) and 7(d)). There existed a link between these genes and immunologic processes, including IFN-*γ*-mediated signalling pathway, cellular response to IFN-*γ*, type-I IFN signalling pathway, and response to IFN-*γ*. Furthermore, the hub genes of the module were determined to be those genes that had a correlation coefficient greater than 0.8 and were remarkably linked to prognosis (*p* < 0.05). Finally, 10 key genes in the orange module were identified, including *CMPK2*, *EPSTI1*, *IFI44*, *IFIH1*, *IFIT3*, *OAS1*, *OAS2*, *OAS3*, *PARP14*, and *UBE2L6*. These genes may be potential markers related to the immune characteristic index. A protein interaction network constructed based on these 10 hub genes (Figure 7(e)) revealed close interaction among them.

### 3.8. Prognostic Analysis and Clinical Validation of 10 Hub Genes

**Figure 8:**
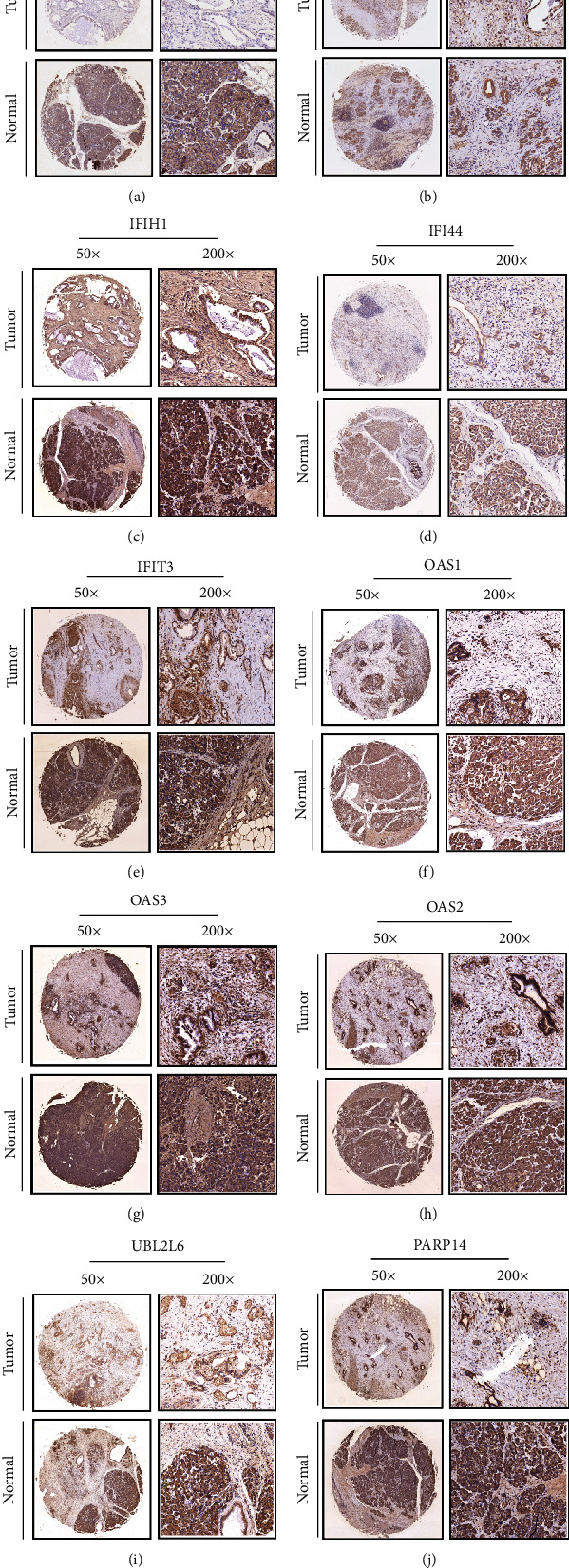
Prognostic analysis and clinical validation of 10 hub genes. (a–j) Expression of CMPK2, EPST1, IFIH1, IFI44, IFIT3, OSAS1, OAS3, OAS2, UBL2L6, and PARP14 in cancer and control tissues.

TMAs comprising 37 tissue samples from PAAD patients and 23 samples from healthy controls were obtained to validate protein expression. The findings illustrated that the expression level of CMPK2, EPST1, IFIH1, IFI44, IFIT3, OSAS1, OAS3, OAS2, UBL2L6, and PARP14 was considerably lowered in cancer tissues in contrasted with the normal tissues (Figures 8(a)–8(j), Supplementary Figure 1A-J). The KM curve of these 10 genes is presented in Supplementary Figure 1K–T. Seven out of the ten hub genes exhibited substantial associations with the prognosis of patients with pancreatic cancer, illustrating that they are possible indicators linked to the immune characteristic index.

## 4. Discussion

Pancreatic cancer is among the most lethal malignancies that pose the greatest risk of death worldwide, and the associated changes in the fibrogenic matrix and cytogenetic or epigenetic landscape create biological and physical barriers to successful treatment [35]. Previous studies have suggested that mismatched repair protein-deficient pancreatic cancer is characterised by microsatellite instability. Immune checkpoint inhibitor therapy is effective, with many patients with pancreatic cancer achieving satisfactory results with immunotherapy [36]. Therefore, to enhance the effectiveness of immune checkpoint inhibitors in pancreatic cancer therapy, it is necessary to identify reliable biological markers. In this research, pancreatic cancer was categorized into 3 subtypes: IS1, IS2, and IS3, to improve the understanding of its immunobiological components, and remarkable variations in prognosis were discovered across these subtypes. The correlation of the molecular subtypes with tumour immune cell infiltration, chemical drugs, and immunotherapy response was further analysed. The findings illustrated that the 3 subtypes exhibited distinct immune-related characteristics, immune cell infiltration levels, and immunotherapeutic effects. In addition, an immune characteristic index was constructed, which was found to be associated with immune checkpoint-related genes. Finally, 10 putative gene markers linked to the immune characteristic index were detected via coexpression network analysis. Therefore, we constructed and validated novel stratified immunoprognostic markers for pancreatic cancer and provided new predictive indices for immunotherapy of pancreatic cancer.

The characteristics of T-cell dysfunction and rejection in tumour-infiltrating lymphocytes (TILs) are considered accurate predictors of the responsiveness to immune checkpoint inhibitors. These characteristics can more accurately predict melanoma patients' prognoses after treatment with first-line anti-CTLA4 or anti-PD1 therapy as opposed to other biological markers such as tumour mutation burden and PD-L1 levels [37]. In this study, TIDE scores were substantially elevated in IS1 and IS2 in contrast with IS3, suggesting that IS3 benefited more from immunotherapy. In addition, IS1 had the highest predicted T-cell dysfunction score; however, no remarkable variations were observed in T-cell dysfunction scores between IS2 and IS3. Based on these findings, it was validated that although IS1 had the highest immune scores, its poor prognosis may be attributed to T-cell dysfunction. Furthermore, IS2 was found to have the highest T-cell rejection score, whereas IS3 exhibited the lowest score, which may be attributed to the dismal prognosis of IS2 and the good prognosis of IS3. Since the mid-1990s, gemcitabine has been administered as a mainstay chemotherapy drug to treat advanced pancreatic cancer, and it is critical for patients with unresectable pancreatic cancer. However, the overall survival response is poor, and pancreatic cancer cells in most patients are resistant to gemcitabine [38]. On the other hand, combination therapy with gemcitabine and the anti-EGFR tyrosine kinase inhibitor erlotinib confers a survival advantage over gemcitabine alone. Additionally, gemcitabine has also shown promising results when combined with platinum or capecitabine [39, 40]. In this study, the response of the three subtypes to gemcitabine, cisplatin, erlotinib, and 5-FU was analysed, and IS1 and IS2 were found to be more sensitive to gemcitabine, cisplatin, and erlotinib, whereas IS3 was found to be most sensitive to 5-FU. These results may serve as a reference for making clinical decisions regarding the administration of appropriate drugs because different groups of patients may exhibit different chemotherapeutic responses.

Different immune subtypes have different responses to immunotherapy, which may lead to different clinical advances. IFN-*γ* is an immune system cytokine that is primarily generated by natural killer cells and activated T cells. It is crucial for regulating immunological responses and anticancer immunity [41]. High lytic activity is associated with increased tumour survival rates, possibly owing to the increased immune and cytolytic activity of M1 macrophages and T cells. Studies on gastric and colorectal cancers have reported the antitumour immune function of the CYT score, which is a prognostic indicator of the effectiveness of immune checkpoint blockade therapy [42, 43]. Angiogenic factors drive immunosuppression by directly inhibiting immune effector cells and antigen-presenting cells or by enhancing the effects of tumour-associated macrophages (TAMs), myeloid inhibitory cells (MDSCs), and regulatory T cells (Tregs). A vicious loop that impairs antitumor immunity may be formed when immunosuppressive cells induce angiogenesis [44]. In this research, the immune cell scores in the 3 subtypes were calculated, and substantial variations were observed in immune characteristics across these subtypes. The proportion of CD4+ memory T cells, CD4+ resting T cells, M0 macrophages, and M1 macrophages was considerably elevated in the three subtypes, illustrating that these cells may have a fundamental function in immunotherapy of pancreatic cancer. Furthermore, the expression level of chemokines and their receptors was elevated in IS1 and IS2 compared to in IS3. The three subtypes had different immune characteristics, and the expression level of a majority of chemokines and checkpoint-associated genes was elevated in IS1 and IS2 in contrast with IS3. In addition, the IS1 subgroup exhibited the highest IFN-*γ* score, angiogenesis score, and immune T-cell lytic activity. These results suggest that immune cell infiltration might vary across various immune subtypes, which might result in differences in the advancement of the tumours as well as the effectiveness of immunotherapy. Nevertheless, the fundamental processes are yet to be elucidated, and the findings of this study need to be validated in additional research. The expression level of most genes linked to immune checkpoints was substantially elevated in IS1 and IS2 as opposed to IS3, suggesting that the activity of cytotoxic T lymphocytes (CTLs) in IS1 may be suppressed by immune checkpoint molecules. This finding may be attributed to the worst prognosis observed in IS1 despite the greatest infiltration levels of immune cells in the TME.

The TGF-*β* signalling pathway exerts a dual modulatory function in the onset and progression of tumor [45]. Apoptosis may be triggered in epithelial cells by TGF-*β*, which also can suppress their growth and proliferation. For this reason, the loss of responsiveness of epithelial cells to TGF-*β*-mediated suppression of proliferation is a key event in the process of carcinogenesis. With tumour progression, TGF-*β* enhances the invasion, metastasis, and drug resistance of tumour cells and maintains cancer cell stemness [46]. In addition, TGF-*β* inhibits or modulates the immune response, recruits macrophages and neutrophils to tumours, and induces their differentiation into the “type-2” phenotype. It also inhibits the normal antitumour function of neutrophils, macrophages, and type-1-differentiated T cells and promotes the secretion of protumour cytokines (including IL-11 and increased release of TGF-*β*) from type-2 immune cells [47–49]. Therefore, TGF-*β* signalling in the TME inhibits the antitumour “cytotoxic” effects of completely differentiated cells in the immune system. In this research, the enrichment scores of the TGF-*β* pathway were significantly elevated in IS1 and IS2 with elevated immune cell infiltration levels as opposed to IS3 with lower immune cell infiltration, suggesting that molecular typing is an efficient way to predict the effects of immunotherapy. Therefore, blocking the TGF-*β* signalling pathway has a powerful therapeutic potential in altering the balance of immune responses and enhancing the mechanisms of antitumour immunity.

Considering the different molecular characteristics of the three subtypes, a subtype classification index was established in this study using LDA. This index may be applied to evaluate the patients' immune features. A high characteristic index predicts lower immunosuppression (IS3), and a low characteristic index predicts higher immunosuppression (IS1).

Analysis of the coexpression network revealed the presence of a core gene module that was determined to be dependent on the immune characteristic index. A total of 29 clinically relevant modules were screened, of which, the orange module was associated with both molecular subtypes and prognosis. This module included 10 core genes, namely, *CMPK2*, *EPSTI1*, *IFI44*, *IFIH1*, *IFIT3*, *OAS1*, *OAS2*, *OAS3*, *PARP14*, and *UBE2L6*, which were strongly linked to the immune characteristic index. Cytidine/uridine monophosphate kinase 2 (CMPK2) is a type of mitochondrial nucleoside monophosphate kinase, which can maintain UTP/CT in cells [50]. It participates in mitochondrial DNA synthesis in mammals and mediates immunomodulation and antiviral activity via both IFN-independent and IFN-dependent pathways. In addition, it is involved in IFN-induced suppression of human HIV infection [51, 52], assumes an instrumental function in promoting the arteriosclerotic effects of IFN*α*, and is a promising treatment target for SLE [53]. Epithelial–stromal interaction 1 (*EPSTI1*) is a gene that responds to interferons and is well known for its role in the metastasis of malignant tumours. When compared with healthy breast tissue, the level of EPSTI1 expression is considerably elevated in invasive breast cancer tissues. EPSTI1 expression is related to the onset and migration, stem cell-like characteristics, epithelial–mesenchymal transition (EMT) as well as the invasion and metastasis of breast cancer cells. It is highest in the basal subtype of breast cancer, which has a poor prognosis. EPST11 can regulate the exogenous apoptosis of breast cancer cells with positive and triple-negative oestrogen receptors. miR-654-5p blocks the growth of breast cancer cells by specifically targeting EPSTI1, demonstrating its potential as a treatment target [54–58]. EPSTI1, an insulin-like growth factor induced by IL-28a, performs a vital function in IL-28a-induced antiviral activity [59]. Recent studies have reported the involvement of EPSTI1 in immune response, immune function, immune amnesty, and autoimmune disorders [60]. IFN-induced protein 44 (IFI44) is linked to various viral infections. It has the potential to be utilised as a target for the regulation of the innate immune system following viral infection [61, 62] and may inhibit signals from extracellular signal-modulated kinase by binding to intracellular GTP, ultimately resulting in an arrest of the cell cycle. LINC01116 is a critical component in the advancement of gefitinib-resistant non-small-cell lung carcinoma (NSCLC) by influencing the expression of IFI44, thus offering a novel treatment target for overcoming TKI resistance in NSCLC [63]. IFN-induced helicase C domain 1 (IFIH1), commonly referred to as melanoma differentiation-associated protein 5 (MDA5), is an intracellular protein that can recognise viral RNA and mediate natural immune responses. In a study on the molecular regulation of M1 macrophages in acute respiratory distress syndrome, IFIH1 was identified as a novel regulator of M1 macrophage polarisation and a potential therapeutic target [64]. The 2′,5′-oligoadenylate synthetase (OAS) family, including OAS1, OAS2, and OAS3, is composed of IFN-induced antiviral enzymes and has been well studied in the field of tuberculosis [65, 66]. *OAS1*, *OAS2*, *OAS3*, and *OASL* have been recognised as pivotal genes in a bioinformatic study focusing on trastuzumab-resistant gastric cancer [67]. Additionally, the expression of IFN-stimulated genes, as well as chemokines produced by human macrophages, is negatively regulated by *OAS1* and *OAS3*. Therefore, OAS proteins may modulate the innate immunological signals produced by macrophages, which has multiple implications for the treatment of viral diseases [68]. Poly(adenosine diphosphate-ribose) polymerase (PARP) is an intracellular ADP ribotransferase that protects lymphocytes from apoptosis. PARP14 accelerates lymphogenesis driven by continuous overexpression of the *c-Myc* oncogene and performs an indispensable function in the glycolysis flux induced by IL-4, suggesting a possible association of PARP14 with metabolic modulation [69, 70]. PARP14 has been reported as a promising drug target among the other 18 members of the PARP family that are known. Recent research has identified the molecular processes of PARP14 as a new prospective therapeutic target for a variety of malignancies, such as prostate cancer, multiple myeloma, ovarian cancer, diffuse large B-cell lymphoma, and breast cancer. PARP14 is involved in the cellular response and signalling pathways of the immune system, in which it regulates the activation of macrophages, and is considered a viable target for treating tumour-related and allergic inflammation [71, 72]. It may promote the proliferation, antiapoptotic activity, and gemcitabine resistance of pancreatic cancer cells through the NF-*Κ*B signalling pathway, thereby illustrating its promise as a target for pancreatic cancer therapy [73]. The elevated expression level of PARP14 is linked to the unfavourable prognosis of primary hepatocellular carcinoma. In addition, targeting PARP14 enhances the sensitivity of hepatoma cells to antihepatoma drugs [74]. Ubiquitin/ISG15-conjugating enzyme E2L6 (UBE2L6) is an E2 ubiquitin/ISG15-binding enzyme that has a decisive function in inhibiting cell proliferation and xenograft tumour advancement by interacting with E3 ubiquitin ligase to target c-Myc for proteasomal disintegration [75]. It is a new autophagy inhibitor, which may impact the chemosensitivity of oesophageal cancer cells [76]. In addition, it assumes a fundamental function in inhibiting the differentiation of leukaemia cells [77] and is a new molecular target to overcome cisplatin resistance [78]. The abovementioned genes have been extensively investigated with regard to tumour progression and prognosis and might act as useful biological markers for prognostic prediction and estimating the efficacy of immunotherapy in pancreatic cancer patients. Because these immune marker-based genes are closely related to tumour development and immune invasion, they warrant further investigation. Moreover, additional comprehensive and comparative studies should be conducted to validate the efficacy of the classification in clinical evaluation and decision-making.

In the present research, pancreatic cancer was divided into distinct immune molecular subtypes, and the association of these subtypes with immune checkpoints was examined. In addition, an immune characterisation index was established to assess the immune-related characteristics of patients. This study provides potential molecular targets for developing new immunotherapeutic approaches for pancreatic cancer, which may eventually help to develop individualised patient therapy. Although the link between the index and immune cells was validated, this research has some drawbacks. First, the findings are founded on a single-centre clinical trial and require further validation in multicentre clinical trials and larger prospective studies. Second, all patients in this study were selected retrospectively, which might have contributed to selection bias owing to the small sample size. Additionally, only microarray expression datasets were included in this study. Hence, the molecular functions of the identified marker genes should be investigated in functional experiments, and future studies should focus on immunotyping and clinical validation of the characterisation index, which may provide more evidence for the use of the index in clinical practice.

As far as we know, this study is the first to stratify patients with pancreatic cancer based on immune characteristics and to construct an immune subtype characteristic index linked to the expression of genes linked to immune cells. The findings of this study offer novel insights into predicting the efficacy and possible therapeutic targets of immunotherapy. Collectively, this research highlights the possible molecular targets for establishing novel immunotherapeutic strategies for pancreatic cancer, which might eventually aid in the establishment of individualised therapies for patients based on their immune characteristics.

## Figures and Tables

**Table 1:** Statistical analysis of the dataset used in this study.

		TCGA-PAAD	ICGC-PAAD	GSE71729
Survival				

OS	Status_0	85	93	41
	Status_1	92	144	84

DSS	Status_0	99	—	—
	Status_1	72	—	—
	NA	6	—	—

DFI	Status_0	46	—	—
	Status_1	23	—	—
	NA	108	—	—

PFI	Status_0	74	—	—
	Status_1	103	—	—

T stage	T1	7	—	—
	T2	24	—	—
	T3	141	—	—
	T4	3	—	—
	NA	2	—	—

N stage	N0	49	—	—
	N1	123	—	—
	NA	5	—	—

M stage	M0	79	—	—
	M1	4	—	—
	NA	94	—	—

Stage	Stage I	21	10	—
	Stage II	146	216	—
	Stage III	3	1	—
	Stage IV	4	6	—
	NA	3	4	—

Grade	G1	31	17	—
	G2	94	143	—
	G3	48	72	—
	G4	2	3	—
	NA	2	2	—

Gender	Female	80	130	—
	Male	97	107	—

## Data Availability

Datasets involved in this research are accessible to the general public.

## Supplementary Materials

Supplementary Materials Supplementary Figure 1: prognostic analysis and clinical validation of 10 hub genes. Supplement Table 1: the enrichment scores of 184 immune-associated features in GEO, ICGC, and TCGA cohorts.
