# Supplementary material for: Systematic Analysis of Molecular Subtypes Based on the Expression Profile of Immune-Related Genes in Pancreatic Cancer
Source: Oxid Med Cell Longev. 2022 Dec 15;2022:3124122. doi: 10.1155/2022/3124122 (PMC9780013; doi:10.1155/2022/3124122)
Supplement: Supplementary Materials — Supplementary Figure 1: prognostic analysis and clinical validation of 10 hub genes. Supplement Table 1: the enrichment scores of 184 immune-associated features in GEO, ICGC, and TCGA cohorts. [file 3124122.f1.docx]

**Supplementary Figure 1.**  Prognostic analysis and clinical validation of 10 hub genes

**Supplement Table1.** The enrichment scores of 184 immune-associated features in GEO, ICGC, and TCGA cohorts

SetName Gene

Angiogenesis CDH5

Angiogenesis CLEC14A

Angiogenesis LDB2

Angiogenesis ECSCR

Angiogenesis MYCT1

Angiogenesis RHOJ

Angiogenesis VWF

Angiogenesis TIE1

Angiogenesis KDR

Angiogenesis ESAM

Angiogenesis PTPRB

Angiogenesis SPARCL1

Angiogenesis EMCN

Angiogenesis ROBO4

Angiogenesis ENG

Angiogenesis TEK

Angiogenesis S1PR1

Angiogenesis A2M

Angiogenesis JAM2

Angiogenesis COL15A1

Angiogenesis PECAM1

Angiogenesis CALCRL

Angiogenesis CLEC3B

Angiogenesis PLVAP

Angiogenesis RGS5

Angiogenesis LRRC32

Angiogenesis EBF1

Angiogenesis ADCY4

Angiogenesis ACVRL1

Angiogenesis APLNR

Angiogenesis TM4SF18

Angiogenesis GNG11

Angiogenesis CNRIP1

Angiogenesis ZNF423

Angiogenesis GIMAP8

Angiogenesis PDGFD

Angiogenesis ITGA9

Angiogenesis EDNRB

Angiogenesis ADGRL4

Angiogenesis ADGRF5

Angiogenesis ADGRA2

APM1 HLA-A

APM1 HLA-B

APM1 HLA-C

APM1 TAP1

APM1 TAP2

APM1 TAPBP

APM1 B2M

APM2 HLA-DMA

APM2 HLA-DMB

APM2 HLA-DOA

APM2 HLA-DOB

APM2 HLA-DPA1

APM2 HLA-DPB1

APM2 HLA-DPB2

APM2 HLA-DQA1

APM2 HLA-DQA2

APM2 HLA-DQB1

APM2 HLA-DQB2

APM2 HLA-DRA

APM2 HLA-DRB1

APM2 HLA-DRB5

APM2 HLA-DRB6

B_cell_PCA_16704732 SLC22A3

B_cell_PCA_16704732 IGKC

B_cell_PCA_16704732 FGD2

B_cell_PCA_16704732 SIDT2

B_cell_PCA_16704732 CACNA2D2

B_cell_PCA_16704732 USP6NL

B_cell_PCA_16704732 CPSF2

B_cell_PCA_16704732 UBR5

B_cell_PCA_16704732 NIPAL4

B_cell_PCA_16704732 WEE1

B_cell_PCA_16704732 PMAIP1

B_cell_PCA_16704732 SLC2A5

B_cell_PCA_16704732 BEND4

B_cell_PCA_16704732 SP140

B_cell_PCA_16704732 GLDC

B_cell_PCA_16704732 GSTZ1

B_cell_PCA_16704732 EML6

B_cell_PCA_16704732 FCRLA

B_cell_PCA_16704732 LRMP

B_cell_PCA_16704732 BTK

B_cell_PCA_16704732 HLA-DMB

B_cell_PCA_16704732 SAV1

B_cell_PCA_16704732 PLEKHF2

B_cell_PCA_16704732 STAG3

B_cell_PCA_16704732 HHEX

B_cell_PCA_16704732 LINC00926

B_cell_PCA_16704732 WDR11

B_cell_PCA_16704732 SSU72

B_cell_PCA_16704732 CD19

B_cell_PCA_16704732 SYNGR2

B_cell_PCA_16704732 ZNF532

B_cell_PCA_16704732 TRIM56

B_cell_PCA_16704732 MEF2C

B_cell_PCA_16704732 INPPL1

B_cell_PCA_16704732 NFKBIE

B_cell_PCA_16704732 JUP

B_cell_PCA_16704732 EGR1

B_cell_PCA_16704732 FCRL2

B_cell_PCA_16704732 TBC1D1

B_cell_PCA_16704732 ANKH

B_cell_PCA_16704732 TRIO

B_cell_PCA_16704732 RGS13

B_cell_PCA_16704732 SSPN

B_cell_PCA_16704732 ATG4A

B_cell_PCA_16704732 C11orf24

B_cell_PCA_16704732 PRKCE

B_cell_PCA_16704732 RALGPS2

B_cell_PCA_16704732 AIM2

B_cell_PCA_16704732 PIK3AP1

B_cell_PCA_16704732 BACE2

B_cell_PCA_16704732 COPS3

B_cell_PCA_16704732 CD22

B_cell_PCA_16704732 FCRL5

B_cell_PCA_16704732 POLD4

B_cell_PCA_16704732 TSPAN33

B_cell_PCA_16704732 EPHX1

B_cell_PCA_16704732 PEA15

B_cell_PCA_16704732 CNR2

B_cell_PCA_16704732 IFNGR2

B_cell_PCA_16704732 TSPAN31

B_cell_PCA_16704732 PCCA

B_cell_PCA_16704732 ANXA4

B_cell_PCA_16704732 RAP1GAP2

B_cell_PCA_16704732 MYBL2

B_cell_PCA_16704732 CD72

B_cell_PCA_16704732 CIITA

B_cell_PCA_16704732 PMEPA1

B_cell_PCA_16704732 CR1

B_cell_PCA_16704732 ITPR1

B_cell_PCA_16704732 IFI27

B_cell_PCA_16704732 HLA-DOA

B_cell_PCA_16704732 CDKN2A

B_cell_PCA_16704732 ZNF207

B_cell_PCA_16704732 SYT17

B_cell_PCA_16704732 EVI5L

B_cell_PCA_16704732 TUBB6

B_cell_PCA_16704732 DDR1

B_cell_PCA_16704732 COL14A1

B_cell_PCA_16704732 CHD7

B_cell_PCA_16704732 APOBEC3B

B_cell_PCA_16704732 FAM129C

B_cell_PCA_16704732 SCRN1

B_cell_PCA_16704732 TAP2

B_cell_PCA_16704732 MARCKS

B_cell_PCA_16704732 SNX10

B_cell_PCA_16704732 CCNG2

B_cell_PCA_16704732 CYBB

B_cell_PCA_16704732 BRD4

B_cell_PCA_16704732 PLEKHO1

B_cell_PCA_16704732 RNASE6

B_cell_PCA_16704732 STAP1

B_cell_PCA_16704732 ATP6V0A1

B_cell_PCA_16704732 VPS53

B_cell_PCA_16704732 RFX3

B_cell_PCA_16704732 MOB3B

B_cell_PCA_16704732 KDM4B

B_cell_PCA_16704732 CORO1C

B_cell_PCA_16704732 TCL1A

B_cell_PCA_16704732 RAB30

B_cell_PCA_16704732 SEMA4B

B_cell_PCA_16704732 DENND5B

B_cell_PCA_16704732 ARHGEF3

B_cell_PCA_16704732 UVRAG

B_cell_PCA_16704732 TSPAN3

B_cell_PCA_16704732 PARP14

B_cell_PCA_16704732 CTSZ

B_cell_PCA_16704732 HLA-DPB1

B_cell_PCA_16704732 F5

B_cell_PCA_16704732 ZYG11B

B_cell_PCA_16704732 GSAP

B_cell_PCA_16704732 BLK

B_cell_PCA_16704732 CLIC4

B_cell_PCA_16704732 HUWE1

B_cell_PCA_16704732 NAA50

B_cell_PCA_16704732 DAPP1

B_cell_PCA_16704732 FIG4

B_cell_PCA_16704732 SRGAP2

B_cell_PCA_16704732 FCGR2B

B_cell_PCA_16704732 BCL7A

B_cell_PCA_16704732 GUSBP11

B_cell_PCA_16704732 OSBPL10

B_cell_PCA_16704732 CD200

B_cell_PCA_16704732 RHOH

B_cell_PCA_16704732 SH3BP5

B_cell_PCA_16704732 CEBPB

B_cell_PCA_16704732 GNA12

B_cell_PCA_16704732 PALM2-AKAP2

B_cell_PCA_16704732 NKG7

B_cell_PCA_16704732 IRF8

B_cell_PCA_16704732 TNFRSF17

B_cell_PCA_16704732 CYB561A3

B_cell_PCA_16704732 ADK

B_cell_PCA_16704732 CORO2B

B_cell_PCA_16704732 CD86

B_cell_PCA_16704732 WDR83OS

B_cell_PCA_16704732 STX7

B_cell_PCA_16704732 CD1D

B_cell_PCA_16704732 HLA-DPA1

B_cell_PCA_16704732 KLHL14

B_cell_PCA_16704732 RALGAPB

B_cell_PCA_16704732 HLA-DQB1

B_cell_PCA_16704732 HCG18

B_cell_PCA_16704732 RCSD1

B_cell_PCA_16704732 PSEN2

B_cell_PCA_16704732 MZB1

B_cell_PCA_16704732 HRK

B_cell_PCA_16704732 TNFRSF18

B_cell_PCA_16704732 IL4R

B_cell_PCA_16704732 MTPN

B_cell_PCA_16704732 DRAM2

B_cell_PCA_16704732 TEAD2

B_cell_PCA_16704732 MTSS1

B_cell_PCA_16704732 SMAGP

B_cell_PCA_16704732 NCF4

B_cell_PCA_16704732 RBMS3

B_cell_PCA_16704732 SKAP2

B_cell_PCA_16704732 HSPA5

B_cell_PCA_16704732 HIST1H2BK

B_cell_PCA_16704732 CR2

B_cell_PCA_16704732 ODC1

B_cell_PCA_16704732 LHFPL2

B_cell_PCA_16704732 TCTN1

B_cell_PCA_16704732 DMXL1

B_cell_PCA_16704732 KYNU

B_cell_PCA_16704732 PIK3C2B

B_cell_PCA_16704732 FUBP1

B_cell_PCA_16704732 HLA-DRA

B_cell_PCA_16704732 CCDC25

B_cell_PCA_16704732 BMF

B_cell_PCA_16704732 SETBP1

B_cell_PCA_16704732 SAMD9

B_cell_PCA_16704732 MGME1

B_cell_PCA_16704732 FCRL1

B_cell_PCA_16704732 PUS10

B_cell_PCA_16704732 CD180

B_cell_PCA_16704732 RNF141

B_cell_PCA_16704732 CD79A

B_cell_PCA_16704732 LY86

B_cell_PCA_16704732 TNS3

B_cell_PCA_16704732 NUP88

B_cell_PCA_16704732 TLR7

B_cell_PCA_16704732 HVCN1

B_cell_PCA_16704732 ZNF154

B_cell_PCA_16704732 BTLA

B_cell_PCA_16704732 RFX5

B_cell_PCA_16704732 ZCCHC7

B_cell_PCA_16704732 SNX29P2

B_cell_PCA_16704732 CYTH1

B_cell_PCA_16704732 BTNL9

B_cell_PCA_16704732 CD79B

B_cell_PCA_16704732 CD38

B_cell_PCA_16704732 SYVN1

B_cell_PCA_16704732 IFIT3

B_cell_PCA_16704732 BLNK

B_cell_PCA_16704732 HERPUD1

B_cell_PCA_16704732 DTX1

B_cell_PCA_16704732 RHOBTB2

B_cell_PCA_16704732 CXCR5

B_cell_PCA_16704732 KLF1

B_cell_PCA_16704732 VPREB3

B_cell_PCA_16704732 CHERP

B_cell_PCA_16704732 SLC2A1

B_cell_PCA_16704732 E2F5

B_cell_PCA_16704732 HECW2

B_cell_PCA_16704732 WDR34

B_cell_PCA_16704732 TLR10

B_cell_PCA_16704732 LYN

B_cell_PCA_16704732 IGHG1

B_cell_PCA_16704732 CCDC50

B_cell_PCA_16704732 KAZN

B_cell_PCA_16704732 PLCG2

B_cell_PCA_16704732 MYO1E

B_cell_PCA_16704732 CORO1A

B_cell_PCA_16704732 POU2F2

B_cell_PCA_16704732 AFF3

B_cell_PCA_16704732 GM2A

B_cell_PCA_16704732 POU2AF1

B_cell_PCA_16704732 FNBP4

B_cell_PCA_16704732 UROS

B_cell_PCA_16704732 BIRC3

B_cell_PCA_16704732 WIPF1

B_cell_PCA_16704732 TPD52

B_cell_PCA_16704732 IGL

B_cell_PCA_16704732 MS4A1

B_cell_PCA_16704732 BCL11A

B_cell_PCA_16704732 SMAD3

B_cell_PCA_16704732 ADAM28

B_cell_PCA_16704732 DNAJC10

B_cell_PCA_16704732 STAT6

B_cell_PCA_16704732 HLA-DQB2

B_cell_PCA_16704732 IGLL1

B_cell_PCA_16704732 ABCA1

B_cell_PCA_16704732 MAP3K8

B_cell_PCA_16704732 BSG

B_cell_PCA_16704732 LETMD1

B_cell_PCA_16704732 SMC6

B_cell_PCA_16704732 SPIB

B_cell_PCA_16704732 NR1H2

B_cell_PCA_16704732 SEL1L3

B_cell_PCA_16704732 FOXP1

B_cell_PCA_16704732 TOP2A

B_cell_PCA_16704732 HLA-DRB1

B_cell_PCA_16704732 IRF4

B_cell_PCA_16704732 LGALS9

B_cell_PCA_16704732 UBE2J1

B_cell_PCA_16704732 ACTA2

B_cell_PCA_16704732 SHMT2

B_cell_PCA_16704732 RRAS2

B_cell_PCA_16704732 SYK

B_cell_PCA_16704732 GCNT1

B_cell_PCA_16704732 SYPL1

B_cell_PCA_16704732 SUN2

B_cell_PCA_16704732 MMP11

B_cell_PCA_16704732 SNX2

B_cell_PCA_16704732 HLA-DQA1

B_cell_PCA_16704732 AMFR

B_cell_PCA_16704732 PRICKLE1

B_cell_PCA_16704732 TTC7A

B_cell_PCA_16704732 SLC15A2

B_cell_PCA_16704732 CD74

B_cell_PCA_16704732 TFEB

B_cell_PCA_16704732 CD83

B_cell_PCA_16704732 ARHGAP24

B_cell_PCA_16704732 FBXO41

B_cell_PCA_16704732 SWAP70

B_cell_PCA_16704732 BANK1

B_cell_PCA_16704732 SERPINA1

B_cell_PCA_16704732 L3MBTL4

B_cell_PCA_16704732 PRCP

B_cell_PCA_16704732 DNAH11

B_cell_PCA_16704732 GRAMD1C

B_cell_PCA_16704732 GUCD1

B_cell_PCA_16704732 TLR1

B_cell_PCA_16704732 43160

B_cell_PCA_16704732 GRK3

B_cell_PCA_16704732 LARGE2

B_cell_PCA_16704732 JCHAIN

B_cell_PCA_16704732 RUBCNL

B_cell_PCA_16704732 JADE3

B_cell_PCA_16704732 PLPP5

Bcell_21978456 IGHA1

Bcell_21978456 IGHG1

Bcell_21978456 IGHM

Bcell_21978456 IGH

Bcell_21978456 IGHA2

Bcell_21978456 IGHD

Bcell_21978456 IGHG3

Bcell_21978456 IGHG4

Bcell_21978456 IGHV4-31

Bcell_21978456 IGHV1-69

Bcell_21978456 IGLJ3

Bcell_21978456 IGHV3-23

Bcell_21978456 IGK

Bcell_21978456 IGHG2

Bcell_21978456 IGKC

Bcell_21978456 GUSBP11

Bcell_21978456 IGLC1

Bcell_21978456 IGLV1-44

Bcell_21978456 CYAT1

Bcell_21978456 IGLV@

Bcell_21978456 IGKV1D-13

Bcell_21978456 IGLL3P

Bcell_21978456 IGLL5

Bcell_21978456 SCFV

Bcell_mg_IGJ IGHM

Bcell_mg_IGJ IGHG3

Bcell_mg_IGJ IGHV1-69

Bcell_mg_IGJ IGKC

Bcell_mg_IGJ IGLJ3

Bcell_mg_IGJ IGKV1D-13

Bcell_mg_IGJ IFI6

Bcell_mg_IGJ IGHA1

Bcell_mg_IGJ IGKV1OR2-108

Bcell_mg_IGJ IGKV1OR15-118

Bcell_mg_IGJ CTA-246H3.1

Bcell_mg_IGJ JCHAIN

Bcell_mg_IGJ IGL

Bcell_mg_IGJ CXCL8

Bcell_mg_IGJ LOC652128

Bcell_mg_IGJ NTN3

Bcell_receptors_score CD19

Bcell_receptors_score CD1C

Bcell_receptors_score CD1D

Bcell_receptors_score CD200

Bcell_receptors_score CD22

Bcell_receptors_score CD24

Bcell_receptors_score CD38

Bcell_receptors_score CD72

Bcell_receptors_score CD74

Bcell_receptors_score CD79A

Bcell_receptors_score CD79B

Bcell_receptors_score CD83

Bcell_receptors_score CD86

Bcell_receptors_score FCGR2B

Bcell_receptors_score MS4A1

Buck14_score CXCL13

Buck14_score CLIC5

Buck14_score RGS4

Buck14_score RPS28

Buck14_score RFX7

Buck14_score EXOC7

Buck14_score HAPLN1

Buck14_score ZNF3

Buck14_score SSX3

Buck14_score PRRG3

Buck14_score ABO

Buck14_score PRTN3

Buck14_score MATN1

Buck14_score AGFG2

CD103neg_mean_25446897 ABCC3

CD103neg_mean_25446897 APOE

CD103neg_mean_25446897 C1QA

CD103neg_mean_25446897 C1QB

CD103neg_mean_25446897 C1QC

CD103neg_mean_25446897 C5AR1

CD103neg_mean_25446897 LYVE1

CD103neg_mean_25446897 MERTK

CD103neg_mean_25446897 MRC1

CD103neg_mean_25446897 MS4A7

CD103neg_mean_25446897 SIGLEC1

CD103neg_mean_25446897 STAB1

CD103neg_mean_25446897 TLR7

CD103neg_mean_25446897 TMEM119

CD103neg_mean_25446897 TMEM37

CD103neg_mean_25446897 TREM2

CD103neg_mean_25446897 CYP4F18/CYP4F3A

CD103pos_CD103neg_ratio_25446897 CD103neg_mean_25446897

CD103pos_CD103neg_ratio_25446897 CD103pos_mean_25446897

CD103pos_mean_25446897 BATF3

CD103pos_mean_25446897 BTLA

CD103pos_mean_25446897 CCR7

CD103pos_mean_25446897 CLEC9A

CD103pos_mean_25446897 FLT3

CD103pos_mean_25446897 IRF8

CD103pos_mean_25446897 KIT

CD103pos_mean_25446897 XCR1

CD103pos_mean_25446897 ZBTB46

CD103pos_mean_25446897 MYCL

CD68 CD68

CD8_CD68_ratio CD8A

CD8_CD68_ratio CD68

CD8_PCA_16704732 DUSP2

CD8_PCA_16704732 CST7

CD8_PCA_16704732 FCGBP

CD8_PCA_16704732 S100B

CD8_PCA_16704732 ADRB2

CD8_PCA_16704732 KLRC4

CD8_PCA_16704732 CCL5

CD8_PCA_16704732 IL2RB

CD8_PCA_16704732 GNLY

CD8_PCA_16704732 KLRC1

CD8_PCA_16704732 CD8A

CD8_PCA_16704732 C1orf21

CD8_PCA_16704732 CCL4L1

CD8_PCA_16704732 TBX21

CD8_PCA_16704732 TSPAN32

CD8_PCA_16704732 KLRG1

CD8_PCA_16704732 PRF1

CD8_PCA_16704732 GZMH

CD8_PCA_16704732 ADGRG1

CD8A CD8A

CHANG_CORE_SERUM_RESPONSE_UP CEP78

CHANG_CORE_SERUM_RESPONSE_UP LSM3

CHANG_CORE_SERUM_RESPONSE_UP LRRC40

CHANG_CORE_SERUM_RESPONSE_UP STK17A

CHANG_CORE_SERUM_RESPONSE_UP RPN1

CHANG_CORE_SERUM_RESPONSE_UP JUNB

CHANG_CORE_SERUM_RESPONSE_UP NUP85

CHANG_CORE_SERUM_RESPONSE_UP FLNC

CHANG_CORE_SERUM_RESPONSE_UP HMGN2

CHANG_CORE_SERUM_RESPONSE_UP RPP40

CHANG_CORE_SERUM_RESPONSE_UP UQCR10

CHANG_CORE_SERUM_RESPONSE_UP AIMP2

CHANG_CORE_SERUM_RESPONSE_UP CHEK1

CHANG_CORE_SERUM_RESPONSE_UP VTA1

CHANG_CORE_SERUM_RESPONSE_UP EXOSC8

CHANG_CORE_SERUM_RESPONSE_UP CENPO

CHANG_CORE_SERUM_RESPONSE_UP PNO1

CHANG_CORE_SERUM_RESPONSE_UP SLC16A1

CHANG_CORE_SERUM_RESPONSE_UP WDR77

CHANG_CORE_SERUM_RESPONSE_UP UBE2J1

CHANG_CORE_SERUM_RESPONSE_UP NOP16

CHANG_CORE_SERUM_RESPONSE_UP NUDT1

CHANG_CORE_SERUM_RESPONSE_UP SMC2

CHANG_CORE_SERUM_RESPONSE_UP SLC25A5

CHANG_CORE_SERUM_RESPONSE_UP DLEU2

CHANG_CORE_SERUM_RESPONSE_UP PDAP1

CHANG_CORE_SERUM_RESPONSE_UP COX17

CHANG_CORE_SERUM_RESPONSE_UP BCCIP

CHANG_CORE_SERUM_RESPONSE_UP PLG

CHANG_CORE_SERUM_RESPONSE_UP RGS8

CHANG_CORE_SERUM_RESPONSE_UP SNRPC

CHANG_CORE_SERUM_RESPONSE_UP PLK4

CHANG_CORE_SERUM_RESPONSE_UP NUTF2

CHANG_CORE_SERUM_RESPONSE_UP LSM4

CHANG_CORE_SERUM_RESPONSE_UP SMS

CHANG_CORE_SERUM_RESPONSE_UP EBNA1BP2

CHANG_CORE_SERUM_RESPONSE_UP VDAC1

CHANG_CORE_SERUM_RESPONSE_UP PSMD14

CHANG_CORE_SERUM_RESPONSE_UP MYCBP

CHANG_CORE_SERUM_RESPONSE_UP SMURF2

CHANG_CORE_SERUM_RESPONSE_UP GNG11

CHANG_CORE_SERUM_RESPONSE_UP F3

CHANG_CORE_SERUM_RESPONSE_UP IL7R

CHANG_CORE_SERUM_RESPONSE_UP BRIP1

CHANG_CORE_SERUM_RESPONSE_UP HNRNPA2B1

CHANG_CORE_SERUM_RESPONSE_UP DCK

CHANG_CORE_SERUM_RESPONSE_UP ALKBH7

CHANG_CORE_SERUM_RESPONSE_UP MSN

CHANG_CORE_SERUM_RESPONSE_UP TPM1

CHANG_CORE_SERUM_RESPONSE_UP HYLS1

CHANG_CORE_SERUM_RESPONSE_UP HAUS1

CHANG_CORE_SERUM_RESPONSE_UP NUP93

CHANG_CORE_SERUM_RESPONSE_UP SNRPE

CHANG_CORE_SERUM_RESPONSE_UP ITGA6

CHANG_CORE_SERUM_RESPONSE_UP CENPN

CHANG_CORE_SERUM_RESPONSE_UP C11orf24

CHANG_CORE_SERUM_RESPONSE_UP GGH

CHANG_CORE_SERUM_RESPONSE_UP PFKP

CHANG_CORE_SERUM_RESPONSE_UP FARSA

CHANG_CORE_SERUM_RESPONSE_UP EIF2AK1

CHANG_CORE_SERUM_RESPONSE_UP CENPW

CHANG_CORE_SERUM_RESPONSE_UP TUBA4A

CHANG_CORE_SERUM_RESPONSE_UP TRA2B

CHANG_CORE_SERUM_RESPONSE_UP UMPS

CHANG_CORE_SERUM_RESPONSE_UP MRTO4

CHANG_CORE_SERUM_RESPONSE_UP NUDT15

CHANG_CORE_SERUM_RESPONSE_UP PGM2

CHANG_CORE_SERUM_RESPONSE_UP DBNDD1

CHANG_CORE_SERUM_RESPONSE_UP SNRPB

CHANG_CORE_SERUM_RESPONSE_UP MNAT1

CHANG_CORE_SERUM_RESPONSE_UP NUP35

CHANG_CORE_SERUM_RESPONSE_UP HSPB11

CHANG_CORE_SERUM_RESPONSE_UP C19orf48

CHANG_CORE_SERUM_RESPONSE_UP ID3

CHANG_CORE_SERUM_RESPONSE_UP IPO4

CHANG_CORE_SERUM_RESPONSE_UP FARSB

CHANG_CORE_SERUM_RESPONSE_UP EIF4G1

CHANG_CORE_SERUM_RESPONSE_UP SKA1

CHANG_CORE_SERUM_RESPONSE_UP MFSD11

CHANG_CORE_SERUM_RESPONSE_UP PLAUR

CHANG_CORE_SERUM_RESPONSE_UP MARVELD2

CHANG_CORE_SERUM_RESPONSE_UP MCM3

CHANG_CORE_SERUM_RESPONSE_UP DHFR

CHANG_CORE_SERUM_RESPONSE_UP RNF41

CHANG_CORE_SERUM_RESPONSE_UP ID2

CHANG_CORE_SERUM_RESPONSE_UP H2AFZ

CHANG_CORE_SERUM_RESPONSE_UP CDK2

CHANG_CORE_SERUM_RESPONSE_UP NCLN

CHANG_CORE_SERUM_RESPONSE_UP ZWILCH

CHANG_CORE_SERUM_RESPONSE_UP DYNLT1

CHANG_CORE_SERUM_RESPONSE_UP SLC25A40

CHANG_CORE_SERUM_RESPONSE_UP RHOC

CHANG_CORE_SERUM_RESPONSE_UP CCT5

CHANG_CORE_SERUM_RESPONSE_UP PDIA4

CHANG_CORE_SERUM_RESPONSE_UP SNRPA

CHANG_CORE_SERUM_RESPONSE_UP RBM14

CHANG_CORE_SERUM_RESPONSE_UP PDLIM7

CHANG_CORE_SERUM_RESPONSE_UP PITPNC1

CHANG_CORE_SERUM_RESPONSE_UP TPM3

CHANG_CORE_SERUM_RESPONSE_UP CORO1C

CHANG_CORE_SERUM_RESPONSE_UP ERLIN1

CHANG_CORE_SERUM_RESPONSE_UP PAICS

CHANG_CORE_SERUM_RESPONSE_UP TPRKB

CHANG_CORE_SERUM_RESPONSE_UP SKA2

CHANG_CORE_SERUM_RESPONSE_UP MYBL1

CHANG_CORE_SERUM_RESPONSE_UP SH3BP5L

CHANG_CORE_SERUM_RESPONSE_UP BRCA2

CHANG_CORE_SERUM_RESPONSE_UP SAR1A

CHANG_CORE_SERUM_RESPONSE_UP POLR3K

CHANG_CORE_SERUM_RESPONSE_UP MRPS28

CHANG_CORE_SERUM_RESPONSE_UP NUP107

CHANG_CORE_SERUM_RESPONSE_UP TUBG1

CHANG_CORE_SERUM_RESPONSE_UP PNN

CHANG_CORE_SERUM_RESPONSE_UP FAM167A

CHANG_CORE_SERUM_RESPONSE_UP RFC3

CHANG_CORE_SERUM_RESPONSE_UP MYL6

CHANG_CORE_SERUM_RESPONSE_UP MCM7

CHANG_CORE_SERUM_RESPONSE_UP MAGOHB

CHANG_CORE_SERUM_RESPONSE_UP FAM89B

CHANG_CORE_SERUM_RESPONSE_UP TOMM40

CHANG_CORE_SERUM_RESPONSE_UP CDCA4

CHANG_CORE_SERUM_RESPONSE_UP MT3

CHANG_CORE_SERUM_RESPONSE_UP MTHFD1

CHANG_CORE_SERUM_RESPONSE_UP PSMD12

CHANG_CORE_SERUM_RESPONSE_UP MYBL2

CHANG_CORE_SERUM_RESPONSE_UP CKLF

CHANG_CORE_SERUM_RESPONSE_UP NRIP3

CHANG_CORE_SERUM_RESPONSE_UP EZR

CHANG_CORE_SERUM_RESPONSE_UP GPLD1

CHANG_CORE_SERUM_RESPONSE_UP SRM

CHANG_CORE_SERUM_RESPONSE_UP RAB3B

CHANG_CORE_SERUM_RESPONSE_UP NLN

CHANG_CORE_SERUM_RESPONSE_UP MT1F

CHANG_CORE_SERUM_RESPONSE_UP TNFRSF12A

CHANG_CORE_SERUM_RESPONSE_UP TPI1

CHANG_CORE_SERUM_RESPONSE_UP HAS2

CHANG_CORE_SERUM_RESPONSE_UP APOO

CHANG_CORE_SERUM_RESPONSE_UP FBXO41

CHANG_CORE_SERUM_RESPONSE_UP MRPL37

CHANG_CORE_SERUM_RESPONSE_UP GSTCD

CHANG_CORE_SERUM_RESPONSE_UP SDC1

CHANG_CORE_SERUM_RESPONSE_UP WDR54

CHANG_CORE_SERUM_RESPONSE_UP RNF138

CHANG_CORE_SERUM_RESPONSE_UP RMND5B

CHANG_CORE_SERUM_RESPONSE_UP ENO1

CHANG_CORE_SERUM_RESPONSE_UP MAP3K8

CHANG_CORE_SERUM_RESPONSE_UP TMEM130

CHANG_CORE_SERUM_RESPONSE_UP SNX17

CHANG_CORE_SERUM_RESPONSE_UP KRR1

CHANG_CORE_SERUM_RESPONSE_UP TAGLN

CHANG_CORE_SERUM_RESPONSE_UP PA2G4

CHANG_CORE_SERUM_RESPONSE_UP RUVBL1

CHANG_CORE_SERUM_RESPONSE_UP SNRPD1

CHANG_CORE_SERUM_RESPONSE_UP LOXL2

CHANG_CORE_SERUM_RESPONSE_UP POLE2

CHANG_CORE_SERUM_RESPONSE_UP MAPRE1

CHANG_CORE_SERUM_RESPONSE_UP IMP4

CHANG_CORE_SERUM_RESPONSE_UP EMP2

CHANG_CORE_SERUM_RESPONSE_UP PSMD2

CHANG_CORE_SERUM_RESPONSE_UP MET

CHANG_CORE_SERUM_RESPONSE_UP IFRD2

CHANG_CORE_SERUM_RESPONSE_UP LMNB2

CHANG_CORE_SERUM_RESPONSE_UP PLOD2

CHANG_CORE_SERUM_RESPONSE_UP NCEH1

CHANG_CORE_SERUM_RESPONSE_UP NME1

CHANG_CORE_SERUM_RESPONSE_UP ACTL6A

CHANG_CORE_SERUM_RESPONSE_UP DLEU1

CHANG_CORE_SERUM_RESPONSE_UP SNRPA1

CHANG_CORE_SERUM_RESPONSE_UP CBX1

CHANG_CORE_SERUM_RESPONSE_UP LYAR

CHANG_CORE_SERUM_RESPONSE_UP PFN1

CHANG_CORE_SERUM_RESPONSE_UP CENPJ

CHANG_CORE_SERUM_RESPONSE_UP COTL1

CHANG_CORE_SERUM_RESPONSE_UP SPRYD7

CHANG_CORE_SERUM_RESPONSE_UP USPL1

CHANG_CORE_SERUM_RESPONSE_UP MRPL12

CHANG_CORE_SERUM_RESPONSE_UP ADAMTS1

CHANG_CORE_SERUM_RESPONSE_UP GLRX3

CHANG_CORE_SERUM_RESPONSE_UP WSB2

CHANG_CORE_SERUM_RESPONSE_UP MRPS16

CHANG_CORE_SERUM_RESPONSE_UP DCLRE1B

CHANG_CORE_SERUM_RESPONSE_UP MKKS

CHANG_CORE_SERUM_RESPONSE_UP CPEB4

CHANG_CORE_SERUM_RESPONSE_UP SPAG17

CHANG_CORE_SERUM_RESPONSE_UP UAP1

CHANG_CORE_SERUM_RESPONSE_UP COQ2

CHANG_CORE_SERUM_RESPONSE_UP WDHD1

CHANG_CORE_SERUM_RESPONSE_UP DCBLD2

CHANG_CORE_SERUM_RESPONSE_UP SAR1B

CHANG_CORE_SERUM_RESPONSE_UP PSMA7

CHANG_CORE_SERUM_RESPONSE_UP PSMC3

CHANG_CORE_SERUM_RESPONSE_UP COPS6

CHANG_CORE_SERUM_RESPONSE_UP DUT

CHANG_CORE_SERUM_RESPONSE_UP PPIH

CHANG_CORE_SERUM_RESPONSE_UP PHF19

CHANG_CORE_SERUM_RESPONSE_UP TPM2

CHANG_CORE_SERUM_RESPONSE_UP MCTS1

CHANG_CORE_SERUM_RESPONSE_UP EIF4EBP1

CHANG_CORE_SERUM_RESPONSE_UP HNRNPR

CHANG_CORE_SERUM_RESPONSE_UP APITD1

CHANG_CORE_SERUM_RESPONSE_UP FAM216A

CHANG_CORE_SERUM_RESPONSE_UP TEX30

CHANG_CORE_SERUM_RESPONSE_UP CMC2

CHANG_CORE_SERUM_RESPONSE_UP CMSS1

CHANG_CORE_SERUM_RESPONSE_UP KYAT3

CHANG_CORE_SERUM_RESPONSE_UP JPT2

CHANG_CORE_SERUM_RESPONSE_UP EMC1

CHANG_CORE_SERUM_RESPONSE_UP CENPU

CHANG_CORE_SERUM_RESPONSE_UP NUP58

CHANG_CORE_SERUM_RESPONSE_UP HACD2

CHANG_CORE_SERUM_RESPONSE_UP BHLHE40

CHANG_CORE_SERUM_RESPONSE_UP ELOC

Chemokine12_score CCL2

Chemokine12_score CCL3

Chemokine12_score CCL4

Chemokine12_score CCL5

Chemokine12_score CCL8

Chemokine12_score CCL18

Chemokine12_score CCL19

Chemokine12_score CCL21

Chemokine12_score CXCL9

Chemokine12_score CXCL10

Chemokine12_score CXCL11

Chemokine12_score CXCL13

CSF1_response CORO1A

CSF1_response MNDA

CSF1_response CCRL2

CSF1_response SLC7A7

CSF1_response HLA-DMA

CSF1_response RNASE6

CSF1_response TLR2

CSF1_response CTSC

CSF1_response LILRB4

CSF1_response CTSS

CSF1_response RASSF4

CSF1_response MSN

CSF1_response CYBB

CSF1_response LAPTM5

CSF1_response DOCK2

CSF1_response FCGR1A

CSF1_response EVI2B

CSF1_response ADCY7

CSF1_response CD48

CSF1_response ARHGAP15

CSF1_response ARRB2

CSF1_response SYK

CSF1_response BTK

CSF1_response TNFAIP3

CSF1_response FCGR2A

CSF1_response VSIG4

CSF1_response IL10RA

CSF1_response IFI16

CSF1_response ITGB2

CSF1_response IL7R

CSF1_response TBXAS1

CSF1_response FMNL1

CSF1_response FLI1

CSF1_response RASSF2

CSF1_response LYZ

CSF1_response CD163

CSF1_response CCL2

CSF1_response FCGR2B

CSF1_response MERTK

CSF1_response CD84

CSF1_response CD53

CSF1_response CD86

CSF1_response CTSL

CSF1_response EVI2A

CSF1_response TNFRSF1B

CSF1_response CXCR4

CSF1_response LCP1

CSF1_response SAMHD1

CSF1_response CPVL

CSF1_response HLA-DRB1

CSF1_response GIMAP4

CSF1_response SAMSN1

CSF1_response PLCG2

CSF1_response OSBPL3

CSF1_response CD8A

CSF1_response RUNX3

CSF1_response FCGR3A

CSF1_response AMPD3

CSF1_response MYO1F

CSF1_response LYN

CSF1_response MPP1

CSF1_response LRMP

CSF1_response FGL2

CSF1_response NCKAP1L

CSF1_response HCLS1

CSF1_response SELL

CSF1_response CASP1

CSF1_response SELPLG

CSF1_response CD33

CSF1_response GPNMB

CSF1_response NCF2

CSF1_response FNBP1

CSF1_response IL18

CSF1_response B2M

CSF1_response SP140

CSF1_response FCER1G

CSF1_response LCP2

CSF1_response LY86

CSF1_response LAIR1

CSF1_response IFI30

CSF1_response TNFSF13B

CSF1_response LST1

CSF1_response FGR

CSF1_response NPL

CSF1_response PLEK

CSF1_response CCL5

CSF1_response PTPRC

CSF1_response GNPTAB

CSF1_response SLC1A3

CSF1_response HCK

CSF1_response NPC2

CSF1_response C3AR1

CSF1_response PIK3CG

CSF1_response DAPK1

CSF1_response ALOX5AP

CSF1_response CSF1R

CSF1_response APOE

CSF1_response APOC1

CSF1_response CD52

CSF1_response LHFPL2

CSF1_response C1orf54

CSF1_response IKZF1

CSF1_response SH2B3

CSF1_response WIPF1

CSF1_response RUBCNL

CSF1_response ADGRE5

CSF1_response ADA2

CSF1_response CELF2

CSF1_response FPR3

CSF1_response FYB1

CSF1_response ARHGAP45

CSF1_response CYTIP

CSR_Activated_15701700 ADAMTS1

CSR_Activated_15701700 ADD3

CSR_Activated_15701700 APOD

CSR_Activated_15701700 APP

CSR_Activated_15701700 ARHGAP12

CSR_Activated_15701700 ARHGEF3

CSR_Activated_15701700 ATP6V0A1

CSR_Activated_15701700 AXIN2

CSR_Activated_15701700 BBC3

CSR_Activated_15701700 BCCIP

CSR_Activated_15701700 BCL6

CSR_Activated_15701700 BRCA2

CSR_Activated_15701700 BRIP1

CSR_Activated_15701700 C11ORF24

CSR_Activated_15701700 C1S

CSR_Activated_15701700 CAMLG

CSR_Activated_15701700 CBX1

CSR_Activated_15701700 CCNG2

CSR_Activated_15701700 CCNL2

CSR_Activated_15701700 CCT5

CSR_Activated_15701700 CDC14B

CSR_Activated_15701700 CDCA4

CSR_Activated_15701700 CDK2

CSR_Activated_15701700 CDKN1A

CSR_Activated_15701700 CDKN1C

CSR_Activated_15701700 CENPJ

CSR_Activated_15701700 CHEK1

CSR_Activated_15701700 CKLF

CSR_Activated_15701700 CLCN6

CSR_Activated_15701700 CLIC2

CSR_Activated_15701700 CNTNAP1

CSR_Activated_15701700 COPS6

CSR_Activated_15701700 CORO1C

CSR_Activated_15701700 COTL1

CSR_Activated_15701700 COX17

CSR_Activated_15701700 CRTAP

CSR_Activated_15701700 CSF1

CSR_Activated_15701700 CST3

CSR_Activated_15701700 CTNS

CSR_Activated_15701700 CTSF

CSR_Activated_15701700 CYP27A1

CSR_Activated_15701700 DCK

CSR_Activated_15701700 DCLRE1B

CSR_Activated_15701700 DHFR

CSR_Activated_15701700 DLEU1

CSR_Activated_15701700 DLEU2

CSR_Activated_15701700 DNM1

CSR_Activated_15701700 DPP7

CSR_Activated_15701700 DPYSL2

CSR_Activated_15701700 DUSP22

CSR_Activated_15701700 DUT

CSR_Activated_15701700 EBNA1BP2

CSR_Activated_15701700 EEF1E1

CSR_Activated_15701700 EIF4EBP1

CSR_Activated_15701700 EIF4G1

CSR_Activated_15701700 EMP2

CSR_Activated_15701700 ENO1

CSR_Activated_15701700 EPHB1

CSR_Activated_15701700 F10

CSR_Activated_15701700 F3

CSR_Activated_15701700 FABP3

CSR_Activated_15701700 FADS1

CSR_Activated_15701700 FADS2

CSR_Activated_15701700 FCGRT

CSR_Activated_15701700 FDPS

CSR_Activated_15701700 FLNC

CSR_Activated_15701700 FYCO1

CSR_Activated_15701700 GABARAPL1

CSR_Activated_15701700 GABBR1

CSR_Activated_15701700 GATM

CSR_Activated_15701700 GGH

CSR_Activated_15701700 GLS

CSR_Activated_15701700 GNG11

CSR_Activated_15701700 GPLD1

CSR_Activated_15701700 GSN

CSR_Activated_15701700 H2AFZ

CSR_Activated_15701700 HAS2

CSR_Activated_15701700 HDAC5

CSR_Activated_15701700 HECA

CSR_Activated_15701700 HMGB1

CSR_Activated_15701700 HMGCS1

CSR_Activated_15701700 HMGN2

CSR_Activated_15701700 ID2

CSR_Activated_15701700 ID3

CSR_Activated_15701700 IDI1

CSR_Activated_15701700 IFRD2

CSR_Activated_15701700 IGBP1

CSR_Activated_15701700 IL1R1

CSR_Activated_15701700 IL6ST

CSR_Activated_15701700 IL7R

CSR_Activated_15701700 IMP4

CSR_Activated_15701700 INSIG1

CSR_Activated_15701700 IPO4

CSR_Activated_15701700 ITGA6

CSR_Activated_15701700 ITPKB

CSR_Activated_15701700 JAG1

CSR_Activated_15701700 KIAA1109

CSR_Activated_15701700 KIT

CSR_Activated_15701700 KLHL5

CSR_Activated_15701700 LDB1

CSR_Activated_15701700 LDLR

CSR_Activated_15701700 LIPA

CSR_Activated_15701700 LMNB2

CSR_Activated_15701700 LOXL2

CSR_Activated_15701700 LPIN1

CSR_Activated_15701700 LRIG2

CSR_Activated_15701700 LRP1

CSR_Activated_15701700 LSM3

CSR_Activated_15701700 LSM4

CSR_Activated_15701700 LSS

CSR_Activated_15701700 LUM

CSR_Activated_15701700 LYAR

CSR_Activated_15701700 MAF

CSR_Activated_15701700 MAN1A1

CSR_Activated_15701700 MAP3K8

CSR_Activated_15701700 MAPRE1

CSR_Activated_15701700 MBP

CSR_Activated_15701700 MCM3

CSR_Activated_15701700 MCM7

CSR_Activated_15701700 MEF2D

CSR_Activated_15701700 MET

CSR_Activated_15701700 MFGE8

CSR_Activated_15701700 MKKS

CSR_Activated_15701700 MNAT1

CSR_Activated_15701700 MRPL12

CSR_Activated_15701700 MRPL37

CSR_Activated_15701700 MRPS16

CSR_Activated_15701700 MRPS28

CSR_Activated_15701700 MSN

CSR_Activated_15701700 MT1F

CSR_Activated_15701700 MT3

CSR_Activated_15701700 MTHFD1

CSR_Activated_15701700 MVD

CSR_Activated_15701700 MXI1

CSR_Activated_15701700 MYBL1

CSR_Activated_15701700 MYBL2

CSR_Activated_15701700 MYCBP

CSR_Activated_15701700 MYL6

CSR_Activated_15701700 NCOA3

CSR_Activated_15701700 NLN

CSR_Activated_15701700 NME1

CSR_Activated_15701700 NUDT1

CSR_Activated_15701700 NUP107

CSR_Activated_15701700 NUTF2

CSR_Activated_15701700 OSBPL8

CSR_Activated_15701700 PA2G4

CSR_Activated_15701700 PAICS

CSR_Activated_15701700 PBXIP1

CSR_Activated_15701700 PCSK7

CSR_Activated_15701700 PDAP1

CSR_Activated_15701700 PDK2

CSR_Activated_15701700 PFKP

CSR_Activated_15701700 PFN1

CSR_Activated_15701700 PITPNC1

CSR_Activated_15701700 PKIG

CSR_Activated_15701700 PLA2R1

CSR_Activated_15701700 PLAUR

CSR_Activated_15701700 PLD3

CSR_Activated_15701700 PLG

CSR_Activated_15701700 PLOD2

CSR_Activated_15701700 PLXNB1

CSR_Activated_15701700 PNN

CSR_Activated_15701700 POLE2

CSR_Activated_15701700 POLR3K

CSR_Activated_15701700 POR

CSR_Activated_15701700 PPIH

CSR_Activated_15701700 PSMA7

CSR_Activated_15701700 PSMC3

CSR_Activated_15701700 PSMD12

CSR_Activated_15701700 PSMD14

CSR_Activated_15701700 PSMD2

CSR_Activated_15701700 PTPRU

CSR_Activated_15701700 PTS

CSR_Activated_15701700 RARRES3

CSR_Activated_15701700 RBM14

CSR_Activated_15701700 RBMX

CSR_Activated_15701700 REV3L

CSR_Activated_15701700 RFC3

CSR_Activated_15701700 RNASEH2A

CSR_Activated_15701700 RNF138

CSR_Activated_15701700 RNF41

CSR_Activated_15701700 RPN1

CSR_Activated_15701700 RRM2B

CSR_Activated_15701700 RUVBL1

CSR_Activated_15701700 SATB1

CSR_Activated_15701700 SCD

CSR_Activated_15701700 SDC1

CSR_Activated_15701700 SELENBP1

CSR_Activated_15701700 SERPING1

CSR_Activated_15701700 SFTPB

CSR_Activated_15701700 SLC16A1

CSR_Activated_15701700 SLC25A5

CSR_Activated_15701700 SLC40A1

CSR_Activated_15701700 SLC5A3

CSR_Activated_15701700 SLPI

CSR_Activated_15701700 SMS

CSR_Activated_15701700 SMURF2

CSR_Activated_15701700 SNRPA

CSR_Activated_15701700 SNRPA1

CSR_Activated_15701700 SNRPB

CSR_Activated_15701700 SNRPC

CSR_Activated_15701700 SNRPD1

CSR_Activated_15701700 SNRPE

CSR_Activated_15701700 SQLE

CSR_Activated_15701700 SRM

CSR_Activated_15701700 SSR3

CSR_Activated_15701700 SSSCA1

CSR_Activated_15701700 STK17A

CSR_Activated_15701700 SVIL

CSR_Activated_15701700 TAGLN

CSR_Activated_15701700 TBRG1

CSR_Activated_15701700 TFPI2

CSR_Activated_15701700 TIMP2

CSR_Activated_15701700 TNFAIP2

CSR_Activated_15701700 TNFRSF12A

CSR_Activated_15701700 TNFSF12

CSR_Activated_15701700 TNXB

CSR_Activated_15701700 TOMM40

CSR_Activated_15701700 TP53INP1

CSR_Activated_15701700 TPI1

CSR_Activated_15701700 TPM1

CSR_Activated_15701700 TPM2

CSR_Activated_15701700 TRIM22

CSR_Activated_15701700 TUBG1

CSR_Activated_15701700 UAP1

CSR_Activated_15701700 UBE2J1

CSR_Activated_15701700 UMPS

CSR_Activated_15701700 VAMP4

CSR_Activated_15701700 VDAC1

CSR_Activated_15701700 WBP2

CSR_Activated_15701700 WDHD1

CSR_Activated_15701700 WSB1

CSR_Activated_15701700 WSB2

CSR_Activated_15701700 WTAP

CSR_Activated_15701700 ZNF219

CSR_Activated_15701700 ZNF83

CSR_Activated_15701700 ACSS2

CSR_Activated_15701700 ACIN1

CSR_Activated_15701700 WDR1

CSR_Activated_15701700 ARMCX3

CSR_Activated_15701700 RHOC

CSR_Activated_15701700 ACTL6A

CSR_Activated_15701700 CFB

CSR_Activated_15701700 PHF21A

CSR_Activated_15701700 BHLHE40

CSR_Activated_15701700 CENPN

CSR_Activated_15701700 SLC22A17

CSR_Activated_15701700 NRIP3

CSR_Activated_15701700 SPRYD7

CSR_Activated_15701700 MRTO4

CSR_Activated_15701700 FAM210B

CSR_Activated_15701700 FAXDC2

CSR_Activated_15701700 VTA1

CSR_Activated_15701700 FAM167A

CSR_Activated_15701700 TPRKB

CSR_Activated_15701700 COQ2

CSR_Activated_15701700 CCPG1

CSR_Activated_15701700 CMC2

CSR_Activated_15701700 DEPP1

CSR_Activated_15701700 BDH2

CSR_Activated_15701700 DKFZP434B103

CSR_Activated_15701700 FAM198B

CSR_Activated_15701700 DKFZP434O1427

CSR_Activated_15701700 DKFZP586A0522

CSR_Activated_15701700 DKFZP727G051

CSR_Activated_15701700 DKFZP761L1417

CSR_Activated_15701700 DKFZP762H185

CSR_Activated_15701700 PDLIM7

CSR_Activated_15701700 PDIA4

CSR_Activated_15701700 DCBLD2

CSR_Activated_15701700 ACSL3

CSR_Activated_15701700 FARSA

CSR_Activated_15701700 FLJ10036

CSR_Activated_15701700 FLJ10292

CSR_Activated_15701700 FLJ10407

CSR_Activated_15701700 FLJ10618

CSR_Activated_15701700 FLJ10849

CSR_Activated_15701700 FLJ10948

CSR_Activated_15701700 FLJ10983

CSR_Activated_15701700 FLJ11286

CSR_Activated_15701700 FLJ12643

CSR_Activated_15701700 FLJ12953

CSR_Activated_15701700 FLJ14525

CSR_Activated_15701700 FLJ20059

CSR_Activated_15701700 FLJ20154

CSR_Activated_15701700 FLJ20331

CSR_Activated_15701700 FLJ21016

CSR_Activated_15701700 FLJ21986

CSR_Activated_15701700 FLJ23462

CSR_Activated_15701700 FLJ23468

CSR_Activated_15701700 FLJ30532

CSR_Activated_15701700 FLJ30574

CSR_Activated_15701700 FLJ31033

CSR_Activated_15701700 FLJ32731

CSR_Activated_15701700 FLJ32915

CSR_Activated_15701700 FLJ90754

CSR_Activated_15701700 FLJ90798

CSR_Activated_15701700 FARSB

CSR_Activated_15701700 TNFAIP8

CSR_Activated_15701700 ADGRA2

CSR_Activated_15701700 H2AFV

CSR_Activated_15701700 JPT2

CSR_Activated_15701700 HNRNPA2B1

CSR_Activated_15701700 HNRNPR

CSR_Activated_15701700 KRR1

CSR_Activated_15701700 EIF2AK1

CSR_Activated_15701700 NOP16

CSR_Activated_15701700 FAM216A

CSR_Activated_15701700 AIMP2

CSR_Activated_15701700 CD82

CSR_Activated_15701700 ERLIN1

CSR_Activated_15701700 EMC1

CSR_Activated_15701700 NUP93

CSR_Activated_15701700 KHNYN

CSR_Activated_15701700 TRANK1

CSR_Activated_15701700 PRUNE2

CSR_Activated_15701700 ANKRD12

CSR_Activated_15701700 VASH1

CSR_Activated_15701700 ESYT2

CSR_Activated_15701700 PARP14

CSR_Activated_15701700 NYNRIN

CSR_Activated_15701700 NCEH1

CSR_Activated_15701700 CALCOCO1

CSR_Activated_15701700 RNF213

CSR_Activated_15701700 SH3BP5L

CSR_Activated_15701700 FAM171B

CSR_Activated_15701700 MCP

CSR_Activated_15701700 MCTS1

CSR_Activated_15701700 WDR77

CSR_Activated_15701700 C19orf48

CSR_Activated_15701700 APOO

CSR_Activated_15701700 ERRFI1

CSR_Activated_15701700 ARID5B

CSR_Activated_15701700 NUDT15

CSR_Activated_15701700 ISCU

CSR_Activated_15701700 NHP2

CSR_Activated_15701700 NUP58

CSR_Activated_15701700 EXOSC8

CSR_Activated_15701700 NUPR1

CSR_Activated_15701700 SESN1

CSR_Activated_15701700 NUP85

CSR_Activated_15701700 CPQ

CSR_Activated_15701700 ACCS

CSR_Activated_15701700 KDM5B

CSR_Activated_15701700 PNRC1

CSR_Activated_15701700 RAB11FIP2

CSR_Activated_15701700 RPP40

CSR_Activated_15701700 MSMO1

CSR_Activated_15701700 NPTN

CSR_Activated_15701700 PDZRN3

CSR_Activated_15701700 SESN2

CSR_Activated_15701700 TRA2B

CSR_Activated_15701700 SRSF2

CSR_Activated_15701700 SLC35E2B

CSR_Activated_15701700 SMC2

CSR_Activated_15701700 SRRM1

CSR_Activated_15701700 SQOR

CSR_Activated_15701700 PLK4

CSR_Activated_15701700 ELOC

CSR_Activated_15701700 DYNLT1

CSR_Activated_15701700 TIMM50

CSR_Activated_15701700 TSC22D1

CSR_Activated_15701700 TUBA4A

CSR_Activated_15701700 GLRX3

CSR_Activated_15701700 CMPK1

CSR_Activated_15701700 EZR

CSR_Activated_15701700 ZNF106

CSR_Activated_15701700 ZBTB17

CSR_Activated_15701700 ZKSCAN1

CTLA4_data CTLA4

DAP12_data TYROBP

G_CD3E CD3E

G_CD3E CD3D

G_CD3E IL10RA

G_CD3E CD3G

G_CD3E JAML

G_CYTH4 CYTH4

G_CYTH4 NCF4

G_CYTH4 RAC2

G_CYTH4 IL2RB

G_CYTH4 CSF2RB

G_GIMAP4 GIMAP4

G_GIMAP4 GIMAP5

G_GIMAP4 GIMAP6

G_GIMAP4 GIMAP8

G_GIMAP4 GIMAP7

G_GIMAP4 GIMAP1

G_HLA_DPA1 HLA-DPA1

G_HLA_DPA1 HLA-DRA

G_HLA_DPA1 HLA-DPB1

G_HLA_DPA1 HLA-DRB1

G_HLA_DPA1 HLA-DMB

G_HLA_DPA1 HLA-DMA

G_HLA_DPA1 HLA-DOA

G_HLA_DPA1 HLA-DQA1

G_HLA_DPA1 HLA-DQB1

G_HLA_DPA1 HLA-DRB5

G_LILRB4 LILRB4

G_LILRB4 LILRB2

G_LILRB4 LAIR1

G_LILRB4 LILRB1

G_LILRB4 LILRB3

G_LILRB4 LILRA6

G_LILRB4 OSCAR

G_SIGLEC9 SIGLEC9

G_SIGLEC9 SIGLEC7

G_SIGLEC9 FPR3

G_SIGLEC9 SIGLEC5

G_SIGLEC9 CD33

G_SIGLEC9 SIGLEC10

G_SIGLEC9 SIGLEC14

G_SIGLEC9 FPR1

G_SLAMF6 SLAMF6

G_SLAMF6 SLAMF7

G_SLAMF6 CD48

G_SLAMF6 LY9

G_SLAMF6 SLAMF1

G_SLAMF6 ARHGAP30

G_SLAMF6 SLAMF8

GRANS_PCA_16704732 SPTBN2

GRANS_PCA_16704732 SLPI

GRANS_PCA_16704732 CMTM6

GRANS_PCA_16704732 SLC9A7

GRANS_PCA_16704732 SLC25A37

GRANS_PCA_16704732 GABARAP

GRANS_PCA_16704732 QPCT

GRANS_PCA_16704732 PARPBP

GRANS_PCA_16704732 AOX1

GRANS_PCA_16704732 PPM1A

GRANS_PCA_16704732 RER1

GRANS_PCA_16704732 LAMP2

GRANS_PCA_16704732 SGCE

GRANS_PCA_16704732 OSBP2

GRANS_PCA_16704732 FAXDC2

GRANS_PCA_16704732 TGFA

GRANS_PCA_16704732 LRRK2

GRANS_PCA_16704732 SPRTN

GRANS_PCA_16704732 NFIA

GRANS_PCA_16704732 MOG

GRANS_PCA_16704732 BNIP3L

GRANS_PCA_16704732 CDA

GRANS_PCA_16704732 AGO4

GRANS_PCA_16704732 CAMSAP2

GRANS_PCA_16704732 TNFRSF10A

GRANS_PCA_16704732 CYP4F3

GRANS_PCA_16704732 ELF4

GRANS_PCA_16704732 RAB2B

GRANS_PCA_16704732 TST

GRANS_PCA_16704732 TBC1D10C

GRANS_PCA_16704732 IL18RAP

GRANS_PCA_16704732 BST2

GRANS_PCA_16704732 FIS1

GRANS_PCA_16704732 SERPINE1

GRANS_PCA_16704732 ATP6V0C

GRANS_PCA_16704732 SLC31A2

GRANS_PCA_16704732 ARRB2

GRANS_PCA_16704732 ZNF598

GRANS_PCA_16704732 RTN3

GRANS_PCA_16704732 OAZ1

GRANS_PCA_16704732 GYPB

GRANS_PCA_16704732 USP47

GRANS_PCA_16704732 CHI3L1

GRANS_PCA_16704732 C9orf78

GRANS_PCA_16704732 BACH1

GRANS_PCA_16704732 SLC7A7

GRANS_PCA_16704732 ALOX5AP

GRANS_PCA_16704732 HLA-E

GRANS_PCA_16704732 TNFRSF10C

GRANS_PCA_16704732 TSPAN7

GRANS_PCA_16704732 MX2

GRANS_PCA_16704732 CLEC2D

GRANS_PCA_16704732 PIP

GRANS_PCA_16704732 MECOM

GRANS_PCA_16704732 SELENBP1

GRANS_PCA_16704732 TSPAN2

GRANS_PCA_16704732 GLIPR1

GRANS_PCA_16704732 SMOX

GRANS_PCA_16704732 CCL4L1

GRANS_PCA_16704732 ALPL

GRANS_PCA_16704732 FCGRT

GRANS_PCA_16704732 BIN2

GRANS_PCA_16704732 ANAPC16

GRANS_PCA_16704732 SDCBP

GRANS_PCA_16704732 DLX4

GRANS_PCA_16704732 DHRS7

GRANS_PCA_16704732 FTL

GRANS_PCA_16704732 C16orf72

GRANS_PCA_16704732 CPQ

GRANS_PCA_16704732 SLC7A5P1

GRANS_PCA_16704732 PYGL

GRANS_PCA_16704732 ICAM3

GRANS_PCA_16704732 SLC45A4

GRANS_PCA_16704732 C9orf40

GRANS_PCA_16704732 XK

GRANS_PCA_16704732 STX3

GRANS_PCA_16704732 PSMB3

GRANS_PCA_16704732 DEFA1

GRANS_PCA_16704732 DYSF

GRANS_PCA_16704732 CDK18

GRANS_PCA_16704732 GSPT1

GRANS_PCA_16704732 NDE1

GRANS_PCA_16704732 SLC15A4

GRANS_PCA_16704732 TAS2R38

GRANS_PCA_16704732 PLEKHG3

GRANS_PCA_16704732 DUSP6

GRANS_PCA_16704732 ST14

GRANS_PCA_16704732 S100A11

GRANS_PCA_16704732 FAM104A

GRANS_PCA_16704732 PPM1D

GRANS_PCA_16704732 RAC1

GRANS_PCA_16704732 FKBP8

GRANS_PCA_16704732 RGS20

GRANS_PCA_16704732 GRINA

GRANS_PCA_16704732 IDH2

GRANS_PCA_16704732 IL13RA1

GRANS_PCA_16704732 HCAR2

GRANS_PCA_16704732 PLEK2

GRANS_PCA_16704732 BCL2L1

GRANS_PCA_16704732 SLC25A44

GRANS_PCA_16704732 ANKIB1

GRANS_PCA_16704732 GNB2

GRANS_PCA_16704732 HSPA1A

GRANS_PCA_16704732 TIAM2

GRANS_PCA_16704732 CSF3R

GRANS_PCA_16704732 CD46

GRANS_PCA_16704732 NFE2

GRANS_PCA_16704732 CHI3L2

GRANS_PCA_16704732 S100P

GRANS_PCA_16704732 TALDO1

GRANS_PCA_16704732 NAGA

GRANS_PCA_16704732 SAP30

GRANS_PCA_16704732 C3AR1

GRANS_PCA_16704732 TM4SF1

GRANS_PCA_16704732 TMCC2

GRANS_PCA_16704732 VNN2

GRANS_PCA_16704732 RIPK2

GRANS_PCA_16704732 PDK1

GRANS_PCA_16704732 NRBF2

GRANS_PCA_16704732 ACVR1B

GRANS_PCA_16704732 SYNE2

GRANS_PCA_16704732 ZNF185

GRANS_PCA_16704732 CXCR2

GRANS_PCA_16704732 CAMP

GRANS_PCA_16704732 PSMB9

GRANS_PCA_16704732 SNCA

GRANS_PCA_16704732 IKBKG

GRANS_PCA_16704732 WASH5P

GRANS_PCA_16704732 DHRS9

GRANS_PCA_16704732 CHST15

GRANS_PCA_16704732 MKRN1

GRANS_PCA_16704732 KCTD1

GRANS_PCA_16704732 CD1B

GRANS_PCA_16704732 TIGD3

GRANS_PCA_16704732 RPS2

GRANS_PCA_16704732 LYZ

GRANS_PCA_16704732 CREB5

GRANS_PCA_16704732 RPS6KA5

GRANS_PCA_16704732 LGALS3BP

GRANS_PCA_16704732 TMCC3

GRANS_PCA_16704732 DIRAS3

GRANS_PCA_16704732 CSF2RB

GRANS_PCA_16704732 DNAJC8

GRANS_PCA_16704732 OXCT1

GRANS_PCA_16704732 CTSC

GRANS_PCA_16704732 IQSEC1

GRANS_PCA_16704732 PRC1

GRANS_PCA_16704732 IL1R2

GRANS_PCA_16704732 ANXA5

GRANS_PCA_16704732 EMC3

GRANS_PCA_16704732 ERRFI1

GRANS_PCA_16704732 CITED2

GRANS_PCA_16704732 YBX3

GRANS_PCA_16704732 IMPA2

GRANS_PCA_16704732 LDHB

GRANS_PCA_16704732 PLBD1

GRANS_PCA_16704732 ALDH2

GRANS_PCA_16704732 CD48

GRANS_PCA_16704732 GUK1

GRANS_PCA_16704732 NINJ2

GRANS_PCA_16704732 MPP1

GRANS_PCA_16704732 LRRN3

GRANS_PCA_16704732 RFXANK

GRANS_PCA_16704732 RASSF2

GRANS_PCA_16704732 PICALM

GRANS_PCA_16704732 SH3GLB1

GRANS_PCA_16704732 CDC34

GRANS_PCA_16704732 NCOA4

GRANS_PCA_16704732 ACOX1

GRANS_PCA_16704732 MYD88

GRANS_PCA_16704732 SLC22A4

GRANS_PCA_16704732 RNASE2

GRANS_PCA_16704732 SEC14L1

GRANS_PCA_16704732 ACSL1

GRANS_PCA_16704732 PLXNC1

GRANS_PCA_16704732 UBN1

GRANS_PCA_16704732 FAM129A

GRANS_PCA_16704732 RXRA

GRANS_PCA_16704732 C20orf194

GRANS_PCA_16704732 AQP9

GRANS_PCA_16704732 CDIPT

GRANS_PCA_16704732 TMEM140

GRANS_PCA_16704732 GLUL

GRANS_PCA_16704732 NXPH3

GRANS_PCA_16704732 MAP2K3

GRANS_PCA_16704732 FMOD

GRANS_PCA_16704732 CPVL

GRANS_PCA_16704732 HLX

GRANS_PCA_16704732 TM9SF2

GRANS_PCA_16704732 OLIG1

GRANS_PCA_16704732 JAZF1

GRANS_PCA_16704732 MARVELD2

GRANS_PCA_16704732 CCDC91

GRANS_PCA_16704732 DCAF12

GRANS_PCA_16704732 BEST1

GRANS_PCA_16704732 DNASE1

GRANS_PCA_16704732 ANK2

GRANS_PCA_16704732 NPL

GRANS_PCA_16704732 ITM2B

GRANS_PCA_16704732 ST6GALNAC2

GRANS_PCA_16704732 TLR8

GRANS_PCA_16704732 ABHD5

GRANS_PCA_16704732 ABTB1

GRANS_PCA_16704732 GSN

GRANS_PCA_16704732 IFIT2

GRANS_PCA_16704732 MSRA

GRANS_PCA_16704732 GATA6

GRANS_PCA_16704732 TECPR2

GRANS_PCA_16704732 UBE2B

GRANS_PCA_16704732 TSTD2

GRANS_PCA_16704732 MIIP

GRANS_PCA_16704732 MB

GRANS_PCA_16704732 FGL2

GRANS_PCA_16704732 GRN

GRANS_PCA_16704732 ARAP3

GRANS_PCA_16704732 LGALS3

GRANS_PCA_16704732 ZIC2

GRANS_PCA_16704732 HBG1

GRANS_PCA_16704732 SLA

GRANS_PCA_16704732 RGS2

GRANS_PCA_16704732 NAMPT

GRANS_PCA_16704732 STX6

GRANS_PCA_16704732 UBE2L6

GRANS_PCA_16704732 CA2

GRANS_PCA_16704732 SERPING1

GRANS_PCA_16704732 VMP1

GRANS_PCA_16704732 MFSD1

GRANS_PCA_16704732 PCK1

GRANS_PCA_16704732 LOC644462

GRANS_PCA_16704732 MYCN

GRANS_PCA_16704732 PIM1

GRANS_PCA_16704732 SPOPL

GRANS_PCA_16704732 SIGLEC5

GRANS_PCA_16704732 PIGS

GRANS_PCA_16704732 LIMK2

GRANS_PCA_16704732 TMEM259

GRANS_PCA_16704732 EVI2B

GRANS_PCA_16704732 PAK1

GRANS_PCA_16704732 CREG1

GRANS_PCA_16704732 UBXN6

GRANS_PCA_16704732 ART4

GRANS_PCA_16704732 EIF2AK1

GRANS_PCA_16704732 ALAS2

GRANS_PCA_16704732 LMO2

GRANS_PCA_16704732 CNPPD1

GRANS_PCA_16704732 APOC2

GRANS_PCA_16704732 FCGR3A

GRANS_PCA_16704732 PI3

GRANS_PCA_16704732 PGD

GRANS_PCA_16704732 TBX19

GRANS_PCA_16704732 SIAH2

GRANS_PCA_16704732 IFITM2

GRANS_PCA_16704732 COL16A1

GRANS_PCA_16704732 MME

GRANS_PCA_16704732 RAB9A

GRANS_PCA_16704732 SNX27

GRANS_PCA_16704732 CHMP2A

GRANS_PCA_16704732 CD302

GRANS_PCA_16704732 FAM214B

GRANS_PCA_16704732 KLF1

GRANS_PCA_16704732 AOC1

GRANS_PCA_16704732 SOD2

GRANS_PCA_16704732 CMBL

GRANS_PCA_16704732 GMPR

GRANS_PCA_16704732 ATP6V1B2

GRANS_PCA_16704732 NBPF12

GRANS_PCA_16704732 LYN

GRANS_PCA_16704732 NCF2

GRANS_PCA_16704732 MXD1

GRANS_PCA_16704732 ATP6V0B

GRANS_PCA_16704732 BCL6

GRANS_PCA_16704732 IL1RN

GRANS_PCA_16704732 KLHL12

GRANS_PCA_16704732 MET

GRANS_PCA_16704732 CHP1

GRANS_PCA_16704732 ZNF599

GRANS_PCA_16704732 SOAT1

GRANS_PCA_16704732 SEC24A

GRANS_PCA_16704732 GCA

GRANS_PCA_16704732 PPP1R18

GRANS_PCA_16704732 FPR1

GRANS_PCA_16704732 RBM7

GRANS_PCA_16704732 DCLRE1C

GRANS_PCA_16704732 CD40

GRANS_PCA_16704732 DOCK5

GRANS_PCA_16704732 CNGB3

GRANS_PCA_16704732 ADM

GRANS_PCA_16704732 RAB10

GRANS_PCA_16704732 HPCA

GRANS_PCA_16704732 SH3BGRL3

GRANS_PCA_16704732 TPPP2

GRANS_PCA_16704732 H3F3A

GRANS_PCA_16704732 MAPK1

GRANS_PCA_16704732 MAD2L1BP

GRANS_PCA_16704732 SLC25A39

GRANS_PCA_16704732 TSPAN5

GRANS_PCA_16704732 CBX4

GRANS_PCA_16704732 TMCC1

GRANS_PCA_16704732 MMP9

GRANS_PCA_16704732 TTR

GRANS_PCA_16704732 CELF2

GRANS_PCA_16704732 KRT8

GRANS_PCA_16704732 NPRL3

GRANS_PCA_16704732 RFX1

GRANS_PCA_16704732 MCTP2

GRANS_PCA_16704732 SRGN

GRANS_PCA_16704732 TIMM21

GRANS_PCA_16704732 SUMF1

GRANS_PCA_16704732 EPB42

GRANS_PCA_16704732 LINGO2

GRANS_PCA_16704732 LECT2

GRANS_PCA_16704732 CTSS

GRANS_PCA_16704732 MAP1A

GRANS_PCA_16704732 INHBB

GRANS_PCA_16704732 SERPINB7

GRANS_PCA_16704732 WT1

GRANS_PCA_16704732 LILRA2

GRANS_PCA_16704732 PRSS50

GRANS_PCA_16704732 MOB3A

GRANS_PCA_16704732 RWDD3

GRANS_PCA_16704732 ZDHHC18

GRANS_PCA_16704732 PPP1R3D

GRANS_PCA_16704732 CETN3

GRANS_PCA_16704732 KCNJ2

GRANS_PCA_16704732 PPM1F

GRANS_PCA_16704732 NR1H4

GRANS_PCA_16704732 RNPEP

GRANS_PCA_16704732 MNDA

GRANS_PCA_16704732 MYADM

GRANS_PCA_16704732 PCSK1

GRANS_PCA_16704732 TFE3

GRANS_PCA_16704732 RAD50

GRANS_PCA_16704732 MEGF9

GRANS_PCA_16704732 PGAM1

GRANS_PCA_16704732 GPX1

GRANS_PCA_16704732 TLR1

GRANS_PCA_16704732 XPO6

GRANS_PCA_16704732 43160

GRANS_PCA_16704732 NATD1

GRANS_PCA_16704732 MRNIP

GRANS_PCA_16704732 TMEM121B

GRANS_PCA_16704732 GSDME

GRANS_PCA_16704732 ADGRE2

GRANS_PCA_16704732 LOC102724813

GRANS_PCA_16704732 NOV

GRANS_PCA_16704732 THUMPD3-AS1

GRANS_PCA_16704732 SQOR

HER2_Immune_PCA_18006808 AOC1

HER2_Immune_PCA_18006808 ACTN1

HER2_Immune_PCA_18006808 ACVR1

HER2_Immune_PCA_18006808 ACVRL1

HER2_Immune_PCA_18006808 ADAM8

HER2_Immune_PCA_18006808 AGER

HER2_Immune_PCA_18006808 AIF1

HER2_Immune_PCA_18006808 ALDH1A1

HER2_Immune_PCA_18006808 ALDOB

HER2_Immune_PCA_18006808 AKR1B1

HER2_Immune_PCA_18006808 ALOX5AP

HER2_Immune_PCA_18006808 AMPD1

HER2_Immune_PCA_18006808 AMPD3

HER2_Immune_PCA_18006808 BIN1

HER2_Immune_PCA_18006808 ANG

HER2_Immune_PCA_18006808 ANGPT2

HER2_Immune_PCA_18006808 ANK1

HER2_Immune_PCA_18006808 AOAH

HER2_Immune_PCA_18006808 APBA2

HER2_Immune_PCA_18006808 BIRC3

HER2_Immune_PCA_18006808 APOC4

HER2_Immune_PCA_18006808 APOE

HER2_Immune_PCA_18006808 FASLG

HER2_Immune_PCA_18006808 RHOG

HER2_Immune_PCA_18006808 ARHGAP1

HER2_Immune_PCA_18006808 ARRB2

HER2_Immune_PCA_18006808 ATP2A1

HER2_Immune_PCA_18006808 B2M

HER2_Immune_PCA_18006808 BCL2A1

HER2_Immune_PCA_18006808 TNFRSF17

HER2_Immune_PCA_18006808 BDH1

HER2_Immune_PCA_18006808 CXCR5

HER2_Immune_PCA_18006808 BNIP3

HER2_Immune_PCA_18006808 DST

HER2_Immune_PCA_18006808 BTK

HER2_Immune_PCA_18006808 BTN1A1

HER2_Immune_PCA_18006808 SERPING1

HER2_Immune_PCA_18006808 C1QA

HER2_Immune_PCA_18006808 C1QB

HER2_Immune_PCA_18006808 C1R

HER2_Immune_PCA_18006808 C1S

HER2_Immune_PCA_18006808 C2

HER2_Immune_PCA_18006808 C3

HER2_Immune_PCA_18006808 C3AR1

HER2_Immune_PCA_18006808 FMNL1

HER2_Immune_PCA_18006808 CAPN2

HER2_Immune_PCA_18006808 CASP1

HER2_Immune_PCA_18006808 CASP4

HER2_Immune_PCA_18006808 RUNX1

HER2_Immune_PCA_18006808 RUNX3

HER2_Immune_PCA_18006808 CCND2

HER2_Immune_PCA_18006808 CCND3

HER2_Immune_PCA_18006808 CD1D

HER2_Immune_PCA_18006808 CD1E

HER2_Immune_PCA_18006808 CD2

HER2_Immune_PCA_18006808 CD3D

HER2_Immune_PCA_18006808 CD3E

HER2_Immune_PCA_18006808 CD3G

HER2_Immune_PCA_18006808 CD247

HER2_Immune_PCA_18006808 CD6

HER2_Immune_PCA_18006808 CD7

HER2_Immune_PCA_18006808 CD8A

HER2_Immune_PCA_18006808 CD8B

HER2_Immune_PCA_18006808 CD14

HER2_Immune_PCA_18006808 CD19

HER2_Immune_PCA_18006808 MS4A1

HER2_Immune_PCA_18006808 CD22

HER2_Immune_PCA_18006808 CD27

HER2_Immune_PCA_18006808 CD28

HER2_Immune_PCA_18006808 CD80

HER2_Immune_PCA_18006808 CD86

HER2_Immune_PCA_18006808 CD33

HER2_Immune_PCA_18006808 CD37

HER2_Immune_PCA_18006808 CD38

HER2_Immune_PCA_18006808 ENTPD1

HER2_Immune_PCA_18006808 CD40

HER2_Immune_PCA_18006808 CD47

HER2_Immune_PCA_18006808 CD48

HER2_Immune_PCA_18006808 CD53

HER2_Immune_PCA_18006808 CD69

HER2_Immune_PCA_18006808 CD72

HER2_Immune_PCA_18006808 CD74

HER2_Immune_PCA_18006808 CD79A

HER2_Immune_PCA_18006808 CD79B

HER2_Immune_PCA_18006808 CDKN2D

HER2_Immune_PCA_18006808 CDS1

HER2_Immune_PCA_18006808 CD52

HER2_Immune_PCA_18006808 CTSC

HER2_Immune_PCA_18006808 CHIT1

HER2_Immune_PCA_18006808 CLIC2

HER2_Immune_PCA_18006808 CCR1

HER2_Immune_PCA_18006808 CCR5

HER2_Immune_PCA_18006808 CCR6

HER2_Immune_PCA_18006808 CCR7

HER2_Immune_PCA_18006808 CMKLR1

HER2_Immune_PCA_18006808 COL4A1

HER2_Immune_PCA_18006808 COL4A3

HER2_Immune_PCA_18006808 COL10A1

HER2_Immune_PCA_18006808 COL11A1

HER2_Immune_PCA_18006808 CSF1R

HER2_Immune_PCA_18006808 CSF2RA

HER2_Immune_PCA_18006808 CSF2RB

HER2_Immune_PCA_18006808 CSK

HER2_Immune_PCA_18006808 CSNK1A1

HER2_Immune_PCA_18006808 CSRP1

HER2_Immune_PCA_18006808 CST3

HER2_Immune_PCA_18006808 CTLA4

HER2_Immune_PCA_18006808 CTSG

HER2_Immune_PCA_18006808 CTSH

HER2_Immune_PCA_18006808 CTSS

HER2_Immune_PCA_18006808 CTSW

HER2_Immune_PCA_18006808 CYBA

HER2_Immune_PCA_18006808 CYBB

HER2_Immune_PCA_18006808 CYLD

HER2_Immune_PCA_18006808 CYP11A1

HER2_Immune_PCA_18006808 DGKA

HER2_Immune_PCA_18006808 DBN1

HER2_Immune_PCA_18006808 DNASE1L3

HER2_Immune_PCA_18006808 DNMT1

HER2_Immune_PCA_18006808 DOCK2

HER2_Immune_PCA_18006808 DOK1

HER2_Immune_PCA_18006808 DRP2

HER2_Immune_PCA_18006808 DTNB

HER2_Immune_PCA_18006808 GPR183

HER2_Immune_PCA_18006808 EFNA1

HER2_Immune_PCA_18006808 EFNB1

HER2_Immune_PCA_18006808 EMP3

HER2_Immune_PCA_18006808 ENPEP

HER2_Immune_PCA_18006808 EPHB2

HER2_Immune_PCA_18006808 EPOR

HER2_Immune_PCA_18006808 EVC

HER2_Immune_PCA_18006808 EVI2B

HER2_Immune_PCA_18006808 F13B

HER2_Immune_PCA_18006808 PTK2B

HER2_Immune_PCA_18006808 FAU

HER2_Immune_PCA_18006808 FCER1G

HER2_Immune_PCA_18006808 FCGRT

HER2_Immune_PCA_18006808 FCN1

HER2_Immune_PCA_18006808 FGR

HER2_Immune_PCA_18006808 FHL2

HER2_Immune_PCA_18006808 FOXO1

HER2_Immune_PCA_18006808 FLI1

HER2_Immune_PCA_18006808 FLT3LG

HER2_Immune_PCA_18006808 FOLR2

HER2_Immune_PCA_18006808 FPR1

HER2_Immune_PCA_18006808 FUCA1

HER2_Immune_PCA_18006808 FUT4

HER2_Immune_PCA_18006808 FYN

HER2_Immune_PCA_18006808 G6PC

HER2_Immune_PCA_18006808 GBP1

HER2_Immune_PCA_18006808 GBP2

HER2_Immune_PCA_18006808 GFI1

HER2_Immune_PCA_18006808 GFPT1

HER2_Immune_PCA_18006808 GNA11

HER2_Immune_PCA_18006808 GNG7

HER2_Immune_PCA_18006808 CXCR3

HER2_Immune_PCA_18006808 GPR15

HER2_Immune_PCA_18006808 GPR18

HER2_Immune_PCA_18006808 GPR27

HER2_Immune_PCA_18006808 GPR35

HER2_Immune_PCA_18006808 GRK5

HER2_Immune_PCA_18006808 GRK6

HER2_Immune_PCA_18006808 GRN

HER2_Immune_PCA_18006808 NR3C1

HER2_Immune_PCA_18006808 GRP

HER2_Immune_PCA_18006808 GSPT1

HER2_Immune_PCA_18006808 GYPC

HER2_Immune_PCA_18006808 GZMH

HER2_Immune_PCA_18006808 GZMA

HER2_Immune_PCA_18006808 GZMB

HER2_Immune_PCA_18006808 GZMK

HER2_Immune_PCA_18006808 GZMM

HER2_Immune_PCA_18006808 HBBP1

HER2_Immune_PCA_18006808 HCK

HER2_Immune_PCA_18006808 HCLS1

HER2_Immune_PCA_18006808 NCKAP1L

HER2_Immune_PCA_18006808 CFHR2

HER2_Immune_PCA_18006808 HHEX

HER2_Immune_PCA_18006808 HLA-A

HER2_Immune_PCA_18006808 HLA-B

HER2_Immune_PCA_18006808 HLA-DMA

HER2_Immune_PCA_18006808 HLA-DMB

HER2_Immune_PCA_18006808 HLA-DOA

HER2_Immune_PCA_18006808 HLA-DOB

HER2_Immune_PCA_18006808 HLA-DPA1

HER2_Immune_PCA_18006808 HLA-DPB1

HER2_Immune_PCA_18006808 HLA-DQA1

HER2_Immune_PCA_18006808 HLA-DQB1

HER2_Immune_PCA_18006808 HLA-DRA

HER2_Immune_PCA_18006808 HLA-DRB1

HER2_Immune_PCA_18006808 HLA-E

HER2_Immune_PCA_18006808 HLA-G

HER2_Immune_PCA_18006808 HNRNPH2

HER2_Immune_PCA_18006808 HSD11B1

HER2_Immune_PCA_18006808 HSPA1B

HER2_Immune_PCA_18006808 HTR5A

HER2_Immune_PCA_18006808 ICAM2

HER2_Immune_PCA_18006808 ICAM3

HER2_Immune_PCA_18006808 IRF8

HER2_Immune_PCA_18006808 IFI35

HER2_Immune_PCA_18006808 SP110

HER2_Immune_PCA_18006808 IFNAR2

HER2_Immune_PCA_18006808 IFNG

HER2_Immune_PCA_18006808 IGHA1

HER2_Immune_PCA_18006808 IGHD

HER2_Immune_PCA_18006808 IGHM

HER2_Immune_PCA_18006808 IGLC1

HER2_Immune_PCA_18006808 IGLL1

HER2_Immune_PCA_18006808 IL2RB

HER2_Immune_PCA_18006808 IL2RG

HER2_Immune_PCA_18006808 IL6R

HER2_Immune_PCA_18006808 IL7

HER2_Immune_PCA_18006808 IL7R

HER2_Immune_PCA_18006808 CXCR2

HER2_Immune_PCA_18006808 IL9R

HER2_Immune_PCA_18006808 IL10RA

HER2_Immune_PCA_18006808 IL15

HER2_Immune_PCA_18006808 IL15RA

HER2_Immune_PCA_18006808 IL16

HER2_Immune_PCA_18006808 TNFRSF9

HER2_Immune_PCA_18006808 IDO1

HER2_Immune_PCA_18006808 CXCL10

HER2_Immune_PCA_18006808 INPP5A

HER2_Immune_PCA_18006808 INPP5D

HER2_Immune_PCA_18006808 IRF1

HER2_Immune_PCA_18006808 IRF4

HER2_Immune_PCA_18006808 IRF5

HER2_Immune_PCA_18006808 IRF6

HER2_Immune_PCA_18006808 IRF7

HER2_Immune_PCA_18006808 ISG20

HER2_Immune_PCA_18006808 ITGA4

HER2_Immune_PCA_18006808 ITGAM

HER2_Immune_PCA_18006808 ITGB2

HER2_Immune_PCA_18006808 ITGB7

HER2_Immune_PCA_18006808 ITK

HER2_Immune_PCA_18006808 JAK2

HER2_Immune_PCA_18006808 KIF2A

HER2_Immune_PCA_18006808 KIR2DL4

HER2_Immune_PCA_18006808 KLRB1

HER2_Immune_PCA_18006808 KLRC1

HER2_Immune_PCA_18006808 KLRC3

HER2_Immune_PCA_18006808 KLRD1

HER2_Immune_PCA_18006808 KRTAP5-9

HER2_Immune_PCA_18006808 LAG3

HER2_Immune_PCA_18006808 LAIR1

HER2_Immune_PCA_18006808 LAIR2

HER2_Immune_PCA_18006808 LAMC1

HER2_Immune_PCA_18006808 LCK

HER2_Immune_PCA_18006808 LCP1

HER2_Immune_PCA_18006808 LCP2

HER2_Immune_PCA_18006808 LEPR

HER2_Immune_PCA_18006808 LGALS2

HER2_Immune_PCA_18006808 LGALS9

HER2_Immune_PCA_18006808 LMO2

HER2_Immune_PCA_18006808 LOXL2

HER2_Immune_PCA_18006808 LRMP

HER2_Immune_PCA_18006808 LRCH4

HER2_Immune_PCA_18006808 LSP1

HER2_Immune_PCA_18006808 LTB

HER2_Immune_PCA_18006808 LY9

HER2_Immune_PCA_18006808 CD180

HER2_Immune_PCA_18006808 LY75

HER2_Immune_PCA_18006808 LYL1

HER2_Immune_PCA_18006808 LYN

HER2_Immune_PCA_18006808 SH2D1A

HER2_Immune_PCA_18006808 MAGEB4

HER2_Immune_PCA_18006808 MAL

HER2_Immune_PCA_18006808 MAN2B1

HER2_Immune_PCA_18006808 MAP1B

HER2_Immune_PCA_18006808 MAP4

HER2_Immune_PCA_18006808 MATK

HER2_Immune_PCA_18006808 MBP

HER2_Immune_PCA_18006808 MEF2C

HER2_Immune_PCA_18006808 MFNG

HER2_Immune_PCA_18006808 MICB

HER2_Immune_PCA_18006808 CXCL9

HER2_Immune_PCA_18006808 MNDA

HER2_Immune_PCA_18006808 MPP1

HER2_Immune_PCA_18006808 MRC1

HER2_Immune_PCA_18006808 MYO1F

HER2_Immune_PCA_18006808 MTX1

HER2_Immune_PCA_18006808 MYD88

HER2_Immune_PCA_18006808 MYH10

HER2_Immune_PCA_18006808 MYO1D

HER2_Immune_PCA_18006808 MYO6

HER2_Immune_PCA_18006808 MYO7A

HER2_Immune_PCA_18006808 NUBP1

HER2_Immune_PCA_18006808 NELL2

HER2_Immune_PCA_18006808 NF1

HER2_Immune_PCA_18006808 NFATC3

HER2_Immune_PCA_18006808 NFKBIA

HER2_Immune_PCA_18006808 NFKBIE

HER2_Immune_PCA_18006808 NKG7

HER2_Immune_PCA_18006808 NPAT

HER2_Immune_PCA_18006808 OAZ1

HER2_Immune_PCA_18006808 OPHN1

HER2_Immune_PCA_18006808 P2RX5

HER2_Immune_PCA_18006808 P2RX7

HER2_Immune_PCA_18006808 PARN

HER2_Immune_PCA_18006808 PAX1

HER2_Immune_PCA_18006808 PAX5

HER2_Immune_PCA_18006808 PFKFB4

HER2_Immune_PCA_18006808 PGM3

HER2_Immune_PCA_18006808 ABCB1

HER2_Immune_PCA_18006808 PHKA1

HER2_Immune_PCA_18006808 SERPINB9

HER2_Immune_PCA_18006808 PIM1

HER2_Immune_PCA_18006808 PIK3CD

HER2_Immune_PCA_18006808 PIP4K2A

HER2_Immune_PCA_18006808 PKP2

HER2_Immune_PCA_18006808 PLCG2

HER2_Immune_PCA_18006808 PLEK

HER2_Immune_PCA_18006808 PLOD2

HER2_Immune_PCA_18006808 PLSCR1

HER2_Immune_PCA_18006808 PNOC

HER2_Immune_PCA_18006808 PNLIPRP1

HER2_Immune_PCA_18006808 POU2AF1

HER2_Immune_PCA_18006808 POU2F1

HER2_Immune_PCA_18006808 PPIC

HER2_Immune_PCA_18006808 PPP1CB

HER2_Immune_PCA_18006808 PPP3CC

HER2_Immune_PCA_18006808 PRCP

HER2_Immune_PCA_18006808 PRF1

HER2_Immune_PCA_18006808 PRKCB

HER2_Immune_PCA_18006808 PRKCQ

HER2_Immune_PCA_18006808 MAPK6

HER2_Immune_PCA_18006808 MAPK9

HER2_Immune_PCA_18006808 PSAP

HER2_Immune_PCA_18006808 PSMB8

HER2_Immune_PCA_18006808 PSMB9

HER2_Immune_PCA_18006808 PSMB10

HER2_Immune_PCA_18006808 PTAFR

HER2_Immune_PCA_18006808 PTGDS

HER2_Immune_PCA_18006808 PTGER4

HER2_Immune_PCA_18006808 PTPN1

HER2_Immune_PCA_18006808 PTPN6

HER2_Immune_PCA_18006808 PTPN7

HER2_Immune_PCA_18006808 PTPN12

HER2_Immune_PCA_18006808 PTPRC

HER2_Immune_PCA_18006808 PTPRCAP

HER2_Immune_PCA_18006808 PTPRF

HER2_Immune_PCA_18006808 ABCD4

HER2_Immune_PCA_18006808 PYCR1

HER2_Immune_PCA_18006808 PYGB

HER2_Immune_PCA_18006808 RAB2A

HER2_Immune_PCA_18006808 RAB3IL1

HER2_Immune_PCA_18006808 RAC1

HER2_Immune_PCA_18006808 RAC2

HER2_Immune_PCA_18006808 RARRES3

HER2_Immune_PCA_18006808 RASA2

HER2_Immune_PCA_18006808 RELB

HER2_Immune_PCA_18006808 RFX5

HER2_Immune_PCA_18006808 RGS10

HER2_Immune_PCA_18006808 RGS13

HER2_Immune_PCA_18006808 RNASE6

HER2_Immune_PCA_18006808 RPL23A

HER2_Immune_PCA_18006808 RPL27A

HER2_Immune_PCA_18006808 RPLP2

HER2_Immune_PCA_18006808 RPS6KA3

HER2_Immune_PCA_18006808 RPS28

HER2_Immune_PCA_18006808 RTN2

HER2_Immune_PCA_18006808 MSMO1

HER2_Immune_PCA_18006808 SCP2

HER2_Immune_PCA_18006808 CCL2

HER2_Immune_PCA_18006808 CCL5

HER2_Immune_PCA_18006808 CCL8

HER2_Immune_PCA_18006808 CCL13

HER2_Immune_PCA_18006808 CCL18

HER2_Immune_PCA_18006808 CCL19

HER2_Immune_PCA_18006808 CCL23

HER2_Immune_PCA_18006808 CXCL11

HER2_Immune_PCA_18006808 XCL1

HER2_Immune_PCA_18006808 SDC2

HER2_Immune_PCA_18006808 SELL

HER2_Immune_PCA_18006808 SELP

HER2_Immune_PCA_18006808 SELPLG

HER2_Immune_PCA_18006808 SLA

HER2_Immune_PCA_18006808 SLAMF1

HER2_Immune_PCA_18006808 SLC1A3

HER2_Immune_PCA_18006808 SLC5A5

HER2_Immune_PCA_18006808 SNRPC

HER2_Immune_PCA_18006808 SOD2

HER2_Immune_PCA_18006808 SOX4

HER2_Immune_PCA_18006808 SP4

HER2_Immune_PCA_18006808 SPI1

HER2_Immune_PCA_18006808 SPIB

HER2_Immune_PCA_18006808 SPP1

HER2_Immune_PCA_18006808 SPRR1A

HER2_Immune_PCA_18006808 TRIM21

HER2_Immune_PCA_18006808 STAT1

HER2_Immune_PCA_18006808 STAT4

HER2_Immune_PCA_18006808 STAT5A

HER2_Immune_PCA_18006808 STK10

HER2_Immune_PCA_18006808 SYK

HER2_Immune_PCA_18006808 TAP1

HER2_Immune_PCA_18006808 TAP2

HER2_Immune_PCA_18006808 TBXA2R

HER2_Immune_PCA_18006808 TCF7

HER2_Immune_PCA_18006808 TCN2

HER2_Immune_PCA_18006808 TRA

HER2_Immune_PCA_18006808 DYNLT3

HER2_Immune_PCA_18006808 TGFB1I1

HER2_Immune_PCA_18006808 THBS1

HER2_Immune_PCA_18006808 THBS2

HER2_Immune_PCA_18006808 TIAM1

HER2_Immune_PCA_18006808 TIMP3

HER2_Immune_PCA_18006808 TJP1

HER2_Immune_PCA_18006808 TLR1

HER2_Immune_PCA_18006808 TLR2

HER2_Immune_PCA_18006808 TNF

HER2_Immune_PCA_18006808 TNNC2

HER2_Immune_PCA_18006808 TNFAIP3

HER2_Immune_PCA_18006808 TNFRSF1B

HER2_Immune_PCA_18006808 TPM1

HER2_Immune_PCA_18006808 TPMT

HER2_Immune_PCA_18006808 NR2C2

HER2_Immune_PCA_18006808 TRAF1

HER2_Immune_PCA_18006808 PHLDA2

HER2_Immune_PCA_18006808 TTC3

HER2_Immune_PCA_18006808 TYROBP

HER2_Immune_PCA_18006808 UBA7

HER2_Immune_PCA_18006808 VAV1

HER2_Immune_PCA_18006808 VCAM1

HER2_Immune_PCA_18006808 VCL

HER2_Immune_PCA_18006808 VEGFA

HER2_Immune_PCA_18006808 VLDLR

HER2_Immune_PCA_18006808 WARS

HER2_Immune_PCA_18006808 WAS

HER2_Immune_PCA_18006808 WIPF1

HER2_Immune_PCA_18006808 LAT2

HER2_Immune_PCA_18006808 XPNPEP2

HER2_Immune_PCA_18006808 ZAP70

HER2_Immune_PCA_18006808 TRIM25

HER2_Immune_PCA_18006808 LAPTM5

HER2_Immune_PCA_18006808 PXDN

HER2_Immune_PCA_18006808 CXCR4

HER2_Immune_PCA_18006808 LST1

HER2_Immune_PCA_18006808 NUP214

HER2_Immune_PCA_18006808 PDHX

HER2_Immune_PCA_18006808 TCL1A

HER2_Immune_PCA_18006808 ELL

HER2_Immune_PCA_18006808 GTPBP6

HER2_Immune_PCA_18006808 USP9X

HER2_Immune_PCA_18006808 HIST1H2BO

HER2_Immune_PCA_18006808 SNHG3

HER2_Immune_PCA_18006808 RECK

HER2_Immune_PCA_18006808 SOAT2

HER2_Immune_PCA_18006808 DHX16

HER2_Immune_PCA_18006808 GPR65

HER2_Immune_PCA_18006808 MAP4K3

HER2_Immune_PCA_18006808 CNTNAP1

HER2_Immune_PCA_18006808 NDST2

HER2_Immune_PCA_18006808 KCNAB2

HER2_Immune_PCA_18006808 GAS7

HER2_Immune_PCA_18006808 CST7

HER2_Immune_PCA_18006808 CAMK1

HER2_Immune_PCA_18006808 APOL1

HER2_Immune_PCA_18006808 BHLHE40

HER2_Immune_PCA_18006808 PDLIM4

HER2_Immune_PCA_18006808 SKAP1

HER2_Immune_PCA_18006808 ABCB11

HER2_Immune_PCA_18006808 S1PR4

HER2_Immune_PCA_18006808 TNFRSF25

HER2_Immune_PCA_18006808 EED

HER2_Immune_PCA_18006808 TNFSF14

HER2_Immune_PCA_18006808 TNFRSF14

HER2_Immune_PCA_18006808 RAB11A

HER2_Immune_PCA_18006808 TNFRSF6B

HER2_Immune_PCA_18006808 IL18RAP

HER2_Immune_PCA_18006808 IL18R1

HER2_Immune_PCA_18006808 CD84

HER2_Immune_PCA_18006808 KAT2B

HER2_Immune_PCA_18006808 VNN2

HER2_Immune_PCA_18006808 FUBP3

HER2_Immune_PCA_18006808 P4HA2

HER2_Immune_PCA_18006808 F2RL3

HER2_Immune_PCA_18006808 MAP3K14

HER2_Immune_PCA_18006808 BRSK2

HER2_Immune_PCA_18006808 TM4SF5

HER2_Immune_PCA_18006808 DOK2

HER2_Immune_PCA_18006808 SH2D2A

HER2_Immune_PCA_18006808 PSTPIP1

HER2_Immune_PCA_18006808 SLC7A7

HER2_Immune_PCA_18006808 FCGR2C

HER2_Immune_PCA_18006808 NMI

HER2_Immune_PCA_18006808 ARHGEF1

HER2_Immune_PCA_18006808 IL32

HER2_Immune_PCA_18006808 UBE2L6

HER2_Immune_PCA_18006808 STK17A

HER2_Immune_PCA_18006808 CD83

HER2_Immune_PCA_18006808 CD163

HER2_Immune_PCA_18006808 SLC22A6

HER2_Immune_PCA_18006808 LPXN

HER2_Immune_PCA_18006808 CHST2

HER2_Immune_PCA_18006808 AIM2

HER2_Immune_PCA_18006808 MAP4K4

HER2_Immune_PCA_18006808 ITM2A

HER2_Immune_PCA_18006808 HOMER1

HER2_Immune_PCA_18006808 ARHGEF6

HER2_Immune_PCA_18006808 IL27RA

HER2_Immune_PCA_18006808 PCYT1B

HER2_Immune_PCA_18006808 THEMIS2

HER2_Immune_PCA_18006808 MAGED1

HER2_Immune_PCA_18006808 GMFG

HER2_Immune_PCA_18006808 PREPL

HER2_Immune_PCA_18006808 CYTIP

HER2_Immune_PCA_18006808 NFE2L3

HER2_Immune_PCA_18006808 ZNF592

HER2_Immune_PCA_18006808 IKBKE

HER2_Immune_PCA_18006808 MICAL2

HER2_Immune_PCA_18006808 RALGPS1

HER2_Immune_PCA_18006808 FAM53B

HER2_Immune_PCA_18006808 VGLL4

HER2_Immune_PCA_18006808 HERPUD1

HER2_Immune_PCA_18006808 ACAP1

HER2_Immune_PCA_18006808 RASSF2

HER2_Immune_PCA_18006808 SPOCK2

HER2_Immune_PCA_18006808 TESPA1

HER2_Immune_PCA_18006808 HEPH

HER2_Immune_PCA_18006808 ELMO1

HER2_Immune_PCA_18006808 TRANK1

HER2_Immune_PCA_18006808 NUAK1

HER2_Immune_PCA_18006808 LPGAT1

HER2_Immune_PCA_18006808 P2RY14

HER2_Immune_PCA_18006808 ARHGAP25

HER2_Immune_PCA_18006808 GOLGA5

HER2_Immune_PCA_18006808 NR1H3

HER2_Immune_PCA_18006808 TSPAN32

HER2_Immune_PCA_18006808 ARL4A

HER2_Immune_PCA_18006808 EBI3

HER2_Immune_PCA_18006808 CTDSPL

HER2_Immune_PCA_18006808 KLRG1

HER2_Immune_PCA_18006808 CD96

HER2_Immune_PCA_18006808 RASGRP2

HER2_Immune_PCA_18006808 IGSF6

HER2_Immune_PCA_18006808 ZMPSTE24

HER2_Immune_PCA_18006808 AKAP8

HER2_Immune_PCA_18006808 LILRB2

HER2_Immune_PCA_18006808 NMUR1

HER2_Immune_PCA_18006808 IKZF1

HER2_Immune_PCA_18006808 TRIM22

HER2_Immune_PCA_18006808 BTN3A3

HER2_Immune_PCA_18006808 BTN2A2

HER2_Immune_PCA_18006808 SCML2

HER2_Immune_PCA_18006808 ATP8A1

HER2_Immune_PCA_18006808 CDIPT

HER2_Immune_PCA_18006808 IFI30

HER2_Immune_PCA_18006808 TACC3

HER2_Immune_PCA_18006808 CLEC10A

HER2_Immune_PCA_18006808 SEMA4D

HER2_Immune_PCA_18006808 UBD

HER2_Immune_PCA_18006808 BATF

HER2_Immune_PCA_18006808 CXCL13

HER2_Immune_PCA_18006808 GNLY

HER2_Immune_PCA_18006808 SORBS1

HER2_Immune_PCA_18006808 IFITM2

HER2_Immune_PCA_18006808 PDLIM5

HER2_Immune_PCA_18006808 TXNIP

HER2_Immune_PCA_18006808 CELF2

HER2_Immune_PCA_18006808 CXCR6

HER2_Immune_PCA_18006808 VAMP5

HER2_Immune_PCA_18006808 LILRB1

HER2_Immune_PCA_18006808 ADAM28

HER2_Immune_PCA_18006808 ARID5A

HER2_Immune_PCA_18006808 FGL2

HER2_Immune_PCA_18006808 BTNL3

HER2_Immune_PCA_18006808 COPS8

HER2_Immune_PCA_18006808 MSL3

HER2_Immune_PCA_18006808 IFI44L

HER2_Immune_PCA_18006808 FERMT2

HER2_Immune_PCA_18006808 LILRB4

HER2_Immune_PCA_18006808 KDELR3

HER2_Immune_PCA_18006808 LILRA2

HER2_Immune_PCA_18006808 RAB31

HER2_Immune_PCA_18006808 PIM2

HER2_Immune_PCA_18006808 ESM1

HER2_Immune_PCA_18006808 ADAM30

HER2_Immune_PCA_18006808 BTN3A2

HER2_Immune_PCA_18006808 BTN3A1

HER2_Immune_PCA_18006808 BTN2A1

HER2_Immune_PCA_18006808 CD160

HER2_Immune_PCA_18006808 CORO1A

HER2_Immune_PCA_18006808 MAP4K1

HER2_Immune_PCA_18006808 SEC63

HER2_Immune_PCA_18006808 SP140

HER2_Immune_PCA_18006808 TREX1

HER2_Immune_PCA_18006808 SLCO2B1

HER2_Immune_PCA_18006808 TFEC

HER2_Immune_PCA_18006808 NTNG1

HER2_Immune_PCA_18006808 NLRP1

HER2_Immune_PCA_18006808 SEC31A

HER2_Immune_PCA_18006808 CARD8

HER2_Immune_PCA_18006808 KLRK1

HER2_Immune_PCA_18006808 PDCD11

HER2_Immune_PCA_18006808 MAST3

HER2_Immune_PCA_18006808 KIF21B

HER2_Immune_PCA_18006808 FNBP1

HER2_Immune_PCA_18006808 SETX

HER2_Immune_PCA_18006808 CMTR1

HER2_Immune_PCA_18006808 PASK

HER2_Immune_PCA_18006808 RGL1

HER2_Immune_PCA_18006808 RFTN1

HER2_Immune_PCA_18006808 CYFIP1

HER2_Immune_PCA_18006808 SYT11

HER2_Immune_PCA_18006808 FBXO28

HER2_Immune_PCA_18006808 NUP210

HER2_Immune_PCA_18006808 PLCL2

HER2_Immune_PCA_18006808 SEL1L3

HER2_Immune_PCA_18006808 CAMSAP2

HER2_Immune_PCA_18006808 WWC1

HER2_Immune_PCA_18006808 EHBP1

HER2_Immune_PCA_18006808 SYNE1

HER2_Immune_PCA_18006808 SUN1

HER2_Immune_PCA_18006808 KIAA0895

HER2_Immune_PCA_18006808 ARHGEF18

HER2_Immune_PCA_18006808 PPWD1

HER2_Immune_PCA_18006808 COTL1

HER2_Immune_PCA_18006808 ISCU

HER2_Immune_PCA_18006808 CBX7

HER2_Immune_PCA_18006808 TNFRSF13B

HER2_Immune_PCA_18006808 ZFYVE26

HER2_Immune_PCA_18006808 LILRA4

HER2_Immune_PCA_18006808 PRG1

HER2_Immune_PCA_18006808 HEBP2

HER2_Immune_PCA_18006808 SH3BP4

HER2_Immune_PCA_18006808 MAFF

HER2_Immune_PCA_18006808 APOL2

HER2_Immune_PCA_18006808 TNFAIP8

HER2_Immune_PCA_18006808 PRKD2

HER2_Immune_PCA_18006808 IFFO1

HER2_Immune_PCA_18006808 SAMHD1

HER2_Immune_PCA_18006808 KBTBD2

HER2_Immune_PCA_18006808 MOXD1

HER2_Immune_PCA_18006808 IPCEF1

HER2_Immune_PCA_18006808 GLCE

HER2_Immune_PCA_18006808 PPP1R16B

HER2_Immune_PCA_18006808 CCDC9

HER2_Immune_PCA_18006808 FBXW2

HER2_Immune_PCA_18006808 PTPN22

HER2_Immune_PCA_18006808 STAP1

HER2_Immune_PCA_18006808 FBXO3

HER2_Immune_PCA_18006808 PLA2G2D

HER2_Immune_PCA_18006808 GALNT8

HER2_Immune_PCA_18006808 CYFIP2

HER2_Immune_PCA_18006808 LAT

HER2_Immune_PCA_18006808 LAMP3

HER2_Immune_PCA_18006808 TSPAN13

HER2_Immune_PCA_18006808 DKK3

HER2_Immune_PCA_18006808 CYTH4

HER2_Immune_PCA_18006808 DISC1

HER2_Immune_PCA_18006808 GPR162

HER2_Immune_PCA_18006808 SIT1

HER2_Immune_PCA_18006808 ADAMDEC1

HER2_Immune_PCA_18006808 P2RY10

HER2_Immune_PCA_18006808 APOBEC3C

HER2_Immune_PCA_18006808 MAT2B

HER2_Immune_PCA_18006808 SLCO3A1

HER2_Immune_PCA_18006808 IGKV1-5

HER2_Immune_PCA_18006808 IGLV2-14

HER2_Immune_PCA_18006808 IGLJ3

HER2_Immune_PCA_18006808 IGKV1D-13

HER2_Immune_PCA_18006808 COA3

HER2_Immune_PCA_18006808 TMEM176B

HER2_Immune_PCA_18006808 CTNNA3

HER2_Immune_PCA_18006808 CLEC2D

HER2_Immune_PCA_18006808 C11orf21

HER2_Immune_PCA_18006808 ICOS

HER2_Immune_PCA_18006808 SNX15

HER2_Immune_PCA_18006808 GPR171

HER2_Immune_PCA_18006808 PILRA

HER2_Immune_PCA_18006808 TBX21

HER2_Immune_PCA_18006808 CD209

HER2_Immune_PCA_18006808 TAX1BP3

HER2_Immune_PCA_18006808 PODXL2

HER2_Immune_PCA_18006808 IL21R

HER2_Immune_PCA_18006808 DEF6

HER2_Immune_PCA_18006808 CUZD1

HER2_Immune_PCA_18006808 TRAT1

HER2_Immune_PCA_18006808 C6orf48

HER2_Immune_PCA_18006808 CLEC4A

HER2_Immune_PCA_18006808 NTM

HER2_Immune_PCA_18006808 DESI2

HER2_Immune_PCA_18006808 UBXN1

HER2_Immune_PCA_18006808 LAP3

HER2_Immune_PCA_18006808 ANGPTL4

HER2_Immune_PCA_18006808 INSIG2

HER2_Immune_PCA_18006808 LEF1

HER2_Immune_PCA_18006808 PLEKHO1

HER2_Immune_PCA_18006808 HERC5

HER2_Immune_PCA_18006808 BET1L

HER2_Immune_PCA_18006808 TLR7

HER2_Immune_PCA_18006808 COA4

HER2_Immune_PCA_18006808 GMIP

HER2_Immune_PCA_18006808 PLAC8

HER2_Immune_PCA_18006808 MS4A4A

HER2_Immune_PCA_18006808 PLA1A

HER2_Immune_PCA_18006808 ZNF589

HER2_Immune_PCA_18006808 TRPV2

HER2_Immune_PCA_18006808 NOL7

HER2_Immune_PCA_18006808 BIN2

HER2_Immune_PCA_18006808 SUCO

HER2_Immune_PCA_18006808 CHMP3

HER2_Immune_PCA_18006808 ACSL5

HER2_Immune_PCA_18006808 UIMC1

HER2_Immune_PCA_18006808 CD244

HER2_Immune_PCA_18006808 TUBA8

HER2_Immune_PCA_18006808 NUDT9

HER2_Immune_PCA_18006808 UBASH3A

HER2_Immune_PCA_18006808 S1PR5

HER2_Immune_PCA_18006808 C21orf91

HER2_Immune_PCA_18006808 TREM1

HER2_Immune_PCA_18006808 SH3TC1

HER2_Immune_PCA_18006808 CPVL

HER2_Immune_PCA_18006808 RHOF

HER2_Immune_PCA_18006808 EPB41L4B

HER2_Immune_PCA_18006808 TMEM106B

HER2_Immune_PCA_18006808 LEPROT

HER2_Immune_PCA_18006808 ASPN

HER2_Immune_PCA_18006808 LAX1

HER2_Immune_PCA_18006808 TXNL4B

HER2_Immune_PCA_18006808 HCFC1R1

HER2_Immune_PCA_18006808 BANK1

HER2_Immune_PCA_18006808 TAPBPL

HER2_Immune_PCA_18006808 PLEKHJ1

HER2_Immune_PCA_18006808 ZNF532

HER2_Immune_PCA_18006808 TMEM140

HER2_Immune_PCA_18006808 GIMAP4

HER2_Immune_PCA_18006808 SLC29A3

HER2_Immune_PCA_18006808 C19orf66

HER2_Immune_PCA_18006808 GIMAP5

HER2_Immune_PCA_18006808 PI4K2A

HER2_Immune_PCA_18006808 TMEM176A

HER2_Immune_PCA_18006808 DNAH3

HER2_Immune_PCA_18006808 GALNT10

HER2_Immune_PCA_18006808 DOCK10

HER2_Immune_PCA_18006808 AMBRA1

HER2_Immune_PCA_18006808 BCAS4

HER2_Immune_PCA_18006808 IARS2

HER2_Immune_PCA_18006808 NECAP2

HER2_Immune_PCA_18006808 DHX32

HER2_Immune_PCA_18006808 WWC3

HER2_Immune_PCA_18006808 ARHGAP15

HER2_Immune_PCA_18006808 BIN3

HER2_Immune_PCA_18006808 MOSPD1

HER2_Immune_PCA_18006808 CRTAM

HER2_Immune_PCA_18006808 SLC2A9

HER2_Immune_PCA_18006808 ASCL3

HER2_Immune_PCA_18006808 SLAMF8

HER2_Immune_PCA_18006808 POGLUT1

HER2_Immune_PCA_18006808 PGLYRP4

HER2_Immune_PCA_18006808 ATP13A1

HER2_Immune_PCA_18006808 RTN4

HER2_Immune_PCA_18006808 GRAMD1B

HER2_Immune_PCA_18006808 MTUS1

HER2_Immune_PCA_18006808 BAHCC1

HER2_Immune_PCA_18006808 WDR48

HER2_Immune_PCA_18006808 SLAMF7

HER2_Immune_PCA_18006808 C6orf47

HER2_Immune_PCA_18006808 CADM3

HER2_Immune_PCA_18006808 CELA2A

HER2_Immune_PCA_18006808 TNN

HER2_Immune_PCA_18006808 GPSM3

HER2_Immune_PCA_18006808 SAMSN1

HER2_Immune_PCA_18006808 RTP4

HER2_Immune_PCA_18006808 DPEP2

HER2_Immune_PCA_18006808 MS4A6A

HER2_Immune_PCA_18006808 CLEC7A

HER2_Immune_PCA_18006808 ACBD3

HER2_Immune_PCA_18006808 PARP12

HER2_Immune_PCA_18006808 BCL11B

HER2_Immune_PCA_18006808 MRPS6

HER2_Immune_PCA_18006808 ZNF747

HER2_Immune_PCA_18006808 PVRIG

HER2_Immune_PCA_18006808 PLEKHF1

HER2_Immune_PCA_18006808 TMEM243

HER2_Immune_PCA_18006808 LILRA6

HER2_Immune_PCA_18006808 ZNF576

HER2_Immune_PCA_18006808 EFHD2

HER2_Immune_PCA_18006808 FCRL2

HER2_Immune_PCA_18006808 ZBED2

HER2_Immune_PCA_18006808 TNFAIP8L2

HER2_Immune_PCA_18006808 PARP8

HER2_Immune_PCA_18006808 ANKRD55

HER2_Immune_PCA_18006808 ARHGAP28

HER2_Immune_PCA_18006808 TREML2

HER2_Immune_PCA_18006808 CPSF7

HER2_Immune_PCA_18006808 PLBD1

HER2_Immune_PCA_18006808 RIN3

HER2_Immune_PCA_18006808 DENND1C

HER2_Immune_PCA_18006808 WLS

HER2_Immune_PCA_18006808 TMEM156

HER2_Immune_PCA_18006808 ATF7IP2

HER2_Immune_PCA_18006808 HEXA-AS1

HER2_Immune_PCA_18006808 MUS81

HER2_Immune_PCA_18006808 RUNX1-IT1

HER2_Immune_PCA_18006808 CXorf21

HER2_Immune_PCA_18006808 MED28

HER2_Immune_PCA_18006808 TRAF3IP3

HER2_Immune_PCA_18006808 THUMPD2

HER2_Immune_PCA_18006808 LIMD2

HER2_Immune_PCA_18006808 APOL5

HER2_Immune_PCA_18006808 APOL3

HER2_Immune_PCA_18006808 NPL

HER2_Immune_PCA_18006808 SLC2A10

HER2_Immune_PCA_18006808 FAM49A

HER2_Immune_PCA_18006808 PLA2G12A

HER2_Immune_PCA_18006808 ITM2C

HER2_Immune_PCA_18006808 RASSF4

HER2_Immune_PCA_18006808 ZDHHC18

HER2_Immune_PCA_18006808 GLYR1

HER2_Immune_PCA_18006808 CBR4

HER2_Immune_PCA_18006808 GUSBP11

HER2_Immune_PCA_18006808 SP140L

HER2_Immune_PCA_18006808 EGLN3

HER2_Immune_PCA_18006808 TOE1

HER2_Immune_PCA_18006808 EFHC1

HER2_Immune_PCA_18006808 OSBPL9

HER2_Immune_PCA_18006808 MBOAT2

HER2_Immune_PCA_18006808 RAB40A

HER2_Immune_PCA_18006808 A2M-AS1

HER2_Immune_PCA_18006808 PYHIN1

HER2_Immune_PCA_18006808 DSTNP2

HER2_Immune_PCA_18006808 PAOX

HER2_Immune_PCA_18006808 U2AF1L4

HER2_Immune_PCA_18006808 APOBEC3A

HER2_Immune_PCA_18006808 APOBEC3F

HER2_Immune_PCA_18006808 FGD2

HER2_Immune_PCA_18006808 CCZ1B

HER2_Immune_PCA_18006808 NCR3

HER2_Immune_PCA_18006808 SIRPB2

HER2_Immune_PCA_18006808 IGKV1OR2-118

HER2_Immune_PCA_18006808 GVINP1

HER2_Immune_PCA_18006808 LOC391020

HER2_Immune_PCA_18006808 MXRA7

HER2_Immune_PCA_18006808 CCDC88C

HER2_Immune_PCA_18006808 IRS3P

HER2_Immune_PCA_18006808 GIMAP6

HER2_Immune_PCA_18006808 NCF1

HER2_Immune_PCA_18006808 LOC728392

HER2_Immune_PCA_18006808 43349

HER2_Immune_PCA_18006808 BABAM2

HER2_Immune_PCA_18006808 DGLUCY

HER2_Immune_PCA_18006808 ADGRE5

HER2_Immune_PCA_18006808 ADA2

HER2_Immune_PCA_18006808 CGB2

HER2_Immune_PCA_18006808 FCMR

HER2_Immune_PCA_18006808 OTULINL

HER2_Immune_PCA_18006808 RTL8C

HER2_Immune_PCA_18006808 WASHC2A

HER2_Immune_PCA_18006808 RIPOR2

HER2_Immune_PCA_18006808 MRM2

HER2_Immune_PCA_18006808 FYB1

HER2_Immune_PCA_18006808 NOP53

HER2_Immune_PCA_18006808 ADGRF5

HER2_Immune_PCA_18006808 GUCY1B1

HER2_Immune_PCA_18006808 ARHGAP45

HER2_Immune_PCA_18006808 JCHAIN

HER2_Immune_PCA_18006808 FAM30A

HER2_Immune_PCA_18006808 RUBCN

HER2_Immune_PCA_18006808 RUBCNL

HER2_Immune_PCA_18006808 KIAA1551

HER2_Immune_PCA_18006808 MTERF4

HER2_Immune_PCA_18006808 CIAO3

HER2_Immune_PCA_18006808 JADE2

HER2_Immune_PCA_18006808 SELENOP

HER2_Immune_PCA_18006808 TCEAL9

ICR_ACT_SCORE IFNG

ICR_ACT_SCORE IRF1

ICR_ACT_SCORE STAT1

ICR_ACT_SCORE IL12B

ICR_ACT_SCORE TBX21

ICR_ACT_SCORE GZMB

ICR_ACT_SCORE GNLY

ICR_ACT_SCORE PRF1

ICR_ACT_SCORE GZMH

ICR_ACT_SCORE GZMA

ICR_ACT_SCORE CXCL10

ICR_ACT_SCORE CXCL9

ICR_ACT_SCORE CCL5

ICR_ACT_SCORE CD8A

ICR_ACT_SCORE CD8B

ICR_ACT_SCORE PDCD1

ICR_INHIB_SCORE CTLA4

ICR_INHIB_SCORE FOXP3

ICR_INHIB_SCORE IDO1

ICR_INHIB_SCORE CD274

ICR_SCORE IFNG

ICR_SCORE IRF1

ICR_SCORE STAT1

ICR_SCORE IL12B

ICR_SCORE TBX21

ICR_SCORE GZMB

ICR_SCORE GNLY

ICR_SCORE PRF1

ICR_SCORE GZMH

ICR_SCORE GZMA

ICR_SCORE CXCL10

ICR_SCORE CXCL9

ICR_SCORE CCL5

ICR_SCORE CD8A

ICR_SCORE CD8B

ICR_SCORE CTLA4

ICR_SCORE PDCD1

ICR_SCORE FOXP3

ICR_SCORE IDO1

ICR_SCORE CD274

ICS5_score CXCL13

ICS5_score CLIC5

ICS5_score HLA-F

ICS5_score TNFRSF17

ICS5_score XCL2

IFIT3 IFIT3

IFIT3 IFI44L

IFIT3 IFIT1

IFIT3 RSAD2

IFIT3 MX1

IFIT3 IFI44

IFIT3 OAS2

IFIT3 CMPK2

IFIT3 IFI6

IFIT3 IFIT2

IFN_21978456 IFI27

IFN_21978456 MX1

IFN_21978456 ISG15

IFN_21978456 IFIT1

IFN_21978456 IFIT3

IFN_21978456 RSAD2

IFN_21978456 IFI44L

IFN_21978456 IFI44

IFN_21978456 OAS1

IFN_21978456 OAS2

IFN_21978456 OAS3

IFN_21978456 HERC6

IFN_21978456 OASL

IFNG_score_21050467 MAP3K10

IFNG_score_21050467 CSRP3

IFNG_score_21050467 FOSL1

IFNG_score_21050467 HLA-B

IFNG_score_21050467 SF3A1

IFNG_score_21050467 XRCC6

IFNG_score_21050467 CEBPD

IFNG_score_21050467 HLA-A

IFNG_score_21050467 NMI

IFNG_score_21050467 IFI35

IFNG_score_21050467 HIF3A

IFNG_score_21050467 TAS2R5

IFNG_score_21050467 C1S

IFNG_score_21050467 COL16A1

IFNG_score_21050467 PSME1

IFNG_score_21050467 IFITM1

IFNG_score_21050467 CYCS

IFNG_score_21050467 BAK1

IFNG_score_21050467 EPS15

IFNG_score_21050467 GBP1

IFNG_score_21050467 FAS

IFNG_score_21050467 PML

IFNG_score_21050467 HADH

IFNG_score_21050467 EIF2B1

IFNG_score_21050467 HLA-E

IFNG_score_21050467 HADHB

IFNG_score_21050467 IRF1

IFNG_score_21050467 ADAR

IFNG_score_21050467 IFIT3

IFNG_score_21050467 SDCBP

IFNG_score_21050467 ATP6V0B

IFNG_score_21050467 ZFP36L2

IFNG_score_21050467 TRIM21

IFNG_score_21050467 PRAME

IFNG_score_21050467 ELK4

IFNG_score_21050467 BBC3

IFNG_score_21050467 PSMB10

IFNG_score_21050467 CASP8

IFNG_score_21050467 PHLDA1

IFNG_score_21050467 PPP3CA

IFNG_score_21050467 BTG1

IFNG_score_21050467 RBBP4

IFNG_score_21050467 PMAIP1

IFNG_score_21050467 TEAD4

IFNG_score_21050467 SRP9

IFNG_score_21050467 ISG15

IFNG_score_21050467 BST2

IFNG_score_21050467 PYHIN1

IFNG_score_21050467 IL15RA

IFNG_score_21050467 VEGFC

IFNG_score_21050467 RHOC

IFNG_score_21050467 PLOD2

IFNG_score_21050467 STAT1

IFNG_score_21050467 PSMB8

IFNG_score_21050467 VAT1

IFNG_score_21050467 PARP1

IFNG_score_21050467 ICAM1

IFNG_score_21050467 TAP1

IFNG_score_21050467 IL6

IFNG_score_21050467 BAG1

IFNG_score_21050467 SRSF2

IFNG_score_21050467 SEM1

IFNG_score_21050467 SKP1

IgG_19272155 IGKV1OR2-118

IgG_19272155 IGKV1D-13

IgG_19272155 IGLL3P

IgG_19272155 IGKV1-5

IgG_19272155 IGHV1-69

IgG_19272155 POU2AF1

IgG_19272155 IGLJ3

IgG_19272155 GUSBP11

IgG_19272155 IGLC2

IgG_19272155 IGLV3-25

IgG_19272155 IGKC

IgG_19272155 IGLV2-14

IgG_19272155 IGHG1

IgG_19272155 IGHM

IgG_19272155 IGLV4-3

IgG_19272155 IGHD

IgG_19272155 IGHA1

IgG_19272155 IGL

IgG_19272155 IGH

IgG_19272155 IGLC1

IgG_19272155 LOC96610

IGG_Cluster_21214954 CD27

IGG_Cluster_21214954 IL2RG

IGG_Cluster_21214954 CD79A

IGG_Cluster_21214954 PIM2

IGG_Cluster_21214954 POU2AF1

IGG_Cluster_21214954 IGKC

IGG_Cluster_21214954 IGLV3-25

IGG_Cluster_21214954 IGL

IGG_Cluster_21214954 TNFRSF17

IGG_Cluster_21214954 NTN3

IGG_Cluster_21214954 HLA-C

IGG_Cluster_21214954 LAX1

IGG_Cluster_21214954 JCHAIN

IGG_Cluster_21214954 CXCL8

IL12_score_21050467 CD3E

IL12_score_21050467 IL12B

IL12_score_21050467 STAT4

IL12_score_21050467 IFNG

IL12_score_21050467 JAK2

IL12_score_21050467 IL12RB1

IL12_score_21050467 IL12RB2

IL12_score_21050467 MAPK14

IL12_score_21050467 IL12A

IL12_score_21050467 MAP2K6

IL12_score_21050467 TYK2

IL12_score_21050467 JUN

IL12_score_21050467 IL18

IL12_score_21050467 CXCR3

IL12_score_21050467 CD3G

IL12_score_21050467 ETV5

IL12_score_21050467 CCR5

IL12_score_21050467 CD3D

IL12_score_21050467 MAPK8

IL12_score_21050467 IL18R1

IL12_score_21050467 CD247

IL12_score_21050467 TRA

IL12_score_21050467 TRB

IL13_score_21050467 IL13

IL13_score_21050467 IL13RA2

IL13_score_21050467 JAK2

IL13_score_21050467 IL13RA1

IL13_score_21050467 TYK2

IL13_score_21050467 IL4R

IL13_score_21050467 JAK1

IL2_score_21050467 RAF1

IL2_score_21050467 IL2

IL2_score_21050467 STAT5A

IL2_score_21050467 IL2RB

IL2_score_21050467 SOS1

IL2_score_21050467 GRB2

IL2_score_21050467 STAT5B

IL2_score_21050467 HRAS

IL2_score_21050467 LCK

IL2_score_21050467 JUN

IL2_score_21050467 MAPK3

IL2_score_21050467 IL2RG

IL2_score_21050467 JAK1

IL2_score_21050467 JAK3

IL2_score_21050467 CSNK2A1

IL2_score_21050467 MAP2K1

IL2_score_21050467 FOS

IL2_score_21050467 SHC1

IL2_score_21050467 SYK

IL2_score_21050467 ELK1

IL2_score_21050467 IL2RA

IL2_score_21050467 MAPK8

IL4_score_21050467 JAK3

IL4_score_21050467 GRB2

IL4_score_21050467 STAT6

IL4_score_21050467 SHC1

IL4_score_21050467 IL4

IL4_score_21050467 IL4R

IL4_score_21050467 AKT1

IL4_score_21050467 IRS1

IL4_score_21050467 IL2RG

IL4_score_21050467 JAK1

IL4_score_21050467 RPS6KB1

IL8_21978456 CXCL1

IL8_21978456 CXCL8

IL8_21978456 CXCL2

Immune_cell_Cluster_21214954 WARS

Immune_cell_Cluster_21214954 IDO1

Immune_cell_Cluster_21214954 RNASE6

Immune_cell_Cluster_21214954 GPR65

Immune_cell_Cluster_21214954 MNDA

Immune_cell_Cluster_21214954 LAMP3

Immune_cell_Cluster_21214954 TNFAIP8

Immune_cell_Cluster_21214954 LYN

Immune_cell_Cluster_21214954 TNFAIP3

Immune_cell_Cluster_21214954 PLAC8

Immune_cell_Cluster_21214954 IL7R

Immune_cell_Cluster_21214954 CSF2RB

Immune_cell_Cluster_21214954 IRF1

Immune_cell_Cluster_21214954 TNFRSF1B

Immune_cell_Cluster_21214954 ADAMDEC1

Immune_cell_Cluster_21214954 SLAMF8

Immune_cell_Cluster_21214954 SIRPG

Immune_cell_Cluster_21214954 LTB

Immune_cell_Cluster_21214954 PTPRCAP

Immune_cell_Cluster_21214954 CCR7

Immune_cell_Cluster_21214954 SPOCK2

Immune_cell_Cluster_21214954 PLEK

Immune_cell_Cluster_21214954 CD74

Immune_cell_Cluster_21214954 HLA-DMA

Immune_cell_Cluster_21214954 HLA-DRA

Immune_cell_Cluster_21214954 HLA-DMB

Immune_cell_Cluster_21214954 FCER1G

Immune_cell_Cluster_21214954 TYROBP

Immune_cell_Cluster_21214954 LAPTM5

Immune_cell_Cluster_21214954 ITGB2

Immune_cell_Cluster_21214954 C1QA

Immune_cell_Cluster_21214954 STAT1

Immune_cell_Cluster_21214954 CXCL11

Immune_cell_Cluster_21214954 CXCL10

Immune_cell_Cluster_21214954 GBP1

Immune_cell_Cluster_21214954 TAP1

Immune_cell_Cluster_21214954 PSMB9

Immune_cell_Cluster_21214954 GIMAP4

Immune_cell_Cluster_21214954 SRGN

Immune_cell_Cluster_21214954 FGL2

Immune_cell_Cluster_21214954 HCLS1

Immune_cell_Cluster_21214954 ARHGAP25

Immune_cell_Cluster_21214954 LCK

Immune_cell_Cluster_21214954 EVI2B

Immune_cell_Cluster_21214954 ARHGAP15

Immune_cell_Cluster_21214954 CYTIP

Immune_cell_Cluster_21214954 GZMA

Immune_cell_Cluster_21214954 LCP2

Immune_cell_Cluster_21214954 CD53

Immune_cell_Cluster_21214954 IL10RA

Immune_cell_Cluster_21214954 CD48

Immune_cell_Cluster_21214954 CCL5

Immune_cell_Cluster_21214954 CD3D

Immune_cell_Cluster_21214954 CD2

Immune_cell_Cluster_21214954 GZMK

Immune_cell_Cluster_21214954 PTPRC

Immune_cell_Cluster_21214954 CXCL9

Immune_cell_Cluster_21214954 GMFG

Immune_cell_Cluster_21214954 IRF8

Immune_cell_Cluster_21214954 CTSS

Immune_cell_Cluster_21214954 TFEC

Immune_cell_Cluster_21214954 SLA

Immune_cell_Cluster_21214954 SLC7A7

Immune_cell_Cluster_21214954 LST1

Immune_cell_Cluster_21214954 AIF1

Immune_cell_Cluster_21214954 CD86

Immune_cell_Cluster_21214954 NCKAP1L

Immune_cell_Cluster_21214954 THEMIS2

Immune_cell_Cluster_21214954 LYZ

Immune_cell_Cluster_21214954 CD52

Immune_cell_Cluster_21214954 SELL

Immune_cell_Cluster_21214954 HLA-F

Immune_cell_Cluster_21214954 HLA-B

Immune_cell_Cluster_21214954 HLA-E

Immune_cell_Cluster_21214954 GZMB

Immune_cell_Cluster_21214954 SEMA4D

Immune_cell_Cluster_21214954 BTN3A2

Immune_cell_Cluster_21214954 BTN3A3

Immune_cell_Cluster_21214954 CASP1

Immune_NSCLC_score PLEK

Immune_NSCLC_score RHOH

Immune_NSCLC_score TCEA2

Immune_NSCLC_score CLEC4E

Immune_NSCLC_score USP51

Immune_NSCLC_score WFDC10B

Immune_NSCLC_score SLC4A3

Immune_NSCLC_score CD53

Immune_NSCLC_score SCFV

Immune_NSCLC_score PRDM13

Immune_NSCLC_score OBSL1

Immune_NSCLC_score TAGAP

Immune_NSCLC_score IGLV6-57

Immune_NSCLC_score CD38

Immune_NSCLC_score FKBP9

Immune_NSCLC_score ADAMTSL2

Immune_NSCLC_score CD48

Immune_NSCLC_score GNPTAB

Immune_NSCLC_score CNIH3

Immune_NSCLC_score PSMA6

Immune_NSCLC_score SHROOM1

Immune_NSCLC_score GPSM1

Immune_NSCLC_score TRO

Immune_NSCLC_score GSTT2

Immune_NSCLC_score NQO2

Immune_NSCLC_score EAF2

Immune_NSCLC_score MUC4

Immune_NSCLC_score PABPC1

Immune_NSCLC_score PLA2G7

Immune_NSCLC_score AOAH

Immune_NSCLC_score TMSB4X

Immune_NSCLC_score ACOT8

Immune_NSCLC_score GIMAP7

Immune_NSCLC_score ASAH1

Immune_NSCLC_score TRIM45

Immune_NSCLC_score EXT2

Immune_NSCLC_score IFI6

Immune_NSCLC_score KCNE3

Immune_NSCLC_score CTSF

Immune_NSCLC_score RASL11B

Immune_NSCLC_score HMGCL

Immune_NSCLC_score IGHA1

Immune_NSCLC_score C1QTNF3

Immune_NSCLC_score IL11RA

Immune_NSCLC_score ADRA2C

Immune_NSCLC_score IGKC

Immune_NSCLC_score CEACAM5

Immune_NSCLC_score PURB

Immune_NSCLC_score TPD52

Immune_NSCLC_score SERP2

Immune_NSCLC_score FAM129A

Immune_NSCLC_score TMEM74B

Immune_NSCLC_score ERLEC1

Immune_NSCLC_score FAM198A

Immune_NSCLC_score SNHG15

Immune_NSCLC_score CDK20

Immune_NSCLC_score HSD17B11

Immune_NSCLC_score FLJ21963

Immune_NSCLC_score IGH

Immune_NSCLC_score IGL

Immune_NSCLC_score LOC148898

Immune_NSCLC_score LOC375010

Immune_NSCLC_score LOC388886

Immune_NSCLC_score LOC390712

Immune_NSCLC_score LOC401431

Immune_NSCLC_score LOC642480

Immune_NSCLC_score MGC11271

Immune_NSCLC_score MUM1L1

Immune_NSCLC_score PRKN

Immune_NSCLC_score CYTIP

Immune_NSCLC_score SULT1C2

Immune_NSCLC_score MED13L

Interferon_19272155 RSAD2

Interferon_19272155 OAS3

Interferon_19272155 IFI44

Interferon_19272155 ISG15

Interferon_19272155 DDX60

Interferon_19272155 IFI44L

Interferon_19272155 IFI6

Interferon_19272155 MX1

Interferon_19272155 IFIT3

Interferon_19272155 IFI27

Interferon_19272155 IFIT1

Interferon_19272155 OAS1

Interferon_19272155 OAS2

Interferon_Cluster_21214954 C19orf66

Interferon_Cluster_21214954 BST2

Interferon_Cluster_21214954 SP110

Interferon_Cluster_21214954 PARP12

Interferon_Cluster_21214954 IFIT5

Interferon_Cluster_21214954 OAS1

Interferon_Cluster_21214954 IRF9

Interferon_Cluster_21214954 USP18

Interferon_Cluster_21214954 DDX58

Interferon_Cluster_21214954 MX2

Interferon_Cluster_21214954 IFI27

Interferon_Cluster_21214954 OAS2

Interferon_Cluster_21214954 IFI6

Interferon_Cluster_21214954 IFIT3

Interferon_Cluster_21214954 IFI44L

Interferon_Cluster_21214954 IFI44

Interferon_Cluster_21214954 IFIT1

Interferon_Cluster_21214954 ISG15

Interferon_Cluster_21214954 MX1

Interferon_Cluster_21214954 RSAD2

Interferon_Cluster_21214954 OAS3

Interferon_Cluster_21214954 HERC6

Interferon_Cluster_21214954 HERC5

Interferon_Cluster_21214954 DDX60

Interferon_Cluster_21214954 PLSCR1

Interferon_Cluster_21214954 UBE2L6

Interferon_Cluster_21214954 SAMD9

Interferon_Cluster_21214954 RTP4

Interferon_Cluster_21214954 XAF1

Interferon_Cluster_21214954 NMI

Interferon_Cluster_21214954 IFITM1

Interferon_Cluster_21214954 IFITM3

Interferon_Cluster_21214954 ISG20

Interferon_Cluster_21214954 PSME2

Interferon_Cluster_21214954 PSME1

Interferon_Cluster_21214954 IRF7

IR7_score C1QA

IR7_score IGLC2

IR7_score LY9

IR7_score TNFRSF17

IR7_score SPP1

IR7_score XCL2

IR7_score HLA-F

LCK_19272155 ARHGAP15

LCK_19272155 KLRK1

LCK_19272155 SASH3

LCK_19272155 ARHGAP25

LCK_19272155 SLAMF1

LCK_19272155 GIMAP5

LCK_19272155 SH2D1A

LCK_19272155 CCR7

LCK_19272155 ITK

LCK_19272155 TRBV19

LCK_19272155 RAC2

LCK_19272155 CORO1A

LCK_19272155 PIK3CD

LCK_19272155 LPXN

LCK_19272155 PRKCB

LCK_19272155 STAT4

LCK_19272155 GMFG

LCK_19272155 SELPLG

LCK_19272155 FGL2

LCK_19272155 EVI2B

LCK_19272155 CSF2RB

LCK_19272155 GZMK

LCK_19272155 SAMSN1

LCK_19272155 GIMAP4

LCK_19272155 IRF8

LCK_19272155 NCKAP1L

LCK_19272155 IL10RA

LCK_19272155 TRAC

LCK_19272155 INPP5D

LCK_19272155 PLAC8

LCK_19272155 CCL5

LCK_19272155 CD247

LCK_19272155 LTB

LCK_19272155 IL2RG

LCK_19272155 HCLS1

LCK_19272155 IL7R

LCK_19272155 CD53

LCK_19272155 LCK

LCK_19272155 CD3D

LCK_19272155 CD27

LCK_19272155 GZMA

LCK_19272155 SELL

LCK_19272155 TRBV21-1

LCK_19272155 CD2

LCK_19272155 CD48

LCK_19272155 SLA

LCK_19272155 LCP2

LCK_19272155 SRGN

LCK_19272155 PTPRC

LIexpression_score CCL5

LIexpression_score CD19

LIexpression_score CD37

LIexpression_score CD3D

LIexpression_score CD3E

LIexpression_score CD3G

LIexpression_score CD79A

LIexpression_score CD79B

LIexpression_score CD8A

LIexpression_score IGHG3

LIexpression_score IGLC1

LIexpression_score CD14

LIexpression_score LCK

LIexpression_score LTB

LIexpression_score MS4A1

LIexpression_score CD247

LIexpression_score CD8B

LIexpression_score JCHAIN

LYM CD53

LYM SASH3

LYM IL10RA

LYM NCKAP1L

LYM LCP2

LYM ITGAL

LYM CCR5

LYM CD4

LYM MYO1F

LYM ARHGAP30

LYMPHS_PCA_16704732 UQCRB

LYMPHS_PCA_16704732 SESTD1

LYMPHS_PCA_16704732 QTRT1

LYMPHS_PCA_16704732 TIPIN

LYMPHS_PCA_16704732 REL

LYMPHS_PCA_16704732 STXBP2

LYMPHS_PCA_16704732 HSBP1

LYMPHS_PCA_16704732 COX6C

LYMPHS_PCA_16704732 RPL11

LYMPHS_PCA_16704732 MECOM

LYMPHS_PCA_16704732 ANKRD28

LYMPHS_PCA_16704732 JUN

LYMPHS_PCA_16704732 ZC3H15

LYMPHS_PCA_16704732 RPL23

LYMPHS_PCA_16704732 RPS6KA2

LYMPHS_PCA_16704732 EEF2

LYMPHS_PCA_16704732 TMA7

LYMPHS_PCA_16704732 RPS6

LYMPHS_PCA_16704732 RPL27

LYMPHS_PCA_16704732 RPS21

LYMPHS_PCA_16704732 COX7B

LYMPHS_PCA_16704732 PRRC2B

LYMPHS_PCA_16704732 CYP17A1

LYMPHS_PCA_16704732 NSUN4

LYMPHS_PCA_16704732 TOMM34

LYMPHS_PCA_16704732 MINOS1

LYMPHS_PCA_16704732 STAMBPL1

LYMPHS_PCA_16704732 FGF9

LYMPHS_PCA_16704732 ATF4

LYMPHS_PCA_16704732 RPL35

LYMPHS_PCA_16704732 RPL31

LYMPHS_PCA_16704732 RPS24

LYMPHS_PCA_16704732 DCLRE1C

LYMPHS_PCA_16704732 C5orf49

LYMPHS_PCA_16704732 FAM162A

LYMPHS_PCA_16704732 ITGB2

LYMPHS_PCA_16704732 SLC19A1

LYMPHS_PCA_16704732 RPL32

LYMPHS_PCA_16704732 TPP2

LYMPHS_PCA_16704732 MALAT1

LYMPHS_PCA_16704732 LSM3

LYMPHS_PCA_16704732 ATXN2L

LYMPHS_PCA_16704732 SERPINB6

LYMPHS_PCA_16704732 TPI1

LYMPHS_PCA_16704732 EIPR1

MCD3_CD8_21214954 SRPX

MCD3_CD8_21214954 IGFBP6

MCD3_CD8_21214954 ENPP2

MCD3_CD8_21214954 SEMA3G

MCD3_CD8_21214954 CIDEA

MCD3_CD8_21214954 GPX3

MCD3_CD8_21214954 GPD1

MCD3_CD8_21214954 CD36

MCD3_CD8_21214954 RBP4

MCD3_CD8_21214954 AOC3

MCD3_CD8_21214954 LPL

MCD3_CD8_21214954 FABP4

MCD3_CD8_21214954 ADIPOQ

MCD3_CD8_21214954 PLIN1

MCD3_CD8_21214954 ADH1B

MCD3_CD8_21214954 FHL1

MCD3_CD8_21214954 LEP

MCD3_CD8_21214954 CD34

MCD3_CD8_21214954 SPRY1

MCD3_CD8_21214954 PROS1

MCD3_CD8_21214954 AKAP12

MCD3_CD8_21214954 JAM3

MCD3_CD8_21214954 NDN

MCD3_CD8_21214954 SPARCL1

MCD3_CD8_21214954 ITM2A

MCD3_CD8_21214954 AQP1

MCD3_CD8_21214954 C7

MCD3_CD8_21214954 TSPAN7

MCD3_CD8_21214954 JAM2

MCD3_CD8_21214954 CAV1

MCD3_CD8_21214954 GNG11

MCD3_CD8_21214954 LDB2

MCD3_CD8_21214954 CDH5

MCD3_CD8_21214954 ABCA8

MCD3_CD8_21214954 MEOX1

MCD3_CD8_21214954 VWF

MCD3_CD8_21214954 CLDN5

MCD3_CD8_21214954 ERG

MCD3_CD8_21214954 SLIT2

MCD3_CD8_21214954 PECAM1

MCD3_CD8_21214954 CDO1

MCD3_CD8_21214954 CFD

MCD3_CD8_21214954 ACKR1

MCD3_CD8_21214954 LHFPL6

MCD3_CD8_21214954 PLPP1

MDACC_FNA_1_20805453 TMEM176B

MDACC_FNA_1_20805453 SLCO2B1

MDACC_FNA_1_20805453 CKAP2

MDACC_FNA_1_20805453 CD74

MDACC_FNA_1_20805453 VCAM1

MDACC_FNA_1_20805453 IGLL1

MDACC_FNA_1_20805453 CCL2

MDACC_FNA_1_20805453 GPNMB

MDACC_FNA_1_20805453 PTGDS

MDACC_FNA_1_20805453 CD163

MDACC_FNA_1_20805453 MAFB

MDACC_FNA_1_20805453 HLA-DRB1

MDACC_FNA_1_20805453 PLTP

MDACC_FNA_1_20805453 APOE

MDACC_FNA_1_20805453 C1QB

MDACC_FNA_1_20805453 NTN3

MDACC_FNA_1_20805453 PECAM1

MDACC_FNA_1_20805453 IGKV1D-13

MDACC_FNA_1_20805453 IGKV1OR2-108

MDACC_FNA_1_20805453 IGLJ3

MDACC_FNA_1_20805453 IGKC

MDACC_FNA_1_20805453 IGL

MDACC_FNA_1_20805453 IGHG3

MDACC_FNA_1_20805453 IGHM

MDACC_FNA_1_20805453 HLA-DPA1

MDACC_FNA_1_20805453 HLA-DQB1

MDACC_FNA_1_20805453 HLA-DQA1

MDACC_FNA_1_20805453 IGHG1

MDACC_FNA_1_20805453 C1QA

MDACC_FNA_1_20805453 HLA-DRA

MDACC_FNA_1_20805453 JCHAIN

MHC_I_19272155 HLA-G

MHC_I_19272155 HLA-A

MHC_I_19272155 HLA-B

MHC_I_19272155 HLA-C

MHC_I_19272155 HLA-F

MHC_II_19272155 CTSS

MHC_II_19272155 CD74

MHC_II_19272155 HLA-DRB1

MHC_II_19272155 HLA-DQA1

MHC_II_19272155 HLA-DMB

MHC_II_19272155 HLA-DPA1

MHC_II_19272155 HLA-DPB1

MHC_II_19272155 HLA-DMA

MHC_II_19272155 HLA-DRA

MHC_II_19272155 LCP2

MHC_II_19272155 SRGN

MHC_II_19272155 PTPRC

MHC1_21978456 HLA-E

MHC1_21978456 HLA-G

MHC1_21978456 HLA-B

MHC1_21978456 HLA-C

MHC1_21978456 HLA-J

MHC1_21978456 HLA-F

MHC1_21978456 HLA-A

MHC2_21978456 HLA-DPB1

MHC2_21978456 HLA-DMB

MHC2_21978456 CD74

MHC2_21978456 HLA-DRB5

MHC2_21978456 HLA-DRB1

MHC2_21978456 HLA-DRB4

MHC2_21978456 HLA-DRB3

MHC2_21978456 HLA-DRA

MHC2_21978456 HLA-DPA1

MHC2_21978456 HLA-DQA1

MHC2_21978456 HLA-DQA2

MHC2_21978456 HLA-DMA

MHC2_21978456 HLA-DQB1

MHC2_21978456 LOC100509457

MHC2_21978456 LOC101060835

Minterferon_Cluster_21214954 STAT1

Minterferon_Cluster_21214954 IL18BP

Minterferon_Cluster_21214954 XAF1

Minterferon_Cluster_21214954 B2M

Minterferon_Cluster_21214954 RSAD2

Minterferon_Cluster_21214954 EPSTI1

Minterferon_Cluster_21214954 PSMB8

Minterferon_Cluster_21214954 PSMB9

Minterferon_Cluster_21214954 ZC3HAV1

Minterferon_Cluster_21214954 IFI35

Minterferon_Cluster_21214954 DDX58

Minterferon_Cluster_21214954 GBP7

Minterferon_Cluster_21214954 CXCL10

Minterferon_Cluster_21214954 CCL5

Minterferon_Cluster_21214954 GBP6

Minterferon_Cluster_21214954 CXCL9

Minterferon_Cluster_21214954 CD274

Minterferon_Cluster_21214954 HELZ2

Minterferon_Cluster_21214954 UBA7

Minterferon_Cluster_21214954 LGALS3BP

Minterferon_Cluster_21214954 PARP9

Minterferon_Cluster_21214954 IRF9

Minterferon_Cluster_21214954 ADAR

Minterferon_Cluster_21214954 IFIT2

Minterferon_Cluster_21214954 OASL

Minterferon_Cluster_21214954 DDX60

Minterferon_Cluster_21214954 IFIH1

Minterferon_Cluster_21214954 RTP4

Minterferon_Cluster_21214954 GBP4

Minterferon_Cluster_21214954 IFI44

Minterferon_Cluster_21214954 MX1

Minterferon_Cluster_21214954 ISG15

Minterferon_Cluster_21214954 BST2

Minterferon_Cluster_21214954 USP18

Minterferon_Cluster_21214954 IFIT1

Minterferon_Cluster_21214954 OAS2

Minterferon_Cluster_21214954 ZBP1

Minterferon_Cluster_21214954 PARP14

Minterferon_Cluster_21214954 IRGM

Minterferon_Cluster_21214954 STAT2

Minterferon_Cluster_21214954 PARP12

Minterferon_Cluster_21214954 SP100

Minterferon_Cluster_21214954 GBP1

Minterferon_Cluster_21214954 BATF2

Minterferon_Cluster_21214954 NMI

Minterferon_Cluster_21214954 SLFN13

Minterferon_Cluster_21214954 GVINP1

Minterferon_Cluster_21214954 IRF1

Module11_Prolif_score CDKN3

Module11_Prolif_score NDC80

Module11_Prolif_score RNASEH2A

Module11_Prolif_score CENPA

Module11_Prolif_score SMC2

Module11_Prolif_score CENPE

Module11_Prolif_score RAD51AP1

Module11_Prolif_score PLK4

Module11_Prolif_score NMU

Module11_Prolif_score KIF2C

Module11_Prolif_score TMSB15A

Module11_Prolif_score UBE2C

Module11_Prolif_score CHEK1

Module11_Prolif_score ZWINT

Module11_Prolif_score OIP5

Module11_Prolif_score CRABP1

Module11_Prolif_score ECT2

Module11_Prolif_score EIF4EBP1

Module11_Prolif_score EZH2

Module11_Prolif_score FEN1

Module11_Prolif_score HSPA4L

Module11_Prolif_score TPX2

Module11_Prolif_score FOXM1

Module11_Prolif_score NCAPH

Module11_Prolif_score PRAME

Module11_Prolif_score PDSS1

Module11_Prolif_score KIF4A

Module11_Prolif_score RAD54B

Module11_Prolif_score ASPM

Module11_Prolif_score FBXO5

Module11_Prolif_score ATAD2

Module11_Prolif_score RACGAP1

Module11_Prolif_score GPSM2

Module11_Prolif_score DONSON

Module11_Prolif_score HMMR

Module11_Prolif_score BIRC5

Module11_Prolif_score KIF11

Module11_Prolif_score LMNB1

Module11_Prolif_score MAD2L1

Module11_Prolif_score MCM4

Module11_Prolif_score MCM5

Module11_Prolif_score MKI67

Module11_Prolif_score MMP1

Module11_Prolif_score MYBL1

Module11_Prolif_score MYBL2

Module11_Prolif_score NEK2

Module11_Prolif_score NUSAP1

Module11_Prolif_score GTSE1

Module11_Prolif_score GINS2

Module11_Prolif_score PLK1

Module11_Prolif_score ERCC6L

Module11_Prolif_score NCAPG2

Module11_Prolif_score CEP55

Module11_Prolif_score FANCI

Module11_Prolif_score HJURP

Module11_Prolif_score MCM10

Module11_Prolif_score DEPDC1

Module11_Prolif_score C1orf112

Module11_Prolif_score CENPN

Module11_Prolif_score PBK

Module11_Prolif_score KIF15

Module11_Prolif_score CIAPIN1

Module11_Prolif_score ACTR3B

Module11_Prolif_score SPC25

Module11_Prolif_score RAD21

Module11_Prolif_score RFC3

Module11_Prolif_score RFC4

Module11_Prolif_score RRM2

Module11_Prolif_score NCAPG

Module11_Prolif_score STIL

Module11_Prolif_score SKP2

Module11_Prolif_score SOX11

Module11_Prolif_score SQLE

Module11_Prolif_score AURKA

Module11_Prolif_score TAF2

Module11_Prolif_score TARS

Module11_Prolif_score BUB1B

Module11_Prolif_score TK1

Module11_Prolif_score TMPO

Module11_Prolif_score TOP2A

Module11_Prolif_score PHLDA2

Module11_Prolif_score TTK

Module11_Prolif_score LRP8

Module11_Prolif_score DSCC1

Module11_Prolif_score E2F8

Module11_Prolif_score SHCBP1

Module11_Prolif_score SLC7A5

Module11_Prolif_score ANP32E

Module11_Prolif_score KIF18A

Module11_Prolif_score CDC7

Module11_Prolif_score CDC45

Module11_Prolif_score RAD54L

Module11_Prolif_score TTF2

Module11_Prolif_score PIR

Module11_Prolif_score ACTL6A

Module11_Prolif_score GGH

Module11_Prolif_score CCNA2

Module11_Prolif_score CCNB1

Module11_Prolif_score PRC1

Module11_Prolif_score CCNB2

Module11_Prolif_score CCNE2

Module11_Prolif_score EXO1

Module11_Prolif_score AURKB

Module11_Prolif_score PTTG1

Module11_Prolif_score TRIP13

Module11_Prolif_score KIF23

Module11_Prolif_score APOBEC3B

Module11_Prolif_score MTFR1

Module11_Prolif_score ESPL1

Module11_Prolif_score DLGAP5

Module11_Prolif_score CDK1

Module11_Prolif_score MELK

Module11_Prolif_score GINS1

Module11_Prolif_score CDC6

Module11_Prolif_score CDC20

Module11_Prolif_score NCAPD2

Module11_Prolif_score KIF14

Module11_Prolif_score PIMREG

Module11_Prolif_score ADGRG6

Module11_Prolif_score CENPU

Module3_IFN_score IFI44

Module3_IFN_score IFI44L

Module3_IFN_score DDX58

Module3_IFN_score IFI6

Module3_IFN_score IFI27

Module3_IFN_score IFIT2

Module3_IFN_score IFIT1

Module3_IFN_score IFIT3

Module3_IFN_score CXCL10

Module3_IFN_score MX1

Module3_IFN_score OAS1

Module3_IFN_score OAS2

Module3_IFN_score OAS3

Module3_IFN_score HERC5

Module3_IFN_score SAMD9

Module3_IFN_score HERC6

Module3_IFN_score DDX60

Module3_IFN_score RTP4

Module3_IFN_score IFIH1

Module3_IFN_score STAT1

Module3_IFN_score TAP1

Module3_IFN_score OASL

Module3_IFN_score RSAD2

Module3_IFN_score ISG15

Module4_TcellBcell_score CD96

Module4_TcellBcell_score CD52

Module4_TcellBcell_score SEMA4D

Module4_TcellBcell_score CXCL13

Module4_TcellBcell_score SP140

Module4_TcellBcell_score CCR7

Module4_TcellBcell_score CTSW

Module4_TcellBcell_score DOCK2

Module4_TcellBcell_score EVI2B

Module4_TcellBcell_score FCN1

Module4_TcellBcell_score KLRK1

Module4_TcellBcell_score FLI1

Module4_TcellBcell_score PLCL2

Module4_TcellBcell_score IPCEF1

Module4_TcellBcell_score PPP1R16B

Module4_TcellBcell_score CCDC69

Module4_TcellBcell_score STAP1

Module4_TcellBcell_score GPR18

Module4_TcellBcell_score ICOS

Module4_TcellBcell_score GPR171

Module4_TcellBcell_score GZMA

Module4_TcellBcell_score GZMB

Module4_TcellBcell_score GZMK

Module4_TcellBcell_score IL2RB

Module4_TcellBcell_score IL2RG

Module4_TcellBcell_score IL7R

Module4_TcellBcell_score ITGA4

Module4_TcellBcell_score ITK

Module4_TcellBcell_score KLRB1

Module4_TcellBcell_score LCK

Module4_TcellBcell_score LGALS2

Module4_TcellBcell_score LRMP

Module4_TcellBcell_score LTB

Module4_TcellBcell_score SH2D1A

Module4_TcellBcell_score CXCL9

Module4_TcellBcell_score NCF4

Module4_TcellBcell_score GIMAP6

Module4_TcellBcell_score IL21R

Module4_TcellBcell_score TRAT1

Module4_TcellBcell_score PLAC8

Module4_TcellBcell_score UBASH3A

Module4_TcellBcell_score POU2AF1

Module4_TcellBcell_score RHOF

Module4_TcellBcell_score LAX1

Module4_TcellBcell_score BANK1

Module4_TcellBcell_score SIRPG

Module4_TcellBcell_score PRF1

Module4_TcellBcell_score DOCK10

Module4_TcellBcell_score PRKCB

Module4_TcellBcell_score CRTAM

Module4_TcellBcell_score PTGDS

Module4_TcellBcell_score PTPRC

Module4_TcellBcell_score PTPRCAP

Module4_TcellBcell_score TNFRSF17

Module4_TcellBcell_score CCL19

Module4_TcellBcell_score SELL

Module4_TcellBcell_score BCL11B

Module4_TcellBcell_score SLAMF1

Module4_TcellBcell_score TNFRSF1B

Module4_TcellBcell_score CCR2

Module4_TcellBcell_score TRAF3IP3

Module4_TcellBcell_score TCL1A

Module4_TcellBcell_score VNN2

Module4_TcellBcell_score PSTPIP1

Module4_TcellBcell_score CD2

Module4_TcellBcell_score CD3G

Module4_TcellBcell_score CD247

Module4_TcellBcell_score CD7

Module4_TcellBcell_score CD8A

Module4_TcellBcell_score CD19

Module4_TcellBcell_score MS4A1

Module4_TcellBcell_score CD27

Module4_TcellBcell_score AIM2

Module4_TcellBcell_score CD37

Module4_TcellBcell_score CYTIP

Module4_TcellBcell_score CD69

Module4_TcellBcell_score CD79A

Module4_TcellBcell_score P2RY14

Module4_TcellBcell_score RIPOR2

Module4_TcellBcell_score FYB1

Module4_TcellBcell_score JCHAIN

Module4_TcellBcell_score FAM30A

Module5_TcellBcell_score IGSF6

Module5_TcellBcell_score LILRB2

Module5_TcellBcell_score BTN3A3

Module5_TcellBcell_score UBD

Module5_TcellBcell_score CXCL13

Module5_TcellBcell_score GNLY

Module5_TcellBcell_score CXCR6

Module5_TcellBcell_score CTSC

Module5_TcellBcell_score HCP5

Module5_TcellBcell_score PIM2

Module5_TcellBcell_score SP140

Module5_TcellBcell_score CCR7

Module5_TcellBcell_score CTSS

Module5_TcellBcell_score CYBB

Module5_TcellBcell_score FCN1

Module5_TcellBcell_score TFEC

Module5_TcellBcell_score SEL1L3

Module5_TcellBcell_score GBP1

Module5_TcellBcell_score LAMP3

Module5_TcellBcell_score ADAMDEC1

Module5_TcellBcell_score GPR18

Module5_TcellBcell_score ICOS

Module5_TcellBcell_score GPR171

Module5_TcellBcell_score GZMH

Module5_TcellBcell_score GZMB

Module5_TcellBcell_score GZMK

Module5_TcellBcell_score BIRC3

Module5_TcellBcell_score IFNG

Module5_TcellBcell_score IL2RG

Module5_TcellBcell_score IL15

Module5_TcellBcell_score IDO1

Module5_TcellBcell_score CXCL10

Module5_TcellBcell_score IRF1

Module5_TcellBcell_score ISG20

Module5_TcellBcell_score ITK

Module5_TcellBcell_score LAG3

Module5_TcellBcell_score LCK

Module5_TcellBcell_score LYN

Module5_TcellBcell_score CXCL9

Module5_TcellBcell_score NKG7

Module5_TcellBcell_score TRAT1

Module5_TcellBcell_score PLAC8

Module5_TcellBcell_score POU2AF1

Module5_TcellBcell_score CRTAM

Module5_TcellBcell_score SLAMF8

Module5_TcellBcell_score PSMB9

Module5_TcellBcell_score PTPN7

Module5_TcellBcell_score SLAMF7

Module5_TcellBcell_score BCL2A1

Module5_TcellBcell_score TNFRSF17

Module5_TcellBcell_score CCL5

Module5_TcellBcell_score CCL8

Module5_TcellBcell_score CCL13

Module5_TcellBcell_score CCL18

Module5_TcellBcell_score CCL19

Module5_TcellBcell_score CXCL11

Module5_TcellBcell_score SELL

Module5_TcellBcell_score SAMSN1

Module5_TcellBcell_score RTP4

Module5_TcellBcell_score CLEC7A

Module5_TcellBcell_score TAP1

Module5_TcellBcell_score WARS

Module5_TcellBcell_score PLA2G7

Module5_TcellBcell_score ZBED2

Module5_TcellBcell_score NPL

Module5_TcellBcell_score RUNX3

Module5_TcellBcell_score VNN2

Module5_TcellBcell_score CD3G

Module5_TcellBcell_score IL32

Module5_TcellBcell_score CD8B

Module5_TcellBcell_score CD19

Module5_TcellBcell_score CD86

Module5_TcellBcell_score AIM2

Module5_TcellBcell_score CD38

Module5_TcellBcell_score CYTIP

Module5_TcellBcell_score CD69

Module5_TcellBcell_score CD79A

Module5_TcellBcell_score FYB1

Module5_TcellBcell_score LOC96610

Module5_TcellBcell_score MGC29506

NHI_5gene_score GBP1

NHI_5gene_score STAT1

NHI_5gene_score IGLL5

NHI_5gene_score OCLN

NHI_5gene_score IGK

PD1_data PDCD1

PD1_PDL1_score PDCD1

PD1_PDL1_score CD274

PDL1_data CD274

Rotterdam_ERneg_PCA_15721472 PARP4

Rotterdam_ERneg_PCA_15721472 COL2A1

Rotterdam_ERneg_PCA_15721472 FUT3

Rotterdam_ERneg_PCA_15721472 GAS2

Rotterdam_ERneg_PCA_15721472 MYH2

Rotterdam_ERneg_PCA_15721472 SAT1

Rotterdam_ERneg_PCA_15721472 TNFSF10

Rotterdam_ERneg_PCA_15721472 CEP57

Rotterdam_ERneg_PCA_15721472 ANAPC15

Rotterdam_ERneg_PCA_15721472 GABRQ

Rotterdam_ERneg_PCA_15721472 RFX7

Rotterdam_ERneg_PCA_15721472 BCL2L14

Rotterdam_ERneg_PCA_15721472 RPL23AP7

Rotterdam_ERneg_PCA_15721472 ZNF362

STAT1_19272155 TAP1

STAT1_19272155 GBP1

STAT1_19272155 IFIH1

STAT1_19272155 PSMB9

STAT1_19272155 CXCL9

STAT1_19272155 IRF1

STAT1_19272155 CXCL11

STAT1_19272155 CXCL10

STAT1_19272155 IDO1

STAT1_19272155 STAT1

STAT1_score STAT1

STAT1_score CXCL10

STAT1_score TAP1

STAT1_score CXCL11

STAT1_score CXCL9

STAT1_score MX1

STAT1_score LAMP3

STAT1_score ISG15

STAT1_score RTP4

STAT1_score HERC6

STAT1_score IFI44L

STAT1_score MX2

STAT1_score IFIT3

STAT1_score HERC5

STAT1_score RSAD2

STAT1_score DDX58

STAT1_score CCL5

STAT1_score ADAMDEC1

STAT1_score CD2

STAT1_score HCP5

STAT1_score NMI

STAT1_score SPOCK2

STAT1_score CCL8

STAT1_score TRIM22

STAT1_score LYZ

STAT1_score IRF1

STAT1_score LAG3

STAT1_score TFEC

STAT1_score UBD

STAT1_score SP140

STAT1_score CTSC

STAT1_score IFI6

STAT1_score PLA2G7

STAT1_score CD3G

STAT1_score PLAC8

STAT1_score FGL2

STAT1_score GZMK

STAT1_score CD48

STAT1_score STAT4

STAT1_score GPR18

STAT1_score P2RX5

STAT1_score IFI30

STAT1_score SH2D1A

STAT1_score LAPTM5

STAT1_score CD69

STAT1_score PTPN7

STAT1_score IRF8

STAT1_score PIM2

STAT1_score ETV7

STAT1_score GPR171

STAT1_score PSME1

STAT1_score BIRC3

STAT1_score FASLG

STAT1_score IFITM1

STAT1_score IFIT5

STAT1_score ITGB2

STAT1_score BTN3A2

STAT1_score HCLS1

STAT1_score SECTM1

STAT1_score ARHGAP15

STAT1_score KLRK1

STAT1_score IGSF6

STAT1_score SNX10

STAT1_score BST2

STAT1_score APOC1

STAT1_score ZC3HAV1

STAT1_score DDAH2

STAT1_score LILRA4

STAT1_score EBI3

STAT1_score KLRC3

STAT1_score CLEC4A

STAT1_score CD40LG

STAT1_score VAV1

STAT1_score GLRX

STAT1_score ACP5

STAT1_score RFX5

STAT1_score TRAF3

STAT1_score RAB8A

STAT1_score IL18

STAT1_score EFNA1

STAT1_score RASGRP1

STAT1_score CCRL2

STAT1_score DNAL4

STAT1_score ADA2

STAT1_score GPR183

STAT1_score TYMP

STAT1_score IDO1

STAT1_score CYTIP

STAT1_score REC8

T_cell_PCA_16704732 SLC35D2

T_cell_PCA_16704732 CD3E

T_cell_PCA_16704732 S100A8

T_cell_PCA_16704732 GATA3

T_cell_PCA_16704732 GIPC1

T_cell_PCA_16704732 CCL5

T_cell_PCA_16704732 PRKCA

T_cell_PCA_16704732 SLCO3A1

T_cell_PCA_16704732 CEP85L

T_cell_PCA_16704732 KLRB1

T_cell_PCA_16704732 CD5

T_cell_PCA_16704732 SH2D1A

T_cell_PCA_16704732 S100A10

T_cell_PCA_16704732 IL6ST

T_cell_PCA_16704732 CD247

T_cell_PCA_16704732 CD6

T_cell_PCA_16704732 SBK1

T_cell_PCA_16704732 GBP2

T_cell_PCA_16704732 KLRG1

T_cell_PCA_16704732 TIAM1

T_cell_PCA_16704732 MYBL1

T_cell_PCA_16704732 TACC3

T_cell_PCA_16704732 RASGRP1

T_cell_PCA_16704732 LAT

T_cell_PCA_16704732 OSBP2

T_cell_PCA_16704732 LIMA1

T_cell_PCA_16704732 UPP1

T_cell_PCA_16704732 RNF213

T_cell_PCA_16704732 MAPKAPK5

T_cell_PCA_16704732 ACVR2B

T_cell_PCA_16704732 HSPA1L

T_cell_PCA_16704732 LEF1

T_cell_PCA_16704732 MRPL27

T_cell_PCA_16704732 LRIG1

T_cell_PCA_16704732 RARRES3

T_cell_PCA_16704732 DPP4

T_cell_PCA_16704732 TRA

T_cell_PCA_16704732 APBA2

T_cell_PCA_16704732 SPOCK2

T_cell_PCA_16704732 MLLT3

T_cell_PCA_16704732 PCSK5

T_cell_PCA_16704732 BCL11B

T_cell_PCA_16704732 VIPR1

T_cell_PCA_16704732 RUNX2

T_cell_PCA_16704732 GALT

T_cell_PCA_16704732 ITGA6

T_cell_PCA_16704732 OLAH

T_cell_PCA_16704732 CD3G

T_cell_PCA_16704732 DNASE1L3

T_cell_PCA_16704732 CAMK4

T_cell_PCA_16704732 DNAJB1

T_cell_PCA_16704732 KLRC4

T_cell_PCA_16704732 TNFSF8

T_cell_PCA_16704732 NSG1

T_cell_PCA_16704732 SATB1

T_cell_PCA_16704732 GZMK

T_cell_PCA_16704732 IL18R1

T_cell_PCA_16704732 IFITM1

T_cell_PCA_16704732 LYAR

T_cell_PCA_16704732 CISH

T_cell_PCA_16704732 PRSS1

T_cell_PCA_16704732 PRKCI

T_cell_PCA_16704732 BIN2

T_cell_PCA_16704732 TRAT1

T_cell_PCA_16704732 SORL1

T_cell_PCA_16704732 CD3D

T_cell_PCA_16704732 PIK3IP1

T_cell_PCA_16704732 PCYT2

T_cell_PCA_16704732 CCND2

T_cell_PCA_16704732 CTSW

T_cell_PCA_16704732 ITK

T_cell_PCA_16704732 DISC1

T_cell_PCA_16704732 STAT4

T_cell_PCA_16704732 MAN1C1

T_cell_PCA_16704732 ITM2A

T_cell_PCA_16704732 MAP7D1

T_cell_PCA_16704732 TCF7

T_cell_PCA_16704732 TOMM40

T_cell_PCA_16704732 PKM

T_cell_PCA_16704732 SYT1

T_cell_PCA_16704732 NAP1L5

T_cell_PCA_16704732 LPAR6

T_cell_PCA_16704732 MATN2

T_cell_PCA_16704732 NR4A2

T_cell_PCA_16704732 SLFN5

T_cell_PCA_16704732 TOB1

T_cell_PCA_16704732 CD28

T_cell_PCA_16704732 PDE9A

T_cell_PCA_16704732 TMEM173

T_cell_PCA_16704732 DOCK9

T_cell_PCA_16704732 MORC2-AS1

T_cell_PCA_16704732 AOC1

T_cell_PCA_16704732 PTGER2

T_cell_PCA_16704732 LCP2

T_cell_PCA_16704732 RORA

T_cell_PCA_16704732 AKTIP

T_cell_PCA_16704732 NELL2

T_cell_PCA_16704732 SPEG

T_cell_PCA_16704732 LPIN2

T_cell_PCA_16704732 RTKN2

T_cell_PCA_16704732 FHIT

T_cell_PCA_16704732 PDE4D

T_cell_PCA_16704732 SLC39A8

T_cell_PCA_16704732 SELPLG

T_cell_PCA_16704732 SYNE2

T_cell_PCA_16704732 LEPROTL1

T_cell_PCA_16704732 BUB1B

T_cell_PCA_16704732 ACTN1

T_cell_PCA_16704732 AQP3

T_cell_PCA_16704732 PRKCQ

T_cell_PCA_16704732 RSU1

T_cell_PCA_16704732 GPSM3

T_cell_PCA_16704732 TSEN54

T_cell_PCA_16704732 SOCS3

T_cell_PCA_16704732 GABARAPL1

T_cell_PCA_16704732 C15orf62

T_cell_PCA_16704732 FYN

T_cell_PCA_16704732 ATP1A1

T_cell_PCA_16704732 DUSP16

T_cell_PCA_16704732 GBP1

T_cell_PCA_16704732 RGS10

T_cell_PCA_16704732 INPP4B

T_cell_PCA_16704732 TRABD2A

T_cell_PCA_16704732 RBMS1

T_cell_PCA_16704732 MAL

T_cell_PCA_16704732 RAB43

T_cell_PCA_16704732 ATP13A4

T_cell_PCA_16704732 ITPKB

T_cell_PCA_16704732 DUSP2

T_cell_PCA_16704732 TNIK

T_cell_PCA_16704732 LPAR2

T_cell_PCA_16704732 IL32

T_cell_PCA_16704732 APBB1

T_cell_PCA_16704732 ARL4C

T_cell_PCA_16704732 ID2

T_cell_PCA_16704732 PRKCQ-AS1

T_cell_PCA_16704732 TNFRSF25

T_cell_PCA_16704732 HOXB2

T_cell_PCA_16704732 CD2

T_cell_PCA_16704732 ADA

T_cell_PCA_16704732 JAKMIP1

T_cell_PCA_16704732 MGAT4A

T_cell_PCA_16704732 FAM102A

T_cell_PCA_16704732 RMDN1

T_cell_PCA_16704732 WWP1

T_cell_PCA_16704732 TARP

T_cell_PCA_16704732 NPTXR

T_cell_PCA_16704732 TESPA1

T_cell_PCA_16704732 LDHB

T_cell_PCA_16704732 PXN

T_cell_PCA_16704732 FBLN5

T_cell_PCA_16704732 TNFAIP3

T_cell_PCA_16704732 WNT10B

T_cell_PCA_16704732 LCK

T_cell_PCA_16704732 PIK3R1

T_cell_PCA_16704732 MEN1

T_cell_PCA_16704732 FLT3LG

T_cell_PCA_16704732 NPDC1

T_cell_PCA_16704732 ANXA1

T_cell_PCA_16704732 TXK

T_cell_PCA_16704732 MPP7

T_cell_PCA_16704732 IL7R

T_cell_PCA_16704732 ZAP70

T_cell_PCA_16704732 CEP41

T_cell_PCA_16704732 CAPZB

T_cell_PCA_16704732 CDC14A

T_cell_PCA_16704732 SNPH

T_cell_PCA_16704732 NOL4L

T_cell_PCA_16704732 RETREG1

T_cell_PCA_16704732 FYB1

T_cell_PCA_16704732 LINS1

T_cell_PCA_16704732 BEX3

T_cell_PCA_16704732 SEM1

TAMsurr_score CXCL10

TAMsurr_score CXCL11

TAMsurr_score CCL8

TAMsurr_score LAMP3

TAMsurr_TcClassII_ratio CXCL10

TAMsurr_TcClassII_ratio CXCL11

TAMsurr_TcClassII_ratio CCL8

TAMsurr_TcClassII_ratio LAMP3

TAMsurr_TcClassII_ratio CD2

TAMsurr_TcClassII_ratio CD3G

TAMsurr_TcClassII_ratio CD8A

TAMsurr_TcClassII_ratio IFNG

TAMsurr_TcClassII_ratio TNF

TAMsurr_TcClassII_ratio GZMB

TAMsurr_TcClassII_ratio GZMH

TAMsurr_TcClassII_ratio PRF1

TAMsurr_TcClassII_ratio ZAP70

TAMsurr_TcClassII_ratio HLA-DMA

TAMsurr_TcClassII_ratio HLA-DOA

TAMsurr_TcClassII_ratio HLA-DOB

TAMsurr_TcClassII_ratio HLA-DPA1

TAMsurr_TcClassII_ratio HLA-DPB1

TAMsurr_TcClassII_ratio HLA-DQA1

TAMsurr_TcClassII_ratio HLA-DQB1

TAMsurr_TcClassII_ratio HLA-DQB2

TAMsurr_TcClassII_ratio HLA-DRA

TAMsurr_TcClassII_ratio HLA-DRB1

TAMsurr_TcClassII_ratio HLA-DRB2

TAMsurr_TcClassII_ratio HLA-DRB3

TAMsurr_TcClassII_ratio HLA-DRB4

TAMsurr_TcClassII_ratio HLA-DRB5

TAMsurr_TcClassII_ratio HLA-DRB6

TAMsurr_TcClassII_ratio CIITA

TAMsurr_TcClassII_ratio CD74

TcClassII_score CD2

TcClassII_score CD3G

TcClassII_score CD8A

TcClassII_score IFNG

TcClassII_score TNF

TcClassII_score GZMB

TcClassII_score GZMH

TcClassII_score PRF1

TcClassII_score ZAP70

TcClassII_score HLA-DMA

TcClassII_score HLA-DOA

TcClassII_score HLA-DOB

TcClassII_score HLA-DPA1

TcClassII_score HLA-DPB1

TcClassII_score HLA-DQA1

TcClassII_score HLA-DQB1

TcClassII_score HLA-DQB2

TcClassII_score HLA-DRA

TcClassII_score HLA-DRB1

TcClassII_score HLA-DRB2

TcClassII_score HLA-DRB3

TcClassII_score HLA-DRB4

TcClassII_score HLA-DRB5

TcClassII_score HLA-DRB6

TcClassII_score CIITA

TcClassII_score CD74

Tcell_21978456 CORO1A

Tcell_21978456 LCK

Tcell_21978456 GZMK

Tcell_21978456 LAPTM5

Tcell_21978456 IL10RA

Tcell_21978456 CD27

Tcell_21978456 SELL

Tcell_21978456 TRAC

Tcell_21978456 CD48

Tcell_21978456 CD2

Tcell_21978456 TRBC1

Tcell_21978456 CD3D

Tcell_21978456 IL23A

Tcell_21978456 ITK

Tcell_21978456 CD53

Tcell_21978456 EVI2B

Tcell_21978456 PTPRC

Tcell_21978456 YME1L1

Tcell_21978456 TRAV20

Tcell_21978456 TRAJ17

Tcell_21978456 SAMSN1

Tcell_21978456 ARHGAP25

Tcell_21978456 PLAC8

Tcell_21978456 CD52

Tcell_receptors_score CD3D

Tcell_receptors_score CD3E

Tcell_receptors_score CD3G

Tcell_receptors_score CD247

TGFB_PCA_17349583 COL1A1

TGFB_PCA_17349583 FN1

TGFB_PCA_17349583 MMP9

TGFB_PCA_17349583 SERPINE1

TGFB_PCA_17349583 SPARC

TGFB_PCA_17349583 TAGLN

TGFB_PCA_17349583 TGFB1

TGFB_PCA_17349583 TGFB1I1

TGFB_PCA_17349583 TGFB3

TGFB_PCA_17349583 TGFBI

TGFB_PCA_17349583 TGFBR1

TGFB_PCA_17349583 TGIF1

TGFB_PCA_17349583 THBS1

TGFB_PCA_17349583 TGIF2

TGFB_PCA_17349583 CTGF

TGFB_score_21050467 MMP3

TGFB_score_21050467 MARCKSL1

TGFB_score_21050467 IGF2R

TGFB_score_21050467 LAMB1

TGFB_score_21050467 SPARC

TGFB_score_21050467 FN1

TGFB_score_21050467 ITGA4

TGFB_score_21050467 SMO

TGFB_score_21050467 MMP19

TGFB_score_21050467 ITGB8

TGFB_score_21050467 ITGA5

TGFB_score_21050467 NID1

TGFB_score_21050467 TIMP1

TGFB_score_21050467 SEMA3F

TGFB_score_21050467 RHOQ

TGFB_score_21050467 CTNNB1

TGFB_score_21050467 MMP2

TGFB_score_21050467 SERPINE1

TGFB_score_21050467 EPHB2

TGFB_score_21050467 COL16A1

TGFB_score_21050467 EPHA2

TGFB_score_21050467 TNC

TGFB_score_21050467 JUP

TGFB_score_21050467 ITGA3

TGFB_score_21050467 TCF7L2

TGFB_score_21050467 COL3A1

TGFB_score_21050467 CDH6

TGFB_score_21050467 WNT2B

TGFB_score_21050467 ADAM9

TGFB_score_21050467 DSP

TGFB_score_21050467 HSPG2

TGFB_score_21050467 ARHGAP1

TGFB_score_21050467 ITGB5

TGFB_score_21050467 IGFBP5

TGFB_score_21050467 ARHGDIA

TGFB_score_21050467 LRP1

TGFB_score_21050467 IGFBP2

TGFB_score_21050467 CTNNA1

TGFB_score_21050467 LRRC17

TGFB_score_21050467 MMP14

TGFB_score_21050467 NEO1

TGFB_score_21050467 EFNA5

TGFB_score_21050467 ITGB3

TGFB_score_21050467 EPHB3

TGFB_score_21050467 CD44

TGFB_score_21050467 IGFBP4

TGFB_score_21050467 TNFRSF1A

TGFB_score_21050467 RAC1

TGFB_score_21050467 PXN

TGFB_score_21050467 PLAT

TGFB_score_21050467 COL8A1

TGFB_score_21050467 WNT8B

TGFB_score_21050467 IGFBP3

TGFB_score_21050467 RHOA

TGFB_score_21050467 EPHB4

TGFB_score_21050467 MMP1

TGFB_score_21050467 PAK1

TGFB_score_21050467 MTA1

TGFB_score_21050467 THBS2

TGFB_score_21050467 MMP17

TGFB_score_21050467 CD59

TGFB_score_21050467 DVL3

TGFB_score_21050467 RHOB

TGFB_score_21050467 COL6A3

TGFB_score_21050467 NOTCH2

TGFB_score_21050467 BSG

TGFB_score_21050467 MMP11

TGFB_score_21050467 COL1A2

TGFB_score_21050467 ZYX

TGFB_score_21050467 RND3

TGFB_score_21050467 THBS1

TGFB_score_21050467 RHOG

TGFB_score_21050467 ICAM1

TGFB_score_21050467 LAMA4

TGFB_score_21050467 DVL1

TGFB_score_21050467 PAK2

TGFB_score_21050467 ITGB2

TGFB_score_21050467 COL6A1

TGFB_score_21050467 FGD1

TGFB_score_21050467 VCAN

TREM1_data TREM1

Troester_WoundSig_19887484 ADH1A

Troester_WoundSig_19887484 APOH

Troester_WoundSig_19887484 BMPR1B

Troester_WoundSig_19887484 DST

Troester_WoundSig_19887484 CAPN6

Troester_WoundSig_19887484 CD86

Troester_WoundSig_19887484 CDK4

Troester_WoundSig_19887484 COL17A1

Troester_WoundSig_19887484 COX7B

Troester_WoundSig_19887484 CSF3

Troester_WoundSig_19887484 CTSK

Troester_WoundSig_19887484 DMBT1

Troester_WoundSig_19887484 ARID3A

Troester_WoundSig_19887484 DSC3

Troester_WoundSig_19887484 ELANE

Troester_WoundSig_19887484 EYA4

Troester_WoundSig_19887484 F3

Troester_WoundSig_19887484 FABP4

Troester_WoundSig_19887484 FAP

Troester_WoundSig_19887484 FBLN1

Troester_WoundSig_19887484 EFEMP1

Troester_WoundSig_19887484 FPR1

Troester_WoundSig_19887484 FRZB

Troester_WoundSig_19887484 GJB5

Troester_WoundSig_19887484 HBB

Troester_WoundSig_19887484 HDC

Troester_WoundSig_19887484 HMGA1

Troester_WoundSig_19887484 HPCAL1

Troester_WoundSig_19887484 IL1B

Troester_WoundSig_19887484 IL13RA2

Troester_WoundSig_19887484 IDO1

Troester_WoundSig_19887484 INHBA

Troester_WoundSig_19887484 KRT6A

Troester_WoundSig_19887484 KRT6B

Troester_WoundSig_19887484 KRT34

Troester_WoundSig_19887484 AFF3

Troester_WoundSig_19887484 LAMA3

Troester_WoundSig_19887484 LAMC2

Troester_WoundSig_19887484 LTBP2

Troester_WoundSig_19887484 LUM

Troester_WoundSig_19887484 MSMB

Troester_WoundSig_19887484 ND4

Troester_WoundSig_19887484 NODAL

Troester_WoundSig_19887484 OGN

Troester_WoundSig_19887484 PCDH8

Troester_WoundSig_19887484 PDGFRL

Troester_WoundSig_19887484 PER1

Troester_WoundSig_19887484 PFKFB3

Troester_WoundSig_19887484 PITX1

Troester_WoundSig_19887484 PML

Troester_WoundSig_19887484 PNLIPRP2

Troester_WoundSig_19887484 S100A8

Troester_WoundSig_19887484 SAA4

Troester_WoundSig_19887484 CCL2

Troester_WoundSig_19887484 TGFB2

Troester_WoundSig_19887484 THBS1

Troester_WoundSig_19887484 TLR2

Troester_WoundSig_19887484 UGT2B17

Troester_WoundSig_19887484 ZIC2

Troester_WoundSig_19887484 TFPI2

Troester_WoundSig_19887484 NR4A3

Troester_WoundSig_19887484 SPARCL1

Troester_WoundSig_19887484 TP63

Troester_WoundSig_19887484 PER2

Troester_WoundSig_19887484 SPAG9

Troester_WoundSig_19887484 CXCL14

Troester_WoundSig_19887484 TMCC2

Troester_WoundSig_19887484 TNFSF15

Troester_WoundSig_19887484 CLEC4M

Troester_WoundSig_19887484 NPM2

Troester_WoundSig_19887484 HOXB13

Troester_WoundSig_19887484 PPP6R1

Troester_WoundSig_19887484 SULF1

Troester_WoundSig_19887484 DICER1

Troester_WoundSig_19887484 ABI3BP

Troester_WoundSig_19887484 EGFL6

Troester_WoundSig_19887484 TIPARP

Troester_WoundSig_19887484 RGS22

Troester_WoundSig_19887484 IGLV2-14

Troester_WoundSig_19887484 A1CF

Troester_WoundSig_19887484 TREM1

Troester_WoundSig_19887484 PGPEP1

Troester_WoundSig_19887484 RGMA

Troester_WoundSig_19887484 ADAMTS9

Troester_WoundSig_19887484 TENM2

Troester_WoundSig_19887484 VPS18

Troester_WoundSig_19887484 CACHD1

Troester_WoundSig_19887484 GUF1

Troester_WoundSig_19887484 ELSPBP1

Troester_WoundSig_19887484 ZBED2

Troester_WoundSig_19887484 LRRC2

Troester_WoundSig_19887484 CHPF

Troester_WoundSig_19887484 HMBOX1

Troester_WoundSig_19887484 TMC5

Troester_WoundSig_19887484 BAALC

Troester_WoundSig_19887484 APOLD1

Troester_WoundSig_19887484 VMP1

Troester_WoundSig_19887484 SPACA1

Troester_WoundSig_19887484 FRMD8P1

Troester_WoundSig_19887484 FAM71F1

Troester_WoundSig_19887484 TNS4

Troester_WoundSig_19887484 CROCCP3

Troester_WoundSig_19887484 OSR2

Troester_WoundSig_19887484 LRR1

Troester_WoundSig_19887484 TMEM139

Troester_WoundSig_19887484 CCBE1

Troester_WoundSig_19887484 FAM19A4

Troester_WoundSig_19887484 BMPER

Troester_WoundSig_19887484 YPEL4

Troester_WoundSig_19887484 JAKMIP3

Troester_WoundSig_19887484 LOC284454

Troester_WoundSig_19887484 KRT6C

Troester_WoundSig_19887484 ZNF713

Troester_WoundSig_19887484 SIMC1

Troester_WoundSig_19887484 SPDYE8P

Troester_WoundSig_19887484 LOC440934

Troester_WoundSig_19887484 MUC5B

Troester_WoundSig_19887484 KRT17P3

Troester_WoundSig_19887484 LMO7DN

Troester_WoundSig_19887484 CTGF

Troester_WoundSig_19887484 CYR61

Troester_WoundSig_19887484 CFAP74

Troester_WoundSig_19887484 LOC100131138

Troester_WoundSig_19887484 LOC389332

Troester_WoundSig_19887484 MKL2

Activated_B_cell TNFRSF17

Activated_B_cell BLK

Activated_B_cell CD19

Activated_B_cell MS4A1

Activated_B_cell CD27

Activated_B_cell CD38

Activated_B_cell CD79B

Activated_B_cell CR2

Activated_B_cell GNG7

Activated_B_cell HLA-DOB

Activated_B_cell IGHM

Activated_B_cell IGKC

Activated_B_cell CD180

Activated_B_cell PNOC

Activated_B_cell CCL21

Activated_B_cell SPIB

Activated_B_cell TCL1A

Activated_B_cell ARHGAP25

Activated_B_cell ADAM28

Activated_B_cell MICAL3

Activated_B_cell BACH2

Activated_B_cell FCRL2

Activated_B_cell AKNA

Activated_B_cell CLECL1

Activated_B_cell CLEC9A

Activated_B_cell CLEC17A

Activated_CD4_T_cell BIRC3

Activated_CD4_T_cell CCNB1

Activated_CD4_T_cell CCR7

Activated_CD4_T_cell DUSP2

Activated_CD4_T_cell ETS1

Activated_CD4_T_cell IARS

Activated_CD4_T_cell ITK

Activated_CD4_T_cell KIF11

Activated_CD4_T_cell RGS1

Activated_CD4_T_cell CCL4

Activated_CD4_T_cell CCL5

Activated_CD4_T_cell CCL20

Activated_CD4_T_cell SELL

Activated_CD4_T_cell PRC1

Activated_CD4_T_cell EXO1

Activated_CD4_T_cell AIM2

Activated_CD4_T_cell KNTC1

Activated_CD4_T_cell PSAT1

Activated_CD4_T_cell TRAT1

Activated_CD4_T_cell EXOC6

Activated_CD4_T_cell SAMSN1

Activated_CD4_T_cell NUF2

Activated_CD4_T_cell BRIP1

Activated_CD4_T_cell ESCO2

Activated_CD4_T_cell RTKN2

Activated_CD8_T_cell CD3D

Activated_CD8_T_cell CD3E

Activated_CD8_T_cell CD3G

Activated_CD8_T_cell CD8A

Activated_CD8_T_cell CD37

Activated_CD8_T_cell CD69

Activated_CD8_T_cell CETN3

Activated_CD8_T_cell CSE1L

Activated_CD8_T_cell GZMH

Activated_CD8_T_cell GZMA

Activated_CD8_T_cell GZMK

Activated_CD8_T_cell IL2RB

Activated_CD8_T_cell LCK

Activated_CD8_T_cell NKG7

Activated_CD8_T_cell ZAP70

Activated_CD8_T_cell MPZL1

Activated_CD8_T_cell GNLY

Activated_CD8_T_cell AHSA1

Activated_CD8_T_cell CCT6B

Activated_CD8_T_cell ADRM1

Activated_CD8_T_cell TIMM13

Activated_CD8_T_cell C1GALT1C1

Activated_CD8_T_cell PTRH2

Activated_CD8_T_cell GEMIN6

Activated_CD8_T_cell GPT2

Activated_CD8_T_cell PIK3IP1

Activated_dendritic_cell ABCD1

Activated_dendritic_cell RHOA

Activated_dendritic_cell ATP6V1A

Activated_dendritic_cell BCL2L1

Activated_dendritic_cell C1QB

Activated_dendritic_cell C1QC

Activated_dendritic_cell CAPG

Activated_dendritic_cell CEACAM8

Activated_dendritic_cell HLA-DQA2

Activated_dendritic_cell NOS2

Activated_dendritic_cell RAB1A

Activated_dendritic_cell SLA

Activated_dendritic_cell TNFAIP2

Activated_dendritic_cell TNFSF14

Activated_dendritic_cell TNFRSF6B

Activated_dendritic_cell SIGLEC5

Activated_dendritic_cell VNN2

Activated_dendritic_cell CCNA1

Activated_dendritic_cell SNURF

Activated_dendritic_cell CD302

Activated_dendritic_cell SRA1

Activated_dendritic_cell ACTR3

Activated_dendritic_cell UBD

Activated_dendritic_cell XPO6

Activated_dendritic_cell CD207

Activated_dendritic_cell SLC25A37

Activated_dendritic_cell TREM1

Activated_dendritic_cell SPCS3

Activated_dendritic_cell SLAMF9

Activated_dendritic_cell CLEC4C

Activated_dendritic_cell TREML4

Activated_dendritic_cell TREML1

Activated_dendritic_cell CCL3L3

Activated_dendritic_cell ATP5F1B

Activated_dendritic_cell ATP5MG

CD56bright_natural_killer_cell ABAT

CD56bright_natural_killer_cell ENTPD5

CD56bright_natural_killer_cell CDH3

CD56bright_natural_killer_cell CLIC2

CD56bright_natural_killer_cell CSF1

CD56bright_natural_killer_cell CSNK2A2

CD56bright_natural_killer_cell CSTA

CD56bright_natural_killer_cell CSTB

CD56bright_natural_killer_cell CTSD

CD56bright_natural_killer_cell CYP27B1

CD56bright_natural_killer_cell GATA2

CD56bright_natural_killer_cell GMPR

CD56bright_natural_killer_cell HDC

CD56bright_natural_killer_cell HOXA1

CD56bright_natural_killer_cell KRT86

CD56bright_natural_killer_cell GPR137B

CD56bright_natural_killer_cell DYNLL1

CD56bright_natural_killer_cell COX7A2L

CD56bright_natural_killer_cell HS2ST1

CD56bright_natural_killer_cell HS3ST1

CD56bright_natural_killer_cell FST

CD56bright_natural_killer_cell EIF3M

CD56bright_natural_killer_cell HCP5

CD56bright_natural_killer_cell HEY1

CD56bright_natural_killer_cell DCAF12

CD56bright_natural_killer_cell C5orf15

CD56bright_natural_killer_cell MLST8

CD56bright_natural_killer_cell BCL11B

CD56bright_natural_killer_cell NAA16

CD56bright_natural_killer_cell ELMOD3

CD56bright_natural_killer_cell CDHR1

CD56bright_natural_killer_cell MYL6B

CD56bright_natural_killer_cell CREB3L4

CD56bright_natural_killer_cell SMCO4

CD56bright_natural_killer_cell HDGFL2

CD56bright_natural_killer_cell METTL21A

CD56bright_natural_killer_cell TOGARAM2

CD56bright_natural_killer_cell CTPS1

CD56bright_natural_killer_cell C1QA

CD56bright_natural_killer_cell ClQB

CD56dim_natural_killer_cell CYP27A1

CD56dim_natural_killer_cell GRIN1

CD56dim_natural_killer_cell HLA-E

CD56dim_natural_killer_cell KIR2DS1

CD56dim_natural_killer_cell KIR2DS5

CD56dim_natural_killer_cell NOTCH3

CD56dim_natural_killer_cell PSMC4

CD56dim_natural_killer_cell RPL37A

CD56dim_natural_killer_cell UPP1

CD56dim_natural_killer_cell DYRK2

CD56dim_natural_killer_cell AKR7A3

CD56dim_natural_killer_cell IL21R

CD56dim_natural_killer_cell GPRC5C

CD56dim_natural_killer_cell DDX55

CD56dim_natural_killer_cell PORCN

CD56dim_natural_killer_cell KIR2DS2

Central_memory_CD4_T_cell ANXA2P2

Central_memory_CD4_T_cell AQP3

Central_memory_CD4_T_cell BMI1

Central_memory_CD4_T_cell KLF5

Central_memory_CD4_T_cell CD63

Central_memory_CD4_T_cell COL4A1

Central_memory_CD4_T_cell CYLD

Central_memory_CD4_T_cell FYN

Central_memory_CD4_T_cell XRCC6

Central_memory_CD4_T_cell GSS

Central_memory_CD4_T_cell ITGB1

Central_memory_CD4_T_cell ITGB2

Central_memory_CD4_T_cell LSP1

Central_memory_CD4_T_cell SMAD4

Central_memory_CD4_T_cell NDUFB9

Central_memory_CD4_T_cell PKM

Central_memory_CD4_T_cell STX4

Central_memory_CD4_T_cell VIM

Central_memory_CD4_T_cell TRADD

Central_memory_CD4_T_cell IFITM2

Central_memory_CD4_T_cell GLIPR1

Central_memory_CD4_T_cell BZW2

Central_memory_CD4_T_cell SIRPG

Central_memory_CD4_T_cell ELMO2

Central_memory_CD4_T_cell AHNAK

Central_memory_CD4_T_cell PGGHG

Central_memory_CD4_T_cell SFXN3

Central_memory_CD4_T_cell ABHD3

Central_memory_CD8_T_cell ACTN4

Central_memory_CD8_T_cell ADCY9

Central_memory_CD8_T_cell F13A1

Central_memory_CD8_T_cell FCER1G

Central_memory_CD8_T_cell FCGR3B

Central_memory_CD8_T_cell FGF7

Central_memory_CD8_T_cell FKBP4

Central_memory_CD8_T_cell GLUD1

Central_memory_CD8_T_cell GM2A

Central_memory_CD8_T_cell GUSB

Central_memory_CD8_T_cell IL1RN

Central_memory_CD8_T_cell NTRK1

Central_memory_CD8_T_cell RARA

Central_memory_CD8_T_cell SIGLEC1

Central_memory_CD8_T_cell UBA52

Central_memory_CD8_T_cell ADAM12

Central_memory_CD8_T_cell TNFRSF11A

Central_memory_CD8_T_cell TOX4

Central_memory_CD8_T_cell NOL11

Central_memory_CD8_T_cell RNF128

Central_memory_CD8_T_cell ULBP1

Effector_memory_CD4_T_cell ATM

Effector_memory_CD4_T_cell CASP3

Effector_memory_CD4_T_cell CASQ1

Effector_memory_CD4_T_cell SIGLEC6

Effector_memory_CD4_T_cell DARS

Effector_memory_CD4_T_cell EZH2

Effector_memory_CD4_T_cell NEFL

Effector_memory_CD4_T_cell PDGFRL

Effector_memory_CD4_T_cell EXOSC9

Effector_memory_CD4_T_cell PTGS1

Effector_memory_CD4_T_cell TAL1

Effector_memory_CD4_T_cell UQCRB

Effector_memory_CD4_T_cell WIPF1

Effector_memory_CD4_T_cell SCG2

Effector_memory_CD4_T_cell NCOA4

Effector_memory_CD4_T_cell USP9Y

Effector_memory_CD4_T_cell CAVIN2

Effector_memory_CD4_T_cell TFEC

Effector_memory_CD4_T_cell DOCK9

Effector_memory_CD4_T_cell TPK1

Effector_memory_CD4_T_cell GDE1

Effector_memory_CD4_T_cell TIPIN

Effector_memory_CD4_T_cell REPS1

Effector_memory_CD4_T_cell ZCRB1

Effector_memory_CD4_T_cell IL34

Effector_memory_CD4_T_cell CD300E

Effector_memory_CD4_T_cell SIGLEC14

Effector_memory_CD8_T_cell C3AR1

Effector_memory_CD8_T_cell CCR5

Effector_memory_CD8_T_cell CMKLR1

Effector_memory_CD8_T_cell CD55

Effector_memory_CD8_T_cell FLT3LG

Effector_memory_CD8_T_cell GZMM

Effector_memory_CD8_T_cell HLA-DMB

Effector_memory_CD8_T_cell HLA-DPA1

Effector_memory_CD8_T_cell HLA-DPB1

Effector_memory_CD8_T_cell IFI16

Effector_memory_CD8_T_cell LTK

Effector_memory_CD8_T_cell NFKBIA

Effector_memory_CD8_T_cell CFLAR

Effector_memory_CD8_T_cell ACAP1

Effector_memory_CD8_T_cell CD160

Effector_memory_CD8_T_cell DAPP1

Effector_memory_CD8_T_cell TRIB2

Effector_memory_CD8_T_cell LIME1

Effector_memory_CD8_T_cell ATP10D

Effector_memory_CD8_T_cell ARHGAP10

Effector_memory_CD8_T_cell APOL3

Effector_memory_CD8_T_cell SETD7

Effector_memory_CD8_T_cell HAPLN3

Effector_memory_CD8_T_cell SIK1

Effector_memory_CD8_T_cell FCRL6

Eosinophil DACH1

Eosinophil GPR183

Eosinophil FOSB

Eosinophil GIPR

Eosinophil IL5RA

Eosinophil LRMP

Eosinophil PDE6C

Eosinophil NR4A3

Eosinophil GPR65

Eosinophil DEPDC5

Eosinophil P2RY14

Eosinophil ST3GAL6

Eosinophil RRP12

Eosinophil DAPK2

Eosinophil PKD2L2

Eosinophil KRT18P50

Eosinophil ADGRE3

Gamma_delta_T_cell ACP5

Gamma_delta_T_cell AQP9

Gamma_delta_T_cell CD33

Gamma_delta_T_cell CD36

Gamma_delta_T_cell CDK5

Gamma_delta_T_cell FABP1

Gamma_delta_T_cell FABP5

Gamma_delta_T_cell IL10RB

Gamma_delta_T_cell LAMC1

Gamma_delta_T_cell LGALS1

Gamma_delta_T_cell LMNB1

Gamma_delta_T_cell MEIS3P1

Gamma_delta_T_cell MPL

Gamma_delta_T_cell MAPK7

Gamma_delta_T_cell RPS7

Gamma_delta_T_cell RPS9

Gamma_delta_T_cell RPS24

Gamma_delta_T_cell CCL13

Gamma_delta_T_cell CCL18

Gamma_delta_T_cell FADD

Gamma_delta_T_cell MINPP1

Gamma_delta_T_cell MFAP3L

Gamma_delta_T_cell BTN3A2

Gamma_delta_T_cell CARD8

Gamma_delta_T_cell DBNL

Gamma_delta_T_cell CD209

Gamma_delta_T_cell KLRF1

Gamma_delta_T_cell KLHL7

Gamma_delta_T_cell C1orf54

Gamma_delta_T_cell KRT80

Gamma_delta_T_cell LCORL

Immature__B_cell CD22

Immature__B_cell CYBB

Immature__B_cell HLA-DQA1

Immature__B_cell SP100

Immature__B_cell HDAC9

Immature__B_cell TXNIP

Immature__B_cell STAP1

Immature__B_cell P2RY10

Immature__B_cell ZCCHC2

Immature__B_cell FCRL5

Immature__B_cell HVCN1

Immature__B_cell FCRLA

Immature__B_cell FCRL1

Immature__B_cell FCRL3

Immature__B_cell TAGAP

Immature__B_cell FAM129C

Immature__B_cell NCF1

Immature__B_cell NCF1B

Immature__B_cell RUBCN

Immature_dendritic_cell ACADM

Immature_dendritic_cell ALDH9A1

Immature_dendritic_cell ALDH3A2

Immature_dendritic_cell ALOX15

Immature_dendritic_cell AMPD2

Immature_dendritic_cell AMT

Immature_dendritic_cell ARL1

Immature_dendritic_cell ATIC

Immature_dendritic_cell CAPZA1

Immature_dendritic_cell CLTB

Immature_dendritic_cell CSF3R

Immature_dendritic_cell TACSTD2

Immature_dendritic_cell PLAU

Immature_dendritic_cell RDX

Immature_dendritic_cell SLC18A2

Immature_dendritic_cell ALDH1A2

Immature_dendritic_cell AHCYL1

Immature_dendritic_cell INPP5F

Immature_dendritic_cell RAB38

Immature_dendritic_cell RRAGD

Immature_dendritic_cell C1orf162

Immature_dendritic_cell LILRA5

Immature_dendritic_cell ATP5F1A

Macrophage AIF1

Macrophage CD4

Macrophage CNR1

Macrophage CNR2

Macrophage CRYBB1

Macrophage EIF4A1

Macrophage MS4A2

Macrophage FES

Macrophage FPR1

Macrophage FPR2

Macrophage FZD2

Macrophage GPR27

Macrophage HK3

Macrophage HNMT

Macrophage HRH1

Macrophage RNASE2

Macrophage CCL1

Macrophage CCL14

Macrophage CCL23

Macrophage VNN1

Macrophage EIF1

Macrophage IGSF6

Macrophage CCL26

Macrophage BASP1

Macrophage FRAT2

Macrophage SLC15A3

Macrophage FRMD4A

Macrophage NPL

Macrophage WNT5B

Macrophage CD300LB

Macrophage C5AR2

Macrophage FAM198B

Macrophage NME8

Mast_cell CMA1

Mast_cell CPA3

Mast_cell CPM

Mast_cell CTSG

Mast_cell EGR3

Mast_cell FCN1

Mast_cell FTL

Mast_cell HSPA6

Mast_cell ITGA9

Mast_cell PTGS2

Mast_cell RNASE3

Mast_cell S100A4

Mast_cell SLC6A4

Mast_cell ADAMTS3

Mast_cell SIGLEC8

Mast_cell PILRA

Mast_cell ARHGAP15

MDSC CD2

MDSC CD14

MDSC CD86

MDSC FCGR2A

MDSC FCGR2B

MDSC FCGR3A

MDSC IL4R

MDSC ITGAL

MDSC ITGAM

MDSC PSAP

MDSC PTGER2

MDSC S100A8

MDSC S100A9

MDSC CXCR4

MDSC IL18BP

MDSC GPSM3

MDSC PARVG

MDSC PTGES2

MDSC FERMT3

MDSC CCR2

Memory_B_cell RUNX2

Memory_B_cell CCNA2

Memory_B_cell CDKN3

Memory_B_cell CLCN5

Memory_B_cell FCER1A

Memory_B_cell MYC

Memory_B_cell ENPP1

Memory_B_cell SORL1

Memory_B_cell SOX5

Memory_B_cell STAT5A

Memory_B_cell STAT5B

Memory_B_cell TLR9

Memory_B_cell AICDA

Memory_B_cell FCRL4

Monocyte ACTG1

Monocyte ANXA5

Monocyte ASGR1

Monocyte ASGR2

Monocyte ATP6V1B2

Monocyte CD1D

Monocyte CFL1

Monocyte CTBS

Monocyte HIVEP2

Monocyte MBP

Monocyte MMP15

Monocyte CFP

Monocyte TMBIM6

Monocyte UPK3A

Monocyte DAZAP2

Monocyte PQBP1

Monocyte IKZF1

Monocyte PNPLA6

Monocyte TEX264

Monocyte MARCKSL1

Monocyte ADGRE4P

Natural_killer_cell FASLG

Natural_killer_cell AXL

Natural_killer_cell BCL2

Natural_killer_cell BST2

Natural_killer_cell CDC5L

Natural_killer_cell CDH2

Natural_killer_cell CSF2RA

Natural_killer_cell CTSZ

Natural_killer_cell DAXX

Natural_killer_cell DPYD

Natural_killer_cell ERBB3

Natural_killer_cell FCGR1A

Natural_killer_cell FN1

Natural_killer_cell FUCA1

Natural_killer_cell FUT5

Natural_killer_cell GBP3

Natural_killer_cell GRB2

Natural_killer_cell CXCL1

Natural_killer_cell IGFBP5

Natural_killer_cell LST1

Natural_killer_cell FGF18

Natural_killer_cell CYTH1

Natural_killer_cell AKT3

Natural_killer_cell LDB3

Natural_killer_cell FSTL1

Natural_killer_cell KANK2

Natural_killer_cell GLS2

Natural_killer_cell F11R

Natural_killer_cell FZR1

Natural_killer_cell DLL4

Natural_killer_cell CRTAM

Natural_killer_cell FAM49A

Natural_killer_cell DGKH

Natural_killer_cell FAM27C

Natural_killer_cell GAGE2A

Natural_killer_T_cell CREB1

Natural_killer_T_cell CSF2

Natural_killer_T_cell FUT4

Natural_killer_T_cell HSPA4

Natural_killer_T_cell ICAM2

Natural_killer_T_cell ITIH2

Natural_killer_T_cell KIR2DL1

Natural_killer_T_cell KIR2DL3

Natural_killer_T_cell KIR2DS4

Natural_killer_T_cell KIR3DL1

Natural_killer_T_cell KIR3DL2

Natural_killer_T_cell KLRC1

Natural_killer_T_cell LAMP2

Natural_killer_T_cell MICB

Natural_killer_T_cell SDCBP

Natural_killer_T_cell SPP1

Natural_killer_T_cell THBD

Natural_killer_T_cell VCAM1

Natural_killer_T_cell FOSL1

Natural_killer_T_cell SLC7A7

Natural_killer_T_cell IL32

Natural_killer_T_cell CD101

Natural_killer_T_cell NCR1

Natural_killer_T_cell KLRG1

Natural_killer_T_cell BTN2A2

Natural_killer_T_cell PDPN

Natural_killer_T_cell CNPY3

Natural_killer_T_cell LILRB5

Natural_killer_T_cell KDM4C

Natural_killer_T_cell YBX2

Natural_killer_T_cell CLEC1A

Natural_killer_T_cell UBASH3A

Natural_killer_T_cell TREM2

Natural_killer_T_cell CCDC88A

Natural_killer_T_cell CRTC3

Natural_killer_T_cell KIRREL3

Natural_killer_T_cell NFATC2IP

Natural_killer_T_cell TSLP

Natural_killer_T_cell HSPB6

Natural_killer_T_cell CD109

Natural_killer_T_cell ISM2

Natural_killer_T_cell CRTC2

Natural_killer_T_cell CNPY4

Natural_killer_T_cell ADGRE1

Neutrophil CASP5

Neutrophil CDA

Neutrophil FFAR2

Neutrophil HAL

Neutrophil CXCR1

Neutrophil CXCR2

Neutrophil MAK

Neutrophil S100A12

Neutrophil TNFRSF10C

Neutrophil MGAM

Neutrophil CREB5

Neutrophil CHST15

Neutrophil VNN3

Neutrophil MMP25

Neutrophil STEAP4

Neutrophil BTNL8

Neutrophil APOBEC3A

Neutrophil INKA2

Plasmacytoid_dendritic_cell DAB2

Plasmacytoid_dendritic_cell EMP3

Plasmacytoid_dendritic_cell ENG

Plasmacytoid_dendritic_cell FCAR

Plasmacytoid_dendritic_cell GPX1

Plasmacytoid_dendritic_cell IDH3A

Plasmacytoid_dendritic_cell IGF1

Plasmacytoid_dendritic_cell IL3RA

Plasmacytoid_dendritic_cell ITGA2B

Plasmacytoid_dendritic_cell NUCB2

Plasmacytoid_dendritic_cell RALB

Plasmacytoid_dendritic_cell SEC14L1

Plasmacytoid_dendritic_cell OGT

Plasmacytoid_dendritic_cell OFD1

Plasmacytoid_dendritic_cell MAGED1

Plasmacytoid_dendritic_cell PDIA4

Plasmacytoid_dendritic_cell SERTAD2

Plasmacytoid_dendritic_cell KLHL21

Plasmacytoid_dendritic_cell DDX17

Plasmacytoid_dendritic_cell TMED2

Plasmacytoid_dendritic_cell GABARAP

Plasmacytoid_dendritic_cell CBX6

Plasmacytoid_dendritic_cell KRT23

Plasmacytoid_dendritic_cell HIGD1A

Plasmacytoid_dendritic_cell RNF141

Plasmacytoid_dendritic_cell PHRF1

Plasmacytoid_dendritic_cell PROK2

Plasmacytoid_dendritic_cell ABTB1

Plasmacytoid_dendritic_cell RETNLB

Plasmacytoid_dendritic_cell SIRPA

Plasmacytoid_dendritic_cell CD300LF

Plasmacytoid_dendritic_cell MSRB1

Regulatory_T_cell CD72

Regulatory_T_cell ITGA4

Regulatory_T_cell L1CAM

Regulatory_T_cell LIPA

Regulatory_T_cell LRP1

Regulatory_T_cell MMP12

Regulatory_T_cell MNDA

Regulatory_T_cell MRC1

Regulatory_T_cell PLEK

Regulatory_T_cell PTGIR

Regulatory_T_cell CCL3L1

Regulatory_T_cell ST8SIA4

Regulatory_T_cell MARCO

Regulatory_T_cell PRSS23

Regulatory_T_cell STAB1

Regulatory_T_cell CLEC5A

Regulatory_T_cell FOXP3

Regulatory_T_cell PELO

Regulatory_T_cell MS4A6A

Regulatory_T_cell LRRC42

T_follicular_helper_cell BCL6

T_follicular_helper_cell CEBPA

T_follicular_helper_cell CSF1R

T_follicular_helper_cell CTSS

T_follicular_helper_cell DPP4

T_follicular_helper_cell FGF2

T_follicular_helper_cell LRRC32

T_follicular_helper_cell GPR18

T_follicular_helper_cell KIR2DL2

T_follicular_helper_cell MC5R

T_follicular_helper_cell CD200

T_follicular_helper_cell NCAM1

T_follicular_helper_cell PRDX1

T_follicular_helper_cell PDCD1

T_follicular_helper_cell LGMN

T_follicular_helper_cell TYRO3

T_follicular_helper_cell RAE1

T_follicular_helper_cell NRP1

T_follicular_helper_cell CD84

T_follicular_helper_cell CDK5R1

T_follicular_helper_cell CLIC3

T_follicular_helper_cell CD83

T_follicular_helper_cell NCR2

T_follicular_helper_cell PDCD6

T_follicular_helper_cell CLEC10A

T_follicular_helper_cell IVNS1ABP

T_follicular_helper_cell SIGLEC7

T_follicular_helper_cell B3GAT1

T_follicular_helper_cell SIGLEC9

T_follicular_helper_cell CLEC4A

T_follicular_helper_cell CHST12

T_follicular_helper_cell PDCD1LG2

T_follicular_helper_cell RAET1E

T_follicular_helper_cell MICA

T_follicular_helper_cell ADA2

T_follicular_helper_cell SYNM

Type_1_T_helper_cell ADAM8

Type_1_T_helper_cell ALCAM

Type_1_T_helper_cell BST1

Type_1_T_helper_cell CALD1

Type_1_T_helper_cell CD6

Type_1_T_helper_cell CD7

Type_1_T_helper_cell CD47

Type_1_T_helper_cell CD48

Type_1_T_helper_cell CD53

Type_1_T_helper_cell CD59

Type_1_T_helper_cell CD68

Type_1_T_helper_cell CD70

Type_1_T_helper_cell CD151

Type_1_T_helper_cell CD52

Type_1_T_helper_cell CHRM3

Type_1_T_helper_cell COL4A4

Type_1_T_helper_cell DAB1

Type_1_T_helper_cell EMP1

Type_1_T_helper_cell F12

Type_1_T_helper_cell GATM

Type_1_T_helper_cell GPR25

Type_1_T_helper_cell HSD11B1

Type_1_T_helper_cell HTR2B

Type_1_T_helper_cell ICAM3

Type_1_T_helper_cell IGF2

Type_1_T_helper_cell IL12A

Type_1_T_helper_cell IRF1

Type_1_T_helper_cell ITGB4

Type_1_T_helper_cell LTC4S

Type_1_T_helper_cell MDH1

Type_1_T_helper_cell P2RX5

Type_1_T_helper_cell FURIN

Type_1_T_helper_cell RGS16

Type_1_T_helper_cell RYR1

Type_1_T_helper_cell SELE

Type_1_T_helper_cell SELP

Type_1_T_helper_cell STAC

Type_1_T_helper_cell TNFRSF1A

Type_1_T_helper_cell TRAF1

Type_1_T_helper_cell DOC2B

Type_1_T_helper_cell ENC1

Type_1_T_helper_cell SKAP1

Type_1_T_helper_cell B3GALNT1

Type_1_T_helper_cell FCGR2C

Type_1_T_helper_cell ZEB2

Type_1_T_helper_cell GFPT2

Type_1_T_helper_cell CD96

Type_1_T_helper_cell TRAF3IP2

Type_1_T_helper_cell CFHR3

Type_1_T_helper_cell DUSP14

Type_1_T_helper_cell MAN1B1

Type_1_T_helper_cell AHCYL2

Type_1_T_helper_cell MMD

Type_1_T_helper_cell HAVCR1

Type_1_T_helper_cell SIT1

Type_1_T_helper_cell TBX21

Type_1_T_helper_cell SH3KBP1

Type_1_T_helper_cell HUNK

Type_1_T_helper_cell COL5A3

Type_1_T_helper_cell SLC35B3

Type_1_T_helper_cell TLR8

Type_1_T_helper_cell MOCOS

Type_1_T_helper_cell SAV1

Type_1_T_helper_cell GREM2

Type_1_T_helper_cell CLEC7A

Type_1_T_helper_cell THUMPD2

Type_1_T_helper_cell FBXO30

Type_1_T_helper_cell SIGLEC10

Type_1_T_helper_cell COL23A1

Type_1_T_helper_cell RCSD1

Type_1_T_helper_cell GAB3

Type_1_T_helper_cell BBS12

Type_1_T_helper_cell TIGIT

Type_1_T_helper_cell DLEU7

Type_1_T_helper_cell METRNL

Type_1_T_helper_cell RETREG1

Type_1_T_helper_cell ADGRE5

Type_17_T_helper_cell ABCA1

Type_17_T_helper_cell ANK1

Type_17_T_helper_cell CA2

Type_17_T_helper_cell CD40

Type_17_T_helper_cell CEACAM3

Type_17_T_helper_cell IL17A

Type_17_T_helper_cell ABCB1

Type_17_T_helper_cell B3GALT2

Type_17_T_helper_cell CCR9

Type_17_T_helper_cell CAMTA1

Type_17_T_helper_cell IL17RA

Type_17_T_helper_cell IL17C

Type_17_T_helper_cell IL23A

Type_17_T_helper_cell IFT80

Type_17_T_helper_cell TNIP2

Type_17_T_helper_cell LONRF3

Type_17_T_helper_cell ADAMTS12

Type_17_T_helper_cell IL17RC

Type_17_T_helper_cell CCDC65

Type_17_T_helper_cell IL17F

Type_17_T_helper_cell ANKRD22

Type_17_T_helper_cell IL17RE

Type_17_T_helper_cell C2CD4A

Type_17_T_helper_cell SH2D6

Type_17_T_helper_cell ILDR1

Type_17_T_helper_cell C2CD4B

Type_17_T_helper_cell PTGDR2

Type_2_T_helper_cell BIRC5

Type_2_T_helper_cell CDC25C

Type_2_T_helper_cell CENPF

Type_2_T_helper_cell CSRP2

Type_2_T_helper_cell DAPK1

Type_2_T_helper_cell DHFR

Type_2_T_helper_cell DUSP6

Type_2_T_helper_cell GATA3

Type_2_T_helper_cell GNAI1

Type_2_T_helper_cell GSTA4

Type_2_T_helper_cell HELLS

Type_2_T_helper_cell LAIR2

Type_2_T_helper_cell PDE4B

Type_2_T_helper_cell PLA2G4A

Type_2_T_helper_cell RAB27B

Type_2_T_helper_cell EVI5

Type_2_T_helper_cell CDC7

Type_2_T_helper_cell NRP2

Type_2_T_helper_cell DLC1

Type_2_T_helper_cell CXCR6

Type_2_T_helper_cell PHLDA1

Type_2_T_helper_cell LAMP3

Type_2_T_helper_cell RBMS3

Type_2_T_helper_cell ASB2

Type_2_T_helper_cell RNF125

Type_2_T_helper_cell IL26

Type_2_T_helper_cell DNAJC12

Type_2_T_helper_cell TMPRSS3

Type_2_T_helper_cell OSBPL1A

B_Cell ZNF532

B_Cell ZNF207

B_Cell ZNF154

B_Cell ZCCHC7

B_Cell XYLT1

B_Cell WIPF1

B_Cell WEE1

B_Cell WDR83OS

B_Cell WDR34

B_Cell WDR11

B_Cell VPS53

B_Cell VPS28

B_Cell VPREB3

B_Cell UVRAG

B_Cell USP6NL

B_Cell UROS

B_Cell UBE2J1

B_Cell TUBB6

B_Cell TTC7A

B_Cell TSPAN9

B_Cell TSPAN33

B_Cell TSPAN3

B_Cell TRIO

B_Cell TRIM56

B_Cell TRIM26

B_Cell TPD52

B_Cell TNS3

B_Cell TNFRSF18

B_Cell TNFRSF17

B_Cell TMED8

B_Cell TLR7

B_Cell TLR10

B_Cell TFEB

B_Cell TEAD2

B_Cell TCL1A

B_Cell TCF4

B_Cell TBC1D1

B_Cell SYPL1

B_Cell SYNGR2

B_Cell SYK

B_Cell SWAP70

B_Cell SUN2

B_Cell STX7

B_Cell STRBP

B_Cell STAT6

B_Cell STAP1

B_Cell STAG3

B_Cell ST14

B_Cell SSU72

B_Cell SSPN

B_Cell SRGAP2

B_Cell SPIB

B_Cell SPI1

B_Cell SP140

B_Cell SNX2

B_Cell SNX10

B_Cell SMC6

B_Cell SMAD3

B_Cell SLC7A7

B_Cell SLC2A5

B_Cell SLC2A1

B_Cell SLC25A27

B_Cell SLC22A3

B_Cell SKAP2

B_Cell SIDT2

B_Cell SHMT2

B_Cell SH3BP5

B_Cell SETBP1

B_Cell SEMA4B

B_Cell SEL1L3

B_Cell SCRN1

B_Cell SAV1

B_Cell SAMD9

B_Cell RUBCNL

B_Cell RRAS2

B_Cell RNF141

B_Cell RNASE6

B_Cell RIPK2

B_Cell RHOH

B_Cell RHOBTB2

B_Cell RGS13

B_Cell RFX5

B_Cell RCSD1

B_Cell RALGPS2

B_Cell RALGAPB

B_Cell RAB30

B_Cell PSEN2

B_Cell PRKCE

B_Cell PRICKLE1

B_Cell PRCP

B_Cell PPP3CA

B_Cell POU2F2

B_Cell POU2AF1

B_Cell POLD4

B_Cell PNOC

B_Cell PMEPA1

B_Cell PMAIP1

B_Cell PLPP5

B_Cell PLEKHO1

B_Cell PLEKHF2

B_Cell PLCG2

B_Cell PIK3C2B

B_Cell PIK3AP1

B_Cell PEA15

B_Cell PCCA

B_Cell PAX5

B_Cell PARP14

B_Cell PALM2-AKAP2

B_Cell OSBPL10

B_Cell ODC1

B_Cell NUP88

B_Cell NKG7

B_Cell NFKBIE

B_Cell NCF4

B_Cell NCF1

B_Cell NAPSB

B_Cell NAP1L1

B_Cell NANS

B_Cell MYO1E

B_Cell MYBL2

B_Cell MTSS1

B_Cell MTPN

B_Cell MS4A1

B_Cell MRPL49

B_Cell MOB3B

B_Cell MMP11

B_Cell MIR600HG

B_Cell MGME1

B_Cell METTL7A

B_Cell MEF2C

B_Cell MARCKS

B_Cell 1-Mar

B_Cell MAP3K8

B_Cell LYN

B_Cell LY86

B_Cell LRMP

B_Cell LHFPL2

B_Cell LGALS9

B_Cell LARGE2

B_Cell KYNU

B_Cell KLHL14

B_Cell KLF1

B_Cell KDM4B

B_Cell KAZN

B_Cell JUP

B_Cell JCHAIN

B_Cell JADE3

B_Cell ITPR1

B_Cell ITGB1

B_Cell IRF8

B_Cell IRF4

B_Cell INPPL1

B_Cell IL4R

B_Cell IGLL3P

B_Cell IGLL1

B_Cell IGLC2

B_Cell IGL

B_Cell IGKV3D-11

B_Cell IGKV1-5

B_Cell IGKC

B_Cell IGHM

B_Cell IGHG3

B_Cell IGHG1

B_Cell IGHD

B_Cell IGH

B_Cell IFNGR2

B_Cell IFIT3

B_Cell IFI27

B_Cell HVCN1

B_Cell HUWE1

B_Cell HSPA6

B_Cell HSPA5

B_Cell HRK

B_Cell HLA-DRB6

B_Cell HLA-DRB5

B_Cell HLA-DRB1

B_Cell HLA-DRA

B_Cell HLA-DQB2

B_Cell HLA-DQB1

B_Cell HLA-DQA2

B_Cell HLA-DQA1

B_Cell HLA-DPB1

B_Cell HLA-DPA1

B_Cell HLA-DOB

B_Cell HLA-DOA

B_Cell HLA-DMB

B_Cell HLA-DMA

B_Cell HIST1H2BK

B_Cell HHEX

B_Cell HERPUD1

B_Cell HECW2

B_Cell GUCD1

B_Cell GSTZ1

B_Cell GSAP

B_Cell GRK3

B_Cell GNA12

B_Cell GM2A

B_Cell GLDC

B_Cell GCNT1

B_Cell FZD5

B_Cell FUBP1

B_Cell FOXP1

B_Cell FLII

B_Cell FIG4

B_Cell FGD2

B_Cell FCRLA

B_Cell FCRL5

B_Cell FCRL2

B_Cell FCRL1

B_Cell FCGR2B

B_Cell FBXO41

B_Cell FBXO10

B_Cell FAM30A

B_Cell FAM129C

B_Cell F5

B_Cell EVI5L

B_Cell EPHX1

B_Cell EPB41L2

B_Cell EGR1

B_Cell EBF1

B_Cell E2F5

B_Cell DTX1

B_Cell DRAM2

B_Cell DMXL1

B_Cell DENND5B

B_Cell DENND4B

B_Cell DDR1

B_Cell DAPP1

B_Cell CYTH1

B_Cell CYSLTR1

B_Cell CYBB

B_Cell CYB561D2

B_Cell CYB561A3

B_Cell CXCR5

B_Cell CTSZ

B_Cell CTSH

B_Cell CR2

B_Cell CR1

B_Cell CORO2B

B_Cell CORO1C

B_Cell CORO1A

B_Cell COPS3

B_Cell COL14A1

B_Cell CNR2

B_Cell CLIC4

B_Cell CIITA

B_Cell CHERP

B_Cell CHD7

B_Cell CEBPB

B_Cell CDKN2A

B_Cell CDCA7L

B_Cell CD86

B_Cell CD83

B_Cell CD79B

B_Cell CD79A

B_Cell CD74

B_Cell CD72

B_Cell CD38

B_Cell CD24

B_Cell CD22

B_Cell CD200

B_Cell CD1D

B_Cell CD1C

B_Cell CD19

B_Cell CD180

B_Cell CCNG2

B_Cell CCDC50

B_Cell C11orf24

B_Cell BTNL9

B_Cell BTLA

B_Cell BTK

B_Cell BSG

B_Cell BRD4

B_Cell BMF

B_Cell BLNK

B_Cell BLK

B_Cell BIRC3

B_Cell BCL7A

B_Cell BCL11A

B_Cell BANK1

B_Cell BACE2

B_Cell ATP6V0A1

B_Cell ATP5F1B

B_Cell ATG4A

B_Cell ARHGEF3

B_Cell ARHGAP10

B_Cell APOBEC3B

B_Cell ANXA4

B_Cell AMFR

B_Cell ALOX5

B_Cell AIM2

B_Cell AFF3

B_Cell ADK

B_Cell ADCYAP1

B_Cell ADAM28

B_Cell ADAM19

B_Cell ACTA2

B_Cell ABCA1

B_Cell GRAMD1C

B_Cell UBR5

B_Cell DNAJC10

B_Cell TBC1D10C

B_Cell CCDC25

B_Cell TCTN1

B_Cell RAP1GAP2

B_Cell_60gene SEL1L3

B_Cell_60gene SCFV

B_Cell_60gene POU2AF1

B_Cell_60gene MZB1

B_Cell_60gene MIR8071-2

B_Cell_60gene MIR8071-1

B_Cell_60gene JCHAIN

B_Cell_60gene IGLV1-44

B_Cell_60gene IGLV@

B_Cell_60gene IGLL5

B_Cell_60gene IGLL3P

B_Cell_60gene IGLJ3

B_Cell_60gene IGLC1

B_Cell_60gene IGKV1D-13

B_Cell_60gene IGKC

B_Cell_60gene IGK

B_Cell_60gene IGHV4-31

B_Cell_60gene IGHV3-23

B_Cell_60gene IGHV1-69

B_Cell_60gene IGHM

B_Cell_60gene IGHG4

B_Cell_60gene IGHG3

B_Cell_60gene IGHG2

B_Cell_60gene IGHG1

B_Cell_60gene IGHD

B_Cell_60gene IGHA2

B_Cell_60gene IGHA1

B_Cell_60gene IGH

B_Cell_60gene GUSBP11

B_Cell_60gene CYAT1

B_Cell_60gene CKAP2

B_Cell_60gene CD38

B_Cell_cluster TNFRSF17

B_Cell_cluster TNFRSF13B

B_Cell_cluster POU2AF1

B_Cell_cluster PAX5

B_Cell_cluster NUGGC

B_Cell_cluster MS4A1

B_Cell_cluster MEI1

B_Cell_cluster JCHAIN

B_Cell_cluster FCRLA

B_Cell_cluster FCRL5

B_Cell_cluster FCRL2

B_Cell_cluster FCRL1

B_Cell_cluster FCER2

B_Cell_cluster FAM30A

B_Cell_cluster CXCR5

B_Cell_cluster CNR2

B_Cell_cluster CD79B

B_Cell_cluster CD79A

B_Cell_cluster CD19

B_Cell_cluster BMS1P20

B_Cell_cluster BLK

B_Cell_cluster BANK1

B_Cell_cluster ADAM6

CD68_cluster SIGLEC9

CD68_cluster SIGLEC7

CD68_cluster LRRC25

CD68_cluster ITGAX

CD68_cluster CD68

CD68_cluster C3AR1

CD68_cluster ADAP2

CD8 ZBTB16

CD8 TSPAN32

CD8 TBX21

CD8 S100B

CD8 PRF1

CD8 KLRG1

CD8 KLRC1

CD8 IL2RB

CD8 GZMH

CD8 GNLY

CD8 FCGBP

CD8 DUSP2

CD8 CST7

CD8 CD8B

CD8 CD8A

CD8 CCL5

CD8 CCL4L1

CD8 C1orf21

CD8 ADRB2

CD8 ADGRG1

CD8 ?KLRK1

CD8_cluster ZNF831

CD8_cluster ZAP70

CD8_cluster UBASH3A

CD8_cluster TRAT1

CD8_cluster TIGIT

CD8_cluster TBX21

CD8_cluster TBC1D10C

CD8_cluster SLAMF6

CD8_cluster SLAMF1

CD8_cluster SLA2

CD8_cluster SIT1

CD8_cluster SIRPG

CD8_cluster SH2D1A

CD8_cluster SCML4

CD8_cluster SAMD3

CD8_cluster S1PR4

CD8_cluster PYHIN1

CD8_cluster PTPRCAP

CD8_cluster PTPN7

CD8_cluster PRKCB

CD8_cluster PRF1

CD8_cluster NKG7

CD8_cluster LY9

CD8_cluster LCK

CD8_cluster KLRK1

CD8_cluster ITK

CD8_cluster IL2RG

CD8_cluster GZMK

CD8_cluster GZMA

CD8_cluster GPR171

CD8_cluster CXCR6

CD8_cluster CXCR3

CD8_cluster CD96

CD8_cluster CD8A

CD8_cluster CD6

CD8_cluster CD5

CD8_cluster CD48

CD8_cluster CD3G

CD8_cluster CD3E

CD8_cluster CD3D

CD8_cluster CD27

CD8_cluster CD247

CD8_cluster CD2

CD8_cluster CCL5

CD8_cluster C16orf54

CD8_cluster BTLA

CD8_cluster ACAP1

IGG_Cluster TNFRSF17

IGG_Cluster POU2AF1

IGG_Cluster PIM2

IGG_Cluster NTN3

IGG_Cluster LAX1

IGG_Cluster JCHAIN

IGG_Cluster IL2RG

IGG_Cluster IGLV3-25

IGG_Cluster IGL

IGG_Cluster IGKC

IGG_Cluster HLA-C

IGG_Cluster CXCL8

IGG_Cluster CD79A

IGG_Cluster CD27

LCK IL2RG

LCK IL12RG

LCK CCL9

LCK CCL8

LCK CCL7

LCK CCL6

LCK CCL5

LCK CCL28

LCK CCL27

LCK CCL26

LCK CCL25

LCK CCL24

LCK CCL23

LCK CCL22

LCK CCL21

LCK CCL20

LCK CCL19

LCK CCL18

LCK CCL17

LCK CCL16

LCK CCL15

LCK CCL14

LCK CCL13

LCK CCL12

LCK CCL11

LCK CCL10

Mac_CSF1 ZNRF2

Mac_CSF1 ZNF44

Mac_CSF1 ZNF414

Mac_CSF1 ZNF384

Mac_CSF1 ZNF124

Mac_CSF1 ZEB2

Mac_CSF1 ZCCHC2

Mac_CSF1 YWHAZ

Mac_CSF1 YWHAH

Mac_CSF1 YPEL2

Mac_CSF1 XPC

Mac_CSF1 WISP2

Mac_CSF1 WIPF1

Mac_CSF1 WASHC4

Mac_CSF1 VSIG4

Mac_CSF1 VAMP8

Mac_CSF1 USP6NL

Mac_CSF1 USP24

Mac_CSF1 UHMK1

Mac_CSF1 UCP2

Mac_CSF1 UBE2W

Mac_CSF1 UAP1

Mac_CSF1 TYMP

Mac_CSF1 TWISTNB

Mac_CSF1 TTC13

Mac_CSF1 TRPS1

Mac_CSF1 TRAK2

Mac_CSF1 TRAK1

Mac_CSF1 TPR

Mac_CSF1 TPP1

Mac_CSF1 TPM3

Mac_CSF1 TNFSF13B

Mac_CSF1 TNFSF10

Mac_CSF1 TNFRSF21

Mac_CSF1 TNFRSF1B

Mac_CSF1 TNFAIP6

Mac_CSF1 TNFAIP3

Mac_CSF1 TNFAIP2

Mac_CSF1 TMSB4Y

Mac_CSF1 TMSB4X

Mac_CSF1 TMPRSS13

Mac_CSF1 TMEM9B

Mac_CSF1 TMEM37

Mac_CSF1 TMEM273

Mac_CSF1 TMEM206

Mac_CSF1 TMEM106A

Mac_CSF1 TMCC3

Mac_CSF1 TM7SF3

Mac_CSF1 TLR2

Mac_CSF1 TLR1

Mac_CSF1 TIMP3

Mac_CSF1 TIAM1

Mac_CSF1 THBD

Mac_CSF1 TGOLN2

Mac_CSF1 TGFBR1

Mac_CSF1 TES

Mac_CSF1 TEC

Mac_CSF1 TCN1

Mac_CSF1 TC2N

Mac_CSF1 TBXAS1

Mac_CSF1 TBC1D12

Mac_CSF1 SYK

Mac_CSF1 SWAP70

Mac_CSF1 STXBP3

Mac_CSF1 STX7

Mac_CSF1 STX2

Mac_CSF1 STK4

Mac_CSF1 STK38L

Mac_CSF1 STEAP4

Mac_CSF1 SRGN

Mac_CSF1 SQOR

Mac_CSF1 SPTLC2

Mac_CSF1 SPRR1B

Mac_CSF1 SPRED1

Mac_CSF1 SPINT2

Mac_CSF1 SP140

Mac_CSF1 SOS2

Mac_CSF1 SORL1

Mac_CSF1 SORBS2

Mac_CSF1 SOD2

Mac_CSF1 SOCS6

Mac_CSF1 SNX24

Mac_CSF1 SNX2

Mac_CSF1 SNAPC3

Mac_CSF1 SLF1

Mac_CSF1 SLC9A6

Mac_CSF1 SLC9A3R1

Mac_CSF1 SLC7A7

Mac_CSF1 SLC6A8

Mac_CSF1 SLC43A3

Mac_CSF1 SLC39A8

Mac_CSF1 SLC38A6

Mac_CSF1 SLC36A1

Mac_CSF1 SLC31A2

Mac_CSF1 SLC2A5

Mac_CSF1 SLC29A1

Mac_CSF1 SLC20A1

Mac_CSF1 SLC1A3

Mac_CSF1 SLC18B1

Mac_CSF1 SLC11A2

Mac_CSF1 SKAP2

Mac_CSF1 SIRPA

Mac_CSF1 SHMT1

Mac_CSF1 SHBG

Mac_CSF1 SH3BP2

Mac_CSF1 SH2B3

Mac_CSF1 SGPL1

Mac_CSF1 SGMS1

Mac_CSF1 SGK1

Mac_CSF1 SETDB2

Mac_CSF1 SERPINE2

Mac_CSF1 SERPINB8

Mac_CSF1 SERPINB1

Mac_CSF1 8-Sep 6-Sep

Mac_CSF1 SEMA6A

Mac_CSF1 SELPLG

Mac_CSF1 SELL

Mac_CSF1 SCPEP1

Mac_CSF1 SCP2

Mac_CSF1 SCD

Mac_CSF1 SCAMP2

Mac_CSF1 SAT1

Mac_CSF1 SAMSN1

Mac_CSF1 SAMHD1

Mac_CSF1 SAMD9L

Mac_CSF1 S1PR1

Mac_CSF1 S100B

Mac_CSF1 S100A9

Mac_CSF1 S100A8

Mac_CSF1 S100A11

Mac_CSF1 RUNX3

Mac_CSF1 RUBCNL

Mac_CSF1 RTN3

Mac_CSF1 RRM2

Mac_CSF1 RPS6KA1

Mac_CSF1 RNH1

Mac_CSF1 RNF149

Mac_CSF1 RNF144B

Mac_CSF1 RNF135

Mac_CSF1 RNF130

Mac_CSF1 RNF13

Mac_CSF1 RNASE6

Mac_CSF1 RNASE1

Mac_CSF1 RHOG

Mac_CSF1 RHOA

Mac_CSF1 RGS10

Mac_CSF1 RETREG1

Mac_CSF1 REN

Mac_CSF1 RELL1

Mac_CSF1 RCBTB2

Mac_CSF1 RCBTB1

Mac_CSF1 RASSF4

Mac_CSF1 RASSF2

Mac_CSF1 RARRES1

Mac_CSF1 RAPH1

Mac_CSF1 RAP2B

Mac_CSF1 RAP1A

Mac_CSF1 RAC2

Mac_CSF1 RAB31

Mac_CSF1 RAB11FIP1

Mac_CSF1 QKI

Mac_CSF1 PYGL

Mac_CSF1 PTTG1

Mac_CSF1 PTPRE

Mac_CSF1 PTPRC

Mac_CSF1 PTPN21

Mac_CSF1 PSMD1

Mac_CSF1 PRRG1

Mac_CSF1 PRPF38B

Mac_CSF1 PRKAG2

Mac_CSF1 PRIMA1

Mac_CSF1 PRDX1

Mac_CSF1 PPP1CC

Mac_CSF1 PNPO

Mac_CSF1 PMP22

Mac_CSF1 PLXNB1

Mac_CSF1 PLXDC2

Mac_CSF1 PLTP

Mac_CSF1 PLN

Mac_CSF1 PLEKHF2

Mac_CSF1 PLEK

Mac_CSF1 PLCG2

Mac_CSF1 PLCB4

Mac_CSF1 PLBD1

Mac_CSF1 PLAU

Mac_CSF1 PLA2G2A

Mac_CSF1 PKIB

Mac_CSF1 PIK3R5

Mac_CSF1 PIK3IP1

Mac_CSF1 PIK3CG

Mac_CSF1 PIK3CD

Mac_CSF1 PIK3AP1

Mac_CSF1 PICALM

Mac_CSF1 PGM5

Mac_CSF1 PFKFB3

Mac_CSF1 PFKFB2

Mac_CSF1 PELI1

Mac_CSF1 PECAM1

Mac_CSF1 PCSK6

Mac_CSF1 PCOLCE2

Mac_CSF1 PBRM1

Mac_CSF1 PARP9

Mac_CSF1 PARP8

Mac_CSF1 PARP14

Mac_CSF1 PAQR8

Mac_CSF1 PAPLN

Mac_CSF1 PAK1

Mac_CSF1 PAG1

Mac_CSF1 OTULINL

Mac_CSF1 OSTF1

Mac_CSF1 OSGEP

Mac_CSF1 OSBPL3

Mac_CSF1 OSBPL11

Mac_CSF1 OAS3

Mac_CSF1 OAS2

Mac_CSF1 NTN4

Mac_CSF1 NRIP1

Mac_CSF1 NPL

Mac_CSF1 NPC2

Mac_CSF1 NLN

Mac_CSF1 NFATC2

Mac_CSF1 NFATC1

Mac_CSF1 NEAT1

Mac_CSF1 NDST1

Mac_CSF1 NCOA4

Mac_CSF1 NCKAP1L

Mac_CSF1 NCF2

Mac_CSF1 NCEH1

Mac_CSF1 NAIP

Mac_CSF1 NADK

Mac_CSF1 NAAA

Mac_CSF1 MYO5A

Mac_CSF1 MYO1F

Mac_CSF1 MTUS1

Mac_CSF1 MTSS1

Mac_CSF1 MTM1

Mac_CSF1 MTAP

Mac_CSF1 MSN

Mac_CSF1 MRPL19

Mac_CSF1 MRC1

Mac_CSF1 MPP1

Mac_CSF1 MPDU1

Mac_CSF1 MNDA

Mac_CSF1 MMP9

Mac_CSF1 MMP14

Mac_CSF1 MGAT4A

Mac_CSF1 MFSD1

Mac_CSF1 MFNG

Mac_CSF1 MERTK

Mac_CSF1 MEOX1

Mac_CSF1 MEF2C

Mac_CSF1 MEF2A

Mac_CSF1 ME2

Mac_CSF1 ME1

Mac_CSF1 MCAM

Mac_CSF1 MBOAT1

Mac_CSF1 MBNL1

Mac_CSF1 MAT2A

Mac_CSF1 MAPKAPK3

Mac_CSF1 MAP3K5

Mac_CSF1 MAP3K1

Mac_CSF1 MAP2K6

Mac_CSF1 MANBA

Mac_CSF1 MAF

Mac_CSF1 M6PR

Mac_CSF1 LYZ

Mac_CSF1 LYN

Mac_CSF1 LY86

Mac_CSF1 LTBR

Mac_CSF1 LST1

Mac_CSF1 LRRFIP1

Mac_CSF1 LRRC8C

Mac_CSF1 LRMP

Mac_CSF1 LRCH1

Mac_CSF1 LPL

Mac_CSF1 LPCAT2

Mac_CSF1 LPAR6

Mac_CSF1 LMO2

Mac_CSF1 LMBRD1

Mac_CSF1 LITAF

Mac_CSF1 LIPA

Mac_CSF1 LILRB4

Mac_CSF1 LHFPL2

Mac_CSF1 LGALS9

Mac_CSF1 LDLRAD4

Mac_CSF1 LCP2

Mac_CSF1 LCP1

Mac_CSF1 LAT2

Mac_CSF1 LAPTM5

Mac_CSF1 LAP3

Mac_CSF1 LAIR1

Mac_CSF1 LACTB

Mac_CSF1 KYNU

Mac_CSF1 KRR1

Mac_CSF1 KIF21B

Mac_CSF1 KIAA1551

Mac_CSF1 KIAA1147

Mac_CSF1 KIAA0258

Mac_CSF1 KIAA0040

Mac_CSF1 KCNMB1

Mac_CSF1 KANSL1L

Mac_CSF1 JDP2

Mac_CSF1 ITGB8

Mac_CSF1 ITGB2

Mac_CSF1 ITGAX

Mac_CSF1 ITGAM

Mac_CSF1 ITGA6

Mac_CSF1 ITGA4

Mac_CSF1 IRF2

Mac_CSF1 IQGAP2

Mac_CSF1 IQGAP1

Mac_CSF1 IL7R

Mac_CSF1 IL2RG

Mac_CSF1 IL23A

Mac_CSF1 IL18

Mac_CSF1 IL13RA1

Mac_CSF1 IL10RA

Mac_CSF1 IKZF1

Mac_CSF1 IFNGR1

Mac_CSF1 IFIT1

Mac_CSF1 IFI30

Mac_CSF1 IFI16

Mac_CSF1 ID2

Mac_CSF1 ICAM1

Mac_CSF1 ICA1

Mac_CSF1 HTR2B

Mac_CSF1 HSPBAP1

Mac_CSF1 HSD17B11

Mac_CSF1 HRCT1

Mac_CSF1 HPGDS

Mac_CSF1 HPCAL1

Mac_CSF1 HLA-DRB4

Mac_CSF1 HLA-DRB1

Mac_CSF1 HLA-DQB1

Mac_CSF1 HLA-DQA1

Mac_CSF1 HLA-DOA

Mac_CSF1 HLA-DMA

Mac_CSF1 HK2

Mac_CSF1 HIF1A

Mac_CSF1 HHEX

Mac_CSF1 HDGFL3

Mac_CSF1 HCP5

Mac_CSF1 HCLS1

Mac_CSF1 HCK

Mac_CSF1 HACD1

Mac_CSF1 H2AFY

Mac_CSF1 H2AFV

Mac_CSF1 GSDME

Mac_CSF1 GSAP

Mac_CSF1 GRK3

Mac_CSF1 GRB2

Mac_CSF1 GPX1

Mac_CSF1 GPSM3

Mac_CSF1 GPR34

Mac_CSF1 GPR137B

Mac_CSF1 GPNMB

Mac_CSF1 GNPTAB

Mac_CSF1 GNA13

Mac_CSF1 GLUL

Mac_CSF1 GLUD1

Mac_CSF1 GLRX

Mac_CSF1 GLIPR1

Mac_CSF1 GLA

Mac_CSF1 GJB2

Mac_CSF1 GIMAP4

Mac_CSF1 GIMAP2

Mac_CSF1 GFRA3

Mac_CSF1 GFPT2

Mac_CSF1 GDE1

Mac_CSF1 GCA

Mac_CSF1 GATM

Mac_CSF1 GAPT

Mac_CSF1 GAP43

Mac_CSF1 GAB3

Mac_CSF1 FYB1

Mac_CSF1 FUCA1

Mac_CSF1 FTL

Mac_CSF1 FRMD4B

Mac_CSF1 FRMD4A

Mac_CSF1 FPR3

Mac_CSF1 FPGS

Mac_CSF1 FOXN2

Mac_CSF1 FOXK2

Mac_CSF1 FNBP1

Mac_CSF1 FMNL1

Mac_CSF1 FLVCR2

Mac_CSF1 FLNA

Mac_CSF1 FLI1

Mac_CSF1 FILIP1L

Mac_CSF1 FGR

Mac_CSF1 FGL2

Mac_CSF1 FGD4

Mac_CSF1 FCHO2

Mac_CSF1 FCGR3A

Mac_CSF1 FCGR2B

Mac_CSF1 FCGR2A

Mac_CSF1 FCGR1A

Mac_CSF1 FCER1G

Mac_CSF1 FBL

Mac_CSF1 FAM49B

Mac_CSF1 FAM129A

Mac_CSF1 FAM111A

Mac_CSF1 FAM107B

Mac_CSF1 FABP3

Mac_CSF1 F5

Mac_CSF1 F13A1

Mac_CSF1 EXT1

Mac_CSF1 EVI2B

Mac_CSF1 EVI2A

Mac_CSF1 ETV5

Mac_CSF1 ETV2

Mac_CSF1 EPS8

Mac_CSF1 ENTPD1

Mac_CSF1 ENPP7

Mac_CSF1 ENPP4

Mac_CSF1 EMB

Mac_CSF1 ELMO1

Mac_CSF1 EIF4A1

Mac_CSF1 ECHS1

Mac_CSF1 DUSP6

Mac_CSF1 DSC2

Mac_CSF1 DRAM2

Mac_CSF1 DPYSL2

Mac_CSF1 DOCK4

Mac_CSF1 DOCK2

Mac_CSF1 DOCK11

Mac_CSF1 DOCK10

Mac_CSF1 DMP1

Mac_CSF1 DISP1

Mac_CSF1 DISC1

Mac_CSF1 DIRC2

Mac_CSF1 DENND4B

Mac_CSF1 DEDD2

Mac_CSF1 DDX60L

Mac_CSF1 DDX58

Mac_CSF1 DCK

Mac_CSF1 DBNDD2

Mac_CSF1 DBI

Mac_CSF1 DAPK1

Mac_CSF1 DAB2

Mac_CSF1 CYTIP

Mac_CSF1 CYP51A1

Mac_CSF1 CYP2S1

Mac_CSF1 CYP1B1

Mac_CSF1 CYBB

Mac_CSF1 CXCR4

Mac_CSF1 CXCL16

Mac_CSF1 CX3CR1

Mac_CSF1 CTSS

Mac_CSF1 CTSL

Mac_CSF1 CTSD

Mac_CSF1 CTSC

Mac_CSF1 CST6

Mac_CSF1 CST3

Mac_CSF1 CSNK1G1

Mac_CSF1 CSNK1A1

Mac_CSF1 CSGALNACT1

Mac_CSF1 CSF3R

Mac_CSF1 CSF2RB

Mac_CSF1 CSF1R

Mac_CSF1 CRYL1

Mac_CSF1 CRIP1

Mac_CSF1 CREG1

Mac_CSF1 CREBL2

Mac_CSF1 CREB5

Mac_CSF1 CPVL

Mac_CSF1 CPM

Mac_CSF1 CPEB4

Mac_CSF1 COX7B

Mac_CSF1 COTL1

Mac_CSF1 CORO1A

Mac_CSF1 COPB2

Mac_CSF1 CMBL

Mac_CSF1 CMAHP

Mac_CSF1 CLDN7

Mac_CSF1 CKLF

Mac_CSF1 CKB

Mac_CSF1 CHKA

Mac_CSF1 CHD7

Mac_CSF1 CEP350

Mac_CSF1 CEP170

Mac_CSF1 CELF2

Mac_CSF1 CDK7

Mac_CSF1 CDH13

Mac_CSF1 CD9

Mac_CSF1 CD8A

Mac_CSF1 CD86

Mac_CSF1 CD84

Mac_CSF1 CD68

Mac_CSF1 CD58

Mac_CSF1 CD55

Mac_CSF1 CD53

Mac_CSF1 CD52

Mac_CSF1 CD48

Mac_CSF1 CD46

Mac_CSF1 CD44

Mac_CSF1 CD4

Mac_CSF1 CD37

Mac_CSF1 CD33

Mac_CSF1 CD302

Mac_CSF1 CD163L1

Mac_CSF1 CD163

Mac_CSF1 CCRL2

Mac_CSF1 CCL5

Mac_CSF1 CCL4L1

Mac_CSF1 CCL2

Mac_CSF1 CCDC88A

Mac_CSF1 CCDC112

Mac_CSF1 CBX4

Mac_CSF1 CBL

Mac_CSF1 CAST

Mac_CSF1 CASP5

Mac_CSF1 CASP4

Mac_CSF1 CASP1

Mac_CSF1 CARD8

Mac_CSF1 CAPZB

Mac_CSF1 CAPZA1

Mac_CSF1 CAPG

Mac_CSF1 CAP1

Mac_CSF1 CAB39

Mac_CSF1 CA2

Mac_CSF1 CA12

Mac_CSF1 C6orf62

Mac_CSF1 C3AR1

Mac_CSF1 C1QL2

Mac_CSF1 C1orf54

Mac_CSF1 C1GALT1

Mac_CSF1 BTK

Mac_CSF1 BMP2K

Mac_CSF1 BEST1

Mac_CSF1 B3GNT5

Mac_CSF1 B3GNT2

Mac_CSF1 B2M

Mac_CSF1 ATP6V1E1

Mac_CSF1 ATP6V1B2

Mac_CSF1 ATP6V1A

Mac_CSF1 ATP6AP2

Mac_CSF1 ATP1B1

Mac_CSF1 ATF6

Mac_CSF1 ASAP2

Mac_CSF1 ASAH1

Mac_CSF1 ARSD

Mac_CSF1 ARRB2

Mac_CSF1 ARPC5

Mac_CSF1 ARPC3

Mac_CSF1 ARPC2

Mac_CSF1 ARNTL

Mac_CSF1 ARMT1

Mac_CSF1 ARL6IP5

Mac_CSF1 ARHGEF6

Mac_CSF1 ARHGEF37

Mac_CSF1 ARHGEF3

Mac_CSF1 ARHGDIB

Mac_CSF1 ARHGAP45

Mac_CSF1 ARHGAP26

Mac_CSF1 ARHGAP24

Mac_CSF1 ARHGAP22

Mac_CSF1 ARHGAP18

Mac_CSF1 ARHGAP15

Mac_CSF1 APOE

Mac_CSF1 APOC2

Mac_CSF1 APOC1

Mac_CSF1 APCS

Mac_CSF1 AP1S2

Mac_CSF1 AP1B1

Mac_CSF1 ANXA2

Mac_CSF1 ANKRD44

Mac_CSF1 ANKH

Mac_CSF1 ANK2

Mac_CSF1 AMPD3

Mac_CSF1 AMD1

Mac_CSF1 ALOX5AP

Mac_CSF1 ALDH3B1

Mac_CSF1 ALDH2

Mac_CSF1 ALDH1A3

Mac_CSF1 AKR1C3

Mac_CSF1 AKAP6

Mac_CSF1 AK2

Mac_CSF1 AIF1

Mac_CSF1 AGO4

Mac_CSF1 ADSSL1

Mac_CSF1 ADRB2

Mac_CSF1 ADPGK

Mac_CSF1 ADIPOR1

Mac_CSF1 ADGRG6

Mac_CSF1 ADGRE5

Mac_CSF1 ADCY7

Mac_CSF1 ADAM9

Mac_CSF1 ADA2

Mac_CSF1 ACSL5

Mac_CSF1 ACSL1

Mac_CSF1 ACKR1

Mac_CSF1 ACER3

Mac_CSF1 ABRACL

Mac_CSF1 ABCG1

Mac_CSF1 ABCC3

Mac_CSF1 ABCA1

MacTh1_cluster WIPF1

MacTh1_cluster WDFY4

MacTh1_cluster WAS

MacTh1_cluster TNFRSF1B

MacTh1_cluster TNFAIP8L2

MacTh1_cluster TMC8

MacTh1_cluster TLR8

MacTh1_cluster TFEC

MacTh1_cluster TAGAP

MacTh1_cluster SRGN

MacTh1_cluster SPN

MacTh1_cluster SPI1

MacTh1_cluster SNX20

MacTh1_cluster SLCO2B1

MacTh1_cluster SLC7A7

MacTh1_cluster SLA

MacTh1_cluster SIGLEC9

MacTh1_cluster SIGLEC7

MacTh1_cluster SIGLEC10

MacTh1_cluster SELPLG

MacTh1_cluster SCIMP

MacTh1_cluster SASH3

MacTh1_cluster SAMSN1

MacTh1_cluster RNASE6

MacTh1_cluster RASAL3

MacTh1_cluster PTPRC

MacTh1_cluster PTPN22

MacTh1_cluster PLEK

MacTh1_cluster PLCB2

MacTh1_cluster PIK3R5

MacTh1_cluster PIK3CG

MacTh1_cluster PARVG

MacTh1_cluster NCKAP1L

MacTh1_cluster NCF4

MacTh1_cluster MYO1F

MacTh1_cluster MS4A6A

MacTh1_cluster MPEG1

MacTh1_cluster MNDA

MacTh1_cluster LST1

MacTh1_cluster LRRC25

MacTh1_cluster LILRB4

MacTh1_cluster LILRB2

MacTh1_cluster LILRB1

MacTh1_cluster LCP2

MacTh1_cluster LAPTM5

MacTh1_cluster LAIR1

MacTh1_cluster KLHL6

MacTh1_cluster ITGB2

MacTh1_cluster ITGAX

MacTh1_cluster IRF8

MacTh1_cluster INPP5D

MacTh1_cluster IL12RB1

MacTh1_cluster IL10RA

MacTh1_cluster IKZF1

MacTh1_cluster IGSF6

MacTh1_cluster HLA-DRA

MacTh1_cluster HLA-DPB1

MacTh1_cluster HLA-DPA1

MacTh1_cluster HLA-DOA

MacTh1_cluster HLA-DMB

MacTh1_cluster HLA-DMA

MacTh1_cluster HCLS1

MacTh1_cluster HAVCR2

MacTh1_cluster GPR65

MacTh1_cluster GAB3

MacTh1_cluster FYB1

MacTh1_cluster FMNL1

MacTh1_cluster FGL2

MacTh1_cluster FGD2

MacTh1_cluster FERMT3

MacTh1_cluster FCER1G

MacTh1_cluster FAM78A

MacTh1_cluster EVI2B

MacTh1_cluster EVI2A

MacTh1_cluster DOCK2

MacTh1_cluster CYTH4

MacTh1_cluster CYBB

MacTh1_cluster CXorf21

MacTh1_cluster CTSS

MacTh1_cluster CSF1R

MacTh1_cluster CLEC4A

MacTh1_cluster CD86

MacTh1_cluster CD84

MacTh1_cluster CD74

MacTh1_cluster CD68

MacTh1_cluster CD53

MacTh1_cluster CD4

MacTh1_cluster CD37

MacTh1_cluster CD33

MacTh1_cluster CD300LF

MacTh1_cluster CCR5

MacTh1_cluster CARD11

MacTh1_cluster C3AR1

MacTh1_cluster C1QC

MacTh1_cluster C1QB

MacTh1_cluster C1QA

MacTh1_cluster BTK

MacTh1_cluster BIN2

MacTh1_cluster ARHGAP9

MacTh1_cluster ARHGAP30

MacTh1_cluster ARHGAP25

MacTh1_cluster APBB1IP

MacTh1_cluster AOAH

MacTh1_cluster AIF1

MacTh1_cluster ADAP2

T_Cell ZAP70

T_Cell WWP1

T_Cell WNT10B

T_Cell VIPR1

T_Cell UPP1

T_Cell TXK

T_Cell TSPAN14

T_Cell TSEN54

T_Cell TRERF1

T_Cell TRB

T_Cell TRAT1

T_Cell TRA

T_Cell TOB1

T_Cell TNIK

T_Cell TNFSF8

T_Cell TNFRSF25

T_Cell TNFAIP3

T_Cell TMC6

T_Cell TIAM1

T_Cell TESPA1

T_Cell TCF7

T_Cell TARP

T_Cell TACC3

T_Cell SYT1

T_Cell SYNE2

T_Cell STAT4

T_Cell SPOCK2

T_Cell SPEG

T_Cell SORL1

T_Cell SOCS3

T_Cell SNPH

T_Cell SLFN5

T_Cell SLCO3A1

T_Cell SLC39A8

T_Cell SLC35D2

T_Cell SH2D1A

T_Cell SEMA4D

T_Cell SEM1

T_Cell SELPLG

T_Cell SATB1

T_Cell S100A8

T_Cell S100A10

T_Cell RUNX2

T_Cell RTKN2

T_Cell RORA

T_Cell RNF213

T_Cell RNF144A

T_Cell RGS10

T_Cell RETREG1

T_Cell RBMS1

T_Cell RASGRP1

T_Cell RARRES3

T_Cell RAB43

T_Cell PXN

T_Cell PTGER2

T_Cell PRKCQ

T_Cell PRKCI

T_Cell PRKCA

T_Cell PLXDC1

T_Cell PKM

T_Cell PIM1

T_Cell PIK3R1

T_Cell PIK3IP1

T_Cell PDE9A

T_Cell PDE4D

T_Cell PCYT2

T_Cell PCSK5

T_Cell PAG1

T_Cell OPTN

T_Cell OLAH

T_Cell NSG1

T_Cell NR4A2

T_Cell NPTXR

T_Cell NPDC1

T_Cell NOL4L

T_Cell NELL2

T_Cell NBPF14

T_Cell NAP1L5

T_Cell MYBL1

T_Cell MPP7

T_Cell MORC2-AS1

T_Cell MLLT3

T_Cell MEN1

T_Cell MATN2

T_Cell MAST4

T_Cell MAPKAPK5

T_Cell MAP7D1

T_Cell MAN1C1

T_Cell MAL

T_Cell LYAR

T_Cell LTBP4

T_Cell LRIG1

T_Cell LPIN2

T_Cell LPAR6

T_Cell LPAR2

T_Cell LINS1

T_Cell LIMA1

T_Cell LEPROTL1

T_Cell LEF1

T_Cell LDHB

T_Cell LDHA

T_Cell LCP2

T_Cell LCK

T_Cell LAT

T_Cell KLRK1

T_Cell KLRG1

T_Cell KLRB1

T_Cell JAKMIP1

T_Cell ITPKB

T_Cell ITM2A

T_Cell ITK

T_Cell ITGA6

T_Cell INPP4B

T_Cell INPP4A

T_Cell IL7R

T_Cell IL6ST

T_Cell IL6R

T_Cell IL32

T_Cell IL18R1

T_Cell IFITM1

T_Cell ID2

T_Cell HSPA1L

T_Cell HOXB2

T_Cell GZMK

T_Cell GPSM3

T_Cell GIPC1

T_Cell GIMAP4

T_Cell GIMAP2

T_Cell GFI1

T_Cell GBP2

T_Cell GBP1

T_Cell GATA3

T_Cell GALT

T_Cell GABARAPL1

T_Cell FYN

T_Cell FYB1

T_Cell FLT3LG

T_Cell FHIT

T_Cell FBLN5

T_Cell FAM102A

T_Cell DUSP2

T_Cell DUSP16

T_Cell DPP4

T_Cell DOCK9

T_Cell DNASE1L3

T_Cell DNAJC17

T_Cell DNAJB1

T_Cell CTSW

T_Cell COA5

T_Cell CISH

T_Cell CEP41

T_Cell CDR2

T_Cell CDC25B

T_Cell CDC14A

T_Cell CD96

T_Cell CD6

T_Cell CD5

T_Cell CD3G

T_Cell CD3E

T_Cell CD3D

T_Cell CD28

T_Cell CD247

T_Cell CD2

T_Cell CCND2

T_Cell CCL5

T_Cell CAMK4

T_Cell BUB1B

T_Cell BIN2

T_Cell BEX3

T_Cell BCL11B

T_Cell BAG3

T_Cell ATP1A1

T_Cell ARL4C

T_Cell ARID3A

T_Cell AQP3

T_Cell APOE

T_Cell APBA2

T_Cell ANXA1

T_Cell AKTIP

T_Cell ADA

T_Cell ACVR2B

T_Cell ACTN1

T_Cell AAK1

T_Cell_cluster ZNF831

T_Cell_cluster ZAP70

T_Cell_cluster UBASH3A

T_Cell_cluster TRAT1

T_Cell_cluster TIGIT

T_Cell_cluster TIFAB

T_Cell_cluster THEMIS

T_Cell_cluster TESPA1

T_Cell_cluster TBX21

T_Cell_cluster TBC1D10C

T_Cell_cluster SPOCK2

T_Cell_cluster SP140

T_Cell_cluster SLAMF7

T_Cell_cluster SLAMF6

T_Cell_cluster SLAMF1

T_Cell_cluster SLA2

T_Cell_cluster SIT1

T_Cell_cluster SIRPG

T_Cell_cluster SH2D1A

T_Cell_cluster SCML4

T_Cell_cluster SAMD3

T_Cell_cluster S1PR4

T_Cell_cluster PYHIN1

T_Cell_cluster PTPRCAP

T_Cell_cluster PTPN7

T_Cell_cluster PSTPIP1

T_Cell_cluster PRKCB

T_Cell_cluster PRF1

T_Cell_cluster PLA2G2D

T_Cell_cluster PDCD1

T_Cell_cluster NKG7

T_Cell_cluster MAP4K1

T_Cell_cluster LY9

T_Cell_cluster LTA

T_Cell_cluster LINC00426

T_Cell_cluster LCK

T_Cell_cluster LAX1

T_Cell_cluster LAT

T_Cell_cluster KLRK1

T_Cell_cluster JAK3

T_Cell_cluster ITK

T_Cell_cluster IRF4

T_Cell_cluster IL2RG

T_Cell_cluster ICOS

T_Cell_cluster GZMM

T_Cell_cluster GZMK

T_Cell_cluster GZMA

T_Cell_cluster GVINP1

T_Cell_cluster GPR174

T_Cell_cluster GPR171

T_Cell_cluster GFI1

T_Cell_cluster FCRL3

T_Cell_cluster FASLG

T_Cell_cluster EOMES

T_Cell_cluster CXCR6

T_Cell_cluster CXCR3

T_Cell_cluster CXCL9

T_Cell_cluster CTLA4

T_Cell_cluster CST7

T_Cell_cluster CRTAM

T_Cell_cluster CORO1A

T_Cell_cluster CD96

T_Cell_cluster CD8A

T_Cell_cluster CD7

T_Cell_cluster CD6

T_Cell_cluster CD52

T_Cell_cluster CD5

T_Cell_cluster CD48

T_Cell_cluster CD40LG

T_Cell_cluster CD3G

T_Cell_cluster CD3E

T_Cell_cluster CD3D

T_Cell_cluster CD38

T_Cell_cluster CD27

T_Cell_cluster CD247

T_Cell_cluster CD2

T_Cell_cluster CCR2

T_Cell_cluster CCL5

T_Cell_cluster C5orf20

T_Cell_cluster C16orf54

T_Cell_cluster BTLA

T_Cell_cluster AMICA1

T_Cell_cluster ACAP1

TNBC_B_Cell IGLL1

TNBC_B_Cell IGLJ3

TNBC_B_Cell IGLC2

TNBC_B_Cell IGL

TNBC_B_Cell IGKV1D-13

TNBC_B_Cell IGKC

TNBC_B_Cell IGHM

TNBC_B_Cell IGHG3

TNBC_B_Cell IGHG1

TNBC_B_Cell IGHD

TNBC_B_Cell IGH

TNBC_T_Cell TRBC1

TNBC_T_Cell TRA@

TNBC_T_Cell TNFRSF7

TNBC_T_Cell SELL

TNBC_T_Cell SAMSN1

TNBC_T_Cell PTPRC

TNBC_T_Cell PLAC8

TNBC_T_Cell LCK

TNBC_T_Cell LAPTM5

TNBC_T_Cell KIAA0053

TNBC_T_Cell ITK

TNBC_T_Cell IL10RA

TNBC_T_Cell GZMK

TNBC_T_Cell EVI2B

TNBC_T_Cell CORO1A

TNBC_T_Cell CD53

TNBC_T_Cell CD52

TNBC_T_Cell CD48

TNBC_T_Cell CD3D

TNBC_T_Cell CD2

B_cells_naive IGKC

B_cells_naive IGHM

B_cells_naive MS4A1

B_cells_naive CD37

B_cells_naive CD79A

B_cells_naive TCL1A

B_cells_naive FCMR

B_cells_naive SELL

B_cells_naive IL4R

B_cells_naive IGHD

B_cells_naive LTB

B_cells_naive P2RX5

B_cells_naive IRF8

B_cells_naive CD69

B_cells_naive GUSBP11

B_cells_naive BANK1

B_cells_naive CD19

B_cells_naive RUBCNL

B_cells_naive LY86

B_cells_naive IGLL3P

B_cells_naive HLA-DOB

B_cells_naive CD79B

B_cells_naive RASGRP2

B_cells_naive SIK1

B_cells_naive RIPOR2

B_cells_naive HHEX

B_cells_naive BACH2

B_cells_naive FCRL2

B_cells_naive PNOC

B_cells_naive PTPRCAP

B_cells_naive SPIB

B_cells_naive CCR7

B_cells_naive VPREB3

B_cells_naive STAP1

B_cells_naive FCGR2B

B_cells_naive CD22

B_cells_naive EAF2

B_cells_naive GPR18

B_cells_naive BRAF

B_cells_naive PIK3IP1

B_cells_naive IL2RB

B_cells_naive LRMP

B_cells_naive ADAM28

B_cells_naive TRIB2

B_cells_naive ALOX5

B_cells_naive RNASE6

B_cells_naive BLK

B_cells_naive BIRC3

B_cells_naive CCL4

B_cells_naive P2RY14

B_cells_naive NPIPB15

B_cells_naive RALGPS2

B_cells_naive NIPSNAP3B

B_cells_naive PSG2

B_cells_naive SPOCK2

B_cells_naive CD180

B_cells_naive CD40

B_cells_naive CR2

B_cells_naive CD72

B_cells_naive SKAP1

B_cells_naive CD1B

B_cells_naive KLRG1

B_cells_naive CD1C

B_cells_naive CD1A

B_cells_naive APOBEC3G

B_cells_naive CD3E

B_cells_naive MS4A6A

B_cells_naive TPSAB1

B_cells_naive BCL7A

B_cells_naive CLEC2D

B_cells_memory IGKC

B_cells_memory MS4A1

B_cells_memory IGHM

B_cells_memory LTB

B_cells_memory CD37

B_cells_memory GUSBP11

B_cells_memory CD79A

B_cells_memory BANK1

B_cells_memory P2RX5

B_cells_memory IGLL3P

B_cells_memory FCMR

B_cells_memory SELL

B_cells_memory IRF8

B_cells_memory LY86

B_cells_memory SIK1

B_cells_memory SPIB

B_cells_memory CD19

B_cells_memory RIPOR2

B_cells_memory RUBCNL

B_cells_memory RASGRP2

B_cells_memory CD69

B_cells_memory HLA-DOB

B_cells_memory CD79B

B_cells_memory AIM2

B_cells_memory FCGR2B

B_cells_memory PNOC

B_cells_memory CD27

B_cells_memory PTPRCAP

B_cells_memory FCRL2

B_cells_memory BLK

B_cells_memory LRMP

B_cells_memory CD1C

B_cells_memory NPIPB15

B_cells_memory HHEX

B_cells_memory VPREB3

B_cells_memory IGHD

B_cells_memory ALOX5

B_cells_memory SP140

B_cells_memory GPR183

B_cells_memory CD180

B_cells_memory EAF2

B_cells_memory STAP1

B_cells_memory IL2RB

B_cells_memory CCR7

B_cells_memory ACP5

B_cells_memory IL4R

B_cells_memory GPR18

B_cells_memory CD22

B_cells_memory SIT1

B_cells_memory TNFRSF13B

B_cells_memory GNG7

B_cells_memory HLA-DQA1

B_cells_memory CD40

B_cells_memory RALGPS2

B_cells_memory BACH2

B_cells_memory PSG2

B_cells_memory RNASE6

B_cells_memory PVRIG

B_cells_memory ADAM28

B_cells_memory APOBEC3G

B_cells_memory PIK3IP1

B_cells_memory SPOCK2

B_cells_memory KLRG1

B_cells_memory BIRC3

B_cells_memory TRAC

Plasma_cells IGLL3P

Plasma_cells IGKC

Plasma_cells IGHM

Plasma_cells GUSBP11

Plasma_cells IGHD

Plasma_cells MZB1

Plasma_cells CD38

Plasma_cells TNFRSF17

Plasma_cells CD27

Plasma_cells SIK1

Plasma_cells EAF2

Plasma_cells IGHE

Plasma_cells HLA-DOB

Plasma_cells MAN1A1

Plasma_cells CD79A

Plasma_cells SPAG4

Plasma_cells RASGRP3

Plasma_cells PNOC

Plasma_cells AMPD1

Plasma_cells FOSB

Plasma_cells FCGR2B

Plasma_cells P2RX5

Plasma_cells RNASE6

Plasma_cells QPCT

Plasma_cells CHST15

Plasma_cells GNG7

Plasma_cells C11orf80

Plasma_cells ST6GALNAC4

Plasma_cells APOBEC3G

Plasma_cells CD19

Plasma_cells CCR2

Plasma_cells AIM2

Plasma_cells PDK1

Plasma_cells NPIPB15

Plasma_cells TNFRSF13B

Plasma_cells KCNA3

Plasma_cells CD3E

Plasma_cells RPL3P7

Plasma_cells CD180

Plasma_cells ACP5

Plasma_cells LRMP

Plasma_cells LIME1

Plasma_cells SLAMF1

Plasma_cells SP140

T_cells_CD8 IL7R

T_cells_CD8 CD3D

T_cells_CD8 TRAC

T_cells_CD8 CCL5

T_cells_CD8 CD8A

T_cells_CD8 LTB

T_cells_CD8 TRBC1

T_cells_CD8 KLRB1

T_cells_CD8 GZMK

T_cells_CD8 LCK

T_cells_CD8 NKG7

T_cells_CD8 GZMH

T_cells_CD8 KLRK1

T_cells_CD8 CD2

T_cells_CD8 PRF1

T_cells_CD8 SELL

T_cells_CD8 ITK

T_cells_CD8 GZMA

T_cells_CD8 CD3E

T_cells_CD8 FCMR

T_cells_CD8 CD247

T_cells_CD8 CD27

T_cells_CD8 PIK3IP1

T_cells_CD8 CST7

T_cells_CD8 CCR7

T_cells_CD8 CTSW

T_cells_CD8 CD37

T_cells_CD8 PTPRCAP

T_cells_CD8 BCL11B

T_cells_CD8 DUSP2

T_cells_CD8 IL2RB

T_cells_CD8 PVRIG

T_cells_CD8 KLRG1

T_cells_CD8 CD69

T_cells_CD8 GNLY

T_cells_CD8 CD8B

T_cells_CD8 RIPOR2

T_cells_CD8 CD3G

T_cells_CD8 SPOCK2

T_cells_CD8 ZAP70

T_cells_CD8 ZFP36L2

T_cells_CD8 LAT

T_cells_CD8 TCF7

T_cells_CD8 IGKC

T_cells_CD8 TRDC

T_cells_CD8 CCL4

T_cells_CD8 RASGRP2

T_cells_CD8 SKAP1

T_cells_CD8 PTGER2

T_cells_CD8 CD7

T_cells_CD8 DGKA

T_cells_CD8 APOBEC3G

T_cells_CD8 GZMB

T_cells_CD8 NCR3

T_cells_CD8 MAP4K1

T_cells_CD8 CD6

T_cells_CD8 CD96

T_cells_CD8 KLRD1

T_cells_CD8 TBX21

T_cells_CD8 PBXIP1

T_cells_CD8 GPR65

T_cells_CD8 CLEC2D

T_cells_CD8 SIT1

T_cells_CD8 CRTAM

T_cells_CD8 PASK

T_cells_CD8 LY9

T_cells_CD8 SIRPG

T_cells_CD8 RPL3P7

T_cells_CD8 LIME1

T_cells_CD8 FLT3LG

T_cells_CD8 SH2D1A

T_cells_CD8 SIK1

T_cells_CD8 APOL3

T_cells_CD8 LEF1

T_cells_CD8 CD160

T_cells_CD8 TRIB2

T_cells_CD8 LAIR2

T_cells_CD8 ACAP1

T_cells_CD8 GZMM

T_cells_CD8 GPR18

T_cells_CD4_naive IL7R

T_cells_CD4_naive CD3D

T_cells_CD4_naive TRAC

T_cells_CD4_naive LTB

T_cells_CD4_naive TRBC1

T_cells_CD4_naive SELL

T_cells_CD4_naive ITK

T_cells_CD4_naive LCK

T_cells_CD4_naive FCMR

T_cells_CD4_naive CCR7

T_cells_CD4_naive CD2

T_cells_CD4_naive BCL11B

T_cells_CD4_naive CD247

T_cells_CD4_naive LAT

T_cells_CD4_naive PGGHG

T_cells_CD4_naive TCF7

T_cells_CD4_naive PIK3IP1

T_cells_CD4_naive RPL3P7

T_cells_CD4_naive RIPOR2

T_cells_CD4_naive CD69

T_cells_CD4_naive ZAP70

T_cells_CD4_naive CD27

T_cells_CD4_naive RASGRP2

T_cells_CD4_naive CD3E

T_cells_CD4_naive DGKA

T_cells_CD4_naive CD37

T_cells_CD4_naive CD3G

T_cells_CD4_naive LEF1

T_cells_CD4_naive SKAP1

T_cells_CD4_naive PVRIG

T_cells_CD4_naive CD4

T_cells_CD4_naive FLT3LG

T_cells_CD4_naive MAP4K1

T_cells_CD4_naive TRAT1

T_cells_CD4_naive TRIB2

T_cells_CD4_naive UBASH3A

T_cells_CD4_naive IL4R

T_cells_CD4_naive SPOCK2

T_cells_CD4_naive CD7

T_cells_CD4_naive PTPRCAP

T_cells_CD4_naive ANKRD55

T_cells_CD4_naive LY9

T_cells_CD4_naive ACAP1

T_cells_CD4_naive CLEC2D

T_cells_CD4_naive CD96

T_cells_CD4_naive BACH2

T_cells_CD4_naive GZMK

T_cells_CD4_naive SIT1

T_cells_CD4_naive TXK

T_cells_CD4_naive GPR18

T_cells_CD4_naive DPEP2

T_cells_CD4_naive GZMM

T_cells_CD4_naive CD5

T_cells_CD4_naive SIRPG

T_cells_CD4_naive PBXIP1

T_cells_CD4_naive PASK

T_cells_CD4_naive ICOS

T_cells_CD4_memory_resting IL7R

T_cells_CD4_memory_resting TRAC

T_cells_CD4_memory_resting CD3D

T_cells_CD4_memory_resting LTB

T_cells_CD4_memory_resting TRBC1

T_cells_CD4_memory_resting KLRB1

T_cells_CD4_memory_resting CD2

T_cells_CD4_memory_resting LCK

T_cells_CD4_memory_resting ITK

T_cells_CD4_memory_resting SELL

T_cells_CD4_memory_resting CD3E

T_cells_CD4_memory_resting CD69

T_cells_CD4_memory_resting CD247

T_cells_CD4_memory_resting FCMR

T_cells_CD4_memory_resting BCL11B

T_cells_CD4_memory_resting PTPRCAP

T_cells_CD4_memory_resting PIK3IP1

T_cells_CD4_memory_resting CD37

T_cells_CD4_memory_resting PTGER2

T_cells_CD4_memory_resting CD27

T_cells_CD4_memory_resting GZMA

T_cells_CD4_memory_resting ZFP36L2

T_cells_CD4_memory_resting GZMK

T_cells_CD4_memory_resting CD4

T_cells_CD4_memory_resting RPL3P7

T_cells_CD4_memory_resting LAT

T_cells_CD4_memory_resting SPOCK2

T_cells_CD4_memory_resting TCF7

T_cells_CD4_memory_resting PBXIP1

T_cells_CD4_memory_resting CD6

T_cells_CD4_memory_resting CCL5

T_cells_CD4_memory_resting CD3G

T_cells_CD4_memory_resting GPR183

T_cells_CD4_memory_resting IL2RB

T_cells_CD4_memory_resting RASGRP2

T_cells_CD4_memory_resting RIPOR2

T_cells_CD4_memory_resting CCR7

T_cells_CD4_memory_resting DGKA

T_cells_CD4_memory_resting ZAP70

T_cells_CD4_memory_resting TRAT1

T_cells_CD4_memory_resting SKAP1

T_cells_CD4_memory_resting TRIB2

T_cells_CD4_memory_resting APOBEC3G

T_cells_CD4_memory_resting PASK

T_cells_CD4_memory_resting PRF1

T_cells_CD4_memory_resting DUSP2

T_cells_CD4_memory_resting KLRG1

T_cells_CD4_memory_resting ETS1

T_cells_CD4_memory_resting PVRIG

T_cells_CD4_memory_resting CD5

T_cells_CD4_memory_resting CLEC2D

T_cells_CD4_memory_resting SAMSN1

T_cells_CD4_memory_resting APOL3

T_cells_CD4_memory_resting SIT1

T_cells_CD4_memory_resting CD96

T_cells_CD4_memory_resting CD7

T_cells_CD4_memory_resting GPR65

T_cells_CD4_memory_resting RGS1

T_cells_CD4_memory_resting FLT3LG

T_cells_CD4_memory_resting CST7

T_cells_CD4_memory_resting MAP4K1

T_cells_CD4_memory_resting SH2D1A

T_cells_CD4_memory_resting LY9

T_cells_CD4_memory_resting LIME1

T_cells_CD4_memory_resting GNLY

T_cells_CD4_memory_resting CCND2

T_cells_CD4_memory_resting LEF1

T_cells_CD4_memory_resting IL4R

T_cells_CD4_memory_resting SIRPG

T_cells_CD4_memory_resting BIRC3

T_cells_CD4_memory_resting SLAMF1

T_cells_CD4_memory_resting DPP4

T_cells_CD4_memory_activated GZMB

T_cells_CD4_memory_activated CCL4

T_cells_CD4_memory_activated TRAC

T_cells_CD4_memory_activated CD2

T_cells_CD4_memory_activated IL2RB

T_cells_CD4_memory_activated CD3D

T_cells_CD4_memory_activated IL9

T_cells_CD4_memory_activated IFNG

T_cells_CD4_memory_activated LTB

T_cells_CD4_memory_activated IL2RA

T_cells_CD4_memory_activated LCK

T_cells_CD4_memory_activated ITK

T_cells_CD4_memory_activated AIM2

T_cells_CD4_memory_activated TRBC1

T_cells_CD4_memory_activated BIRC3

T_cells_CD4_memory_activated CCR7

T_cells_CD4_memory_activated LTA

T_cells_CD4_memory_activated SAMSN1

T_cells_CD4_memory_activated CD3E

T_cells_CD4_memory_activated CD6

T_cells_CD4_memory_activated GPR171

T_cells_CD4_memory_activated SELL

T_cells_CD4_memory_activated CCND2

T_cells_CD4_memory_activated DUSP2

T_cells_CD4_memory_activated CD28

T_cells_CD4_memory_activated GPR183

T_cells_CD4_memory_activated CD247

T_cells_CD4_memory_activated EGR2

T_cells_CD4_memory_activated SLAMF1

T_cells_CD4_memory_activated TNFRSF4

T_cells_CD4_memory_activated IL7R

T_cells_CD4_memory_activated CLEC2D

T_cells_CD4_memory_activated CTLA4

T_cells_CD4_memory_activated CCL5

T_cells_CD4_memory_activated ICOS

T_cells_CD4_memory_activated CD4

T_cells_CD4_memory_activated IL3

T_cells_CD4_memory_activated TRAT1

T_cells_CD4_memory_activated CCL20

T_cells_CD4_memory_activated APOBEC3G

T_cells_CD4_memory_activated PTPRCAP

T_cells_CD4_memory_activated SH2D1A

T_cells_CD4_memory_activated LAT

T_cells_CD4_memory_activated RGS1

T_cells_CD4_memory_activated PRF1

T_cells_CD4_memory_activated CSF2

T_cells_CD4_memory_activated CD7

T_cells_CD4_memory_activated ETS1

T_cells_CD4_memory_activated MAP4K1

T_cells_CD4_memory_activated BCL2A1

T_cells_CD4_memory_activated GZMA

T_cells_CD4_memory_activated TBX21

T_cells_CD4_memory_activated CD3G

T_cells_CD4_memory_activated FCMR

T_cells_CD4_memory_activated CXCR6

T_cells_CD4_memory_activated CD27

T_cells_CD4_memory_activated APOL3

T_cells_CD4_memory_activated CD69

T_cells_CD4_memory_activated RPL3P7

T_cells_CD4_memory_activated PTGER2

T_cells_CD4_memory_activated SOCS1

T_cells_CD4_memory_activated SPOCK2

T_cells_follicular_helper TRAC

T_cells_follicular_helper CD3D

T_cells_follicular_helper TRBC1

T_cells_follicular_helper CD2

T_cells_follicular_helper LTB

T_cells_follicular_helper LCK

T_cells_follicular_helper CXCL13

T_cells_follicular_helper ITK

T_cells_follicular_helper LAT

T_cells_follicular_helper ICOS

T_cells_follicular_helper PASK

T_cells_follicular_helper FCMR

T_cells_follicular_helper ZAP70

T_cells_follicular_helper IL2RB

T_cells_follicular_helper CD69

T_cells_follicular_helper TRIB2

T_cells_follicular_helper FOSB

T_cells_follicular_helper CD27

T_cells_follicular_helper PTPRCAP

T_cells_follicular_helper CD247

T_cells_follicular_helper SIK1

T_cells_follicular_helper CHI3L2

T_cells_follicular_helper TCF7

T_cells_follicular_helper KLRB1

T_cells_follicular_helper CD4

T_cells_follicular_helper MAP4K1

T_cells_follicular_helper SELL

T_cells_follicular_helper PVRIG

T_cells_follicular_helper DGKA

T_cells_follicular_helper CD37

T_cells_follicular_helper BCL11B

T_cells_follicular_helper SPOCK2

T_cells_follicular_helper SIRPG

T_cells_follicular_helper IL7R

T_cells_follicular_helper CD3E

T_cells_follicular_helper SH2D1A

T_cells_follicular_helper ST8SIA1

T_cells_follicular_helper P2RX5

T_cells_follicular_helper PDCD1

T_cells_follicular_helper CXCR5

T_cells_follicular_helper ICA1

T_cells_follicular_helper CD3G

T_cells_follicular_helper SKAP1

T_cells_follicular_helper PIK3IP1

T_cells_follicular_helper DUSP2

T_cells_follicular_helper RPL3P7

T_cells_follicular_helper CD7

T_cells_follicular_helper IGHM

T_cells_follicular_helper CTLA4

T_cells_follicular_helper LRMP

T_cells_follicular_helper RGS1

T_cells_follicular_helper CCR7

T_cells_follicular_helper CD6

T_cells_follicular_helper MYB

T_cells_follicular_helper PGGHG

T_cells_follicular_helper CLEC2D

T_cells_follicular_helper SIT1

T_cells_follicular_helper EGR2

T_cells_follicular_helper PBXIP1

T_cells_follicular_helper GPR18

T_cells_follicular_helper IL21

T_cells_follicular_helper CD5

T_cells_follicular_helper TRAT1

T_cells_follicular_helper CD28

T_cells_follicular_helper LAG3

T_cells_follicular_helper ZBTB10

T_cells_follicular_helper TNFRSF4

T_cells_follicular_helper CD38

T_cells_follicular_helper AIM2

T_cells_follicular_helper ACAP1

T_cells_follicular_helper ANKRD55

T_cells_follicular_helper SLAMF1

T_cells_follicular_helper GZMM

T_cells_regulatory__Tregs TRBC1

T_cells_regulatory__Tregs TRAC

T_cells_regulatory__Tregs LTB

T_cells_regulatory__Tregs CD3D

T_cells_regulatory__Tregs IL2RB

T_cells_regulatory__Tregs CD2

T_cells_regulatory__Tregs CD247

T_cells_regulatory__Tregs PMCH

T_cells_regulatory__Tregs CD7

T_cells_regulatory__Tregs ITK

T_cells_regulatory__Tregs CD27

T_cells_regulatory__Tregs GPR171

T_cells_regulatory__Tregs LCK

T_cells_regulatory__Tregs CCR7

T_cells_regulatory__Tregs SKAP1

T_cells_regulatory__Tregs CD4

T_cells_regulatory__Tregs CD3E

T_cells_regulatory__Tregs SELL

T_cells_regulatory__Tregs CD37

T_cells_regulatory__Tregs PIK3IP1

T_cells_regulatory__Tregs FCMR

T_cells_regulatory__Tregs SPOCK2

T_cells_regulatory__Tregs SIT1

T_cells_regulatory__Tregs CTLA4

T_cells_regulatory__Tregs IL2RA

T_cells_regulatory__Tregs CD5

T_cells_regulatory__Tregs PTPRCAP

T_cells_regulatory__Tregs IFI44L

T_cells_regulatory__Tregs LAT

T_cells_regulatory__Tregs IL4R

T_cells_regulatory__Tregs PBXIP1

T_cells_regulatory__Tregs LAIR2

T_cells_regulatory__Tregs DGKA

T_cells_regulatory__Tregs ZFP36L2

T_cells_regulatory__Tregs CD96

T_cells_regulatory__Tregs TNFRSF4

T_cells_regulatory__Tregs CLEC2D

T_cells_regulatory__Tregs TRAV21

T_cells_regulatory__Tregs BCL11B

T_cells_regulatory__Tregs PTGER2

T_cells_regulatory__Tregs PVRIG

T_cells_regulatory__Tregs BIRC3

T_cells_regulatory__Tregs SIRPG

T_cells_regulatory__Tregs CD6

T_cells_regulatory__Tregs SH2D1A

T_cells_regulatory__Tregs MAP4K1

T_cells_regulatory__Tregs FOXP3

T_cells_regulatory__Tregs CD70

T_cells_regulatory__Tregs ACAP1

T_cells_regulatory__Tregs SAMSN1

T_cells_regulatory__Tregs TRAV9-2

T_cells_regulatory__Tregs TYR

T_cells_regulatory__Tregs ZAP70

T_cells_regulatory__Tregs PRF1

T_cells_regulatory__Tregs CD28

T_cells_regulatory__Tregs SLAMF1

T_cells_regulatory__Tregs NPIPB15

T_cells_regulatory__Tregs RPL3P7

T_cells_regulatory__Tregs PASK

T_cells_regulatory__Tregs RGS1

T_cells_regulatory__Tregs UBASH3A

T_cells_regulatory__Tregs CST7

T_cells_regulatory__Tregs CD3G

T_cells_regulatory__Tregs PTGIR

T_cells_regulatory__Tregs RYR1

T_cells_regulatory__Tregs KLRB1

T_cells_regulatory__Tregs RASGRP2

T_cells_regulatory__Tregs CCND2

T_cells_regulatory__Tregs SEC31B

T_cells_regulatory__Tregs ETS1

T_cells_regulatory__Tregs FRMD8

T_cells_gamma_delta CCL5

T_cells_gamma_delta TRDC

T_cells_gamma_delta GZMK

T_cells_gamma_delta PRF1

T_cells_gamma_delta GZMA

T_cells_gamma_delta KLRB1

T_cells_gamma_delta CST7

T_cells_gamma_delta CD3D

T_cells_gamma_delta IL2RB

T_cells_gamma_delta SELL

T_cells_gamma_delta GZMH

T_cells_gamma_delta NKG7

T_cells_gamma_delta CD247

T_cells_gamma_delta CCL4

T_cells_gamma_delta GNLY

T_cells_gamma_delta GZMB

T_cells_gamma_delta CD2

T_cells_gamma_delta ITK

T_cells_gamma_delta APOBEC3G

T_cells_gamma_delta TRBC1

T_cells_gamma_delta KLRK1

T_cells_gamma_delta FCN1

T_cells_gamma_delta KLRF1

T_cells_gamma_delta GPR171

T_cells_gamma_delta KLRG1

T_cells_gamma_delta DUSP2

T_cells_gamma_delta CD69

T_cells_gamma_delta IL18RAP

T_cells_gamma_delta LCK

T_cells_gamma_delta CTSW

T_cells_gamma_delta PVRIG

T_cells_gamma_delta KLRD1

T_cells_gamma_delta LTB

T_cells_gamma_delta CD160

T_cells_gamma_delta TRAC

T_cells_gamma_delta CD96

T_cells_gamma_delta PTGER2

T_cells_gamma_delta SKAP1

T_cells_gamma_delta LAT

T_cells_gamma_delta BCL11B

T_cells_gamma_delta GPR65

T_cells_gamma_delta CCR5

T_cells_gamma_delta ADRB2

T_cells_gamma_delta RIPOR2

T_cells_gamma_delta S100A12

T_cells_gamma_delta CD27

T_cells_gamma_delta CD300A

T_cells_gamma_delta MNDA

T_cells_gamma_delta MS4A6A

T_cells_gamma_delta IL7R

T_cells_gamma_delta IGKC

T_cells_gamma_delta FCMR

T_cells_gamma_delta NCR3

T_cells_gamma_delta CD37

T_cells_gamma_delta TBX21

T_cells_gamma_delta SPOCK2

T_cells_gamma_delta CD3E

T_cells_gamma_delta PTPRCAP

T_cells_gamma_delta NCF2

T_cells_gamma_delta LY86

T_cells_gamma_delta SAMSN1

T_cells_gamma_delta MAP4K1

T_cells_gamma_delta SH2D1A

T_cells_gamma_delta TRAT1

T_cells_gamma_delta ZAP70

T_cells_gamma_delta LST1

T_cells_gamma_delta PIK3IP1

T_cells_gamma_delta SIT1

T_cells_gamma_delta LAIR2

T_cells_gamma_delta APOL3

T_cells_gamma_delta CCR2

T_cells_gamma_delta LY9

T_cells_gamma_delta CD8A

T_cells_gamma_delta CD3G

T_cells_gamma_delta CD244

T_cells_gamma_delta P2RX5

T_cells_gamma_delta AIF1

T_cells_gamma_delta PLEKHF1

T_cells_gamma_delta CXCR6

T_cells_gamma_delta VNN2

T_cells_gamma_delta IL18R1

NK_cells_resting PRF1

NK_cells_resting GNLY

NK_cells_resting IL2RB

NK_cells_resting CCL5

NK_cells_resting CD247

NK_cells_resting GZMB

NK_cells_resting NKG7

NK_cells_resting GZMA

NK_cells_resting KLRB1

NK_cells_resting GZMH

NK_cells_resting CST7

NK_cells_resting CCL4

NK_cells_resting TRDC

NK_cells_resting KLRF1

NK_cells_resting KLRK1

NK_cells_resting KLRD1

NK_cells_resting CTSW

NK_cells_resting APOBEC3G

NK_cells_resting CD2

NK_cells_resting IL18RAP

NK_cells_resting SELL

NK_cells_resting CD300A

NK_cells_resting ZAP70

NK_cells_resting PVRIG

NK_cells_resting TRBC1

NK_cells_resting TBX21

NK_cells_resting GZMM

NK_cells_resting LCK

NK_cells_resting PTPRCAP

NK_cells_resting DEFA4

NK_cells_resting ITK

NK_cells_resting PTGER2

NK_cells_resting TTC38

NK_cells_resting FCMR

NK_cells_resting CD160

NK_cells_resting GPR65

NK_cells_resting CD37

NK_cells_resting SKAP1

NK_cells_resting GZMK

NK_cells_resting ADRB2

NK_cells_resting CD244

NK_cells_resting CD69

NK_cells_resting LAT

NK_cells_resting KLRG1

NK_cells_resting CD7

NK_cells_resting CAMP

NK_cells_resting CD96

NK_cells_resting PTGDR

NK_cells_resting IRF8

NK_cells_resting LAIR2

NK_cells_resting RIPOR2

NK_cells_resting TXK

NK_cells_resting FASLG

NK_cells_resting PLEKHF1

NK_cells_resting KLRC3

NK_cells_resting NCR3

NK_cells_resting CD38

NK_cells_resting RASGRP2

NK_cells_resting TRIB2

NK_cells_resting SH2D1A

NK_cells_resting SAMSN1

NK_cells_resting LRMP

NK_cells_resting GPR18

NK_cells_resting PIK3IP1

NK_cells_resting KIR2DS4

NK_cells_resting APOL3

NK_cells_resting IL7R

NK_cells_resting S100A12

NK_cells_resting CCND2

NK_cells_resting PRR5L

NK_cells_resting LTB

NK_cells_activated CCL4

NK_cells_activated PRF1

NK_cells_activated GNLY

NK_cells_activated GZMB

NK_cells_activated GZMA

NK_cells_activated IL2RB

NK_cells_activated LTB

NK_cells_activated CD247

NK_cells_activated NKG7

NK_cells_activated CCL5

NK_cells_activated TRDC

NK_cells_activated CTSW

NK_cells_activated CST7

NK_cells_activated APOBEC3G

NK_cells_activated GZMH

NK_cells_activated KLRD1

NK_cells_activated CCND2

NK_cells_activated KLRF1

NK_cells_activated PTGER2

NK_cells_activated IL18RAP

NK_cells_activated KLRB1

NK_cells_activated CD69

NK_cells_activated KLRK1

NK_cells_activated CD300A

NK_cells_activated PVRIG

NK_cells_activated ITK

NK_cells_activated IFNG

NK_cells_activated TBX21

NK_cells_activated PTPRCAP

NK_cells_activated NCR3

NK_cells_activated CD2

NK_cells_activated KIR2DS4

NK_cells_activated LTA

NK_cells_activated SAMSN1

NK_cells_activated TRBC1

NK_cells_activated LCK

NK_cells_activated GZMM

NK_cells_activated IRF8

NK_cells_activated ADRB2

NK_cells_activated FASLG

NK_cells_activated GPR65

NK_cells_activated CD96

NK_cells_activated CD7

NK_cells_activated TNFSF14

NK_cells_activated PTGDR

NK_cells_activated GPR18

NK_cells_activated TXK

NK_cells_activated ZAP70

NK_cells_activated CD244

NK_cells_activated KIR2DL4

NK_cells_activated LAIR2

NK_cells_activated IL4R

NK_cells_activated CD37

NK_cells_activated SELL

NK_cells_activated IL12RB2

NK_cells_activated SKAP1

NK_cells_activated CXCL10

NK_cells_activated CCR5

NK_cells_activated EGR2

NK_cells_activated LAT

NK_cells_activated SOCS1

NK_cells_activated CD8A

NK_cells_activated BIRC3

NK_cells_activated KIR3DL2

NK_cells_activated PRR5L

NK_cells_activated IL18R1

NK_cells_activated TRIB2

NK_cells_activated GPR171

NK_cells_activated ATP8B4

NK_cells_activated APOL3

Monocytes FCN1

Monocytes NCF2

Monocytes MNDA

Monocytes S100A12

Monocytes FPR1

Monocytes MS4A6A

Monocytes AIF1

Monocytes HCK

Monocytes SELL

Monocytes CLEC7A

Monocytes LST1

Monocytes LY86

Monocytes TLR2

Monocytes CD37

Monocytes C5AR1

Monocytes RNASE6

Monocytes CFP

Monocytes RNASE2

Monocytes FOSB

Monocytes LILRB2

Monocytes CD4

Monocytes CHST15

Monocytes VNN2

Monocytes HK3

Monocytes CCL4

Monocytes CCR2

Monocytes CLEC4A

Monocytes P2RY13

Monocytes CD1D

Monocytes IGSF6

Monocytes IRF8

Monocytes RIPOR2

Monocytes APOBEC3A

Monocytes CSF3R

Monocytes BCL2A1

Monocytes CD300A

Monocytes AQP9

Monocytes ALOX5

Monocytes HHEX

Monocytes ADGRE1

Monocytes TREM1

Monocytes LILRA2

Monocytes SLC15A3

Monocytes BST1

Monocytes TLR8

Monocytes NFE2

Monocytes QPCT

Monocytes CD33

Monocytes ASGR2

Monocytes GPR65

Monocytes DPEP2

Monocytes SAMSN1

Monocytes CD86

Monocytes ZFP36L2

Monocytes C3AR1

Monocytes ADGRE2

Monocytes NOD2

Monocytes PLA2G7

Monocytes HPSE

Monocytes IL1B

Monocytes IL4R

Monocytes PTGER2

Monocytes LRMP

Monocytes CDA

Monocytes VNN1

Monocytes APOL3

Macrophages_M0 MMP9

Macrophages_M0 NCF2

Macrophages_M0 ACP5

Macrophages_M0 PLA2G7

Macrophages_M0 CYP27A1

Macrophages_M0 CHI3L1

Macrophages_M0 IGSF6

Macrophages_M0 BCL2A1

Macrophages_M0 BHLHE41

Macrophages_M0 CXCL5

Macrophages_M0 IL7R

Macrophages_M0 C5AR1

Macrophages_M0 AQP9

Macrophages_M0 SLC15A3

Macrophages_M0 ADAMDEC1

Macrophages_M0 EGR2

Macrophages_M0 CLEC7A

Macrophages_M0 PPBP

Macrophages_M0 HK3

Macrophages_M0 HCK

Macrophages_M0 DCSTAMP

Macrophages_M0 KYNU

Macrophages_M0 FPR3

Macrophages_M0 CCL18

Macrophages_M0 QPCT

Macrophages_M0 MNDA

Macrophages_M0 CCL22

Macrophages_M0 RNASE6

Macrophages_M0 SLAMF8

Macrophages_M0 MARCO

Macrophages_M0 CCR5

Macrophages_M0 LST1

Macrophages_M0 TREM2

Macrophages_M0 CCL4

Macrophages_M0 IRF8

Macrophages_M0 AIF1

Macrophages_M0 LY86

Macrophages_M0 CD68

Macrophages_M0 NPL

Macrophages_M0 CXCL3

Macrophages_M0 CD37

Macrophages_M0 CHST15

Macrophages_M0 C1orf54

Macrophages_M0 FCN1

Macrophages_M0 IL1B

Macrophages_M0 COL8A2

Macrophages_M0 CYP27B1

Macrophages_M0 GPC4

Macrophages_M0 CD40

Macrophages_M0 FLVCR2

Macrophages_M0 CLEC4A

Macrophages_M0 IL4R

Macrophages_M0 ADGRE2

Macrophages_M1 CXCL9

Macrophages_M1 CCL19

Macrophages_M1 CXCL10

Macrophages_M1 IDO1

Macrophages_M1 EBI3

Macrophages_M1 TNFAIP6

Macrophages_M1 CCR7

Macrophages_M1 LAMP3

Macrophages_M1 CCL5

Macrophages_M1 CD40

Macrophages_M1 IRF8

Macrophages_M1 CD38

Macrophages_M1 CCL4

Macrophages_M1 APOL3

Macrophages_M1 CXCL11

Macrophages_M1 ADAMDEC1

Macrophages_M1 HCK

Macrophages_M1 BCL2A1

Macrophages_M1 RSAD2

Macrophages_M1 CYP27B1

Macrophages_M1 KYNU

Macrophages_M1 SIGLEC1

Macrophages_M1 SLAMF1

Macrophages_M1 SLC2A6

Macrophages_M1 IFI44L

Macrophages_M1 SAMSN1

Macrophages_M1 LILRB2

Macrophages_M1 TLR2

Macrophages_M1 CCL8

Macrophages_M1 APOBEC3A

Macrophages_M1 AQP9

Macrophages_M1 C3AR1

Macrophages_M1 HLA-DQA1

Macrophages_M1 MNDA

Macrophages_M1 IL2RA

Macrophages_M1 SLC15A3

Macrophages_M1 CD80

Macrophages_M1 ADGRE1

Macrophages_M1 CHI3L1

Macrophages_M1 RASSF4

Macrophages_M1 IL4R

Macrophages_M1 AIM2

Macrophages_M1 PLA1A

Macrophages_M1 MMP9

Macrophages_M1 TLR8

Macrophages_M1 HESX1

Macrophages_M1 SOCS1

Macrophages_M1 LST1

Macrophages_M1 NOD2

Macrophages_M1 IL7R

Macrophages_M1 FLVCR2

Macrophages_M1 BIRC3

Macrophages_M1 PTGIR

Macrophages_M1 CHI3L2

Macrophages_M1 PLA2G7

Macrophages_M1 GPR183

Macrophages_M1 AIF1

Macrophages_M1 CD4

Macrophages_M1 HLA-DOB

Macrophages_M1 MSC

Macrophages_M1 ADGRE2

Macrophages_M1 CCR5

Macrophages_M1 CLEC2D

Macrophages_M1 TNFRSF4

Macrophages_M1 CD86

Macrophages_M1 SPIB

Macrophages_M1 LAG3

Macrophages_M1 DHX58

Macrophages_M1 CLIC2

Macrophages_M1 ACHE

Macrophages_M1 CCL18

Macrophages_M1 LY86

Macrophages_M1 CCL14

Macrophages_M1 TNIP3

Macrophages_M1 C1orf54

Macrophages_M1 MMP25

Macrophages_M1 ACP5

Macrophages_M1 APOBEC3G

Macrophages_M2 MMP9

Macrophages_M2 MS4A6A

Macrophages_M2 CCL18

Macrophages_M2 AIF1

Macrophages_M2 NCF2

Macrophages_M2 CD4

Macrophages_M2 ACP5

Macrophages_M2 CLEC4A

Macrophages_M2 CCL13

Macrophages_M2 LY86

Macrophages_M2 SLC15A3

Macrophages_M2 CLEC10A

Macrophages_M2 CCL23

Macrophages_M2 HLA-DQA1

Macrophages_M2 RNASE6

Macrophages_M2 ADAMDEC1

Macrophages_M2 HCK

Macrophages_M2 NPL

Macrophages_M2 TREM2

Macrophages_M2 IRF8

Macrophages_M2 SIGLEC1

Macrophages_M2 CCL4

Macrophages_M2 SAMSN1

Macrophages_M2 CLEC7A

Macrophages_M2 TLR2

Macrophages_M2 P2RY13

Macrophages_M2 CCL8

Macrophages_M2 CD86

Macrophages_M2 CD180

Macrophages_M2 CD68

Macrophages_M2 CD209

Macrophages_M2 C3AR1

Macrophages_M2 GPR183

Macrophages_M2 CCL14

Macrophages_M2 DPEP2

Macrophages_M2 FPR3

Macrophages_M2 CD37

Macrophages_M2 LST1

Macrophages_M2 CLIC2

Macrophages_M2 HRH1

Macrophages_M2 EGR2

Macrophages_M2 CHI3L1

Macrophages_M2 C1orf54

Macrophages_M2 CFP

Macrophages_M2 C5AR1

Macrophages_M2 MNDA

Macrophages_M2 PTGER2

Macrophages_M2 CD300A

Macrophages_M2 PIK3IP1

Macrophages_M2 IGSF6

Macrophages_M2 RENBP

Macrophages_M2 PLA2G7

Macrophages_M2 FCGR2B

Macrophages_M2 SLAMF8

Macrophages_M2 RASGRP3

Macrophages_M2 QPCT

Macrophages_M2 LILRB2

Macrophages_M2 ATP8B4

Macrophages_M2 PTGIR

Macrophages_M2 FRMD4A

Macrophages_M2 TLR8

Macrophages_M2 KYNU

Macrophages_M2 CHST15

Dendritic_cells_resting MMP12

Dendritic_cells_resting CD1B

Dendritic_cells_resting CD1A

Dendritic_cells_resting ACP5

Dendritic_cells_resting NCF2

Dendritic_cells_resting CD1C

Dendritic_cells_resting CD1E

Dendritic_cells_resting RNASE6

Dendritic_cells_resting HLA-DQA1

Dendritic_cells_resting TREM2

Dendritic_cells_resting FPR3

Dendritic_cells_resting PLA2G7

Dendritic_cells_resting CLEC7A

Dendritic_cells_resting CLEC10A

Dendritic_cells_resting C1orf54

Dendritic_cells_resting MNDA

Dendritic_cells_resting EGR2

Dendritic_cells_resting HCK

Dendritic_cells_resting CLEC4A

Dendritic_cells_resting CCL22

Dendritic_cells_resting MS4A6A

Dendritic_cells_resting CCL13

Dendritic_cells_resting LST1

Dendritic_cells_resting SLC15A3

Dendritic_cells_resting IGSF6

Dendritic_cells_resting AIF1

Dendritic_cells_resting MMP9

Dendritic_cells_resting ALOX15

Dendritic_cells_resting SLAMF8

Dendritic_cells_resting CCL17

Dendritic_cells_resting CD209

Dendritic_cells_resting FLVCR2

Dendritic_cells_resting CLIC2

Dendritic_cells_resting FCER1A

Dendritic_cells_resting IL7R

Dendritic_cells_resting CCL4

Dendritic_cells_resting SAMSN1

Dendritic_cells_resting NPL

Dendritic_cells_resting LY86

Dendritic_cells_resting KYNU

Dendritic_cells_resting CD86

Dendritic_cells_resting CCL18

Dendritic_cells_resting IRF8

Dendritic_cells_resting CD40

Dendritic_cells_resting QPCT

Dendritic_cells_resting FCGR2B

Dendritic_cells_resting CCR5

Dendritic_cells_resting CST7

Dendritic_cells_resting CD4

Dendritic_cells_resting TLR2

Dendritic_cells_resting C5AR1

Dendritic_cells_resting CD68

Dendritic_cells_resting AQP9

Dendritic_cells_resting FCER2

Dendritic_cells_resting IL4R

Dendritic_cells_resting HK3

Dendritic_cells_resting DHRS11

Dendritic_cells_resting LAMP3

Dendritic_cells_activated CCL22

Dendritic_cells_activated LAMP3

Dendritic_cells_activated IDO1

Dendritic_cells_activated CCR7

Dendritic_cells_activated CXCL10

Dendritic_cells_activated BIRC3

Dendritic_cells_activated TNFAIP6

Dendritic_cells_activated IL7R

Dendritic_cells_activated RSAD2

Dendritic_cells_activated SAMSN1

Dendritic_cells_activated KYNU

Dendritic_cells_activated CCL5

Dendritic_cells_activated EBI3

Dendritic_cells_activated HLA-DQA1

Dendritic_cells_activated CCL17

Dendritic_cells_activated BCL2A1

Dendritic_cells_activated CCL13

Dendritic_cells_activated CLIC2

Dendritic_cells_activated PLA2G7

Dendritic_cells_activated CD40

Dendritic_cells_activated SLC15A3

Dendritic_cells_activated CD80

Dendritic_cells_activated CD86

Dendritic_cells_activated CCL4

Dendritic_cells_activated FPR3

Dendritic_cells_activated CST7

Dendritic_cells_activated IFI44L

Dendritic_cells_activated ACP5

Dendritic_cells_activated CXCL11

Dendritic_cells_activated MMP12

Dendritic_cells_activated HCK

Dendritic_cells_activated RGS1

Dendritic_cells_activated CD38

Dendritic_cells_activated NCF2

Dendritic_cells_activated CHST7

Dendritic_cells_activated CCL19

Dendritic_cells_activated ARHGAP22

Dendritic_cells_activated CXCL9

Dendritic_cells_activated MMP9

Dendritic_cells_activated IRF8

Dendritic_cells_activated IL1B

Dendritic_cells_activated SIGLEC1

Dendritic_cells_activated APOBEC3A

Dendritic_cells_activated RASSF4

Dendritic_cells_activated IL4R

Dendritic_cells_activated IL12B

Dendritic_cells_activated CD1B

Dendritic_cells_activated CYP27B1

Dendritic_cells_activated IL2RA

Dendritic_cells_activated NR4A3

Dendritic_cells_activated MNDA

Dendritic_cells_activated ETV3

Dendritic_cells_activated HLA-DOB

Dendritic_cells_activated CLEC2D

Dendritic_cells_activated SLC2A6

Dendritic_cells_activated CYP27A1

Dendritic_cells_activated GPR183

Dendritic_cells_activated MSC

Dendritic_cells_activated ADAMDEC1

Dendritic_cells_activated CCL18

Dendritic_cells_activated TNFRSF4

Dendritic_cells_activated DHX58

Dendritic_cells_activated CCL8

Dendritic_cells_activated CCL20

Dendritic_cells_activated AQP9

Dendritic_cells_activated CD1E

Dendritic_cells_activated APOBEC3G

Dendritic_cells_activated PTGIR

Dendritic_cells_activated CD1A

Dendritic_cells_activated ST3GAL6

Dendritic_cells_activated IGSF6

Dendritic_cells_activated CLEC7A

Dendritic_cells_activated C1orf54

Dendritic_cells_activated MMP25

Dendritic_cells_activated EGR2

Dendritic_cells_activated HESX1

Dendritic_cells_activated FLVCR2

Dendritic_cells_activated SLCO5A1

Dendritic_cells_activated RASGRP3

Dendritic_cells_activated CD1C

Mast_cells_resting CPA3

Mast_cells_resting TPSAB1

Mast_cells_resting HPGDS

Mast_cells_resting PRG2

Mast_cells_resting HDC

Mast_cells_resting SAMSN1

Mast_cells_resting CTSG

Mast_cells_resting ADRB2

Mast_cells_resting C3AR1

Mast_cells_resting ELANE

Mast_cells_resting FCER1A

Mast_cells_resting MS4A2

Mast_cells_resting MYB

Mast_cells_resting CLC

Mast_cells_resting LAT

Mast_cells_resting STAP1

Mast_cells_resting IL1B

Mast_cells_resting IL4R

Mast_cells_resting RGS13

Mast_cells_resting P2RX1

Mast_cells_resting BMP2K

Mast_cells_resting CEACAM8

Mast_cells_resting CST7

Mast_cells_resting P2RY14

Mast_cells_resting MNDA

Mast_cells_resting RNASE2

Mast_cells_resting MS4A3

Mast_cells_resting TRIB2

Mast_cells_resting AIF1

Mast_cells_resting ATP8B4

Mast_cells_resting 3-Mar

Mast_cells_resting GPR65

Mast_cells_resting IL18R1

Mast_cells_resting CMA1

Mast_cells_resting ITK

Mast_cells_resting HHEX

Mast_cells_resting KLRG1

Mast_cells_resting LST1

Mast_cells_resting MMP9

Mast_cells_resting LTC4S

Mast_cells_resting CCL4

Mast_cells_resting CHST15

Mast_cells_resting GFI1

Mast_cells_resting PLA2G7

Mast_cells_resting S100A12

Mast_cells_activated CCL4

Mast_cells_activated TPSAB1

Mast_cells_activated CPA3

Mast_cells_activated PRG2

Mast_cells_activated IL1B

Mast_cells_activated HPGDS

Mast_cells_activated MMP9

Mast_cells_activated HDC

Mast_cells_activated CXCL3

Mast_cells_activated GZMB

Mast_cells_activated CTSG

Mast_cells_activated CCL20

Mast_cells_activated MYB

Mast_cells_activated C3AR1

Mast_cells_activated IL4R

Mast_cells_activated ELANE

Mast_cells_activated SAMSN1

Mast_cells_activated FCER1A

Mast_cells_activated RGS13

Mast_cells_activated BCL2A1

Mast_cells_activated CLC

Mast_cells_activated IL1A

Mast_cells_activated ADRB2

Mast_cells_activated MMP12

Mast_cells_activated 3-Mar

Mast_cells_activated MS4A2

Mast_cells_activated CST7

Mast_cells_activated LAT

Mast_cells_activated BMP2K

Mast_cells_activated TNFAIP6

Mast_cells_activated RNASE2

Mast_cells_activated IL18R1

Mast_cells_activated P2RX1

Mast_cells_activated STAP1

Mast_cells_activated ATP8B4

Mast_cells_activated BIRC3

Mast_cells_activated EGR2

Mast_cells_activated AQP9

Mast_cells_activated CEACAM8

Mast_cells_activated CSF2

Mast_cells_activated AIF1

Mast_cells_activated LST1

Mast_cells_activated CMA1

Mast_cells_activated NCF2

Mast_cells_activated FCGR2B

Mast_cells_activated TLR2

Mast_cells_activated ADAMDEC1

Mast_cells_activated ADGRE2

Mast_cells_activated GPR65

Mast_cells_activated CD69

Mast_cells_activated P2RY14

Mast_cells_activated PPBP

Mast_cells_activated TRIB2

Mast_cells_activated CD38

Mast_cells_activated CCL8

Mast_cells_activated CLEC7A

Mast_cells_activated KLRG1

Mast_cells_activated IL1RL1

Mast_cells_activated MNDA

Mast_cells_activated CCL1

Mast_cells_activated NR4A3

Mast_cells_activated NTRK1

Mast_cells_activated CD37

Mast_cells_activated ITK

Mast_cells_activated CD300A

Mast_cells_activated LTC4S

Mast_cells_activated AZU1

Eosinophils CLC

Eosinophils NCF2

Eosinophils ADGRE1

Eosinophils BCL2A1

Eosinophils SAMSN1

Eosinophils P2RY14

Eosinophils C3AR1

Eosinophils GPR65

Eosinophils FOSB

Eosinophils CCR3

Eosinophils CCL4

Eosinophils RNASE2

Eosinophils CD69

Eosinophils LRMP

Eosinophils RGS1

Eosinophils ZNF165

Eosinophils GPR183

Eosinophils ADGRE3

Eosinophils ADGRG3

Eosinophils LST1

Eosinophils DPEP2

Eosinophils NR4A3

Eosinophils ADGRE2

Eosinophils FPR1

Eosinophils HCK

Eosinophils MYB

Eosinophils MNDA

Eosinophils SELL

Eosinophils RIPOR2

Eosinophils C5AR1

Eosinophils FCER1A

Eosinophils CD244

Eosinophils CD300A

Eosinophils SIK1

Eosinophils LTB

Eosinophils IL1A

Eosinophils ADRB2

Eosinophils CD37

Eosinophils P2RY10

Eosinophils IL18RAP

Eosinophils SMPD3

Eosinophils OSM

Eosinophils RGS13

Eosinophils BIRC3

Eosinophils ST3GAL6

Eosinophils MXD1

Eosinophils RASGRP2

Eosinophils IL18R1

Eosinophils ALOX5

Eosinophils PLEKHG3

Eosinophils P2RY13

Eosinophils EGR2

Eosinophils IDO1

Eosinophils MS4A3

Eosinophils P2RX1

Eosinophils MMP25

Eosinophils HK3

Eosinophils 3-Mar

Eosinophils IL1B

Eosinophils APOBEC3A

Eosinophils HDC

Eosinophils IL4R

Eosinophils IGKC

Eosinophils CHST15

Eosinophils DAPK2

Eosinophils NPL

Eosinophils CPA3

Eosinophils GPR171

Eosinophils RSAD2

Eosinophils ANKRD55

Eosinophils FFAR2

Eosinophils LILRB2

Eosinophils DUSP2

Neutrophils FPR1

Neutrophils FCGR3B

Neutrophils MNDA

Neutrophils VNN2

Neutrophils CXCR2

Neutrophils NCF2

Neutrophils SELL

Neutrophils AQP9

Neutrophils C5AR1

Neutrophils LST1

Neutrophils CSF3R

Neutrophils BCL2A1

Neutrophils S100A12

Neutrophils TREM1

Neutrophils RIPOR2

Neutrophils HCK

Neutrophils MMP9

Neutrophils AIF1

Neutrophils CXCR1

Neutrophils APOBEC3A

Neutrophils LTB

Neutrophils TLR2

Neutrophils FCN1

Neutrophils CLEC7A

Neutrophils MGAM

Neutrophils P2RY13

Neutrophils CLC

Neutrophils CHST15

Neutrophils DPEP2

Neutrophils MMP25

Neutrophils IGSF6

Neutrophils CD300A

Neutrophils FFAR2

Neutrophils ADGRE2

Neutrophils TNFRSF10C

Neutrophils FPR2

Neutrophils CHI3L1

Neutrophils CDA

Neutrophils TNFAIP6

Neutrophils HSPA6

Neutrophils QPCT

Neutrophils LRMP

Neutrophils CD37

Neutrophils HAL

Neutrophils IL1B

Neutrophils ADGRE3

Neutrophils NFE2

Neutrophils ALOX5

Neutrophils LILRB2

Neutrophils CLEC4A

Neutrophils IL4R

Neutrophils CCL4

Neutrophils IL18RAP

Neutrophils GPR65

Neutrophils LILRA2

Neutrophils MXD1

Neutrophils CST7

Neutrophils ADGRG3

Neutrophils VNN3

Neutrophils NPL

Neutrophils CEACAM3

Neutrophils CFP

Neutrophils RUBCNL

Neutrophils RNASE6

Neutrophils BST1

Neutrophils TLR8

Neutrophils SAMSN1

Neutrophils P2RY14

Neutrophils PLEKHG3

Neutrophils PGLYRP1

Neutrophils CAMP

Neutrophils CCR3

Neutrophils SLC15A3

Neutrophils CREB5

Neutrophils REPS2

Neutrophils FCGR2B

Neutrophils RASGRP2

Neutrophils HK3

Neutrophils ADGRE1

Neutrophils PGGHG

Neutrophils P2RX1

Neutrophils MS4A6A

Neutrophils MAK

Neutrophils TREML2

Neutrophils NPIPB15

Neutrophils BTNL8

Neutrophils IL2RB

Neutrophils TPSAB1

B_cells_ImSig AFF3

B_cells_ImSig BANK1

B_cells_ImSig BLK

B_cells_ImSig BTLA

B_cells_ImSig CCR6

B_cells_ImSig CD180

B_cells_ImSig CD19

B_cells_ImSig CD22

B_cells_ImSig CD37

B_cells_ImSig CD72

B_cells_ImSig CD79A

B_cells_ImSig CD79B

B_cells_ImSig CR2

B_cells_ImSig EBF1

B_cells_ImSig FAM129C

B_cells_ImSig FCRL1

B_cells_ImSig FCRL2

B_cells_ImSig FCRL3

B_cells_ImSig FCRL5

B_cells_ImSig FCRLA

B_cells_ImSig HLA-DOB

B_cells_ImSig IGHV5-78

B_cells_ImSig KIAA0125

B_cells_ImSig LINC00926

B_cells_ImSig LOC100507616

B_cells_ImSig LY9

B_cells_ImSig MS4A1

B_cells_ImSig P2RX5

B_cells_ImSig PAX5

B_cells_ImSig PNOC

B_cells_ImSig POU2F2

B_cells_ImSig S1PR4

B_cells_ImSig SNX22

B_cells_ImSig STAP1

B_cells_ImSig TCL1A

B_cells_ImSig TLR10

B_cells_ImSig VPREB3

T_cells_ImSig AMICA1

T_cells_ImSig APBB1IP

T_cells_ImSig ARHGAP15

T_cells_ImSig ARHGAP25

T_cells_ImSig ARHGAP9

T_cells_ImSig BIN2

T_cells_ImSig BTK

T_cells_ImSig C1orf162

T_cells_ImSig CCL19

T_cells_ImSig CCR7

T_cells_ImSig CD2

T_cells_ImSig CD27

T_cells_ImSig CD28

T_cells_ImSig CD3D

T_cells_ImSig CD3E

T_cells_ImSig CD3G

T_cells_ImSig CD48

T_cells_ImSig CD52

T_cells_ImSig CD6

T_cells_ImSig CD8A

T_cells_ImSig CD96

T_cells_ImSig CORO1A

T_cells_ImSig CRTAM

T_cells_ImSig CXCL9

T_cells_ImSig CXCR6

T_cells_ImSig CYTIP

T_cells_ImSig DOCK10

T_cells_ImSig DOCK2

T_cells_ImSig DOCK8

T_cells_ImSig DPEP2

T_cells_ImSig EVI2A

T_cells_ImSig EVI2B

T_cells_ImSig FAM26F

T_cells_ImSig FLI1

T_cells_ImSig FYB

T_cells_ImSig FYN

T_cells_ImSig GAB3

T_cells_ImSig GIMAP2

T_cells_ImSig GIMAP4

T_cells_ImSig GIMAP5

T_cells_ImSig GIMAP6

T_cells_ImSig GIMAP7

T_cells_ImSig GMFG

T_cells_ImSig GPR171

T_cells_ImSig GPR18

T_cells_ImSig GZMK

T_cells_ImSig HCST

T_cells_ImSig HMHA1

T_cells_ImSig HVCN1

T_cells_ImSig ICOS

T_cells_ImSig IL10RA

T_cells_ImSig IL16

T_cells_ImSig IL23A

T_cells_ImSig IL7R

T_cells_ImSig ITGAL

T_cells_ImSig ITK

T_cells_ImSig KLHL6

T_cells_ImSig KLRB1

T_cells_ImSig LCP1

T_cells_ImSig LY86

T_cells_ImSig NCF1B

T_cells_ImSig NLRC3

T_cells_ImSig PARVG

T_cells_ImSig PRKCH

T_cells_ImSig PSTPIP1

T_cells_ImSig PTPRCAP

T_cells_ImSig PVRIG

T_cells_ImSig RASSF5

T_cells_ImSig RCSD1

T_cells_ImSig RGS18

T_cells_ImSig RHOH

T_cells_ImSig SASH3

T_cells_ImSig SH2D1A

T_cells_ImSig SIRPG

T_cells_ImSig SLA

T_cells_ImSig SP140

T_cells_ImSig TARP

T_cells_ImSig TBC1D10C

T_cells_ImSig TNFRSF9

T_cells_ImSig TRAC

T_cells_ImSig TRAF3IP3

T_cells_ImSig TRAT1

T_cells_ImSig TRGC2

T_cells_ImSig TRGV9

T_cells_ImSig UBASH3A

Macrophages_ImSig ADAMDEC1

Macrophages_ImSig ADORA3

Macrophages_ImSig AOAH

Macrophages_ImSig ARRB2

Macrophages_ImSig ATP8B4

Macrophages_ImSig BCL2A1

Macrophages_ImSig C1orf54

Macrophages_ImSig C1QA

Macrophages_ImSig C1QB

Macrophages_ImSig C2

Macrophages_ImSig C3AR1

Macrophages_ImSig C5AR1

Macrophages_ImSig CCR1

Macrophages_ImSig CCRL2

Macrophages_ImSig CD163

Macrophages_ImSig CD300A

Macrophages_ImSig CD4

Macrophages_ImSig CD68

Macrophages_ImSig CD74

Macrophages_ImSig CD86

Macrophages_ImSig CECR1

Macrophages_ImSig CLEC7A

Macrophages_ImSig CMKLR1

Macrophages_ImSig CSF1R

Macrophages_ImSig CTSB

Macrophages_ImSig CTSS

Macrophages_ImSig CYBB

Macrophages_ImSig CYTH4

Macrophages_ImSig DPYD

Macrophages_ImSig EMR2

Macrophages_ImSig FCER1G

Macrophages_ImSig FCGR1A

Macrophages_ImSig FCGR1B

Macrophages_ImSig FCGR2A

Macrophages_ImSig FCGR3B

Macrophages_ImSig FPR3

Macrophages_ImSig GPNMB

Macrophages_ImSig HK3

Macrophages_ImSig HLA-DRB6

Macrophages_ImSig IFI30

Macrophages_ImSig IGSF6

Macrophages_ImSig ITGAM

Macrophages_ImSig ITGAX

Macrophages_ImSig ITGB2

Macrophages_ImSig LAIR1

Macrophages_ImSig LAPTM5

Macrophages_ImSig LILRB4

Macrophages_ImSig LIPA

Macrophages_ImSig LY96

Macrophages_ImSig MAN2B1

Macrophages_ImSig MFSD1

Macrophages_ImSig MNDA

Macrophages_ImSig MS4A4A

Macrophages_ImSig MS4A7

Macrophages_ImSig MSR1

Macrophages_ImSig MYO1F

Macrophages_ImSig NCKAP1L

Macrophages_ImSig NPL

Macrophages_ImSig NR1H3

Macrophages_ImSig PLA2G7

Macrophages_ImSig PLEKHO2

Macrophages_ImSig SCPEP1

Macrophages_ImSig SLAMF8

Macrophages_ImSig SLC15A3

Macrophages_ImSig SLC31A2

Macrophages_ImSig SLCO2B1

Macrophages_ImSig SNX10

Macrophages_ImSig SPI1

Macrophages_ImSig TBXAS1

Macrophages_ImSig TLR8

Macrophages_ImSig TMEM140

Macrophages_ImSig TNFAIP2

Macrophages_ImSig TNFRSF1B

Macrophages_ImSig TNFSF13B

Macrophages_ImSig TRPV2

Macrophages_ImSig TYMP

Macrophages_ImSig TYROBP

Macrophages_ImSig VSIG4

Monocytes_ImSig AGTRAP

Monocytes_ImSig AIF1

Monocytes_ImSig C10orf54

Monocytes_ImSig CD14

Monocytes_ImSig CD300LF

Monocytes_ImSig CD33

Monocytes_ImSig CD93

Monocytes_ImSig CTSD

Monocytes_ImSig EMILIN2

Monocytes_ImSig FCN1

Monocytes_ImSig FES

Monocytes_ImSig FGR

Monocytes_ImSig GNS

Monocytes_ImSig GRN

Monocytes_ImSig HCK

Monocytes_ImSig HMOX1

Monocytes_ImSig KIAA0930

Monocytes_ImSig LILRA6

Monocytes_ImSig LILRB2

Monocytes_ImSig LILRB3

Monocytes_ImSig LRRC25

Monocytes_ImSig LST1

Monocytes_ImSig NFAM1

Monocytes_ImSig NOTCH2

Monocytes_ImSig PILRA

Monocytes_ImSig PLXDC2

Monocytes_ImSig PRAM1

Monocytes_ImSig PSAP

Monocytes_ImSig PYCARD

Monocytes_ImSig RHOG

Monocytes_ImSig SERPINA1

Monocytes_ImSig SLC7A7

Monocytes_ImSig TGFBI

Monocytes_ImSig THEMIS2

Monocytes_ImSig TIMP2

Monocytes_ImSig TPP1

Monocytes_ImSig VCAN

Neutrophils_ImSig ACSL1

Neutrophils_ImSig ALPK1

Neutrophils_ImSig AQP9

Neutrophils_ImSig BASP1

Neutrophils_ImSig BCL6

Neutrophils_ImSig CD97

Neutrophils_ImSig CEP19

Neutrophils_ImSig CFLAR

Neutrophils_ImSig CSF3R

Neutrophils_ImSig CXCR2

Neutrophils_ImSig DENND5A

Neutrophils_ImSig DYSF

Neutrophils_ImSig FAM65B

Neutrophils_ImSig FCGR2C

Neutrophils_ImSig FPR1

Neutrophils_ImSig GLT1D1

Neutrophils_ImSig GPR97

Neutrophils_ImSig IFITM2

Neutrophils_ImSig IL17RA

Neutrophils_ImSig KCNJ2

Neutrophils_ImSig KIAA0247

Neutrophils_ImSig LILRA2

Neutrophils_ImSig LIMK2

Neutrophils_ImSig LINC01002

Neutrophils_ImSig MGAM

Neutrophils_ImSig MOB3A

Neutrophils_ImSig NAMPT

Neutrophils_ImSig NCF4

Neutrophils_ImSig PADI2

Neutrophils_ImSig PHC2

Neutrophils_ImSig PHF21A

Neutrophils_ImSig PLXNC1

Neutrophils_ImSig PREX1

Neutrophils_ImSig RALB

Neutrophils_ImSig RNF149

Neutrophils_ImSig S100A8

Neutrophils_ImSig S100A9

Neutrophils_ImSig SLC25A37

Neutrophils_ImSig SNORD89

Neutrophils_ImSig SSH2

Neutrophils_ImSig STAT3

Neutrophils_ImSig STAT5B

Neutrophils_ImSig THBD

Neutrophils_ImSig TLR2

Neutrophils_ImSig TLR4

Neutrophils_ImSig TMEM154

Neutrophils_ImSig TNFRSF1A

NK_cells_ImSig KIR2DL1

NK_cells_ImSig KIR2DL2

NK_cells_ImSig KIR2DL3

NK_cells_ImSig KIR2DL4

NK_cells_ImSig KIR2DL5A

NK_cells_ImSig KIR2DS1

NK_cells_ImSig KIR2DS2

NK_cells_ImSig KIR2DS3

NK_cells_ImSig KIR2DS5

NK_cells_ImSig KIR3DL1

NK_cells_ImSig KIR3DL2

NK_cells_ImSig KIR3DL3

NK_cells_ImSig KLRC2

NK_cells_ImSig KLRC3

NK_cells_ImSig KLRC4

NK_cells_ImSig KLRD1

NK_cells_ImSig PRF1

NK_cells_ImSig SAMD3

NK_cells_ImSig SH2D1B

NK_cells_ImSig TBX21

Plasma_cells_ImSig GUSBP11

Plasma_cells_ImSig IGH

Plasma_cells_ImSig IGHG3

Plasma_cells_ImSig IGJ

Plasma_cells_ImSig IGKC

Plasma_cells_ImSig IGKV1D-13

Plasma_cells_ImSig IGLC1

Plasma_cells_ImSig IGLJ3

Plasma_cells_ImSig IGLL3P

Plasma_cells_ImSig IGLV@

Plasma_cells_ImSig IGLV1-44

Plasma_cells_ImSig MZB1

Plasma_cells_ImSig TNFRSF17

Plasma_cells_ImSig TXNDC5

Interferon_ImSig APOL1

Interferon_ImSig APOL6

Interferon_ImSig BATF2

Interferon_ImSig BST2

Interferon_ImSig C19orf66

Interferon_ImSig C5orf56

Interferon_ImSig CMPK2

Interferon_ImSig DDX58

Interferon_ImSig DDX60

Interferon_ImSig DHX58

Interferon_ImSig DTX3L

Interferon_ImSig EPSTI1

Interferon_ImSig FBXO6

Interferon_ImSig GBP1

Interferon_ImSig GBP4

Interferon_ImSig HELZ2

Interferon_ImSig HERC5

Interferon_ImSig HERC6

Interferon_ImSig HSH2D

Interferon_ImSig IFI16

Interferon_ImSig IFI35

Interferon_ImSig IFI44

Interferon_ImSig IFI44L

Interferon_ImSig IFI6

Interferon_ImSig IFIH1

Interferon_ImSig IFIT1

Interferon_ImSig IFIT2

Interferon_ImSig IFIT3

Interferon_ImSig IFIT5

Interferon_ImSig IFITM1

Interferon_ImSig IRF7

Interferon_ImSig IRF9

Interferon_ImSig ISG15

Interferon_ImSig LAMP3

Interferon_ImSig LAP3

Interferon_ImSig MX1

Interferon_ImSig MX2

Interferon_ImSig OAS2

Interferon_ImSig OAS3

Interferon_ImSig OASL

Interferon_ImSig PARP10

Interferon_ImSig PARP12

Interferon_ImSig PARP14

Interferon_ImSig PARP9

Interferon_ImSig PHF11

Interferon_ImSig PML

Interferon_ImSig PSMB9

Interferon_ImSig RNF213

Interferon_ImSig RSAD2

Interferon_ImSig RTP4

Interferon_ImSig SAMD9

Interferon_ImSig SAMD9L

Interferon_ImSig SHISA5

Interferon_ImSig SIGLEC1

Interferon_ImSig SP110

Interferon_ImSig STAT1

Interferon_ImSig STAT2

Interferon_ImSig TAP1

Interferon_ImSig TRAFD1

Interferon_ImSig TRIM21

Interferon_ImSig TRIM22

Interferon_ImSig TRIM5

Interferon_ImSig UBE2L6

Interferon_ImSig USP18

Interferon_ImSig XAF1

Interferon_ImSig ZNFX1

Proliferation_ImSig ANLN

Proliferation_ImSig ASPM

Proliferation_ImSig AURKA

Proliferation_ImSig AURKB

Proliferation_ImSig BIRC5

Proliferation_ImSig BUB1

Proliferation_ImSig BUB1B

Proliferation_ImSig CASC5

Proliferation_ImSig CCNA2

Proliferation_ImSig CCNB1

Proliferation_ImSig CCNB2

Proliferation_ImSig CCNE2

Proliferation_ImSig CDC20

Proliferation_ImSig CDC6

Proliferation_ImSig CDCA2

Proliferation_ImSig CDCA3

Proliferation_ImSig CDCA5

Proliferation_ImSig CDCA7

Proliferation_ImSig CDCA8

Proliferation_ImSig CDK1

Proliferation_ImSig CDKN3

Proliferation_ImSig CDT1

Proliferation_ImSig CENPA

Proliferation_ImSig CENPE

Proliferation_ImSig CENPF

Proliferation_ImSig CENPL

Proliferation_ImSig CEP55

Proliferation_ImSig CKS1B

Proliferation_ImSig DEPDC1

Proliferation_ImSig DEPDC1B

Proliferation_ImSig DLGAP5

Proliferation_ImSig DONSON

Proliferation_ImSig DTL

Proliferation_ImSig E2F8

Proliferation_ImSig ECT2

Proliferation_ImSig EZH2

Proliferation_ImSig FAM72C

Proliferation_ImSig FANCI

Proliferation_ImSig FBXO5

Proliferation_ImSig FOXM1

Proliferation_ImSig GINS1

Proliferation_ImSig GINS2

Proliferation_ImSig GMNN

Proliferation_ImSig HJURP

Proliferation_ImSig HMGB3

Proliferation_ImSig HMMR

Proliferation_ImSig KIAA0101

Proliferation_ImSig KIF11

Proliferation_ImSig KIF14

Proliferation_ImSig KIF15

Proliferation_ImSig KIF18B

Proliferation_ImSig KIF20A

Proliferation_ImSig KIF2C

Proliferation_ImSig KIF4A

Proliferation_ImSig MAD2L1

Proliferation_ImSig MCM10

Proliferation_ImSig MCM2

Proliferation_ImSig MCM4

Proliferation_ImSig MCM6

Proliferation_ImSig MELK

Proliferation_ImSig MKI67

Proliferation_ImSig MND1

Proliferation_ImSig MTFR2

Proliferation_ImSig NCAPG

Proliferation_ImSig NCAPG2

Proliferation_ImSig NDC80

Proliferation_ImSig NEK2

Proliferation_ImSig NUF2

Proliferation_ImSig NUSAP1

Proliferation_ImSig OIP5

Proliferation_ImSig PARPBP

Proliferation_ImSig PBK

Proliferation_ImSig PCNA

Proliferation_ImSig PLK4

Proliferation_ImSig POLE2

Proliferation_ImSig POLQ

Proliferation_ImSig PTTG1

Proliferation_ImSig RACGAP1

Proliferation_ImSig RAD51

Proliferation_ImSig RAD51AP1

Proliferation_ImSig RRM1

Proliferation_ImSig RRM2

Proliferation_ImSig SHCBP1

Proliferation_ImSig SKA1

Proliferation_ImSig SMC2

Proliferation_ImSig SPC25

Proliferation_ImSig STIL

Proliferation_ImSig STMN1

Proliferation_ImSig TCF19

Proliferation_ImSig TK1

Proliferation_ImSig TOP2A

Proliferation_ImSig TPX2

Proliferation_ImSig TRIP13

Proliferation_ImSig TTK

Proliferation_ImSig TYMS

Proliferation_ImSig UBE2C

Proliferation_ImSig UHRF1

Proliferation_ImSig ZWILCH

Proliferation_ImSig ZWINT

Translation_ImSig EEF1A1

Translation_ImSig EEF1B2

Translation_ImSig EEF1D

Translation_ImSig EEF1G

Translation_ImSig EIF3D

Translation_ImSig EIF3E

Translation_ImSig EIF3F

Translation_ImSig EIF3G

Translation_ImSig EIF3H

Translation_ImSig EIF3K

Translation_ImSig FAU

Translation_ImSig GNB2L1

Translation_ImSig NACA

Translation_ImSig PFDN5

Translation_ImSig RPL10

Translation_ImSig RPL10L

Translation_ImSig RPL11

Translation_ImSig RPL12

Translation_ImSig RPL13

Translation_ImSig RPL13A

Translation_ImSig RPL14

Translation_ImSig RPL15

Translation_ImSig RPL17

Translation_ImSig RPL18

Translation_ImSig RPL18A

Translation_ImSig RPL19

Translation_ImSig RPL21

Translation_ImSig RPL22

Translation_ImSig RPL23

Translation_ImSig RPL23A

Translation_ImSig RPL24

Translation_ImSig RPL27

Translation_ImSig RPL27A

Translation_ImSig RPL28

Translation_ImSig RPL29

Translation_ImSig RPL3

Translation_ImSig RPL30

Translation_ImSig RPL31

Translation_ImSig RPL32

Translation_ImSig RPL34

Translation_ImSig RPL35

Translation_ImSig RPL35A

Translation_ImSig RPL36A

Translation_ImSig RPL37

Translation_ImSig RPL37A

Translation_ImSig RPL38

Translation_ImSig RPL39

Translation_ImSig RPL4

Translation_ImSig RPL5

Translation_ImSig RPL6

Translation_ImSig RPL7

Translation_ImSig RPL7A

Translation_ImSig RPL8

Translation_ImSig RPL9

Translation_ImSig RPLP0

Translation_ImSig RPLP2

Translation_ImSig RPS10

Translation_ImSig RPS11

Translation_ImSig RPS13

Translation_ImSig RPS14

Translation_ImSig RPS15

Translation_ImSig RPS15A

Translation_ImSig RPS16

Translation_ImSig RPS17

Translation_ImSig RPS18

Translation_ImSig RPS19

Translation_ImSig RPS2

Translation_ImSig RPS20

Translation_ImSig RPS21

Translation_ImSig RPS23

Translation_ImSig RPS25

Translation_ImSig RPS27A

Translation_ImSig RPS28

Translation_ImSig RPS29

Translation_ImSig RPS3

Translation_ImSig RPS3A

Translation_ImSig RPS5

Translation_ImSig RPS6

Translation_ImSig RPS7

Translation_ImSig RPS8

Translation_ImSig RPS9

Translation_ImSig RPSA

Translation_ImSig SNHG6

Translation_ImSig SNHG8

Translation_ImSig SNRPD2

Translation_ImSig UXT

Antigen_Processing_and_Presentation AZGP1

Antigen_Processing_and_Presentation B2M

Antigen_Processing_and_Presentation CALR

Antigen_Processing_and_Presentation CANX

Antigen_Processing_and_Presentation CD1A

Antigen_Processing_and_Presentation CD1B

Antigen_Processing_and_Presentation CD1C

Antigen_Processing_and_Presentation CD1D

Antigen_Processing_and_Presentation CD1E

Antigen_Processing_and_Presentation CD4

Antigen_Processing_and_Presentation CD8A

Antigen_Processing_and_Presentation CD8B

Antigen_Processing_and_Presentation CD74

Antigen_Processing_and_Presentation CREB1

Antigen_Processing_and_Presentation CTSB

Antigen_Processing_and_Presentation CTSE

Antigen_Processing_and_Presentation CTSL1

Antigen_Processing_and_Presentation CTSS

Antigen_Processing_and_Presentation FCER1G

Antigen_Processing_and_Presentation FCGRT

Antigen_Processing_and_Presentation PDIA3

Antigen_Processing_and_Presentation HFE

Antigen_Processing_and_Presentation HLA-A

Antigen_Processing_and_Presentation HLA-B

Antigen_Processing_and_Presentation HLA-C

Antigen_Processing_and_Presentation HLA-DMA

Antigen_Processing_and_Presentation HLA-DMB

Antigen_Processing_and_Presentation HLA-DOA

Antigen_Processing_and_Presentation HLA-DOB

Antigen_Processing_and_Presentation HLA-DPA1

Antigen_Processing_and_Presentation HLA-DPB1

Antigen_Processing_and_Presentation HLA-DQA1

Antigen_Processing_and_Presentation HLA-DQA2

Antigen_Processing_and_Presentation HLA-DQB1

Antigen_Processing_and_Presentation HLA-DRA

Antigen_Processing_and_Presentation HLA-DRB1

Antigen_Processing_and_Presentation HLA-DRB3

Antigen_Processing_and_Presentation HLA-DRB4

Antigen_Processing_and_Presentation HLA-DRB5

Antigen_Processing_and_Presentation HLA-E

Antigen_Processing_and_Presentation HLA-F

Antigen_Processing_and_Presentation HLA-G

Antigen_Processing_and_Presentation HLA-H

Antigen_Processing_and_Presentation MR1

Antigen_Processing_and_Presentation HSPA1A

Antigen_Processing_and_Presentation HSPA1B

Antigen_Processing_and_Presentation HSPA1L

Antigen_Processing_and_Presentation HSPA2

Antigen_Processing_and_Presentation HSPA4

Antigen_Processing_and_Presentation HSPA5

Antigen_Processing_and_Presentation HSPA6

Antigen_Processing_and_Presentation HSPA8

Antigen_Processing_and_Presentation HSP90AA1

Antigen_Processing_and_Presentation HSP90AB1

Antigen_Processing_and_Presentation ICAM1

Antigen_Processing_and_Presentation IFNA1

Antigen_Processing_and_Presentation IFNA2

Antigen_Processing_and_Presentation IFNA4

Antigen_Processing_and_Presentation IFNA5

Antigen_Processing_and_Presentation IFNA6

Antigen_Processing_and_Presentation IFNA7

Antigen_Processing_and_Presentation IFNA8

Antigen_Processing_and_Presentation IFNA10

Antigen_Processing_and_Presentation IFNA13

Antigen_Processing_and_Presentation IFNA14

Antigen_Processing_and_Presentation IFNA16

Antigen_Processing_and_Presentation IFNA17

Antigen_Processing_and_Presentation IFNA21

Antigen_Processing_and_Presentation IFNG

Antigen_Processing_and_Presentation KIR2DL1

Antigen_Processing_and_Presentation KIR2DL2

Antigen_Processing_and_Presentation KIR2DL3

Antigen_Processing_and_Presentation KIR2DL4

Antigen_Processing_and_Presentation KIR2DS1

Antigen_Processing_and_Presentation KIR2DS3

Antigen_Processing_and_Presentation KIR2DS4

Antigen_Processing_and_Presentation KIR2DS5

Antigen_Processing_and_Presentation KIR3DL1

Antigen_Processing_and_Presentation KIR3DL2

Antigen_Processing_and_Presentation KLRC1

Antigen_Processing_and_Presentation KLRC2

Antigen_Processing_and_Presentation KLRC3

Antigen_Processing_and_Presentation KLRD1

Antigen_Processing_and_Presentation LTA

Antigen_Processing_and_Presentation CIITA

Antigen_Processing_and_Presentation MICA

Antigen_Processing_and_Presentation MICB

Antigen_Processing_and_Presentation NFYA

Antigen_Processing_and_Presentation NFYB

Antigen_Processing_and_Presentation NFYC

Antigen_Processing_and_Presentation LGMN

Antigen_Processing_and_Presentation PSMB8

Antigen_Processing_and_Presentation PSMC1

Antigen_Processing_and_Presentation PSMC2

Antigen_Processing_and_Presentation PSMC3

Antigen_Processing_and_Presentation PSMC4

Antigen_Processing_and_Presentation PSMC5

Antigen_Processing_and_Presentation PSMC6

Antigen_Processing_and_Presentation PSMD1

Antigen_Processing_and_Presentation PSMD2

Antigen_Processing_and_Presentation PSMD3

Antigen_Processing_and_Presentation PSMD4

Antigen_Processing_and_Presentation PSMD5

Antigen_Processing_and_Presentation PSMD7

Antigen_Processing_and_Presentation PSMD8

Antigen_Processing_and_Presentation PSMD10

Antigen_Processing_and_Presentation PSMD11

Antigen_Processing_and_Presentation PSMD13

Antigen_Processing_and_Presentation PSME1

Antigen_Processing_and_Presentation PSME2

Antigen_Processing_and_Presentation RELB

Antigen_Processing_and_Presentation RFX5

Antigen_Processing_and_Presentation RFXAP

Antigen_Processing_and_Presentation SLC10A2

Antigen_Processing_and_Presentation TAP1

Antigen_Processing_and_Presentation TAP2

Antigen_Processing_and_Presentation TAPBP

Antigen_Processing_and_Presentation THBS1

Antigen_Processing_and_Presentation SHFM1

Antigen_Processing_and_Presentation KLRC4

Antigen_Processing_and_Presentation AP3B1

Antigen_Processing_and_Presentation RFXANK

Antigen_Processing_and_Presentation PSMD6

Antigen_Processing_and_Presentation PSME3

Antigen_Processing_and_Presentation PSMD14

Antigen_Processing_and_Presentation CLEC4M

Antigen_Processing_and_Presentation IFI30

Antigen_Processing_and_Presentation PROCR

Antigen_Processing_and_Presentation ADRM1

Antigen_Processing_and_Presentation KIAA0368

Antigen_Processing_and_Presentation TRPC4AP

Antigen_Processing_and_Presentation CD209

Antigen_Processing_and_Presentation UBXN1

Antigen_Processing_and_Presentation ERAP1

Antigen_Processing_and_Presentation TAPBPL

Antigen_Processing_and_Presentation KIR2DL5A

Antigen_Processing_and_Presentation ERAP2

Antigen_Processing_and_Presentation ULBP3

Antigen_Processing_and_Presentation ULBP2

Antigen_Processing_and_Presentation ULBP1

Antigen_Processing_and_Presentation KIR3DL3

Antigen_Processing_and_Presentation RAET1E

Antigen_Processing_and_Presentation RAET1L

Antigen_Processing_and_Presentation UBR1

Antigen_Processing_and_Presentation RAET1G

Antigen_Processing_and_Presentation PDIA2

Antimicrobials HAMP

Antimicrobials PI3

Antimicrobials CAMP

Antimicrobials DEFB4

Antimicrobials PPBP

Antimicrobials REG3G

Antimicrobials CXCL14

Antimicrobials CXCL16

Antimicrobials SLPI

Antimicrobials IL8

Antimicrobials CXCL10

Antimicrobials CXCL9

Antimicrobials CXCL5

Antimicrobials CXCL11

Antimicrobials CXCL6

Antimicrobials CXCL1

Antimicrobials CXCL12

Antimicrobials CXCL13

Antimicrobials CXCL2

Antimicrobials PF4

Antimicrobials XCL1

Antimicrobials CXCL3

Antimicrobials DEFB103A

Antimicrobials CCL13

Antimicrobials CCL1

Antimicrobials DEFB1

Antimicrobials CCL8

Antimicrobials ELANE

Antimicrobials DEFB103B

Antimicrobials DEFA3

Antimicrobials DEFA1

Antimicrobials TMSB10

Antimicrobials DEFA6

Antimicrobials DEFA5

Antimicrobials DEFA4

Antimicrobials LCN2

Antimicrobials LCN1

Antimicrobials COLEC10

Antimicrobials BPI

Antimicrobials S100A9

Antimicrobials S100A8

Antimicrobials DCD

Antimicrobials LCN6

Antimicrobials S100A12

Antimicrobials HTN3

Antimicrobials LCN8

Antimicrobials LOC728358

Antimicrobials CCR10

Antimicrobials CELA1

Antimicrobials DEFB106A

Antimicrobials PENK

Antimicrobials BPIL2

Antimicrobials MMP12

Antimicrobials BPIL3

Antimicrobials LEAP2

Antimicrobials SFTPD

Antimicrobials LCN9

Antimicrobials BPIL1

Antimicrobials PTGDS

Antimicrobials TMSB4X

Antimicrobials PGLYRP1

Antimicrobials ZC3HAV1

Antimicrobials TMSB15A

Antimicrobials S100B

Antimicrobials S100A13

Antimicrobials S100A6

Antimicrobials DEFB119

Antimicrobials DEFB107A

Antimicrobials DEFB105A

Antimicrobials SERPIND1

Antimicrobials DEFB129

Antimicrobials DEFB127

Antimicrobials S100P

Antimicrobials S100A7

Antimicrobials DEFB104A

Antimicrobials DEFB126

Antimicrobials DEFB106B

Antimicrobials DEFB104B

Antimicrobials DEFB107B

Antimicrobials PGLYRP3

Antimicrobials PGLYRP2

Antimicrobials S100A10

Antimicrobials S100A2

Antimicrobials DEFB125

Antimicrobials DEFB123

Antimicrobials DEFB105B

Antimicrobials DEFB132

Antimicrobials C20orf185

Antimicrobials LCN12

Antimicrobials PGLYRP4

Antimicrobials S100A11

Antimicrobials S100A5

Antimicrobials S100A3

Antimicrobials S100A1

Antimicrobials DEFB128

Antimicrobials DEFB108B

Antimicrobials HTN1

Antimicrobials LMBR1L

Antimicrobials S100A7A

Antimicrobials DEFB118

Antimicrobials COLEC12

Antimicrobials TMSB4Y

Antimicrobials DEFB131

Antimicrobials DEFB134

Antimicrobials DEFB130

Antimicrobials DEFB124

Antimicrobials DEFB121

Antimicrobials DEFB116

Antimicrobials DEFB115

Antimicrobials DEFB114

Antimicrobials DEFB113

Antimicrobials DEFB112

Antimicrobials DEFB110

Antimicrobials TMSB15B

Antimicrobials DEFB133

Antimicrobials S100Z

Antimicrobials MAVS

Antimicrobials TMSL3

Antimicrobials S100A14

Antimicrobials LCN10

Antimicrobials S100A16

Antimicrobials DEFB137

Antimicrobials DEFB136

Antimicrobials DEFB117

Antimicrobials DEFB111

Antimicrobials ZC3HAV1L

Antimicrobials S100A7L2

Antimicrobials LOC731414

Antimicrobials LOC730963

Antimicrobials COLEC2

Antimicrobials DEFB4P

Antimicrobials C20orf186

Antimicrobials IFNAR1

Antimicrobials AZU1

Antimicrobials LOC729523

Antimicrobials LOC100130154

Antimicrobials LOC100134379

Antimicrobials LOC100134289

Antimicrobials LOC100129216

Antimicrobials DEFA1A3

Antimicrobials LOC100131433

Antimicrobials LCN1L1

Antimicrobials S100G

Antimicrobials LOC648637

Antimicrobials LOC100130969

Antimicrobials LOC100133267

Antimicrobials LOC100133128

Antimicrobials LOC100128174

Antimicrobials TCHHL1

Antimicrobials TINAGL1

Antimicrobials IFNGR1

Antimicrobials SLC22A17

Antimicrobials WFIKKN1

Antimicrobials WFDC2

Antimicrobials IL6

Antimicrobials UMODL1

Antimicrobials TGFB1

Antimicrobials PF4V1

Antimicrobials MMP9

Antimicrobials KAL1

Antimicrobials TLR4

Antimicrobials IFNG

Antimicrobials SPAG11B

Antimicrobials A2M

Antimicrobials CTSL1

Antimicrobials NFKB1

Antimicrobials APOBEC3G

Antimicrobials FABP6

Antimicrobials NOD2

Antimicrobials MBL2

Antimicrobials SFTPA1B

Antimicrobials RBP1

Antimicrobials TLR2

Antimicrobials SLC40A1

Antimicrobials PLAU

Antimicrobials IL1B

Antimicrobials PAEP

Antimicrobials HFE2

Antimicrobials MUC5AC

Antimicrobials CTSS

Antimicrobials OBP2A

Antimicrobials PLTP

Antimicrobials MX1

Antimicrobials DDX58

Antimicrobials IL29

Antimicrobials IRF3

Antimicrobials SFTPA2

Antimicrobials SFTPA2B

Antimicrobials LPA

Antimicrobials LBP

Antimicrobials RBP4

Antimicrobials SFTPA1

Antimicrobials NOX4

Antimicrobials LTF

Antimicrobials IFNB1

Antimicrobials RBP5

Antimicrobials FABP7

Antimicrobials FABP5

Antimicrobials FABP3

Antimicrobials FABP2

Antimicrobials FABP4

Antimicrobials R3HDML

Antimicrobials C20orf71

Antimicrobials C20orf114

Antimicrobials OASL

Antimicrobials CRABP2

Antimicrobials CRABP1

Antimicrobials RBP7

Antimicrobials DUOX1

Antimicrobials OBP2B

Antimicrobials RBP2

Antimicrobials LCN15

Antimicrobials CETP

Antimicrobials FABP12

Antimicrobials FABP9

Antimicrobials PLUNC

Antimicrobials LCNL1

Antimicrobials C8G

Antimicrobials SPAG11A

Antimicrobials PI15

Antimicrobials NOX1

Antimicrobials PMP2

Antimicrobials APOD

Antimicrobials ORM2

Antimicrobials ORM1

Antimicrobials TNF

Antimicrobials CTSG

Antimicrobials PRTN3

Antimicrobials MAPK1

Antimicrobials PML

Antimicrobials AEN

Antimicrobials CYBB

Antimicrobials C20orf70

Antimicrobials ISG20

Antimicrobials BCL3

Antimicrobials ISG20L2

Antimicrobials NOX5

Antimicrobials NOX3

Antimicrobials DUOX2

Antimicrobials TLR3

Antimicrobials TFRC

Antimicrobials IFIH1

Antimicrobials LRP1

Antimicrobials TRIM5

Antimicrobials IDO1

Antimicrobials GDF15

Antimicrobials NEDD4

Antimicrobials ADIPOQ

Antimicrobials STAT3

Antimicrobials STAT1

Antimicrobials IL28A

Antimicrobials SOCS3

Antimicrobials SEMG1

Antimicrobials TNFSF10

Antimicrobials CCL20

Antimicrobials SOCS1

Antimicrobials RNASEL

Antimicrobials IRF1

Antimicrobials IL15

Antimicrobials APOBEC3F

Antimicrobials RARRES3

Antimicrobials CHIT1

Antimicrobials IFNA1

Antimicrobials CD40

Antimicrobials TLR7

Antimicrobials PPIA

Antimicrobials HFE

Antimicrobials ZYX

Antimicrobials NLRX1

Antimicrobials PGC

Antimicrobials VEGFA

Antimicrobials IKBKE

Antimicrobials ISG15

Antimicrobials DHX58

Antimicrobials TNFAIP3

Antimicrobials TFR2

Antimicrobials FCN2

Antimicrobials MUC4

Antimicrobials F2R

Antimicrobials ELN

Antimicrobials IL27

Antimicrobials MAPT

Antimicrobials LYZ

Antimicrobials CCL5

Antimicrobials LEP

Antimicrobials CYLD

Antimicrobials KLKB1

Antimicrobials CST4

Antimicrobials CSRP1

Antimicrobials MAPK14

Antimicrobials JUN

Antimicrobials ITGAV

Antimicrobials IRF5

Antimicrobials CCR6

Antimicrobials IL12B

Antimicrobials TLR8

Antimicrobials GNLY

Antimicrobials CD81

Antimicrobials EIF2AK2

Antimicrobials APOM

Antimicrobials CACYBP

Antimicrobials NOD1

Antimicrobials MAPK8

Antimicrobials MAPK3

Antimicrobials BST2

Antimicrobials BPHL

Antimicrobials PLA2G2A

Antimicrobials GRN

Antimicrobials NEWENTRY

Antimicrobials PDGFRA

Antimicrobials GNAI1

Antimicrobials WNT5A

Antimicrobials FURIN

Antimicrobials ADAR

Antimicrobials TYK2

Antimicrobials NOS2

Antimicrobials TRAF3

Antimicrobials TPT1

Antimicrobials TPM2

Antimicrobials NEO1

Antimicrobials AHNAK

Antimicrobials TLR1

Antimicrobials TK2

Antimicrobials PRDX2

Antimicrobials MX2

Antimicrobials FGF2

Antimicrobials FGA

Antimicrobials TCF7L2

Antimicrobials F2RL1

Antimicrobials DAK

Antimicrobials MSR1

Antimicrobials NFKBIZ

Antimicrobials LMBR1

Antimicrobials SPINLW1

Antimicrobials SRC

Antimicrobials MPO

Antimicrobials ELAVL1

Antimicrobials ROBO3

Antimicrobials SP1

Antimicrobials SOD1

Antimicrobials PDF

Antimicrobials DLL4

Antimicrobials ECD

Antimicrobials SLC11A1

Antimicrobials DMBT1

Antimicrobials TMEM173

Antimicrobials SKIV2L

Antimicrobials SEMG2

Antimicrobials LTA

Antimicrobials DES

Antimicrobials DCK

Antimicrobials DAXX

Antimicrobials TNFRSF10A

Antimicrobials TNFRSF10B

Antimicrobials EED

Antimicrobials CCL4

Antimicrobials LIMS1

Antimicrobials LALBA

Antimicrobials APOBEC3H

Antimicrobials TMPRSS6

Antimicrobials SPINK5

Antimicrobials MARCO

Antimicrobials BECN1

Antimicrobials TNFSF11

Antimicrobials KNG1

Antimicrobials CSK

Antimicrobials KLRK1

Antimicrobials KCNH2

Antimicrobials JUND

Antimicrobials JAK1

Antimicrobials CREB1

Antimicrobials CLDN4

Antimicrobials CCL28

Antimicrobials RNASE3

Antimicrobials RN7SL1

Antimicrobials IRF7

Antimicrobials IREB2

Antimicrobials ILK

Antimicrobials IL18

Antimicrobials IL17A

Antimicrobials LTB4R

Antimicrobials APOBEC3A

Antimicrobials MASP2

Antimicrobials TRIM27

Antimicrobials RELA

Antimicrobials IL7R

Antimicrobials IL1A

Antimicrobials PTX3

Antimicrobials IFNAR2

Antimicrobials IFN1@

Antimicrobials SYTL1

Antimicrobials APOBEC3C

Antimicrobials DDX17

Antimicrobials PTGS2

Antimicrobials HTR1A

Antimicrobials 7-Sep

Antimicrobials CD40LG

Antimicrobials CD14

Antimicrobials CD8A

Antimicrobials CD4

Antimicrobials MASP1

Antimicrobials PROC

Antimicrobials MAP2K2

Antimicrobials MAP2K1

Antimicrobials HRG

Antimicrobials NDRG1

Antimicrobials IRF9

Antimicrobials TRIM22

Antimicrobials LANCL1

Antimicrobials PPP4C

Antimicrobials HMOX1

Antimicrobials HMGB1

Antimicrobials HLA-B

Antimicrobials RNASE7

Antimicrobials ABCC4

Antimicrobials HGF

Antimicrobials HDAC1

Antimicrobials IL28RA

Antimicrobials PLSCR1

Antimicrobials B2M

Antimicrobials BACH2

Antimicrobials TANK

Antimicrobials PIK3CG

Antimicrobials ARRB1

Antimicrobials RSAD2

Antimicrobials STAB2

Antimicrobials TBK1

Antimicrobials PDYN

Antimicrobials PDGFRB

Antimicrobials PDCD1

Antimicrobials PCSK2

Antimicrobials PCSK1

Antimicrobials ARG2

Antimicrobials AQP9

Antimicrobials FASLG

Antimicrobials APOH

Antimicrobials BIRC5

Antimicrobials ANXA6

Antimicrobials IL22

Antimicrobials VTN

Antimicrobials VIM

Antimicrobials VCAM1

Antimicrobials PRDX1

Antimicrobials GFAP

Antimicrobials GBP2

Antimicrobials ALB

Antimicrobials SLC29A3

Antimicrobials OAS1

Antimicrobials AGER

Antimicrobials UNC93B1

Antimicrobials TNFSF4

Antimicrobials NOS1

Antimicrobials ACTG1

Antimicrobials ACTA1

Antimicrobials ACO1

Antimicrobials SERPINA3

Antimicrobials IL8RA

Antimicrobials CCL15

Antimicrobials CCL14

Antimicrobials CCL16

Antimicrobials CCL19

Antimicrobials CCL18

Antimicrobials CCL17

Antimicrobials CCL26

Antimicrobials CCL22

Antimicrobials CCR3

Antimicrobials CCL4L2

Antimicrobials CCBP2

Antimicrobials CCR7

Antimicrobials CCL27

Antimicrobials CCR8

Antimicrobials CCRL1

Antimicrobials CCL2

Antimicrobials CCL21

Antimicrobials CCL7

Antimicrobials CCL3

Antimicrobials CCL11

Antimicrobials CCR5

Antimicrobials CCL23

Antimicrobials CCL25

Antimicrobials CCL3L3

Antimicrobials CCL4L1

Antimicrobials CCL3L1

Antimicrobials CCR1

Antimicrobials CCL24

Antimicrobials XCL2

Antimicrobials CXCR4

Antimicrobials CXCR6

Antimicrobials CCR4

Antimicrobials FAM19A5

Antimicrobials FAM19A3

Antimicrobials FAM19A4

Antimicrobials FAM19A1

Antimicrobials FAM19A2

Antimicrobials CCL14-CCL15

Antimicrobials PTK2B

Antimicrobials IL4

Antimicrobials CDH1

Antimicrobials LTBP1

Antimicrobials IL13

Antimicrobials IL10

Antimicrobials IL2

Antimicrobials PPARG

Antimicrobials FGR

Antimicrobials MIF

Antimicrobials CRP

Antimicrobials JAK2

Antimicrobials PTK2

Antimicrobials PTGDR

Antimicrobials CD86

Antimicrobials HCK

Antimicrobials VDR

Antimicrobials OLR1

Antimicrobials ADRBK1

Antimicrobials TXK

Antimicrobials RNASE2

BCRSignalingPathway CD79A

BCRSignalingPathway CD79B

BCRSignalingPathway LYN

BCRSignalingPathway SYK

BCRSignalingPathway BTK

BCRSignalingPathway BLNK

BCRSignalingPathway VAV3

BCRSignalingPathway VAV1

BCRSignalingPathway VAV2

BCRSignalingPathway RAC1

BCRSignalingPathway RAC2

BCRSignalingPathway RAC3

BCRSignalingPathway PPP3CA

BCRSignalingPathway PPP3CB

BCRSignalingPathway PPP3CC

BCRSignalingPathway CHP

BCRSignalingPathway PPP3R1

BCRSignalingPathway PPP3R2

BCRSignalingPathway CHP2

BCRSignalingPathway NFAT5

BCRSignalingPathway NFATC1

BCRSignalingPathway NFATC2

BCRSignalingPathway NFATC3

BCRSignalingPathway NFATC4

BCRSignalingPathway HRAS

BCRSignalingPathway KRAS

BCRSignalingPathway NRAS

BCRSignalingPathway FOS

BCRSignalingPathway JUN

BCRSignalingPathway CARD11

BCRSignalingPathway BCL10

BCRSignalingPathway MALT1

BCRSignalingPathway CHUK

BCRSignalingPathway IKBKB

BCRSignalingPathway IKBKG

BCRSignalingPathway NFKB1

BCRSignalingPathway RELA

BCRSignalingPathway NFKBIA

BCRSignalingPathway NFKBIB

BCRSignalingPathway NFKBIE

BCRSignalingPathway CD81

BCRSignalingPathway CD19

BCRSignalingPathway CR2

BCRSignalingPathway PIK3R5

BCRSignalingPathway PIK3R1

BCRSignalingPathway PIK3R2

BCRSignalingPathway PIK3R3

BCRSignalingPathway PIK3CA

BCRSignalingPathway PIK3CB

BCRSignalingPathway PIK3CD

BCRSignalingPathway PIK3CG

BCRSignalingPathway AKT3

BCRSignalingPathway AKT1

BCRSignalingPathway AKT2

BCRSignalingPathway GSK3B

BCRSignalingPathway INPP5D

BCRSignalingPathway CD22

BCRSignalingPathway CD72

BCRSignalingPathway PTPN6

BCRSignalingPathway LILRB3

BCRSignalingPathway FCGR2B

BCRSignalingPathway RASGRP3

BCRSignalingPathway PLCG2

BCRSignalingPathway PRKCB

BCRSignalingPathway IFITM1

BCRSignalingPathway IGH@

BCRSignalingPathway IGHA1

BCRSignalingPathway IGHA2

BCRSignalingPathway IGHD

BCRSignalingPathway IGHD@

BCRSignalingPathway IGHD1-1

BCRSignalingPathway IGHD1-14

BCRSignalingPathway IGHD1-20

BCRSignalingPathway IGHD1-26

BCRSignalingPathway IGHD1-7

BCRSignalingPathway IGHD2-15

BCRSignalingPathway IGHD2-2

BCRSignalingPathway IGHD2-21

BCRSignalingPathway IGHD2-8

BCRSignalingPathway IGHD3-10

BCRSignalingPathway IGHD3-16

BCRSignalingPathway IGHD3-22

BCRSignalingPathway IGHD3-3

BCRSignalingPathway IGHD3-9

BCRSignalingPathway IGHD4-11

BCRSignalingPathway IGHD4-17

BCRSignalingPathway IGHD4-23

BCRSignalingPathway IGHD4-4

BCRSignalingPathway IGHD5-12

BCRSignalingPathway IGHD5-18

BCRSignalingPathway IGHD5-24

BCRSignalingPathway IGHD5-5

BCRSignalingPathway IGHD6-13

BCRSignalingPathway IGHD6-19

BCRSignalingPathway IGHD6-25

BCRSignalingPathway IGHD6-6

BCRSignalingPathway IGHD7-27

BCRSignalingPathway IGHE

BCRSignalingPathway IGHG1

BCRSignalingPathway IGHG2

BCRSignalingPathway IGHG3

BCRSignalingPathway IGHG4

BCRSignalingPathway IGHJ@

BCRSignalingPathway IGHJ1

BCRSignalingPathway IGHJ2

BCRSignalingPathway IGHJ3

BCRSignalingPathway IGHJ4

BCRSignalingPathway IGHJ5

BCRSignalingPathway IGHJ6

BCRSignalingPathway IGHM

BCRSignalingPathway IGHV@

BCRSignalingPathway IGHV1-18

BCRSignalingPathway IGHV1-2

BCRSignalingPathway IGHV1-24

BCRSignalingPathway IGHV1-3

BCRSignalingPathway IGHV1-45

BCRSignalingPathway IGHV1-46

BCRSignalingPathway IGHV1-58

BCRSignalingPathway IGHV1-69

BCRSignalingPathway IGHV1-8

BCRSignalingPathway IGHV1-C

BCRSignalingPathway IGHV1-F

BCRSignalingPathway IGHV2-26

BCRSignalingPathway IGHV2-5

BCRSignalingPathway IGHV2-70

BCRSignalingPathway IGHV3-11

BCRSignalingPathway IGHV3-13

BCRSignalingPathway IGHV3-15

BCRSignalingPathway IGHV3-16

BCRSignalingPathway IGHV3-20

BCRSignalingPathway IGHV3-21

BCRSignalingPathway IGHV3-23

BCRSignalingPathway IGHV3-30

BCRSignalingPathway IGHV3-30-3

BCRSignalingPathway IGHV3-30-5

BCRSignalingPathway IGHV3-33

BCRSignalingPathway IGHV3-35

BCRSignalingPathway IGHV3-38

BCRSignalingPathway IGHV3-43

BCRSignalingPathway IGHV3-48

BCRSignalingPathway IGHV3-49

BCRSignalingPathway IGHV3-53

BCRSignalingPathway IGHV3-64

BCRSignalingPathway IGHV3-66

BCRSignalingPathway IGHV3-7

BCRSignalingPathway IGHV3-72

BCRSignalingPathway IGHV3-73

BCRSignalingPathway IGHV3-74

BCRSignalingPathway IGHV3-9

BCRSignalingPathway IGHV3-D

BCRSignalingPathway IGHV3-H

BCRSignalingPathway IGHV4-28

BCRSignalingPathway IGHV4-30-1

BCRSignalingPathway IGHV4-30-2

BCRSignalingPathway IGHV4-30-4

BCRSignalingPathway IGHV4-31

BCRSignalingPathway IGHV4-34

BCRSignalingPathway IGHV4-39

BCRSignalingPathway IGHV4-4

BCRSignalingPathway IGHV4-59

BCRSignalingPathway IGHV4-61

BCRSignalingPathway IGHV4-B

BCRSignalingPathway IGHV5-51

BCRSignalingPathway IGHV5-A

BCRSignalingPathway IGHV6-1

BCRSignalingPathway IGHV7-4-1

BCRSignalingPathway IGHV7-81

BCRSignalingPathway IGK@

BCRSignalingPathway IGKC

BCRSignalingPathway IGKDEL

BCRSignalingPathway IGKJ@

BCRSignalingPathway IGKJ1

BCRSignalingPathway IGKJ2

BCRSignalingPathway IGKJ3

BCRSignalingPathway IGKJ4

BCRSignalingPathway IGKJ5

BCRSignalingPathway IGKV@

BCRSignalingPathway IGKV1-12

BCRSignalingPathway IGKV1-13

BCRSignalingPathway IGKV1-16

BCRSignalingPathway IGKV1-17

BCRSignalingPathway IGKV1-27

BCRSignalingPathway IGKV1-33

BCRSignalingPathway IGKV1-37

BCRSignalingPathway IGKV1-39

BCRSignalingPathway IGKV1-5

BCRSignalingPathway IGKV1-6

BCRSignalingPathway IGKV1-8

BCRSignalingPathway IGKV1-9

BCRSignalingPathway IGKV1D-12

BCRSignalingPathway IGKV1D-13

BCRSignalingPathway IGKV1D-16

BCRSignalingPathway IGKV1D-17

BCRSignalingPathway IGKV1D-33

BCRSignalingPathway IGKV1D-37

BCRSignalingPathway IGKV1D-39

BCRSignalingPathway IGKV1D-42

BCRSignalingPathway IGKV1D-43

BCRSignalingPathway IGKV1D-8

BCRSignalingPathway IGKV2-24

BCRSignalingPathway IGKV2-28

BCRSignalingPathway IGKV2-30

BCRSignalingPathway IGKV2-40

BCRSignalingPathway IGKV2D-24

BCRSignalingPathway IGKV2D-28

BCRSignalingPathway IGKV2D-29

BCRSignalingPathway IGKV2D-30

BCRSignalingPathway IGKV2D-40

BCRSignalingPathway IGKV3-11

BCRSignalingPathway IGKV3-15

BCRSignalingPathway IGKV3-20

BCRSignalingPathway IGKV3-7

BCRSignalingPathway IGKV3D-11

BCRSignalingPathway IGKV3D-15

BCRSignalingPathway IGKV3D-20

BCRSignalingPathway IGKV3D-7

BCRSignalingPathway IGKV4-1

BCRSignalingPathway IGKV5-2

BCRSignalingPathway IGKV6-21

BCRSignalingPathway IGKV6D-21

BCRSignalingPathway IGKV6D-41

BCRSignalingPathway IGL@

BCRSignalingPathway IGLC@

BCRSignalingPathway IGLC1

BCRSignalingPathway IGLC2

BCRSignalingPathway IGLC3

BCRSignalingPathway IGLC6

BCRSignalingPathway IGLC7

BCRSignalingPathway IGLJ@

BCRSignalingPathway IGLJ1

BCRSignalingPathway IGLJ2

BCRSignalingPathway IGLJ3

BCRSignalingPathway IGLJ4

BCRSignalingPathway IGLJ5

BCRSignalingPathway IGLJ6

BCRSignalingPathway IGLJ7

BCRSignalingPathway IGLV@

BCRSignalingPathway IGLV1-36

BCRSignalingPathway IGLV1-40

BCRSignalingPathway IGLV1-44

BCRSignalingPathway IGLV1-47

BCRSignalingPathway IGLV1-50

BCRSignalingPathway IGLV1-51

BCRSignalingPathway IGLV10-54

BCRSignalingPathway IGLV11-55

BCRSignalingPathway IGLV2-11

BCRSignalingPathway IGLV2-14

BCRSignalingPathway IGLV2-18

BCRSignalingPathway IGLV2-23

BCRSignalingPathway IGLV2-33

BCRSignalingPathway IGLV2-8

BCRSignalingPathway IGLV3-1

BCRSignalingPathway IGLV3-10

BCRSignalingPathway IGLV3-12

BCRSignalingPathway IGLV3-16

BCRSignalingPathway IGLV3-19

BCRSignalingPathway IGLV3-21

BCRSignalingPathway IGLV3-22

BCRSignalingPathway IGLV3-25

BCRSignalingPathway IGLV3-27

BCRSignalingPathway IGLV3-32

BCRSignalingPathway IGLV3-9

BCRSignalingPathway IGLV4-3

BCRSignalingPathway IGLV4-60

BCRSignalingPathway IGLV4-69

BCRSignalingPathway IGLV5-37

BCRSignalingPathway IGLV5-39

BCRSignalingPathway IGLV5-45

BCRSignalingPathway IGLV5-48

BCRSignalingPathway IGLV5-52

BCRSignalingPathway IGLV6-57

BCRSignalingPathway IGLV7-43

BCRSignalingPathway IGLV7-46

BCRSignalingPathway IGLV8-61

BCRSignalingPathway IGLV9-49

Chemokines C3

Chemokines C5

Chemokines CAMP

Chemokines CCL1

Chemokines CCL11

Chemokines CCL13

Chemokines CCL14

Chemokines CCL14-CCL15

Chemokines CCL15

Chemokines CCL16

Chemokines CCL17

Chemokines CCL18

Chemokines CCL19

Chemokines CCL2

Chemokines CCL20

Chemokines CCL21

Chemokines CCL22

Chemokines CCL23

Chemokines CCL24

Chemokines CCL25

Chemokines CCL26

Chemokines CCL27

Chemokines CCL28

Chemokines CCL3

Chemokines CCL3L1

Chemokines CCL3L2

Chemokines CCL3L3

Chemokines CCL4

Chemokines CCL4L1

Chemokines CCL4L2

Chemokines CCL5

Chemokines CCL7

Chemokines CCL8

Chemokines CKLF

Chemokines CMA1

Chemokines CTSG

Chemokines CX3CL1

Chemokines CXCL1

Chemokines CXCL10

Chemokines CXCL11

Chemokines CXCL12

Chemokines CXCL13

Chemokines CXCL14

Chemokines CXCL16

Chemokines CXCL17

Chemokines CXCL2

Chemokines CXCL3

Chemokines CXCL5

Chemokines CXCL6

Chemokines CXCL9

Chemokines CYR61

Chemokines DEFA1

Chemokines DEFA3

Chemokines DEFA5

Chemokines DEFB1

Chemokines DEFB103A

Chemokines DEFB104A

Chemokines DEFB4

Chemokines EDN1

Chemokines EDN2

Chemokines EDN3

Chemokines FGF10

Chemokines FGF2

Chemokines HTN3

Chemokines IL8

Chemokines LECT2

Chemokines PF4

Chemokines PF4V1

Chemokines PLAU

Chemokines PPBP

Chemokines PPBPL1

Chemokines PROK2

Chemokines RNASE2

Chemokines SAA1

Chemokines SAA2

Chemokines SBDS

Chemokines SEMA3A

Chemokines SEMA3B

Chemokines SEMA3C

Chemokines SEMA3D

Chemokines SEMA3E

Chemokines SEMA3F

Chemokines SEMA3G

Chemokines SEMA4A

Chemokines SEMA4B

Chemokines SEMA4C

Chemokines SEMA4D

Chemokines SEMA4F

Chemokines SEMA4G

Chemokines SEMA5A

Chemokines SEMA5B

Chemokines SEMA6A

Chemokines SEMA6B

Chemokines SEMA6C

Chemokines SEMA6D

Chemokines SEMA7A

Chemokines SLIT1

Chemokines SLIT2

Chemokines TNC

Chemokines TYMP

Chemokines XCL1

Chemokines XCL2

Chemokine_Receptors C5AR1

Chemokine_Receptors CCBP2

Chemokine_Receptors CCR1

Chemokine_Receptors CCR10

Chemokine_Receptors CCR3

Chemokine_Receptors CCR4

Chemokine_Receptors CCR5

Chemokine_Receptors CCR6

Chemokine_Receptors CCR7

Chemokine_Receptors CCR8

Chemokine_Receptors CCR9

Chemokine_Receptors CCRL1

Chemokine_Receptors CCRL2

Chemokine_Receptors CMKLR1

Chemokine_Receptors CX3CR1

Chemokine_Receptors CXCR3

Chemokine_Receptors CXCR4

Chemokine_Receptors CXCR5

Chemokine_Receptors CXCR6

Chemokine_Receptors CXCR7

Chemokine_Receptors CYSLTR1

Chemokine_Receptors CYSLTR2

Chemokine_Receptors DARC

Chemokine_Receptors EDNRA

Chemokine_Receptors EDNRB

Chemokine_Receptors FPR1

Chemokine_Receptors FPR2

Chemokine_Receptors GPR17

Chemokine_Receptors GPR32

Chemokine_Receptors GPR33

Chemokine_Receptors GPR44

Chemokine_Receptors GPR77

Chemokine_Receptors IL8RA

Chemokine_Receptors IL8RB

Chemokine_Receptors LTB4R

Chemokine_Receptors LTB4R2

Chemokine_Receptors PLAUR

Chemokine_Receptors PLXNA1

Chemokine_Receptors PLXNA2

Chemokine_Receptors PLXNA3

Chemokine_Receptors PLXNA4

Chemokine_Receptors PLXNB1

Chemokine_Receptors PLXNB2

Chemokine_Receptors PLXNB3

Chemokine_Receptors PLXNC1

Chemokine_Receptors PLXND1

Chemokine_Receptors PTAFR

Chemokine_Receptors ROBO1

Chemokine_Receptors ROBO2

Chemokine_Receptors ROBO3

Chemokine_Receptors RXFP3

Chemokine_Receptors XCR1

Cytokines ADIPOQ

Cytokines ADM

Cytokines ADM2

Cytokines AGRP

Cytokines AGT

Cytokines AMBN

Cytokines AMELX

Cytokines AMH

Cytokines ANGPTL5

Cytokines ANGPTL7

Cytokines APLN

Cytokines AREG

Cytokines ARMET

Cytokines ARMETL1

Cytokines ARTN

Cytokines AVP

Cytokines AZU1

Cytokines BDNF

Cytokines BMP1

Cytokines BMP10

Cytokines BMP15

Cytokines BMP2

Cytokines BMP3

Cytokines BMP4

Cytokines BMP5

Cytokines BMP6

Cytokines BMP7

Cytokines BMP8A

Cytokines BMP8B

Cytokines BTC

Cytokines C19orf10

Cytokines C3

Cytokines C5

Cytokines CALCA

Cytokines CALCB

Cytokines CAMP

Cytokines CAT

Cytokines CCK

Cytokines CCL1

Cytokines CCL11

Cytokines CCL13

Cytokines CCL14

Cytokines CCL14-CCL15

Cytokines CCL15

Cytokines CCL16

Cytokines CCL17

Cytokines CCL18

Cytokines CCL19

Cytokines CCL2

Cytokines CCL20

Cytokines CCL21

Cytokines CCL22

Cytokines CCL23

Cytokines CCL24

Cytokines CCL25

Cytokines CCL26

Cytokines CCL27

Cytokines CCL28

Cytokines CCL3

Cytokines CCL3L1

Cytokines CCL3L2

Cytokines CCL3L3

Cytokines CCL4

Cytokines CCL4L1

Cytokines CCL4L2

Cytokines CCL5

Cytokines CCL7

Cytokines CCL8

Cytokines CD320

Cytokines CD40LG

Cytokines CD70

Cytokines CECR1

Cytokines CER1

Cytokines CGA

Cytokines CGB

Cytokines CGB1

Cytokines CGB2

Cytokines CGB5

Cytokines CGB7

Cytokines CGB8

Cytokines CHGA

Cytokines CHGB

Cytokines CKLF

Cytokines CLCF1

Cytokines CLEC11A

Cytokines CMA1

Cytokines CMTM1

Cytokines CMTM2

Cytokines CMTM3

Cytokines CMTM4

Cytokines CMTM5

Cytokines CMTM6

Cytokines CMTM7

Cytokines CMTM8

Cytokines CNTF

Cytokines CORT

Cytokines CRH

Cytokines CSF1

Cytokines CSF2

Cytokines CSF3

Cytokines CSH1

Cytokines CSH2

Cytokines CSHL1

Cytokines CSPG5

Cytokines CTF1

Cytokines CTGF

Cytokines CTSG

Cytokines CX3CL1

Cytokines CXCL1

Cytokines CXCL10

Cytokines CXCL11

Cytokines CXCL12

Cytokines CXCL13

Cytokines CXCL14

Cytokines CXCL16

Cytokines CXCL17

Cytokines CXCL2

Cytokines CXCL3

Cytokines CXCL5

Cytokines CXCL6

Cytokines CXCL9

Cytokines CYR61

Cytokines DEFA1

Cytokines DEFA3

Cytokines DEFA5

Cytokines DEFB1

Cytokines DEFB103A

Cytokines DEFB104A

Cytokines DEFB4

Cytokines DKK1

Cytokines EBI3

Cytokines EDN1

Cytokines EDN2

Cytokines EDN3

Cytokines EGF

Cytokines EPGN

Cytokines EPO

Cytokines EREG

Cytokines ESM1

Cytokines FAM3B

Cytokines FAM3C

Cytokines FAM3D

Cytokines FASLG

Cytokines FGF1

Cytokines FGF10

Cytokines FGF11

Cytokines FGF12

Cytokines FGF13

Cytokines FGF14

Cytokines FGF16

Cytokines FGF17

Cytokines FGF18

Cytokines FGF19

Cytokines FGF2

Cytokines FGF20

Cytokines FGF21

Cytokines FGF22

Cytokines FGF23

Cytokines FGF3

Cytokines FGF4

Cytokines FGF5

Cytokines FGF6

Cytokines FGF7

Cytokines FGF8

Cytokines FGF9

Cytokines FIGF

Cytokines FIGNL2

Cytokines FLT3LG

Cytokines FSHB

Cytokines GAL

Cytokines GALP

Cytokines GAST

Cytokines GCG

Cytokines GDF1

Cytokines GDF10

Cytokines GDF11

Cytokines GDF15

Cytokines GDF2

Cytokines GDF3

Cytokines GDF5

Cytokines GDF6

Cytokines GDF7

Cytokines GDF9

Cytokines GDNF

Cytokines GH1

Cytokines GH2

Cytokines GHRH

Cytokines GHRL

Cytokines GIP

Cytokines GKN1

Cytokines GMFB

Cytokines GMFG

Cytokines GNRH1

Cytokines GNRH2

Cytokines GPHA2

Cytokines GPHB5

Cytokines GPI

Cytokines GREM1

Cytokines GREM2

Cytokines GRN

Cytokines GRP

Cytokines GUCA2A

Cytokines HAMP

Cytokines HBEGF

Cytokines HDGF

Cytokines HDGFRP3

Cytokines HGF

Cytokines HTN3

Cytokines IAPP

Cytokines IFNA1

Cytokines IFNA10

Cytokines IFNA13

Cytokines IFNA14

Cytokines IFNA16

Cytokines IFNA17

Cytokines IFNA2

Cytokines IFNA21

Cytokines IFNA4

Cytokines IFNA5

Cytokines IFNA6

Cytokines IFNA7

Cytokines IFNA8

Cytokines IFNB1

Cytokines IFNE

Cytokines IFNG

Cytokines IFNK

Cytokines IFNW1

Cytokines IGF1

Cytokines IGF2

Cytokines IL10

Cytokines IL11

Cytokines IL12A

Cytokines IL12B

Cytokines IL13

Cytokines IL15

Cytokines IL16

Cytokines IL17A

Cytokines IL17B

Cytokines IL17C

Cytokines IL17D

Cytokines IL17F

Cytokines IL18

Cytokines IL19

Cytokines IL1A

Cytokines IL1B

Cytokines IL1F10

Cytokines IL1F5

Cytokines IL1F6

Cytokines IL1F7

Cytokines IL1F8

Cytokines IL1F9

Cytokines IL1RN

Cytokines IL2

Cytokines IL20

Cytokines IL21

Cytokines IL22

Cytokines IL23A

Cytokines IL24

Cytokines IL25

Cytokines IL26

Cytokines IL27

Cytokines IL28A

Cytokines IL28B

Cytokines IL29

Cytokines IL3

Cytokines IL31

Cytokines IL32

Cytokines IL33

Cytokines IL34

Cytokines IL4

Cytokines IL5

Cytokines IL6

Cytokines IL6ST

Cytokines IL7

Cytokines IL8

Cytokines IL9

Cytokines INHA

Cytokines INHBA

Cytokines INHBB

Cytokines INHBC

Cytokines INHBE

Cytokines INS

Cytokines INS-IGF2

Cytokines INSL3

Cytokines INSL4

Cytokines INSL5

Cytokines INSL6

Cytokines JAG1

Cytokines JAG2

Cytokines KGFLP1

Cytokines KGFLP2

Cytokines KITLG

Cytokines KL

Cytokines LACRT

Cytokines LECT2

Cytokines LEFTY1

Cytokines LEFTY2

Cytokines LEP

Cytokines LHB

Cytokines LIF

Cytokines LRSAM1

Cytokines LTA

Cytokines LTB

Cytokines LTBP1

Cytokines LTBP2

Cytokines LTBP3

Cytokines LTBP4

Cytokines MDK

Cytokines MIA

Cytokines MIF

Cytokines MLN

Cytokines MSTN

Cytokines NAMPT

Cytokines NDP

Cytokines NENF

Cytokines NGF

Cytokines NMB

Cytokines NODAL

Cytokines NOV

Cytokines NPFF

Cytokines NPPA

Cytokines NPPB

Cytokines NPPC

Cytokines NPY

Cytokines NRG1

Cytokines NRG2

Cytokines NRG3

Cytokines NRG4

Cytokines NRTN

Cytokines NTF3

Cytokines NTF4

Cytokines NTS

Cytokines NUDT6

Cytokines OGN

Cytokines OSGIN1

Cytokines OSM

Cytokines OSTN

Cytokines OXT

Cytokines P11

Cytokines PDGFA

Cytokines PDGFB

Cytokines PDGFC

Cytokines PDGFD

Cytokines PDGFRA

Cytokines PDGFRB

Cytokines PDGFRL

Cytokines PDYN

Cytokines PENK

Cytokines PF4

Cytokines PF4V1

Cytokines PGF

Cytokines PLAU

Cytokines PMCH

Cytokines PNOC

Cytokines POMC

Cytokines PPBP

Cytokines PPBPL1

Cytokines PPBPL2

Cytokines PPY

Cytokines PRL

Cytokines PRLH

Cytokines PROK1

Cytokines PROK2

Cytokines PSPN

Cytokines PTH

Cytokines PTH2

Cytokines PTHLH

Cytokines PTN

Cytokines PYY

Cytokines QRFP

Cytokines RABEP1

Cytokines RABEP2

Cytokines REG1A

Cytokines RETN

Cytokines RETNLB

Cytokines RLN1

Cytokines RLN2

Cytokines RLN3

Cytokines RNASE2

Cytokines S100A6

Cytokines SAA1

Cytokines SAA2

Cytokines SBDS

Cytokines SCG2

Cytokines SCGB3A1

Cytokines SCT

Cytokines SCYE1

Cytokines SECTM1

Cytokines SEMA3A

Cytokines SEMA3B

Cytokines SEMA3C

Cytokines SEMA3D

Cytokines SEMA3E

Cytokines SEMA3F

Cytokines SEMA3G

Cytokines SEMA4A

Cytokines SEMA4B

Cytokines SEMA4C

Cytokines SEMA4D

Cytokines SEMA4F

Cytokines SEMA4G

Cytokines SEMA5A

Cytokines SEMA5B

Cytokines SEMA6A

Cytokines SEMA6B

Cytokines SEMA6C

Cytokines SEMA6D

Cytokines SEMA7A

Cytokines SLIT1

Cytokines SLIT2

Cytokines SLURP1

Cytokines SPP1

Cytokines SST

Cytokines STC1

Cytokines STC2

Cytokines TAC1

Cytokines TDGF1

Cytokines TDGF3

Cytokines TG

Cytokines TGFA

Cytokines TGFB1

Cytokines TGFB2

Cytokines TGFB3

Cytokines THPO

Cytokines TNC

Cytokines TNF

Cytokines TNFRSF11B

Cytokines TNFSF10

Cytokines TNFSF11

Cytokines TNFSF12

Cytokines TNFSF13

Cytokines TNFSF13B

Cytokines TNFSF14

Cytokines TNFSF15

Cytokines TNFSF18

Cytokines TNFSF4

Cytokines TNFSF8

Cytokines TNFSF9

Cytokines TOR2A

Cytokines TRH

Cytokines TSHB

Cytokines TSLP

Cytokines TXLNA

Cytokines TYMP

Cytokines UCN

Cytokines UCN2

Cytokines UCN3

Cytokines UTS2

Cytokines UTS2D

Cytokines VEGFA

Cytokines VEGFB

Cytokines VEGFC

Cytokines VGF

Cytokines VIP

Cytokines XCL1

Cytokines XCL2

Cytokine_Receptors ACVR1B

Cytokine_Receptors ACVR1C

Cytokine_Receptors ACVR2A

Cytokine_Receptors ACVR2B

Cytokine_Receptors ACVRL1

Cytokine_Receptors ADCYAP1R1

Cytokine_Receptors ADIPOR1

Cytokine_Receptors ADIPOR2

Cytokine_Receptors ADRB1

Cytokine_Receptors ADRB2

Cytokine_Receptors AGTR1

Cytokine_Receptors AGTR2

Cytokine_Receptors AMHR2

Cytokine_Receptors ANGPT1

Cytokine_Receptors ANGPT4

Cytokine_Receptors ANGPTL1

Cytokine_Receptors ANGPTL2

Cytokine_Receptors ANGPTL3

Cytokine_Receptors ANGPTL4

Cytokine_Receptors ANGPTL6

Cytokine_Receptors APLNR

Cytokine_Receptors AR

Cytokine_Receptors AVPR1A

Cytokine_Receptors AVPR1B

Cytokine_Receptors AVPR2

Cytokine_Receptors BMPR1A

Cytokine_Receptors BMPR1B

Cytokine_Receptors BMPR2

Cytokine_Receptors BRD8

Cytokine_Receptors C3AR1

Cytokine_Receptors C5AR1

Cytokine_Receptors CALCR

Cytokine_Receptors CALCRL

Cytokine_Receptors CCBP2

Cytokine_Receptors CCR1

Cytokine_Receptors CCR10

Cytokine_Receptors CCR3

Cytokine_Receptors CCR4

Cytokine_Receptors CCR5

Cytokine_Receptors CCR6

Cytokine_Receptors CCR7

Cytokine_Receptors CCR8

Cytokine_Receptors CCR9

Cytokine_Receptors CCRL1

Cytokine_Receptors CCRL2

Cytokine_Receptors CD40

Cytokine_Receptors CMKLR1

Cytokine_Receptors CNTFR

Cytokine_Receptors CRHR1

Cytokine_Receptors CRHR2

Cytokine_Receptors CRIM1

Cytokine_Receptors CRLF1

Cytokine_Receptors CRLF2

Cytokine_Receptors CRLF3

Cytokine_Receptors CSF1R

Cytokine_Receptors CSF2RA

Cytokine_Receptors CSF2RB

Cytokine_Receptors CSF3R

Cytokine_Receptors CX3CR1

Cytokine_Receptors CXCR3

Cytokine_Receptors CXCR4

Cytokine_Receptors CXCR5

Cytokine_Receptors CXCR6

Cytokine_Receptors CXCR7

Cytokine_Receptors CYSLTR1

Cytokine_Receptors CYSLTR2

Cytokine_Receptors DARC

Cytokine_Receptors EDNRA

Cytokine_Receptors EDNRB

Cytokine_Receptors EGFR

Cytokine_Receptors ENG

Cytokine_Receptors EPOR

Cytokine_Receptors ESR1

Cytokine_Receptors ESR2

Cytokine_Receptors ESRRA

Cytokine_Receptors ESRRB

Cytokine_Receptors ESRRG

Cytokine_Receptors FGFR1

Cytokine_Receptors FGFR2

Cytokine_Receptors FGFR3

Cytokine_Receptors FGFR4

Cytokine_Receptors FGFRL1

Cytokine_Receptors FLT1

Cytokine_Receptors FLT3

Cytokine_Receptors FLT4

Cytokine_Receptors FPR1

Cytokine_Receptors FPR2

Cytokine_Receptors FSHR

Cytokine_Receptors GALR2

Cytokine_Receptors GALR3

Cytokine_Receptors GCGR

Cytokine_Receptors GHR

Cytokine_Receptors GHRHR

Cytokine_Receptors GHSR

Cytokine_Receptors GIPR

Cytokine_Receptors GLP1R

Cytokine_Receptors GLP2R

Cytokine_Receptors GNRHR

Cytokine_Receptors GPER

Cytokine_Receptors GPR17

Cytokine_Receptors GPR32

Cytokine_Receptors GPR33

Cytokine_Receptors GPR44

Cytokine_Receptors GPR77

Cytokine_Receptors HNF4A

Cytokine_Receptors HNF4G

Cytokine_Receptors HTR3A

Cytokine_Receptors HTR3B

Cytokine_Receptors HTR3C

Cytokine_Receptors HTR3D

Cytokine_Receptors HTR3E

Cytokine_Receptors IFNAR1

Cytokine_Receptors IFNAR2

Cytokine_Receptors IFNGR1

Cytokine_Receptors IFNGR2

Cytokine_Receptors IGF1R

Cytokine_Receptors IGF2R

Cytokine_Receptors IL10RA

Cytokine_Receptors IL10RB

Cytokine_Receptors IL11RA

Cytokine_Receptors IL11RB

Cytokine_Receptors IL12RB1

Cytokine_Receptors IL12RB2

Cytokine_Receptors IL13RA1

Cytokine_Receptors IL13RA2

Cytokine_Receptors IL15RA

Cytokine_Receptors IL15RB

Cytokine_Receptors IL17RA

Cytokine_Receptors IL17RB

Cytokine_Receptors IL17RC

Cytokine_Receptors IL17RD

Cytokine_Receptors IL17RE

Cytokine_Receptors IL18R1

Cytokine_Receptors IL18RAP

Cytokine_Receptors IL1R1

Cytokine_Receptors IL1R2

Cytokine_Receptors IL1RAP

Cytokine_Receptors IL1RL1

Cytokine_Receptors IL1RL2

Cytokine_Receptors IL20RA

Cytokine_Receptors IL20RB

Cytokine_Receptors IL21R

Cytokine_Receptors IL22RA1

Cytokine_Receptors IL22RA2

Cytokine_Receptors IL23R

Cytokine_Receptors IL27RA

Cytokine_Receptors IL28RA

Cytokine_Receptors IL2RA

Cytokine_Receptors IL2RB

Cytokine_Receptors IL2RG

Cytokine_Receptors IL31RA

Cytokine_Receptors IL3RA

Cytokine_Receptors IL4R

Cytokine_Receptors IL5RA

Cytokine_Receptors IL6R

Cytokine_Receptors IL7R

Cytokine_Receptors IL8RA

Cytokine_Receptors IL8RB

Cytokine_Receptors IL9R

Cytokine_Receptors INSR

Cytokine_Receptors KDR

Cytokine_Receptors LEPR

Cytokine_Receptors LGR4

Cytokine_Receptors LGR5

Cytokine_Receptors LGR6

Cytokine_Receptors LHCGR

Cytokine_Receptors LIFR

Cytokine_Receptors LTB4R

Cytokine_Receptors LTB4R2

Cytokine_Receptors LTBR

Cytokine_Receptors MC1R

Cytokine_Receptors MC2R

Cytokine_Receptors MC3R

Cytokine_Receptors MC4R

Cytokine_Receptors MCHR1

Cytokine_Receptors MCHR2

Cytokine_Receptors MET

Cytokine_Receptors MLNR

Cytokine_Receptors MPL

Cytokine_Receptors MTNR1A

Cytokine_Receptors MTNR1B

Cytokine_Receptors NGFR

Cytokine_Receptors NMBR

Cytokine_Receptors NPR1

Cytokine_Receptors NPR3

Cytokine_Receptors NR0B1

Cytokine_Receptors NR0B2

Cytokine_Receptors NR1D1

Cytokine_Receptors NR1D2

Cytokine_Receptors NR1H2

Cytokine_Receptors NR1H3

Cytokine_Receptors NR1H4

Cytokine_Receptors NR1I2

Cytokine_Receptors NR1I3

Cytokine_Receptors NR2C1

Cytokine_Receptors NR2C2

Cytokine_Receptors NR2E1

Cytokine_Receptors NR2E3

Cytokine_Receptors NR2F1

Cytokine_Receptors NR2F2

Cytokine_Receptors NR2F6

Cytokine_Receptors NR3C1

Cytokine_Receptors NR3C2

Cytokine_Receptors NR4A1

Cytokine_Receptors NR4A2

Cytokine_Receptors NR4A3

Cytokine_Receptors NR5A1

Cytokine_Receptors NR5A2

Cytokine_Receptors NR6A1

Cytokine_Receptors NRP1

Cytokine_Receptors NRP2

Cytokine_Receptors OGFR

Cytokine_Receptors OPRD1

Cytokine_Receptors OPRK1

Cytokine_Receptors OPRL1

Cytokine_Receptors OPRM1

Cytokine_Receptors OSMR

Cytokine_Receptors OXTR

Cytokine_Receptors PGR

Cytokine_Receptors PGRMC2

Cytokine_Receptors PLAUR

Cytokine_Receptors PLXNA1

Cytokine_Receptors PLXNA2

Cytokine_Receptors PLXNA3

Cytokine_Receptors PLXNA4

Cytokine_Receptors PLXNB1

Cytokine_Receptors PLXNB2

Cytokine_Receptors PLXNB3

Cytokine_Receptors PLXNC1

Cytokine_Receptors PLXND1

Cytokine_Receptors PPARA

Cytokine_Receptors PPARD

Cytokine_Receptors PPARG

Cytokine_Receptors PRLHR

Cytokine_Receptors PRLR

Cytokine_Receptors PTAFR

Cytokine_Receptors PTGDR

Cytokine_Receptors PTGDS

Cytokine_Receptors PTGER1

Cytokine_Receptors PTGER2

Cytokine_Receptors PTGER3

Cytokine_Receptors PTGER4

Cytokine_Receptors PTGFR

Cytokine_Receptors PTH1R

Cytokine_Receptors PTH2R

Cytokine_Receptors RARA

Cytokine_Receptors RARB

Cytokine_Receptors RARG

Cytokine_Receptors ROBO1

Cytokine_Receptors ROBO2

Cytokine_Receptors ROBO3

Cytokine_Receptors RORA

Cytokine_Receptors RORB

Cytokine_Receptors RORC

Cytokine_Receptors RXFP1

Cytokine_Receptors RXFP2

Cytokine_Receptors RXFP3

Cytokine_Receptors RXRA

Cytokine_Receptors RXRB

Cytokine_Receptors RXRG

Cytokine_Receptors S1PR1

Cytokine_Receptors S1PR2

Cytokine_Receptors SCTR

Cytokine_Receptors SDC1

Cytokine_Receptors SDC2

Cytokine_Receptors SDC3

Cytokine_Receptors SDC4

Cytokine_Receptors SORT1

Cytokine_Receptors SSTR1

Cytokine_Receptors SSTR2

Cytokine_Receptors SSTR5

Cytokine_Receptors ST2

Cytokine_Receptors TACR1

Cytokine_Receptors TEK

Cytokine_Receptors TGFBR1

Cytokine_Receptors TGFBR2

Cytokine_Receptors TGFBR3

Cytokine_Receptors THRA

Cytokine_Receptors THRB

Cytokine_Receptors TIE1

Cytokine_Receptors TNFRSF10A

Cytokine_Receptors TNFRSF10B

Cytokine_Receptors TNFRSF10C

Cytokine_Receptors TNFRSF10D

Cytokine_Receptors TNFRSF11A

Cytokine_Receptors TNFRSF12A

Cytokine_Receptors TNFRSF13B

Cytokine_Receptors TNFRSF13C

Cytokine_Receptors TNFRSF14

Cytokine_Receptors TNFRSF17

Cytokine_Receptors TNFRSF18

Cytokine_Receptors TNFRSF19

Cytokine_Receptors TNFRSF1A

Cytokine_Receptors TNFRSF1B

Cytokine_Receptors TNFRSF21

Cytokine_Receptors TNFRSF25

Cytokine_Receptors TNFRSF4

Cytokine_Receptors TNFRSF6B

Cytokine_Receptors TNFRSF8

Cytokine_Receptors TNFRSF9

Cytokine_Receptors TRHR

Cytokine_Receptors TSHR

Cytokine_Receptors TUBB3

Cytokine_Receptors VDR

Cytokine_Receptors VIPR1

Cytokine_Receptors VIPR2

Cytokine_Receptors XCR1

Interferons IFNA10

Interferons IFNA13

Interferons IFNA14

Interferons IFNA16

Interferons IFNA17

Interferons IFNA2

Interferons IFNA21

Interferons IFNA4

Interferons IFNA5

Interferons IFNA6

Interferons IFNA7

Interferons IFNA8

Interferons IFNB1

Interferons IFNE

Interferons IFNG

Interferons IFNK

Interferons IFNW1

Interferon_Receptor IFNAR2

Interferon_Receptor IFNGR1

Interferon_Receptor IFNGR2

Interleukins IL11

Interleukins IL12A

Interleukins IL12B

Interleukins IL13

Interleukins IL15

Interleukins IL16

Interleukins IL17A

Interleukins IL17B

Interleukins IL17C

Interleukins IL17D

Interleukins IL17F

Interleukins IL18

Interleukins IL19

Interleukins IL1A

Interleukins IL1B

Interleukins IL1F10

Interleukins IL1F5

Interleukins IL1F6

Interleukins IL1F7

Interleukins IL1F8

Interleukins IL1F9

Interleukins IL1RN

Interleukins IL2

Interleukins IL20

Interleukins IL21

Interleukins IL22

Interleukins IL23A

Interleukins IL24

Interleukins IL25

Interleukins IL26

Interleukins IL27

Interleukins IL28A

Interleukins IL28B

Interleukins IL29

Interleukins IL3

Interleukins IL31

Interleukins IL32

Interleukins IL33

Interleukins IL34

Interleukins IL4

Interleukins IL5

Interleukins IL6

Interleukins IL6ST

Interleukins IL7

Interleukins IL8

Interleukins IL9

Interleukins TXLNA

Interleukins_Receptor IL10RA

Interleukins_Receptor IL10RB

Interleukins_Receptor IL11RA

Interleukins_Receptor IL11RB

Interleukins_Receptor IL12RB1

Interleukins_Receptor IL12RB2

Interleukins_Receptor IL13RA1

Interleukins_Receptor IL13RA2

Interleukins_Receptor IL15RA

Interleukins_Receptor IL15RB

Interleukins_Receptor IL17RA

Interleukins_Receptor IL17RB

Interleukins_Receptor IL17RC

Interleukins_Receptor IL17RD

Interleukins_Receptor IL17RE

Interleukins_Receptor IL18R1

Interleukins_Receptor IL18RAP

Interleukins_Receptor IL1R1

Interleukins_Receptor IL1R2

Interleukins_Receptor IL1RAP

Interleukins_Receptor IL1RL1

Interleukins_Receptor IL1RL2

Interleukins_Receptor IL20RA

Interleukins_Receptor IL20RB

Interleukins_Receptor IL21R

Interleukins_Receptor IL22RA1

Interleukins_Receptor IL22RA2

Interleukins_Receptor IL23R

Interleukins_Receptor IL27RA

Interleukins_Receptor IL28RA

Interleukins_Receptor IL2RA

Interleukins_Receptor IL2RB

Interleukins_Receptor IL2RG

Interleukins_Receptor IL31RA

Interleukins_Receptor IL3RA

Interleukins_Receptor IL4R

Interleukins_Receptor IL5RA

Interleukins_Receptor IL6R

Interleukins_Receptor IL7R

Interleukins_Receptor IL8RA

Interleukins_Receptor IL8RB

Interleukins_Receptor IL9R

Interleukins_Receptor ST2

NaturalKiller_Cell_Cytotoxicity HLA-A

NaturalKiller_Cell_Cytotoxicity HLA-B

NaturalKiller_Cell_Cytotoxicity HLA-C

NaturalKiller_Cell_Cytotoxicity HLA-E

NaturalKiller_Cell_Cytotoxicity HLA-G

NaturalKiller_Cell_Cytotoxicity KIR3DL1

NaturalKiller_Cell_Cytotoxicity KIR3DL2

NaturalKiller_Cell_Cytotoxicity KIR2DL1

NaturalKiller_Cell_Cytotoxicity KIR2DL2

NaturalKiller_Cell_Cytotoxicity KIR2DL3

NaturalKiller_Cell_Cytotoxicity KIR2DL4

NaturalKiller_Cell_Cytotoxicity KIR2DL5A

NaturalKiller_Cell_Cytotoxicity KLRC1

NaturalKiller_Cell_Cytotoxicity KLRC2

NaturalKiller_Cell_Cytotoxicity KLRC3

NaturalKiller_Cell_Cytotoxicity KLRD1

NaturalKiller_Cell_Cytotoxicity PTPN6

NaturalKiller_Cell_Cytotoxicity PTPN11

NaturalKiller_Cell_Cytotoxicity ICAM1

NaturalKiller_Cell_Cytotoxicity ICAM2

NaturalKiller_Cell_Cytotoxicity ITGAL

NaturalKiller_Cell_Cytotoxicity ITGB2

NaturalKiller_Cell_Cytotoxicity PTK2B

NaturalKiller_Cell_Cytotoxicity VAV3

NaturalKiller_Cell_Cytotoxicity VAV1

NaturalKiller_Cell_Cytotoxicity VAV2

NaturalKiller_Cell_Cytotoxicity RAC1

NaturalKiller_Cell_Cytotoxicity RAC2

NaturalKiller_Cell_Cytotoxicity RAC3

NaturalKiller_Cell_Cytotoxicity PAK1

NaturalKiller_Cell_Cytotoxicity MAP2K1

NaturalKiller_Cell_Cytotoxicity MAP2K2

NaturalKiller_Cell_Cytotoxicity MAPK1

NaturalKiller_Cell_Cytotoxicity MAPK3

NaturalKiller_Cell_Cytotoxicity TNF

NaturalKiller_Cell_Cytotoxicity CSF2

NaturalKiller_Cell_Cytotoxicity IFNG

NaturalKiller_Cell_Cytotoxicity KIR2DS1

NaturalKiller_Cell_Cytotoxicity KIR2DS3

NaturalKiller_Cell_Cytotoxicity KIR2DS4

NaturalKiller_Cell_Cytotoxicity KIR2DS5

NaturalKiller_Cell_Cytotoxicity NCR2

NaturalKiller_Cell_Cytotoxicity TYROBP

NaturalKiller_Cell_Cytotoxicity LCK

NaturalKiller_Cell_Cytotoxicity FCGR3A

NaturalKiller_Cell_Cytotoxicity FCGR3B

NaturalKiller_Cell_Cytotoxicity LOC652578

NaturalKiller_Cell_Cytotoxicity NCR1

NaturalKiller_Cell_Cytotoxicity NCR3

NaturalKiller_Cell_Cytotoxicity FCER1G

NaturalKiller_Cell_Cytotoxicity CD247

NaturalKiller_Cell_Cytotoxicity ZAP70

NaturalKiller_Cell_Cytotoxicity SYK

NaturalKiller_Cell_Cytotoxicity LCP2

NaturalKiller_Cell_Cytotoxicity LAT

NaturalKiller_Cell_Cytotoxicity PLCG1

NaturalKiller_Cell_Cytotoxicity PLCG2

NaturalKiller_Cell_Cytotoxicity SH3BP2

NaturalKiller_Cell_Cytotoxicity PIK3CA

NaturalKiller_Cell_Cytotoxicity PIK3CB

NaturalKiller_Cell_Cytotoxicity PIK3CD

NaturalKiller_Cell_Cytotoxicity PIK3CG

NaturalKiller_Cell_Cytotoxicity PIK3R5

NaturalKiller_Cell_Cytotoxicity PIK3R1

NaturalKiller_Cell_Cytotoxicity PIK3R2

NaturalKiller_Cell_Cytotoxicity PIK3R3

NaturalKiller_Cell_Cytotoxicity FYN

NaturalKiller_Cell_Cytotoxicity SHC2

NaturalKiller_Cell_Cytotoxicity SHC4

NaturalKiller_Cell_Cytotoxicity SHC3

NaturalKiller_Cell_Cytotoxicity SHC1

NaturalKiller_Cell_Cytotoxicity GRB2

NaturalKiller_Cell_Cytotoxicity SOS1

NaturalKiller_Cell_Cytotoxicity SOS2

NaturalKiller_Cell_Cytotoxicity HRAS

NaturalKiller_Cell_Cytotoxicity KRAS

NaturalKiller_Cell_Cytotoxicity NRAS

NaturalKiller_Cell_Cytotoxicity ARAF

NaturalKiller_Cell_Cytotoxicity BRAF

NaturalKiller_Cell_Cytotoxicity RAF1

NaturalKiller_Cell_Cytotoxicity MICA

NaturalKiller_Cell_Cytotoxicity MICB

NaturalKiller_Cell_Cytotoxicity ULBP3

NaturalKiller_Cell_Cytotoxicity ULBP2

NaturalKiller_Cell_Cytotoxicity ULBP1

NaturalKiller_Cell_Cytotoxicity KLRK1

NaturalKiller_Cell_Cytotoxicity HCST

NaturalKiller_Cell_Cytotoxicity CD48

NaturalKiller_Cell_Cytotoxicity CD244

NaturalKiller_Cell_Cytotoxicity PPP3CA

NaturalKiller_Cell_Cytotoxicity PPP3CB

NaturalKiller_Cell_Cytotoxicity PPP3CC

NaturalKiller_Cell_Cytotoxicity CHP

NaturalKiller_Cell_Cytotoxicity PPP3R1

NaturalKiller_Cell_Cytotoxicity PPP3R2

NaturalKiller_Cell_Cytotoxicity CHP2

NaturalKiller_Cell_Cytotoxicity NFAT5

NaturalKiller_Cell_Cytotoxicity NFATC1

NaturalKiller_Cell_Cytotoxicity NFATC2

NaturalKiller_Cell_Cytotoxicity NFATC3

NaturalKiller_Cell_Cytotoxicity NFATC4

NaturalKiller_Cell_Cytotoxicity PRKCA

NaturalKiller_Cell_Cytotoxicity PRKCB

NaturalKiller_Cell_Cytotoxicity PRKCG

NaturalKiller_Cell_Cytotoxicity SH2D1B

NaturalKiller_Cell_Cytotoxicity SH2D1A

NaturalKiller_Cell_Cytotoxicity IFNGR1

NaturalKiller_Cell_Cytotoxicity IFNGR2

NaturalKiller_Cell_Cytotoxicity IFNA1

NaturalKiller_Cell_Cytotoxicity IFNA2

NaturalKiller_Cell_Cytotoxicity IFNA4

NaturalKiller_Cell_Cytotoxicity IFNA5

NaturalKiller_Cell_Cytotoxicity IFNA6

NaturalKiller_Cell_Cytotoxicity IFNA7

NaturalKiller_Cell_Cytotoxicity IFNA8

NaturalKiller_Cell_Cytotoxicity IFNA10

NaturalKiller_Cell_Cytotoxicity IFNA13

NaturalKiller_Cell_Cytotoxicity IFNA14

NaturalKiller_Cell_Cytotoxicity IFNA16

NaturalKiller_Cell_Cytotoxicity IFNA17

NaturalKiller_Cell_Cytotoxicity IFNA21

NaturalKiller_Cell_Cytotoxicity IFNB1

NaturalKiller_Cell_Cytotoxicity IFNAR1

NaturalKiller_Cell_Cytotoxicity IFNAR2

NaturalKiller_Cell_Cytotoxicity TNFSF10

NaturalKiller_Cell_Cytotoxicity TNFRSF10D

NaturalKiller_Cell_Cytotoxicity TNFRSF10C

NaturalKiller_Cell_Cytotoxicity TNFRSF10B

NaturalKiller_Cell_Cytotoxicity TNFRSF10A

NaturalKiller_Cell_Cytotoxicity FASLG

NaturalKiller_Cell_Cytotoxicity FAS

NaturalKiller_Cell_Cytotoxicity GZMB

NaturalKiller_Cell_Cytotoxicity PRF1

NaturalKiller_Cell_Cytotoxicity CASP3

NaturalKiller_Cell_Cytotoxicity BID

TCRsignalingPathway CD3D

TCRsignalingPathway CD3E

TCRsignalingPathway CD3G

TCRsignalingPathway CD247

TCRsignalingPathway CD4

TCRsignalingPathway CD8A

TCRsignalingPathway CD8B

TCRsignalingPathway PTPRC

TCRsignalingPathway LCK

TCRsignalingPathway FYN

TCRsignalingPathway ZAP70

TCRsignalingPathway LCP2

TCRsignalingPathway LAT

TCRsignalingPathway ITK

TCRsignalingPathway TEC

TCRsignalingPathway NCK1

TCRsignalingPathway NCK2

TCRsignalingPathway VAV3

TCRsignalingPathway VAV1

TCRsignalingPathway VAV2

TCRsignalingPathway GRAP2

TCRsignalingPathway GRB2

TCRsignalingPathway PAK1

TCRsignalingPathway PAK2

TCRsignalingPathway PAK3

TCRsignalingPathway PAK4

TCRsignalingPathway PAK6

TCRsignalingPathway PAK7

TCRsignalingPathway RHOA

TCRsignalingPathway CDC42

TCRsignalingPathway PPP3CA

TCRsignalingPathway PPP3CB

TCRsignalingPathway PPP3CC

TCRsignalingPathway CHP

TCRsignalingPathway PPP3R1

TCRsignalingPathway PPP3R2

TCRsignalingPathway CHP2

TCRsignalingPathway NFAT5

TCRsignalingPathway NFATC1

TCRsignalingPathway NFATC2

TCRsignalingPathway NFATC3

TCRsignalingPathway NFATC4

TCRsignalingPathway SOS1

TCRsignalingPathway SOS2

TCRsignalingPathway HRAS

TCRsignalingPathway KRAS

TCRsignalingPathway NRAS

TCRsignalingPathway FOS

TCRsignalingPathway JUN

TCRsignalingPathway CARD11

TCRsignalingPathway BCL10

TCRsignalingPathway MALT1

TCRsignalingPathway CHUK

TCRsignalingPathway IKBKB

TCRsignalingPathway IKBKG

TCRsignalingPathway NFKB1

TCRsignalingPathway RELA

TCRsignalingPathway NFKBIA

TCRsignalingPathway NFKBIB

TCRsignalingPathway NFKBIE

TCRsignalingPathway CD28

TCRsignalingPathway ICOS

TCRsignalingPathway CD40LG

TCRsignalingPathway PIK3R5

TCRsignalingPathway PIK3R1

TCRsignalingPathway PIK3R2

TCRsignalingPathway PIK3R3

TCRsignalingPathway PIK3CA

TCRsignalingPathway PIK3CB

TCRsignalingPathway PIK3CD

TCRsignalingPathway PIK3CG

TCRsignalingPathway AKT3

TCRsignalingPathway AKT1

TCRsignalingPathway AKT2

TCRsignalingPathway MAP3K8

TCRsignalingPathway MAP3K14

TCRsignalingPathway PDCD1

TCRsignalingPathway CTLA4

TCRsignalingPathway PTPN6

TCRsignalingPathway CBLC

TCRsignalingPathway CBL

TCRsignalingPathway CBLB

TCRsignalingPathway IL2

TCRsignalingPathway IL4

TCRsignalingPathway IL5

TCRsignalingPathway IL10

TCRsignalingPathway IFNG

TCRsignalingPathway CSF2

TCRsignalingPathway TNF

TCRsignalingPathway CDK4

TCRsignalingPathway RASGRP1

TCRsignalingPathway PDK1

TCRsignalingPathway PLCG1

TCRsignalingPathway PRKCQ

TCRsignalingPathway TRAC

TCRsignalingPathway TRAJ1

TCRsignalingPathway TRAJ2

TCRsignalingPathway TRAJ3

TCRsignalingPathway TRAJ4

TCRsignalingPathway TRAJ5

TCRsignalingPathway TRAJ6

TCRsignalingPathway TRAJ7

TCRsignalingPathway TRAJ8

TCRsignalingPathway TRAJ9

TCRsignalingPathway TRAJ10

TCRsignalingPathway TRAJ11

TCRsignalingPathway TRAJ12

TCRsignalingPathway TRAJ13

TCRsignalingPathway TRAJ14

TCRsignalingPathway TRAJ15

TCRsignalingPathway TRAJ16

TCRsignalingPathway TRAJ17

TCRsignalingPathway TRAJ18

TCRsignalingPathway TRAJ19

TCRsignalingPathway TRAJ20

TCRsignalingPathway TRAJ21

TCRsignalingPathway TRAJ22

TCRsignalingPathway TRAJ23

TCRsignalingPathway TRAJ24

TCRsignalingPathway TRAJ25

TCRsignalingPathway TRAJ26

TCRsignalingPathway TRAJ27

TCRsignalingPathway TRAJ28

TCRsignalingPathway TRAJ29

TCRsignalingPathway TRAJ30

TCRsignalingPathway TRAJ31

TCRsignalingPathway TRAJ32

TCRsignalingPathway TRAJ33

TCRsignalingPathway TRAJ34

TCRsignalingPathway TRAJ35

TCRsignalingPathway TRAJ36

TCRsignalingPathway TRAJ37

TCRsignalingPathway TRAJ38

TCRsignalingPathway TRAJ39

TCRsignalingPathway TRAJ40

TCRsignalingPathway TRAJ41

TCRsignalingPathway TRAJ42

TCRsignalingPathway TRAJ43

TCRsignalingPathway TRAJ44

TCRsignalingPathway TRAJ45

TCRsignalingPathway TRAJ46

TCRsignalingPathway TRAJ47

TCRsignalingPathway TRAJ48

TCRsignalingPathway TRAJ49

TCRsignalingPathway TRAJ50

TCRsignalingPathway TRAJ52

TCRsignalingPathway TRAJ53

TCRsignalingPathway TRAJ54

TCRsignalingPathway TRAJ56

TCRsignalingPathway TRAJ57

TCRsignalingPathway TRAJ58

TCRsignalingPathway TRAJ59

TCRsignalingPathway TRAJ61

TCRsignalingPathway TRAV1-1

TCRsignalingPathway TRAV1-2

TCRsignalingPathway TRAV2

TCRsignalingPathway TRAV3

TCRsignalingPathway TRAV4

TCRsignalingPathway TRAV5

TCRsignalingPathway TRAV7

TCRsignalingPathway TRAV8-1

TCRsignalingPathway TRAV8-2

TCRsignalingPathway TRAV8-3

TCRsignalingPathway TRAV8-4

TCRsignalingPathway TRAV8-6

TCRsignalingPathway TRAV8-7

TCRsignalingPathway TRAV9-1

TCRsignalingPathway TRAV9-2

TCRsignalingPathway TRAV10

TCRsignalingPathway TRAV12-1

TCRsignalingPathway TRAV12-2

TCRsignalingPathway TRAV12-3

TCRsignalingPathway TRAV13-1

TCRsignalingPathway TRAV13-2

TCRsignalingPathway TRAV14DV4

TCRsignalingPathway TRAV16

TCRsignalingPathway TRAV17

TCRsignalingPathway TRAV18

TCRsignalingPathway TRAV19

TCRsignalingPathway TRAV20

TCRsignalingPathway TRAV21

TCRsignalingPathway TRAV22

TCRsignalingPathway TRAV23DV6

TCRsignalingPathway TRAV24

TCRsignalingPathway TRAV25

TCRsignalingPathway TRAV26-1

TCRsignalingPathway TRAV26-2

TCRsignalingPathway TRAV27

TCRsignalingPathway TRAV29DV5

TCRsignalingPathway TRAV30

TCRsignalingPathway TRAV34

TCRsignalingPathway TRAV35

TCRsignalingPathway TRAV36DV7

TCRsignalingPathway TRAV38-1

TCRsignalingPathway TRAV38-2DV8

TCRsignalingPathway TRAV39

TCRsignalingPathway TRAV40

TCRsignalingPathway TRAV41

TCRsignalingPathway TRBC1

TCRsignalingPathway TRBC2

TCRsignalingPathway TRBD1

TCRsignalingPathway TRBD2

TCRsignalingPathway TRBJ1-1

TCRsignalingPathway TRBJ1-2

TCRsignalingPathway TRBJ1-3

TCRsignalingPathway TRBJ1-4

TCRsignalingPathway TRBJ1-5

TCRsignalingPathway TRBJ1-6

TCRsignalingPathway TRBJ2-1

TCRsignalingPathway TRBJ2-2

TCRsignalingPathway TRBJ2-3

TCRsignalingPathway TRBJ2-4

TCRsignalingPathway TRBJ2-5

TCRsignalingPathway TRBJ2-6

TCRsignalingPathway TRBJ2-7

TCRsignalingPathway TRBV2

TCRsignalingPathway TRBV3-1

TCRsignalingPathway TRBV4-1

TCRsignalingPathway TRBV4-2

TCRsignalingPathway TRBV4-3

TCRsignalingPathway TRBV5-1

TCRsignalingPathway TRBV5-4

TCRsignalingPathway TRBV5-5

TCRsignalingPathway TRBV5-6

TCRsignalingPathway TRBV5-7

TCRsignalingPathway TRBV5-8

TCRsignalingPathway TRBV6-1

TCRsignalingPathway TRBV6-2

TCRsignalingPathway TRBV6-3

TCRsignalingPathway TRBV6-4

TCRsignalingPathway TRBV6-5

TCRsignalingPathway TRBV6-6

TCRsignalingPathway TRBV6-7

TCRsignalingPathway TRBV6-8

TCRsignalingPathway TRBV6-9

TCRsignalingPathway TRBV7-2

TCRsignalingPathway TRBV7-3

TCRsignalingPathway TRBV7-4

TCRsignalingPathway TRBV7-6

TCRsignalingPathway TRBV7-7

TCRsignalingPathway TRBV7-8

TCRsignalingPathway TRBV7-9

TCRsignalingPathway TRBV9

TCRsignalingPathway TRBV10-1

TCRsignalingPathway TRBV10-2

TCRsignalingPathway TRBV10-3

TCRsignalingPathway TRBV11-1

TCRsignalingPathway TRBV11-2

TCRsignalingPathway TRBV11-3

TCRsignalingPathway TRBV12-3

TCRsignalingPathway TRBV12-4

TCRsignalingPathway TRBV12-5

TCRsignalingPathway TRBV13

TCRsignalingPathway TRBV14

TCRsignalingPathway TRBV15

TCRsignalingPathway TRBV16

TCRsignalingPathway TRBV17

TCRsignalingPathway TRBV18

TCRsignalingPathway TRBV19

TCRsignalingPathway TRBV20-1

TCRsignalingPathway TRBV24-1

TCRsignalingPathway TRBV25-1

TCRsignalingPathway TRBV27

TCRsignalingPathway TRBV28

TCRsignalingPathway TRBV29-1

TCRsignalingPathway TRBV30

TCRsignalingPathway TRDC

TCRsignalingPathway TRDD1

TCRsignalingPathway TRDD2

TCRsignalingPathway TRDD3

TCRsignalingPathway TRDJ1

TCRsignalingPathway TRDJ2

TCRsignalingPathway TRDJ3

TCRsignalingPathway TRDJ4

TCRsignalingPathway TRDV1

TCRsignalingPathway TRDV2

TCRsignalingPathway TRDV3

TCRsignalingPathway TRGV9

TCRsignalingPathway TRGV8

TCRsignalingPathway TRGV5

TCRsignalingPathway TRGV4

TCRsignalingPathway TRGV3

TCRsignalingPathway TRGV2

TCRsignalingPathway TRGJP2

TCRsignalingPathway TRGJP1

TCRsignalingPathway TRGJP

TCRsignalingPathway TRGJ2

TCRsignalingPathway TRGJ1

TCRsignalingPathway TRGC2

TCRsignalingPathway TRGC1

TCRsignalingPathway TRAV6

TGFb_Family_Member BMP1

TGFb_Family_Member BMP10

TGFb_Family_Member BMP15

TGFb_Family_Member BMP2

TGFb_Family_Member BMP3

TGFb_Family_Member BMP4

TGFb_Family_Member BMP5

TGFb_Family_Member BMP6

TGFb_Family_Member BMP7

TGFb_Family_Member BMP8A

TGFb_Family_Member BMP8B

TGFb_Family_Member GDF1

TGFb_Family_Member GDF10

TGFb_Family_Member GDF11

TGFb_Family_Member GDF15

TGFb_Family_Member GDF2

TGFb_Family_Member GDF3

TGFb_Family_Member GDF5

TGFb_Family_Member GDF6

TGFb_Family_Member GDF7

TGFb_Family_Member GDF9

TGFb_Family_Member GDNF

TGFb_Family_Member INHA

TGFb_Family_Member INHBA

TGFb_Family_Member INHBB

TGFb_Family_Member INHBC

TGFb_Family_Member INHBE

TGFb_Family_Member LEFTY1

TGFb_Family_Member LEFTY2

TGFb_Family_Member NODAL

TGFb_Family_Member TGFB1

TGFb_Family_Member TGFB2

TGFb_Family_Member TGFB3

TGFb_Family_Member_Receptor ACVR1B

TGFb_Family_Member_Receptor ACVR1C

TGFb_Family_Member_Receptor ACVR2A

TGFb_Family_Member_Receptor ACVR2B

TGFb_Family_Member_Receptor ACVRL1

TGFb_Family_Member_Receptor AMHR2

TGFb_Family_Member_Receptor BMPR1A

TGFb_Family_Member_Receptor BMPR1B

TGFb_Family_Member_Receptor BMPR2

TGFb_Family_Member_Receptor TGFBR1

TGFb_Family_Member_Receptor TGFBR2

TGFb_Family_Member_Receptor TGFBR3

TNF_Family_Members TNFRSF11B

TNF_Family_Members TNFSF10

TNF_Family_Members TNFSF11

TNF_Family_Members TNFSF12

TNF_Family_Members TNFSF13

TNF_Family_Members TNFSF13B

TNF_Family_Members TNFSF14

TNF_Family_Members TNFSF15

TNF_Family_Members TNFSF18

TNF_Family_Members TNFSF4

TNF_Family_Members TNFSF8

TNF_Family_Members TNFSF9

TNF_Family_Members_Receptors TNFRSF10B

TNF_Family_Members_Receptors TNFRSF10C

TNF_Family_Members_Receptors TNFRSF10D

TNF_Family_Members_Receptors TNFRSF11A

TNF_Family_Members_Receptors TNFRSF12A

TNF_Family_Members_Receptors TNFRSF13B

TNF_Family_Members_Receptors TNFRSF13C

TNF_Family_Members_Receptors TNFRSF14

TNF_Family_Members_Receptors TNFRSF17

TNF_Family_Members_Receptors TNFRSF18

TNF_Family_Members_Receptors TNFRSF19

TNF_Family_Members_Receptors TNFRSF1A

TNF_Family_Members_Receptors TNFRSF1B

TNF_Family_Members_Receptors TNFRSF21

TNF_Family_Members_Receptors TNFRSF25

TNF_Family_Members_Receptors TNFRSF4

TNF_Family_Members_Receptors TNFRSF6B

TNF_Family_Members_Receptors TNFRSF8

TNF_Family_Members_Receptors TNFRSF9

T_cells_MCPcounter CD28

T_cells_MCPcounter CD3D

T_cells_MCPcounter CD3G

T_cells_MCPcounter CD5

T_cells_MCPcounter CD6

T_cells_MCPcounter CHRM3-AS2

T_cells_MCPcounter CTLA4

T_cells_MCPcounter FLT3LG

T_cells_MCPcounter ICOS

T_cells_MCPcounter MAL

T_cells_MCPcounter MGC40069

T_cells_MCPcounter PBX4

T_cells_MCPcounter SIRPG

T_cells_MCPcounter THEMIS

T_cells_MCPcounter TNFRSF25

T_cells_MCPcounter TRAT1

CD8_T_cells_MCPcounter CD8B

Cytotoxic_lymphocytes_MCPcounter CD8A

Cytotoxic_lymphocytes_MCPcounter EOMES

Cytotoxic_lymphocytes_MCPcounter FGFBP2

Cytotoxic_lymphocytes_MCPcounter GNLY

Cytotoxic_lymphocytes_MCPcounter KLRC3

Cytotoxic_lymphocytes_MCPcounter KLRC4

Cytotoxic_lymphocytes_MCPcounter KLRD1

B_lineage_MCPcounter BANK1

B_lineage_MCPcounter CD19

B_lineage_MCPcounter CD22

B_lineage_MCPcounter CD79A

B_lineage_MCPcounter CR2

B_lineage_MCPcounter FCRL2

B_lineage_MCPcounter IGKC

B_lineage_MCPcounter MS4A1

B_lineage_MCPcounter PAX5

NK_cells_MCPcounter CD160

NK_cells_MCPcounter KIR2DL1

NK_cells_MCPcounter KIR2DL3

NK_cells_MCPcounter KIR2DL4

NK_cells_MCPcounter KIR3DL1

NK_cells_MCPcounter KIR3DS1

NK_cells_MCPcounter NCR1

NK_cells_MCPcounter PTGDR

NK_cells_MCPcounter SH2D1B

Monocytic_lineage_MCPcounter ADAP2

Monocytic_lineage_MCPcounter CSF1R

Monocytic_lineage_MCPcounter FPR3

Monocytic_lineage_MCPcounter KYNU

Monocytic_lineage_MCPcounter PLA2G7

Monocytic_lineage_MCPcounter RASSF4

Monocytic_lineage_MCPcounter TFEC

Myeloid_dendritic_cells_MCPcounter CD1A

Myeloid_dendritic_cells_MCPcounter CD1B

Myeloid_dendritic_cells_MCPcounter CD1E

Myeloid_dendritic_cells_MCPcounter CLEC10A

Myeloid_dendritic_cells_MCPcounter CLIC2

Myeloid_dendritic_cells_MCPcounter WFDC21P

Neutrophils_MCPcounter CA4

Neutrophils_MCPcounter CEACAM3

Neutrophils_MCPcounter CXCR1

Neutrophils_MCPcounter CXCR2

Neutrophils_MCPcounter CYP4F3

Neutrophils_MCPcounter FCGR3B

Neutrophils_MCPcounter HAL

Neutrophils_MCPcounter KCNJ15

Neutrophils_MCPcounter MEGF9

Neutrophils_MCPcounter SLC25A37

Neutrophils_MCPcounter STEAP4

Neutrophils_MCPcounter TECPR2

Neutrophils_MCPcounter TLE3

Neutrophils_MCPcounter TNFRSF10C

Neutrophils_MCPcounter VNN3

Endothelial_cells_MCPcounter ACVRL1

Endothelial_cells_MCPcounter APLN

Endothelial_cells_MCPcounter BCL6B

Endothelial_cells_MCPcounter BMP6

Endothelial_cells_MCPcounter BMX

Endothelial_cells_MCPcounter CDH5

Endothelial_cells_MCPcounter CLEC14A

Endothelial_cells_MCPcounter CXorf36

Endothelial_cells_MCPcounter EDN1

Endothelial_cells_MCPcounter ELTD1

Endothelial_cells_MCPcounter EMCN

Endothelial_cells_MCPcounter ESAM

Endothelial_cells_MCPcounter ESM1

Endothelial_cells_MCPcounter FAM124B

Endothelial_cells_MCPcounter HECW2

Endothelial_cells_MCPcounter HHIP

Endothelial_cells_MCPcounter KDR

Endothelial_cells_MCPcounter MMRN1

Endothelial_cells_MCPcounter MMRN2

Endothelial_cells_MCPcounter MYCT1

Endothelial_cells_MCPcounter PALMD

Endothelial_cells_MCPcounter PEAR1

Endothelial_cells_MCPcounter PGF

Endothelial_cells_MCPcounter PLXNA2

Endothelial_cells_MCPcounter PTPRB

Endothelial_cells_MCPcounter ROBO4

Endothelial_cells_MCPcounter SDPR

Endothelial_cells_MCPcounter SHANK3

Endothelial_cells_MCPcounter SHE

Endothelial_cells_MCPcounter TEK

Endothelial_cells_MCPcounter TIE1

Endothelial_cells_MCPcounter VEPH1

Endothelial_cells_MCPcounter VWF

Fibroblasts_MCPcounter COL1A1

Fibroblasts_MCPcounter COL3A1

Fibroblasts_MCPcounter COL6A1

Fibroblasts_MCPcounter COL6A2

Fibroblasts_MCPcounter DCN

Fibroblasts_MCPcounter GREM1

Fibroblasts_MCPcounter PAMR1

Fibroblasts_MCPcounter TAGLN

T_cell_infiltration_1 CD8A

T_cell_infiltration_1 CCL2

T_cell_infiltration_1 CCL3

T_cell_infiltration_1 CCL4

T_cell_infiltration_1 CXCL9

T_cell_infiltration_1 CXCL10

T_cell_infiltration_1 ICOS

T_cell_infiltration_1 GZMK

T_cell_infiltration_1 IRF1

T_cell_infiltration_1 HLA-DMA

T_cell_infiltration_1 HLA-DMB

T_cell_infiltration_1 HLA-DOA

T_cell_infiltration_1 HLA-DOB

T_cell_infiltration_2 CD8B

T_cell_infiltration_2 CCL4

T_cell_infiltration_2 CCL5

T_cell_infiltration_2 CXCL9

T_cell_infiltration_2 XCL2

T_cell_infiltration_2 CXCL10

T_cell_infiltration_2 CXCL11

T_cell_infiltration_2 CXCL13

T_cell_infiltration_2 CCL2

T_cell_infiltration_2 CCL19

T_cell_infiltration_2 CCL21
